# Supplementary material for: Challenges in the medical oxygen ecosystem of Peru: A political economy analysis
Source: PLOS Glob Public Health. 2025 Dec 19;5(12):e0005667. doi: 10.1371/journal.pgph.0005667 (PMC12716698; doi:10.1371/journal.pgph.0005667)
Supplement: S2 Appendix — (DOCX) [file pgph.0005667.s002.docx]

1. Medline

| Interface: Ovid MEDLINE(R) and Epub Ahead of Print, In-Process & Other Non-Indexed Citations and Daily  Date of Search: 31 March 2023  Number of hits: 3620  Comment: In Ovid, two or more words are automatically searched as phrases; i.e. no quotation marks are needed | Field labels   - exp/ = exploded MeSH term - / = non exploded MeSH term - .ti,ab,kf. = title, abstract and author keywords - adjx = within x words, regardless of order - * = truncation of word for alternate endings |
| --- | --- |
| Ovid MEDLINE(R) ALL <1946 to March 30, 2023>   \| 1 \| exp Oxygen Inhalation Therapy/ \| 28246 \| \| --- \| --- \| --- \| \| 2 \| exp Oximetry/ \| 16591 \| \| 3 \| Oxygen/sd \| 107 \| \| 4 \| Oxygen/st \| 32 \| \| 5 \| Oxygen/tu \| 3680 \| \| 6 \| oxygen*.ti,ab,kf. \| 671667 \| \| 7 \| oximet*.ti,ab,kf. \| 16396 \| \| 8 \| Medical oxygen.ti,ab,kf. \| 116 \| \| 9 \| or/1-8 \| 691298 \| \| 10 \| COVID-19/ or exp COVID-19 Testing/ or COVID-19 Vaccines/ or SARS-CoV-2/ \| 222928 \| \| 11 \| (coronavirus/ or betacoronavirus/ or coronavirus infections/) and (disease outbreaks/ or epidemics/ or pandemics/) \| 40224 \| \| 12 \| (nCoV* or 2019nCoV or 19nCoV or COVID19* or COVID or SARS-COV-2 or SARSCOV-2 or SARS-COV2 or SARSCOV2 or SARS coronavirus 2 or Severe Acute Respiratory Syndrome Coronavirus 2 or Severe Acute Respiratory Syndrome Corona Virus 2).ti,ab,kf. \| 335614 \| \| 13 \| (longCOVID* or postCOVID* or postcoronavirus* or postSARS*).ti,ab,kf. \| 81 \| \| 14 \| ((coronavirus* or corona virus* or betacoronavirus*) adj3 (pandemic* or epidemic* or outbreak* or crisis)).ti,ab,kf. \| 15253 \| \| 15 \| or/10-14 \| 347854 \| \| 16 \| 9 or 15 \| 1029755 \| \| 17 \| Sweden/ \| 81195 \| \| 18 \| (sweden* or swedish).ti,ab,kf. \| 84949 \| \| 19 \| Bangladesh/ \| 14154 \| \| 20 \| bangladesh*.ti,ab,kf. \| 20510 \| \| 21 \| Malawi/ \| 6627 \| \| 22 \| (Malawi* or Nyasaland).ti,ab,kf. \| 9360 \| \| 23 \| Nigeria/ \| 33632 \| \| 24 \| Nigeria*.ti,ab,kf. \| 42246 \| \| 25 \|  \| 10654 \| \| 26 \| (peru or peruv*).ti,ab,kf. \| 17061 \| \| 27 \| exp India/ \| 117961 \| \| 28 \| india*.ti,ab,kf. \| 202744 \| \| 29 \| or/17-28 \| 448196 \| \| 30 \| exp "Health Care Economics and Organizations"/ \| 1677889 \| \| 31 \| exp Guidelines as Topic/ \| 172708 \| \| 32 \| exp Legislation as Topic/ \| 174737 \| \| 33 \| exp Policy/ \| 191489 \| \| 34 \| Decision Making/ \| 104139 \| \| 35 \| Decision Making, Shared/ \| 1853 \| \| 36 \| Decision Making, Organizational/ \| 11238 \| \| 37 \| exp Industry/ \| 349838 \| \| 38 \| exp Health Planning/ or Delivery of Health Care/ \| 463497 \| \| 39 \| (policy* or policies).ti,ab,kf. \| 361907 \| \| 40 \| (legislat* or law*).ti,ab,kf. \| 200096 \| \| 41 \| (social control or regulation* or guideline*).ti,ab,kf. \| 1493264 \| \| 42 \| decision making.ti,ab,kf. \| 189918 \| \| 43 \| ((drug or health or healthcare or health care or medical) adj2 (sector* or industr* or market* or financ* or planning* or program* or reform*)).ti,ab,kf. \| 142218 \| \| 44 \| or/30-43 \| 3980854 \| \| 45 \| 16 and 29 and 44 \| 3620 \| | |

2. Embase

| Interface: embase.com  Date of Search: 3 April 2023  Number of hits: 3953  Comment: Emtree is the controlled vocabulary in Embase | Field labels   - /exp = exploded Emtree term - /de = non exploded Emtree term - ti,ab,kw = title, abstract and author keywords - NEAR/x = within x words, regardless of order - * = truncation of word for alternate endings |
| --- | --- |
| No. Query Results   \| #41 \| #13 AND #27 AND #40 \| 3953 \| \| --- \| --- \| --- \| \| #40 \| #28 OR #29 OR #30 OR #31 OR #32 OR #33 OR #34 OR #35 OR #36 OR #37 OR #38 OR #39 \| 561915 \| \| #39 \| india*:ti,ab,kw \| 277211 \| \| #38 \| 'india'/exp OR 'indian'/de \| 219130 \| \| #37 \| peru:ti,ab,kw OR peruv*:ti,ab,kw \| 21910 \| \| #36 \| 'peru'/de OR 'peruvian'/de \| 16326 \| \| #35 \| nigeria*:ti,ab,kw \| 52676 \| \| #34 \| 'nigeria'/de OR 'nigerian'/de \| 47503 \| \| #33 \| malawi*:ti,ab,kw OR nyasaland:ti,ab,kw \| 10935 \| \| #32 \| 'malawi'/de OR 'malawian'/de \| 9772 \| \| #31 \| bangladesh*:ti,ab,kw \| 24417 \| \| #30 \| 'bangladesh'/de OR 'bangladeshi'/de \| 21929 \| \| #29 \| sweden*:ti,ab,kw OR swedish:ti,ab,kw \| 110823 \| \| #28 \| ('sweden'/de OR swedish) AND 'citizen'/de \| 0 \| \| #27 \| #14 OR #15 OR #16 OR #17 OR #18 OR #19 OR #20 OR #21 OR #22 OR #23 OR #24 OR #25 OR #26 \| 4226178 \| \| #26 \| ((drug OR health OR healthcare OR 'health care' OR medical) NEAR/2 (sector* OR industr* OR market* OR financ* OR planning* OR program* OR reform*)):ti,ab,kw \| 173477 \| \| #25 \| 'decision making':ti,ab,kw \| 259190 \| \| #24 \| 'social control':ti,ab,kw OR regulation*:ti,ab,kw OR guideline*:ti,ab,kw \| 1988646 \| \| #23 \| legislat*:ti,ab,kw OR law*:ti,ab,kw \| 238114 \| \| #22 \| policy*:ti,ab,kw OR policies:ti,ab,kw \| 424491 \| \| #21 \| 'health care delivery'/de \| 206325 \| \| #20 \| 'health care planning'/exp \| 112231 \| \| #19 \| 'industry'/exp \| 365675 \| \| #18 \| 'decision making'/exp \| 455296 \| \| #17 \| 'policy'/exp \| 325726 \| \| #16 \| 'law'/exp \| 123509 \| \| #15 \| 'practice guideline'/de \| 533695 \| \| #14 \| 'health care cost'/exp \| 334863 \| \| #13 \| #6 OR #12 \| 785625 \| \| #12 \| #/ OR #8 OR #9 OR #10 OR #11 \| 402215 \| \| #11 \| ((coronavirus* OR 'corona virus*' OR betacoronavirus*) NEAR/3 (pandemic* OR epidemic* OR outbreak* OR crisis)):ti,ab,kw \| 15164 \| \| #10 \| longcovid*:ti,ab,kw OR postcovid*:ti,ab,kw OR postcoronavirus*:ti,ab,kw OR postsars*:ti,ab,kw \| 7747 \| \| #9 \| ncov*:ti,ab,kw OR 2019ncov:ti,ab,kw OR 19ncov:ti,ab,kw OR covid19*:ti,ab,kw OR covid:ti,ab,kw OR 'sars cov 2':ti,ab,kw OR 'sarscov 2':ti,ab,kw OR 'sars cov2':ti,ab,kw OR sarscov2:ti,ab,kw OR 'sars coronavirus 2':ti,ab,kw OR 'severe acute respiratory syndrome coronavirus 2':ti,ab,kw OR 'severe acute respiratory syndrome corona virus 2':ti,ab,kw \| 369309 \| \| #8 \| ('coronavirinae'/de OR 'betacoronavirus'/de OR 'coronavirus infection'/de) AND ('epidemic'/de OR 'pandemic'/de) \| 11515 \| \| #7 \| 'coronavirus disease 2019'/de OR 'covid-19 testing'/exp OR 'sars-cov-2 vaccine'/de OR 'severe acute respiratory syndrome coronavirus 2'/de \| 322811 \| \| #6 \| #1 OR #2 OR #3 OR #4 OR #5 \| 400226 \| \| #5 \| oximet*:ti,ab,kw \| 23791 \| \| #4 \| oxygen*:ti,ab,kw \| 821258 \| \| #3 \| 'oxygen'/de \| 257430 \| \| #2 \| 'oximetry'/exp \| 35192 \| \| #1 \| 'oxygen therapy'/exp \| 104359 \| | |

4. Web of Science Core Collection

| Interface: Clarivate Analytics  Editions = A&HCI , ESCI , SCI-EXPANDED , SSCI  Date of Search: 3 April 2023  Number of hits: 4290 | Field labels   - TS/Topic = title, abstract, author keywords and Keywords Plus - NEAR/x = within x words, regardless of order - * = truncation of word for alternate endings   Note: the *Exact search*-function was used for all the searches |
| --- | --- |
| # Search Query Results   \| 1 \| "TS=(oxygen*)" \| 1360186 \| \| --- \| --- \| --- \| \| 2 \| "TS=oximet*" \| 17384 \| \| 3 \| "#1 OR #2" \| 1367747 \| \| 4 \| "TS=(nCoV* OR 2019nCoV OR 19nCoV OR COVID19* OR COVID OR SARS-COV-2 OR SARSCOV-2 OR SARS-COV2 OR SARSCOV2 OR ""SARS coronavirus 2"" OR ""Severe Acute Respiratory Syndrome Coronavirus 2"" OR ""Severe Acute Respiratory Syndrome Corona Virus 2"" )" \| 410468 \| \| 5 \| "TS=(longCOVID* OR postCOVID* OR postcoronavirus* OR postSARS* )" \| 187 \| \| 6 \| "TS=((coronavirus* OR ""corona virus*"" OR betacoronavirus* ) NEAR/2 (pandemic* OR epidemic* OR outbreak* OR crisis ))" \| 19278 \| \| 7 \| "#4 OR #5 OR #6" \| 414464 \| \| 8 \| "#3 OR #7" \| 1773100 \| \| 9 \| "TS=(policy* OR policies )" \| 1104049 \| \| 10 \| "TS=(legislat* OR law* )" \| 872196 \| \| 11 \| "TS=(""social control"" OR regulation* OR guideline* )" \| 2070976 \| \| 12 \| "TS=""decision making""" \| 414003 \| \| 13 \| "TS=((drug OR health OR healthcare OR ""health care"" OR medical ) NEAR/1 (sector* OR industr* OR market* OR financ* OR planning* OR program* OR reform* ))" \| 131861 \| \| 14 \| "#9 OR #10 OR #11 OR #12 OR #13" \| 4298656 \| \| 15 \| "TS=(sweden* OR swedish )" \| 152988 \| \| 16 \| "TS=bangladesh*" \| 41142 \| \| 17 \| "TS=(Malawi* OR Nyasaland )" \| 16742 \| \| 18 \| "TS=Nigeria*" \| 70492 \| \| 19 \| "TS=(peru OR peruv* )" \| 47350 \| \| 20 \| "TS=india*" \| 503100 \| \| 21 \| "#15 OR #16 OR #17 OR #18 OR #19 OR #20" \| 819351 \| \| 22 \| "#8 AND #14 AND #21" \| 4290 \| | |

5. WHO Global Index Medicus

| Interface: Global Index Medicus web interface https://www.globalindexmedicus.net/  Date of Search: 3 April 2023  Number of hits: 667 | Field labels   - Tw = Title, Abstract, Subject |
| --- | --- |
| (Oxygen OR oximet* OR "covid 19" OR corona*) AND (Policy OR Policies OR Regulaton* OR guideline* OR Legislat* OR law* OR reform* OR finance* OR industr* OR market* OR sector* Or planning OR program*) AND (Sweden* OR Swedish OR Bangladesh* OR Malawi* OR Nyasaland OR Nigeria* OR Peru OR Peruv* OR india*) | |
