# Supplementary material for: Challenges in the medical oxygen ecosystem of Peru: A political economy analysis
Source: PLOS Glob Public Health. 2025 Dec 19;5(12):e0005667. doi: 10.1371/journal.pgph.0005667 (PMC12716698; doi:10.1371/journal.pgph.0005667)
Supplement: S3 Appendix — (PPTX) [file pgph.0005667.s003.pptx]

## Slide 1
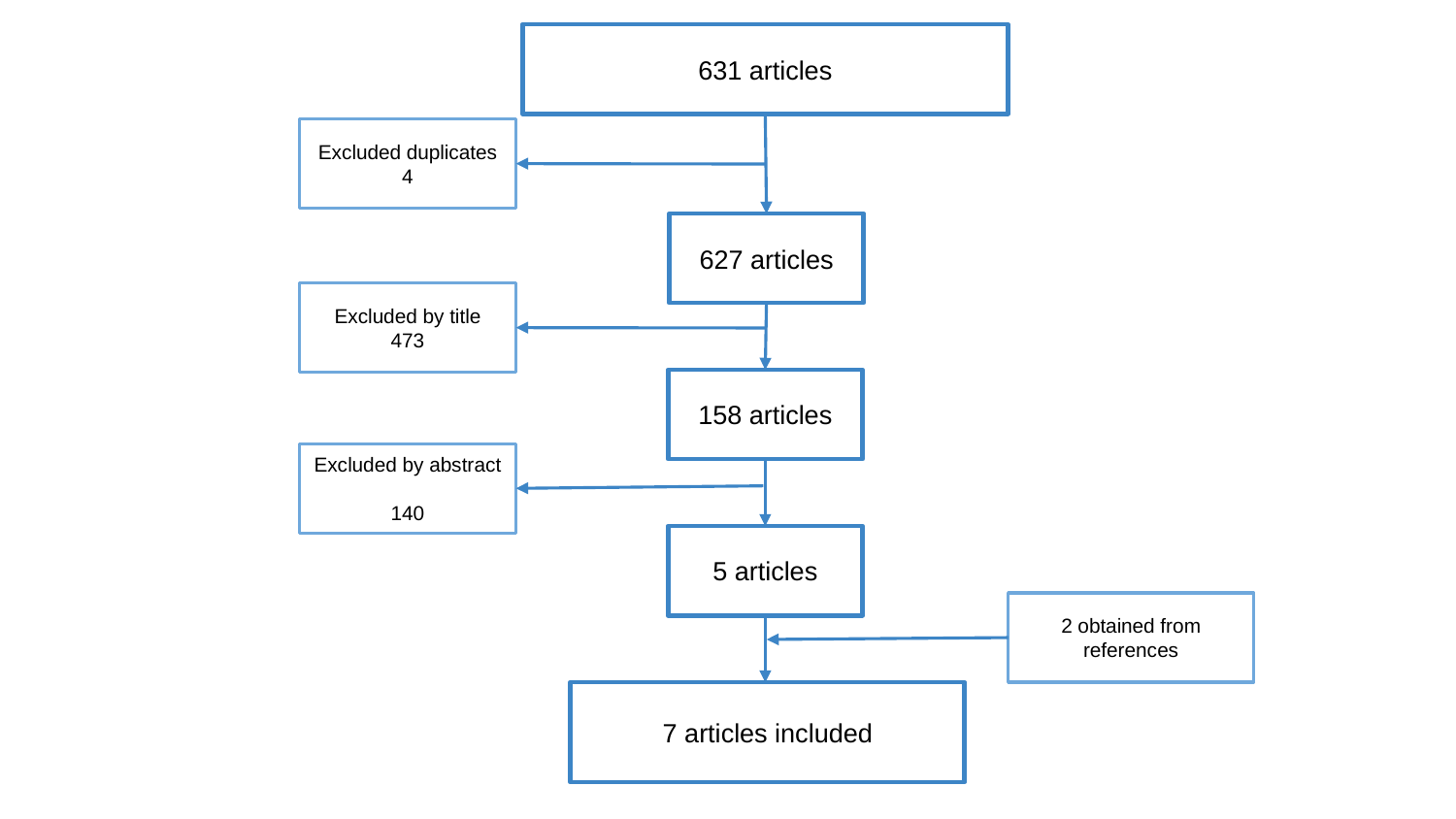

631 articles
Excluded duplicates
4
627 articles
Excluded by title
473
158 articles
Excluded by abstract
140
5 articles
2 obtained from references
7 articles included
