# Supplementary material for: Challenges in the medical oxygen ecosystem of Peru: A political economy analysis
Source: PLOS Glob Public Health. 2025 Dec 19;5(12):e0005667. doi: 10.1371/journal.pgph.0005667 (PMC12716698; doi:10.1371/journal.pgph.0005667)
Supplement: S5 Appendix — (PPTX) [file pgph.0005667.s005.pptx]

## Slide 1
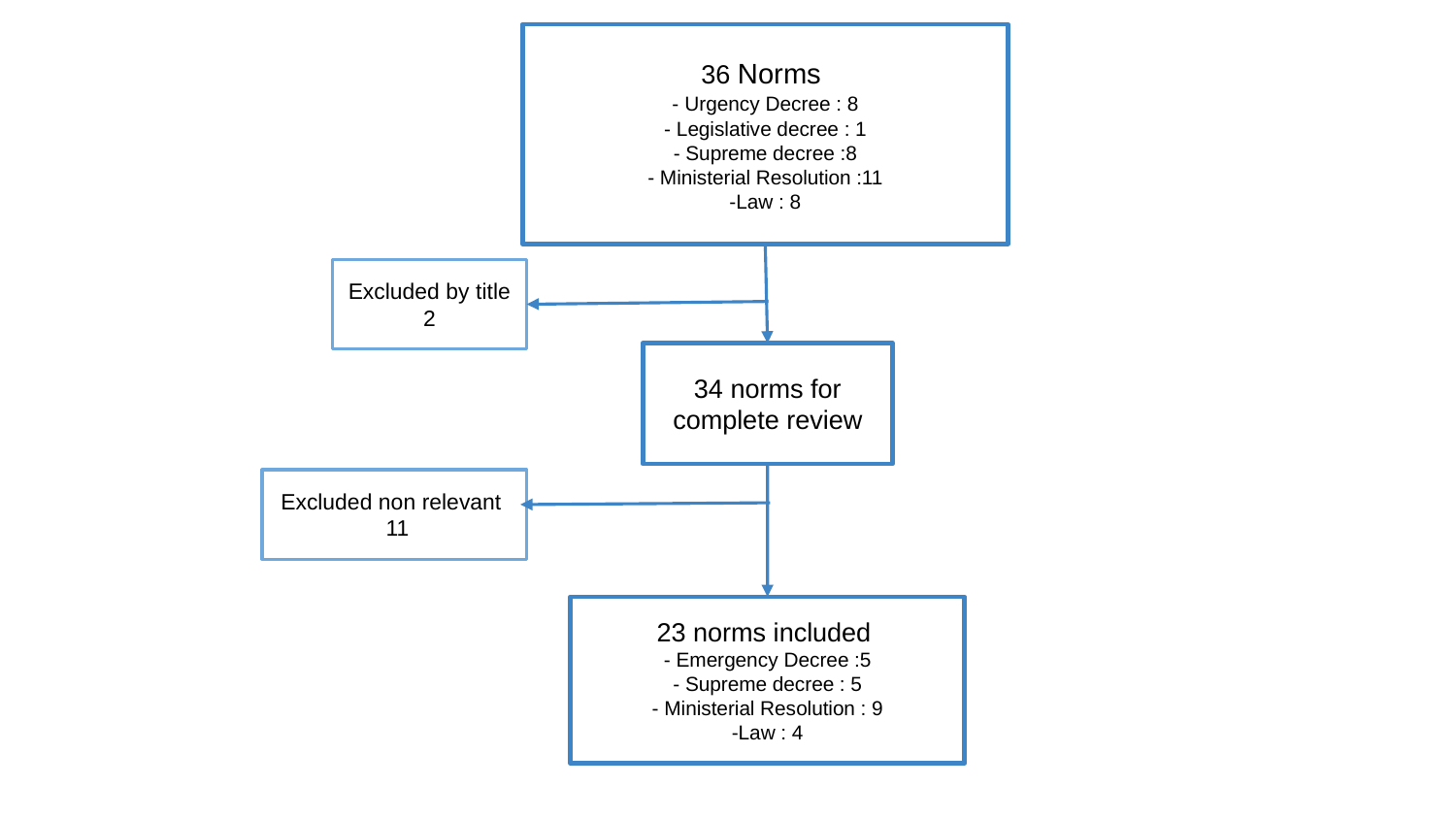

36 Norms
- Urgency Decree : 8
- Legislative decree : 1
- Supreme decree :8
- Ministerial Resolution :11
-Law : 8
Excluded by title
2
34 norms for complete review
Excluded non relevant
 11
23 norms included
- Emergency Decree :5
- Supreme decree : 5
- Ministerial Resolution : 9
-Law : 4
