# Supplementary material for: Challenges in the medical oxygen ecosystem of Peru: A political economy analysis
Source: PLOS Glob Public Health. 2025 Dec 19;5(12):e0005667. doi: 10.1371/journal.pgph.0005667 (PMC12716698; doi:10.1371/journal.pgph.0005667)
Supplement: S6 Appendix — (PDF) [file pgph.0005667.s006.pdf]

**List of interviewees for the Medical Oxygen study Final**

| Code | Sector | Notes | Gender |
|---|---|---|---|
| 001 | Government (National) | In Person/ No recording | F |
| 002 | Civil Society NGO | Through zoom/ recorded | M |
| 003 | International Agency | Through zoom/ recorded | M |
| 004 | Private Sector | In Person/recorded | M |
| 005 | Government (National) | In Person/recorded | M |
| 006 | Private Sector | Through zoom/ recorded | M |
| 007 | Government (National) | Through zoom/ recorded | F |
| 008 | Professional Society | Through zoom/ recorded | M |
| 009 | Civil Society (Catholic Church) | Through zoom/ recorded | M |
| 010 | Government (Regional) | Through zoom/ recorded | F |
| 011 | Government (National) | Through zoom/ recorded | M |
| 012 | Health Provider | Through zoom/ recorded | F |
| 013 | Health Provider | Through zoom/ recorded | M |

**Entrevista a informantes clave**

| | |
|---|---|
| Código de la entrevista: | 001 |
| Fecha: | 06/07/2023 Gobierno Nacional |
| Lugar de la entrevista: | Lima |
| Género del participante | Femenino |

**ENTREVISTA: (Transcription/Notes participant didnt allow for recording).**

**Inicio**

*2. Políticas de oxígeno*

**Primero es importante entender que para obtener Oxigeno en el Perú existen dos (o tres) canales: (1) el oxígeno criogénico : que se obtiene a través de una sola empresa (monopólica) la empresa LINDE . (2) Tener plantas generadoras de Oxígeno y (3) a través de equipos pequeños como concentradores de oxígeno, que solo ofrecen concentraciones bajas del oxígeno.**

**Hasta antes de la pandemia, lo único que teníamos en el país era a LINDE. Ahora ya hay concentradores y hay plantas generadoras. Es importante entender que estas plantas requieren tener mantenimiento y cumplir con ciertos requisitos para la calidad del oxígeno. Lo que hacen las plantas es tomar el aire y con un compresor de aire, filtran y lo enfrían, luego pasan por filtros de alta eficiencia, para reducir riesgo de contaminación con aceite, bacterias, virus, y luego por filtros de carbón activado para que tenga un olor neutro. Por unas válvulas se separa el oxigeno de otros gases del aire y van a un reservorio, y luego del filtrado y compresión se distribuye el oxigeno ya sea por redes o balones. Otro aspecto importante es que las plantas necesitan mantenimiento predictivo, preventivo y correctivo y darles continuidad. La limpieza y cambios de filtros por ejemplo permiten asegurar que el oxigeno producido se mantenga de calidad medicinal sino ya se convierte en oxigeno industrial**

**La Dirección General de Medicamentos, Insumos y Drogas (DIGEMID) con su función de asegurar la calidad y seguridad de medicamentos, insumos y drogas y el adecuado desempeño de los equipos médicos tiene un papel critico en relación con el oxígeno, y durante la pandemia fue quien se encargó de destrabar y permitir que se lograran tener plantas de oxígeno, pero sobre todo garantizar que las plantas generen oxigeno apto para el consumo humano, es decir con concentraciones iguales o mayores a 90%.**

**DIGEMID cuenta con los listados de plantas. Solo una empresa en el país cuenta con licencia (como si fuera un laboratorio para las medicinas, para el oxigeno se le da licencia de**

**laboratorio a la empresa que produce el medicamento oxigeno), y también con un registro sanitario de planta generadora de oxigeno medicinal.**

**Durante la pandemia solo se entregaron licencias por emergencia sanitaria, y lo que se debería hacer ahora es reevaluarlas y solo entregar licencias a aquellas que demuestren que mantienen su producción de oxigeno con 90% o mas de concentración. Tambien durante la pandemia se creó el sistema RENOXI, que era una base de datos para que cada EESS reporte las necesidades de oxigeno en el país. Nadie en el Ministerio de Salud se quería hacer responsable de esto, así que DIGEMID lo asumió, y se lanzo en setiembre del 2021. Sin embargo, a comienzos de este año se cayó la pagina web, ataque informático, y ya no hay la información.**

**Antes del COVID, se creía que el único generador de oxigeno era la planta de oxígeno criogénico, de modo que LINDE (la empresa), había monopolizado todo. Ellos tenían el certificado por la DIGEMID como "laboratorio" para producir y abastecía a todo el país, y era suficiente. Cada EESS y cada hospital compraba su oxígeno y tenia su presupuesto, nadie sabía cuanto era el total. El oxigeno esta registrado como un producto farmacéutico y la planta de LINDE funcionaba como un laboratorio. Pero durante la pandemia, llego un punto en el que el monopolio dijo que no podía mas y no se daba a baso y surgieron otras.**

**En RENOXI se ve las necesidades de oxigeno en el país, pero no se si la han seguido llenando o quien la usa. Recién a raíz de la pandemia se comenzaron a revisar las normas con respecto al oxígeno.**

**Después de la pandemia no se esta haciendo un control, hay que verificar si funcionan o no las plantas, priorizar por riesgo quienes están haciendo o no mantenimiento preventivo y ver que el O2 marque 93% +/- 3%**

**Todo se ha quedado en stand by , los controles, las visitas, los registros (que son de emergencia nomas), Esto es un riesgo inminente. No sabemos que oxigeno se esta usando en los hospitales. Yo tengo gran preocupación especialmente por las zonas mas alejadas porque no tienen capacidades de hacer mantenimiento, no tienen planes, no les llega el insumo y he escuchado que sus % de oxígeno de producción de las plantas esta por el 87%.**

*3. Financiamiento:*

**No hay un financiamiento definido a nivel central para el oxígeno. Cada EESS y hospital tiene su presupuesto y de allí saca. Durante la pandemia no se sabia bien que hacer La gran mayoría de las plantas ingresaron por colectas públicas, se creó RESPIRA PERU, (Caritas creo que esta relacionado). El gobierno contrato unas 40 plantas de oxígeno que las iba a hacer la UNI pero esta no pudo cumplir. Entonces todos se desesperaron y comenzaron las colectas publicas y donaciones. RESPIRA PERU, le daba plata a los CS que tenían que buscar su planta, a otros les donaban los privados, la iglesia. Y se supone que los establecimientos se encargarían del personal y el mantenimiento, que no se como funciono en la emergencia, pero ahora no se sabe o no hacen mantenimiento y no tienen personal y las plantas están abandonadas y los**

**que necesitan vuelven a comprar al monopolio. La mayoría de las plantas de oxigeno durante la pandemia fueron puestas a funcionar por donaciones (Caritas, club de leones etc). El estado tuvo muy poca inversión en esto.**

*4. Regulación, responsabilidad y supervisión*

**No se está viendo el problema del oxígeno a nivel central, ni como sistema ni como país. Todas las plantas tienen registros de autorización excepcionales. Había una lista completa en la pagina web de la DIGEMID, pero se borró con el problema de un virus informático.**

**Desde la DIGEMID se debería vigilar el oxígeno como se hace farmacovigilancia, pero ya no se le da la importancia porque terminó la pandemia. Lo único que se hace es que se da autorizaciones sanitarias desde el nivel central (dirección de dispositivos médicos y productos farmacéuticos). Y ahora ni se esta supervisando. Como se hará en regiones, no lo sé, pero aquí en Lima incluso no hay un encargado propiamente del suministro y calidad del oxígeno.**

**No existe en realidad un programa de oxígeno, solo normas para regulación y control.**

**Las normas fueron desarrolladas por la DIGEMID, y la Dirección General de Operaciones en Salud (DGOS) del MINSA a través de la Dirección General de Infraestructura, Equipamiento y Mantenimiento (DIGIEM). Pero tuvimos que trabajar juntos, la DIGIEM estaba causando cuellos de botella, ellos prohibieron compresores que usen aceite, y en el mundo no había compresoras sin aceite. DIGEMID tuvo que entrar para explicar que bastaba con usar filtros para aceite.**

**Hay regulaciones por parte de DIGEMID sobre si no cumple con especificaciones, lo que puede llevar al cierre temporal o definitivo de las plantas y multas pero no se esta haciendo nada, no se le da importancia.**

5. Compromiso político

**No hubo durante la pandemia compromiso político para solucionar el problema, DIGEMID tuvo que tomar el problema. Como la gente moría, se necesitaba dar soluciones y viabilidad, y ordenar y al menos ver que las plantas generaran oxigeno optimo para consumo humano.**

**Ahora nos encontramos con una situación en la que no se esta dando mantenimiento, hay carencia de personal (entrenado y no entrenado) para asumir en las regiones, hay problemas con los repuestos (todos son importados, en realidad las plantas venían en partes y aquí solo se ensamblaban). Nada se hizo de manera planificada, y nada se esta planificando ahora, no se planteó dónde se requieren o dónde no. Cayeron en donde consiguieron una planta.**

**Normas no faltan, lo que se necesita es una política nacional sobre el oxígeno medicinal, falta decisión política se necesita buena planificación, y adecuar la política a las necesidades nacionales que incluya un proceso racional de vigilancia y control .**

**Se debe trabajar planeando según necesidades, de acuerdo a ubicación, el riesgo es muy alto.**

*Estudio en profundidad del caso*

**Los actores clave en el tema de oxigeno durante la pandemia fueron, primero la DIGEMID, que se sino se ponía las pilas el MINSA no sabia como solucionar el problema de la falta d oxígeno, DIGEMID respondió a todas las solicitudes y le dio viabilidad a las plantas. No estoy segura como funcionó el resto del MINSA, pero la DGOS y la DIGIEM participaron en las normas y planificaron como unas 40-60 plantas, pero no sé cuántas se lograron. El sector privado y la sociedad civil (ONGs) contribuyeron mucho adquiriendo y donando plantas. Al final se tuvieron como 500 plantas de oxigeno en todo el país, el problema es que cada una es un monstruito diferente, muy difícil de dar mantenimiento y hacer seguimiento con tanto desorden.**

**La lección (que no se si hemos aprendido), con esta experiencia es que se requiere organizarnos para garantizar el abastecimiento de este medicamento vital, independientemente de que ahora tengamos la capacidad de abastecernos. Hemos caído nuevamente en los brazos del monopolio. Deberíamos recuperar y tratar de mantener las plantas que son estratégicas y que estén listas a funcionar. Lo malo es que ahora le siguen comprando a LINDE, que tiene nuevamente copado el sistema de oxígeno. Otra pandemia si se viene, no podremos responder sino nos preparamos. LINDE tiene un tope. Plantas estratégicas serian de seguro un ahorro al estado, nadie ha sacado cuentas. Se si invierte en mantenimiento y plantas operativas, y personal adecuado bien entrenado eso seguro que es un ahorro y buena inversión del estado.**

**&&&&&&&&&&&&&&&&&&&&**

## 002 Entrevista

Meeting started: 15/6/2023, 11:31:34

Participantes: E, I

**I: Investigador**

**E: Entrevistado**

I: Mucho mejor. Ahora, sí lo escucho mucho mejor. Gracias.

E: Bueno, como le había compartido en el consentimiento informado, este es un estudio que se está haciendo en varios países, en de la Universidad Cayetano Heredia y como allí lo decimos el objetivo del estudio es comprender el entorno político, financiero… la parte normativa, también sobre el tema del oxígeno medicinal y con la finalidad de, digamos, mirar qué es lo que se podría hacer mejor, en caso se tuviera alguna situación similar a la ocurrida durante la pandemia, no? como para tomar o mirar, si es que todo lo que se hizo está bien o no, o se puede mejorar algo. En fin, ese es el sentido o la finalidad de este estudio. Usted ha podido revisar el consentimiento informado.

E: Sí, sí. La revisé

I: ¿Tiene alguna pregunta alguna duda alguna inquietud al respecto?

E: No, ninguno.

I: Ok, ¿está usted de acuerdo entonces con que pueda grabar la entrevista?

E: Bueno. Está en no hay problema.

I: Ok, Entonces voy a voy a grabar en este momento.

E: No puedo, No me permite grabar aquí. Pues no me permite. Bueno, lo vamos… Voy a grabar en el cel (…) si es que no hay problema por allí dime un minuto, por favor, antes de comenzar yo le voy a enviar el consentimiento informado con la fecha con todas las referencias, de este día para que usted tenga el documento final con la firma, con el nombre (…)

**(COMIENZA AUDIO)**

**I: Entonces, vamos a ir comenzando. Y si usted no tiene preguntas al respecto, vamos a comenzar. Como decía, en el consentimiento informativo, me gustaría que usted se sienta muy libre de poder responder las preguntas; si hay alguna inquietud, duda que tuviera durante la conversación que vamos a tener, pues, me las dice nada más. Si en algún momento usted necesita parar, responder una llamada, que sé yo, me avisa para poner en pausa la grabación y podamos luego continuar grabando. Entonces… como para ir conociéndolo y también conocer el trabajo que usted tuvo, cuénteme un poquito cómo fue su experiencia en este tema del oxígeno medicinal durante el tiempo. ¿Qué labor tenía? ¿Cuál era su trabajo concreto en este tema?**

**E:** Bueno, para presentarme, mi nombre es **E.** Soy administrador de empresas de profesión y tengo la especialidad de agente de aduana, acreditado en la SUNAT. Yo trabajo para Cáritas del Perú, trabajo en el

área de importaciones de Cáritas. Y nosotros… la labor que desarrollamos es ingresar la ayuda humanitaria que viene consignada para la iglesia católica en el Perú. Yo soy el encargado de nacionalizar toda la carga que viene para… el exterior, para hacer los trámites, gestiones ante las autoridades, la SUNAT y entregar a la carga al consignatario, que en este caso son entidades de Iglesia la Conferencia Episcopal y todas las jurisdicciones eclesiásticas. Para el caso específico del tema del oxígeno medicinal, la Conferencia Episcopal junto con el empresariado constituyeron una institución llamada Respira Perú, una organización que iba a apoyar para traer oxígeno… apoyar en el tema de la pandemia. Y es ahí donde nosotros participamos realizando la gestión para traer, en un principio balones de oxígeno, posteriormente concentradores y plantas de oxígeno, y después los accesorios para todo el tema de oxigenación para …que ingresó a través de Respira Perú y todo consignado a la Conferencia Episcopal Peruana.

**I: Usted menciona con un grupo de empresarios. ¿A quiénes nos referimos con ellos?**

E: La Sociedad Nacional de Industrias y la Universidad San Ignacio de Loyola. O sea, ante la necesidad de implementar… plantas de oxígeno en el Perú y ante la necesidad o la urgencia que veíamos en los canales de televisión, donde faltaba oxígeno es donde el presidente de la Sociedad Nacional de Industrias y los señores de la Universidad San Ignacio de Loyola deciden implementar el tema de Respira Perú, pero faltaba la otra parte ¿no? dentro de ese trinomio que formaron, que fue la Conferencia Episcopal para darle legalidad para ingresar la ayuda humanitaria A Perú. Entonces, formaron el empresariado, la parte social la Universidad San Ignacio Loyola y la Conferencia Episcopal, que lideraron el tema del tema del oxígeno medicinal que se iba a apoyar al gobierno.

**I: Me hace mención a una cuestión de legalidad por parte… de Cáritas o del Episcopado. ¿A qué se refiere con eso?**

E: Si, lo que pasa que existe en el Perú una ley que regula el ingreso de donaciones al Perú, la ley 28905, que es la que regula el ingreso de donaciones del exterior hacia el territorio nacional. Y esa ley establece los requisitos legales que tiene que tener una institución beneficiaria de donaciones para exonerarse de impuestos y puede gozar de todos los beneficios que la ley lo señala. Entonces, los empresarios no tienen ese requisito, ¿no? ellos pueden importar, pagar tributos y distribuir alguna… mercadería. Pero el tema se complica cuando traen mercancía restringida, el tema de equipamiento médico. Tendrían que establecerse como un establecimiento de salud o una droguería para poder obtener autorización para importar ciertos equipos. Pero cuando nosotros utilizamos la importación, al amparo de la ley 28905, tenemos otro marco legal y algunos requisitos, procedimientos, se simplifican para instituciones que están debidamente acreditadas; en este caso, la Conferencia Episcopal que tiene el reconocimiento y la inafectación de tributo.

**I: ¿Y a partir de qué momento… cuándo es que empiezan ustedes a intervenir? ¿En qué fecha más o menos recordará usted?**

E: En la pandemia empezó en marzo, fines de… Bueno, ya en marzo teníamos— todavía no teníamos el tema de importación de equipamiento médico o plantas de oxígeno para el tema de oxígeno porque, en ese momento, no había esa necesidad, ¿no? no existía la necesidad de intervenir en el tema del oxígeno. Pero sí teníamos ayuda humanitaria regular que traíamos todo el año. En principio, empezó la pandemia, nos mandaron a la casa a todos, empezamos a implementar el procedimiento virtual para el despacho de donaciones. Y en los meses de junio, julio, donde ya se complica el tema de oxígeno, donde veíamos a la gente en las calles con sus balones, gente muriendo por el oxígeno, es donde los empresarios dicen "¿Y qué hacemos? ¿Qué podemos hacer para ayudar?" Entonces, en el mes de junio es donde empezamos trayendo balones de oxígeno. La Sociedad Nacional de Industrias, con el grupo de Respira Perú, adquieren

un contenedor de balones y lo donan a la comunidad. Y es el primer embarque que traemos de balones de oxígeno.

**I: ¿Eso en qué fecha, usted recuerda?**

E: El mes de junio,

**I: ¿En junio? Ok.**

E: Empieza—empezamos trayendo ese primer embarque de balones. Y después el tema del oxígeno se complica más en Arequipa, y se trae la primera planta de oxígeno para Arequip. Ahí tengo unos links que te voy a compartir donde el tema de las instituciones Respira Perú, dan toda esta información respecto a esa campaña, porque ellos convocan a la población para colaborar, ¿no? O sea, ellos ponían una parte, el empresariado, pero también convocan a la población a colaborar para poder hacer realidad esta primera planta de oxígeno en Arequipa, que fue la primera que empezó todo el procedimiento. Entonces, se trajo eso. Hay bastante información en la web respecto a esa primera planta. La primera planta que se sacó fue para Arequipa. Y de ahí empezaron ellos a replicar esta planta de oxígeno en otras ciudades del Perú, y se trajo mayores plantas, concentradores de oxígeno, unos respiradores que … un aditamento médico que se utilizó en Ecuador con éxito, que lo fabricaba una empresa, que era fácil utilizar.

**I: Un portátil.**

E: Entonces, eso también se empezó a importar que Estados Unidos, se trajo bastante.

**I: ¿Y ustedes tuvieron alguna…? O sea, entiendo que se ampararon en esta ley que les permite, digamos, importar a través del Episcopado, a través de Cáritas, ¿pero habrán tenido como alguna dificultad, o habrán digamos, apelado a algún tipo de otra normativa para tener la facilidad de importar o de recibir todos estos insumos, equipos?**

E: Sí, mira, justo buena pregunta, me he hecho recordar. La ley 28905 habla de un procedimiento especial cuando nos encontramos en una situación de emergencia y desastres naturales, donde el procedimiento se simplifica, ¿no? porque estamos en el entendido que es una situación de emergencia. Entonces, en el Perú, por un tema histórico, sufrimos terremotos, inundaciones, el Fenómeno del Niño y muchas otras emergencias, no sé. En los años que tengo de experiencia, en el año 2008, se publica la ley, ante tanta insistencia de las organizaciones de ayuda humanitaria que recibimos donaciones; y en una situación de emergencia, estar frente a requisitos burocráticos para ingresar ayuda humanitaria es un absurdo, ¿no? Entonces, a tanta insistencia, se saca la ley 28905 y se establece un procedimiento simplificado para temas de emergencia y desastres naturales. Pero el tema está en que el COVID, para las autoridades, tanto de la SUNAT como el Ministerio de Salud, no era una emergencia o desastre natural, o sea, la normativa señalaba qué era una emergencia y desastre naturales, así dice literal. Y para ello decía que en ninguna parte de la ley señalan emergencias sanitarias. O sea, el COVID ha sido el peor, la peor situación de desastre en el Perú, pero para las autoridades y las normas legales nunca fue amparada ni acogida dentro de este procedimiento simplificado.

Entonces, nosotros cursamos cartas al Presidente Vizcarra, al Congreso para que modifiquen la ley en ese instante, porque teníamos todo el tema de paralización de despachos en la aduana ante los Ministerios, pero nunca se hizo caso a nuestra solicitud; por el contrario, nos nombraron algunos edecanes para solucionar el problema inmediato, ¿no? para pagar el incendio: “¿Qué problema tienen?” “Tengo 2 o 3 contenedores en la aduana” “Bueno, vamos a hablar con ellos”, llamaban a la SUNAT, al Ministerio de Salud para que hagan el trámite de simplificación. ¿Pero qué pensábamos? Que la emergencia va a durar 15 días, 10 días, pero un mes, otro mes, otro mes, pero nunca modificaron la ley. En este momento si

hubiera un rebrote del COVID, como está sucediendo en Chile, en otros países, tampoco podríamos utilizar este beneficio que la ley que facilita el ingreso de donaciones establece porque el COVID no está contemplado -ni otra pandemia sanitaria está contemplada- dentro de la ley. Entonces, urge que las instituciones, tanto de ayuda humanitaria como académicas, instituciones que puedan apoyar para que el Gobierno, los congresistas, el legislativo pueda modificar la ley 28905 y pueda incluir las emergencias sanitarias dentro del ámbito de aplicación de facilidades, ¿no? porque es un absurdo que, en una situación de emergencia, sigamos todo el trámite burocrático que se sigue para recibir donaciones, como si estuviésemos en una situación normal.

Hemos tenido que exponer a nuestros empleados, a nuestros colaboradores para realizar verificaciones físicas de mercadería, recoger al personal de DIGEMIT de la región, de la dirección de equipamiento para trasladarse desde sus domicilios, en plena…

**I: Pandemia.**

E: … cuarentena, donde todo el mundo quería evitar contagiarse, teníamos que ir, rogar prácticamente a los inspectores para que salgan de su casa y llevarlos a la aduana a verificar los equipos médicos, los concentradores, para que esos equipos puedan ingresar y puedan servir a la población que estaba muriéndose. Entonces, es por la negativa de los mismos funcionarios del Ministerio de Salud y de la Aduana en reconocer a la emergencia sanitaria como una emergencia contemplada en la ley. Ellos simplemente nos convocaron a todas las organizaciones de ayuda humanitaria para indicarnos que iban a dar facilidades, pero no podían ir más allá de sus funciones por un tema de normatividad, ellos se ampararon indicando "No, no tenemos una norma legal que dé facilidades para el COVID". Entonces si hay un desastre, un terremoto, si hay facilidades; si hay una inundación, hay facilidades; pero una emergencia sanitaria, como ahora estamos viviendo el dengue –porque, por ejemplo, ahorita tengo una… mire, ahorita en Trujillo, no hay paracetamol, no hay en ninguna farmacia; pero, sin embargo, yo tengo una donación de Tylenol para apalear el tema de fiebre, en la aduana, esperando que el personal le DIGEMIT se digne a nombrar un inspector para hacer la inspección y luego pueda yo hacer el trámite para donar y retirar la mercancía, es un absurdo. Estamos una situación difícil.

**I: ¿A qué atribuye usted esta dificultad en las instituciones públicas? Porque me habla de DIGEMIT, me habla del MINSA. ¿Es la parte normativa únicamente o, no sé, tal vez más una cuestión de interés político, más de…? No sé, de otro tipo, tal vez.**

E: Sí, mire, para que usted entienda y se dé cuenta de lo que le digo, cuando empieza el proceso de emergencia, empiezan a importar plantas de oxígeno de forma comercial, ¿no? los que tenían… usualmente traen plantas de oxígeno para la industria. Esas plantas de oxígeno ingresaron al Perú sin ninguna dificultad porque pagaban impuestos y porque los empresarios se estaban llenando de dinero, ¿no? Pero, cuando la iglesia y Respira Perú empiezan a traer equipos para ayudar gratuitamente e implementar los hospitales o los centros de salud, empieza la DIGEMIT a poner problemas y decir "Uy, no, estamos dejando pasar plantas; pero, a partir de ahora que empieza Respira Perú, este producto se vuelve restringido". Arancelariamente, nosotros ingresamos a la información de SUNAT y las plantas de oxígeno no eran productos restringidos, pero como Respira Perú y la Conferencia empiezan a importar estos equipos para ayudar y distribuirlo gratuitamente la DIGEMIT cambió toda la normatividad. ¿Qué dijo? "No, ahora son restringidos. Y ahora yo tengo que controlar y vigilar estos equipos". Entonces, empezó un problema, porque solamente… digamos, esta compuerta de control era para nosotros y no para los importadores. Entonces, empezamos a quejarnos.

Tuvimos una reunión con la SUNAT y DIGEMIT para decirlo "Oye, ¿qué pasó? O sea, ¿por qué a mí me aplicas este un procedimiento diferente al que aplicas para un importador?" Entonces, la SUNAT y la

DIGEMIT se pusieron de acuerdo y dijeron “No, ahora va a ser para todos”. Entonces, mire, en una situación de emergencia, la SUNAT—en este caso, DIGEMIT y la SUNAT por añadidura, porque ellos lo que hacen es aplicar lo que dice DIGEMIT, ¿no? o el Ministerio Salud, “A partir de ahora, a estos productos se les va a exigir un requisito adicional de autorización sanitaria por parte del ministerio”, la SUNAT dijo “Mira, yo, antes, podía dejarte pasar los productos sin la autorización de DIGEMIT porque la información de ese producto arancelariamente no requiere o no exige ningún documento de control, pero como ahora DIGEMIT se dio cuenta de que lo están trayendo para distribuir gratuitamente, ahora les vamos a exigir el documento de control y no te puedo dejar salir los productos sin autorización de DIGEMIT”. Mire, la lógica, cuando más necesitamos la ayuda del Ministerio de Salud y de la SUNAT, empezaron a crear mayores barreras burocráticas para la importación.

Yo tuve una reunión con todos los funcionarios de SUNAT, los de DIGEMIT para explicarles cuál era la situación. Entonces, ¿cuál fue la salida de ellos? “Vamos a hacer más ágiles en el trámite de expedición de autorización”. O sea… porque ellos se pusieron… como es decir, la soga al cuello porque empezaron a exigir un requisito y no tenían funcionarios para hacer las verificaciones; emitían los documentos a destiempo. Entonces, nosotros teníamos las plantas de oxígeno en la aduana, y no las podíamos retirar porque no había una autorización de DIGEMIT, procedimiento que ellos se lo crearon, lo establecieron en plena pandemia. Entonces, estábamos en una situación donde requeríamos que las autoridades liberen de requisitos, den toda la facilidad para ingresar la ayuda, pero no se podía. Ellos empezaron a poner trabas y trabas y trabas, y era absurdo. No era este comprensible, ¿no? ante una situación… Es como que estamos en guerra con otro país, necesitamos balas, pero el Ministerio del Interior sale a decir “No, no podemos importar balas, que solo la puede traer un importador o, si la van a traer otros, hay que establecer requisitos adicionales”. Entonces, estaba muriéndose la gente, ¿no? que en esta situación tan difícil que pasó el país hayan procedido de esa forma las autoridades… Como le digo, la ley no me permitía utilizar ningún procedimiento de facilidad porque la emergencia sanitaria no está reconocida legalmente para acogerse a un beneficio, tanto de procedimiento como requisito, ¿no?

**I: De acuerdo. Y entiendo que eso pasó durante este momento de crisis, pero ¿sabe usted si luego se ha tratado de hacer alguna modificación? ¿Hay algún esfuerzo por ajustar esta normativa u otra normativa para—por la cual DIGEMIT, SUNAT, el MINSA, algún otro entre rector pueda intervenir en el tema o facilitarles a las instituciones—a la sociedad civil esta forma de importar, esta esta forma de también prestar ayuda a la población?**

E: No, ninguna. Ahorita estamos pasando el tema del dengue, le acabo de comentar el problema…

**I: Perdón. Le pierdo. Le perdí un poquito.**

E: Le comentaba que tenemos ahorita la emergencia del dengue y nos hemos dado cuenta que no hemos aprendido nada, ¿no? O sea, estamos en una situación terrible porque ahorita la gente… pasó el COVID, debimos aprender algo, ¿no? Nosotros mandamos varias sugerencias de cómo regularizar o corregir eso, pero ahorita no encontramos ninguna situación nueva, es lo mismo. Mire, seguro de que si aparece otra pandemia, igual se va… No entiendo, debe establecerse un procedimiento sancionador a este tipo de personas porque no pueden seguir ese tipo de funcionarios en una institución del Estado y proceder de esa forma en una situación de tanta emergencia en el Perú o crítica, ¿no? porque incluso nosotros en el terremoto que ocurrió en Arequipa, junto con la Cruz Roja, se estableció un trabajo de campo como… parecido a lo que estaban haciendo ustedes: convocaron a todas las instituciones, diferentes organizaciones que participaron de una u otra forma en el ingreso de ayuda humanitaria porque, después de la emergencia, se encontró que había contenedores con alimentos, con ropa podridos en los almacenes aduaneros. Y habiendo tanta necesidad, cómo era posible que estos productos estén almacenados o no se hayan podido retirar para la atención de la emergencia. Era increíble, ¿no? Entonces, ante tanta crítica,

se hizo muy parecido al que ustedes están haciendo, pero nunca tuvo rebote, nunca se llegó a concretar un procedimiento diferenciado. Y, es más… bueno, algunas cosas se recogieron eso en la ley 28905; pero, como le digo, no existían pandemias, ¿no? como nadie pensó que en el mundo iba a existir este tipo de emergencias sanitarias, y nos agarró desprevenidos a todos, ¿no? Pero no aprendimos nada, ¿se da cuenta? Ahorita, con el dengue, le puedo dar con bastante certeza de que no han modificado ni un artículo para para poder facilitar este procedimiento.

**I: Ok, usted me habla de que presentaron algunas recomendaciones, ¿como cuáles fueron estas recomendaciones para, digamos, mejorar este proceso, facilitar el trabajo de todos?**

E: La modificación a la ley 28 905 y la corrección del procedimiento de donaciones que la SUNAT maneja. Existen dos… la norma básica es la ley 28905 y la ley de… procedimental, que es la ley de procedimiento de despacho aduanero que tiene SUNAT, que recoge todo lo que dice la ley. Entonces, en esos 2 puntos está el destrabe de todo el procedimiento para ayudar en una situación de emergencia y de desastre natural por un tema de pandemia o salud, que no existe, ¿no? O sea, estamos en una situación donde ahorita hay escasez de agua, por ejemplo, estoy seguro que si empezamos a traer plantas para purificar agua, va a salir alguna autoridad a poner trabas. Es increíble que en el Perú pase eso, ¿no? pero ocurre y nos queda solamente acoger o corregir… Por experiencia, yo tengo ya 25 años ingresando ayuda humanitaria, entonces conozco el procedimiento, sé cómo sacarlo; pero, me da mucha pena que no seamos más proactivos en una situación tan difícil, ¿no? creo que… hay sanciones para nosotros cuando tratamos de pasar por alto algún tema de control o un tema administrativo, hay sanciones para nosotros. Entonces, muchas veces yo tengo… detrás mío hay un área que hace la distribución de las donaciones, y a veces no entienden, ¿no? cómo es posible que no pueda sacar de la aduana. Le digo "Tienes que esperar el tiempo, tengo que esperar que vaya el inspector de DIGEMIT tengo que hacer pagos en aduana, tengo que hacer pagos en los terminales de almacenamiento o..." "¿Pero cómo? estamos en una situación…" Mire, cuando privatizaron el puerto y el aeropuerto, se olvidaron de que había ayuda humanitaria en el Perú que ingresa por estos establecimientos, nunca negociaron la inafectación de pagos de servicios para la ayuda humanitaria. Por cada contenedor que llega al Perú, nosotros tenemos que incurrir en gastos que ascienden más o menos a 3000 dólares por contenedor…

**I: ¿Cuánto, perdón?**

E: 3000 dólares, más o menos…

**I: Ah, ya.**

E: … por contenedor. Entonces, el aeropuerto antes estaba listado por CORPAC, ¿no? Teníamos una… un documento de exoneración que expedía la Agencia peruana de cooperación internacional, y no pagamos ningún gasto administrativo. En el terminal portuario estaba ENAPU, y también la Agencia peruana emitía un documento, una constancia de acogimiento que se llamaba, nos acercamos al puerto y con ese documento exonerábamos el pago de servicios del uso del puerto, la descarga, la distribución. Pero, ahora, esto está administrado por DP World y APM. Entonces, para ellos no existen las donaciones, no… somos un cliente más para ellos y todos los servicios se tienen que pagar. Lo mismo sucede con.

**I: ¿Por quiénes me dijo? No le escuché bien. ¿Me podría repetir por quiénes?**

E: El puerto está administrado por DP World y APM Terminals, que son los 2 administradores del puerto del Callao.

**I: De acuerdo.**

E: Entonces, para ellos no existen donaciones, todos son servicios, ¿no?...

**I: ¿Y durante la pandemia…**

E: … todos tienen que pagar.

**I: … para la importación, también, de los equipos, ustedes tuvieron que pagar?**

E: Se pagaron todos los servicios, no se exoneró ningún pago, todo se pagó. Y, bueno, la parte de la colaboración de la gente ha ido para pagar servicios también, ¿no? porque… no, exonera ningún pago, todo hay que pagar, ¿no? Nunca el Estado… nunca hace nada, ¿no? Por eso, nosotros hacemos el trabajo adicional, tenemos que buscar recursos para cubrir esos gastos operativos que no que no se exonera, se paga.

**I: Hasta ahora, usted me ha comentado un… más o menos cómo… se trabajó a nivel de los entes rectores, pero ¿cómo, por ejemplo, ha ocurrido en las regiones? ¿Ustedes tuvieron dificultades, alguna barrera? ¿Cómo pasó en la región? Porque no sé si había siempre la apertura, la buena recepción para tener los equipos, para que los equipos lleguen a las zonas donde debían… se requerían más.**

E: Sí, bueno, los puntos de ingreso de la ayuda humanitaria, en el caso mío, han sido la aduana aérea y la aduana marítima del Callao. Hemos tenido ingreso de planta de oxígeno del Ecuador porque respira Perú… como en el Perú no producían las plantas o había escasez, adquirieron plantas de oxígeno en el Ecuador y las ingresamos por la aduana de Tumbes. Sí, ingresamos por ahí unas plantas que las trajeron para diferentes puntos. Pero, en el tema… era similar, igual que manejamos acá en la Aduana de Perú, ¿no?— de Callao, que diga. El tema de… lo que sí he podido, en algunos casos, intervenir es cuando se va a instalar la planta de oxígeno, ¿no? en un hospital, en un centro de salud. Estaba el tema de la de la lista, ¿no? porque seguro usted habrá visto todos los requisitos que tiene que tener un establecimiento de salud para implementar la planta, para instalarla, ¿no? Sobre todo el tema de requisitos, algunos les exigían… Por ejemplo, en el caso de Arequipa, Respira Perú compró un… alimentador eléctrico, pero no era de las dimensiones que establecía el procedimiento y, entonces, había que cambiar ese equipo; y por eso no le daban la autorización, porque Respira Perú hizo el lanzamiento, el obispo salió en los canales de televisión indicando que estaban entregando la planta de oxígeno y todo, y la gente dijo “Ah, por fin llegó el oxígeno, ya tenemos oxígeno en Arequipa, ya no va a haber problemas”. Pero no podía empezar a operar por un tema… digamos, que era jalado de los cabellos. Era para… volverse loco, ¿no? por un tema que no era la dimensión del alimentador oxígeno, era otro, que en otros lugares le faltó el piso, ¿no? está todo de concreto, hay una parte que no estaba… Entonces, era increíble que en una situación tan difícil nos pongamos así a rajatabla, a exigir todos los requisitos que recién se estaban creando o ellos, ¿no…? Pero fue difícil, ¿no? yo… Bueno, esa parte sí los otros participantes del estudio le van a comentar, pero yo en algunas reuniones de Respira participé y pude escuchar esto. Y me indignaba bastante escuchar este tipo de cosas.

**I: Y… pero… eso es a nivel de los hospitales. Pero ¿qué tal de las autoridades, de los gobiernos regionales? ¿Ustedes tuvieron algún… una comunicación fluida, apoyo, colaboración? Porque, hasta donde recuerdo, por ejemplo, en Arequipa, hubo algunas dificultades con el gobernador regional. No sé si lo mismo habrá ocurrido en otras regiones. ¿Tiene usted información al respecto?**

E: No, no en esa parte, no he participado. Como le comentaba mi trabajo, más estaba sentado a la importación y a conseguir la autorización de ingreso al Perú, ¿no?

**I: ¿Y tiene usted información, por ejemplo, del tema del financiamiento? Sé que ha sido con apoyo, con colaboración de empresas… de la empresa privada, de la población, pero ¿qué tan fácil, difícil habrá sido lograr los fondos suficientes como para comprar todos estos equipos? ¿Cuáles habrán sido, por**

**ejemplo, los retos que ustedes han tenido como como Cáritas, como institución o como Respira Perú para conseguir los fondos?**

E: Sí, ¿no…? Bueno en el tema de Cáritas, por ejemplo, nos mandaron todos a la casa por seguridad, se empezaron a contagiar acá el personal, todos en su domicilio. Casi Cáritas no participó dentro del tema de la campaña de emergencia de salud, pero sí la Conferencia Episcopal asumió ese reto por el tema de la convocatoria que hizo la Sociedad Nacional de Industrias y la universidad San Ignacio Loyola, ¿no? Son ellos los que han tomado la batuta. Pero, quien está… bueno, no fue tanto difícil porque todos estábamos… el empresariado quería colaborar, de cualquier forma ayudamos, ¿no? Pero, por ejemplo, no había… hay un tema de desconfianza de entregar el dinero recaudado a las autoridades, ¿no? Por ejemplo, Respira Perú empezó a recaudar fondos, pero ellos no quisieron entregar eso al Estado, sino a otra institución que sí dé visos de ser eficiente y eficaz en el ingreso de esta ayuda, ¿no? porque el Estado, con tanto dinero que tenía, no concretaba nada. Se da cuenta porque ellos no trajeron las plantas de oxígeno cuando veían que iba a existir esa deficiencia en el país, esperaron que la gente esté en la calle, con sus balones, durmiendo, contagiándose para recién ir implementando sus hospitales con las plantas de oxígeno. Porque todo lo que se ha traído, todas las plantas de oxígeno, todo el equipamiento ha sido entregado al Ministerio de Salud para subsanar la deficiencia que ellos tienen, ¿no? Y usted dirá "Pero si me estás trayendo cosas para ayudarme porque te das cuenta que yo no puedo hacerlas, ¿por qué me haces tan difícil el trabajo?", ¿no? Es un absurdo porque, si el Ministerio de Salud veía que no puede hacer las cosas bien y viene otra institución a ayudarle, ¿y por qué me pones trabas? ¿No? Vamos a apoyar, las plantas de oxígeno eran para el hospital Loayza, el hospital regional y diferentes instituciones, y empiezas a poner tú trabas y problemas. Era algo que no se podía entender, ¿no?

**I: ¿Usted tiene la cifra de cuántas plantas de oxígeno, cuántos equipos se lograron importar o efectivizar en la donación que se hizo a través de Cáritas?**

E: Sí, nosotros tenemos el dato tanto de las… de cuánto se ingresó de… porque todo ha ingresado de forma regular. Entonces, esa información… no le puedo dar, salvo que el Presidente de la Conferencia me autorice, pero también es pública porque ustedes pueden solicitar a la SUNAT la información del tema del ingreso de plantas de oxígeno. Pueden solicitarlo a través del… en el de la página web de SUNAT, donde van a poder recaudar esta información porque es información pública.

**I: Ok, si hablamos de un aproximado, no de cifras exactas, ¿de cuánto estaríamos hablando así…? ¿10, 20, 30?**

E: Son unas 10… 10 plantas, más o menos, 10 a 12 plantas.

**I: Plantas de oxígeno, pero también hemos tenido equipos, ¿no es cierto?**

E: Concentradores… por decir, para oxígeno, los otros respiradores también que se trajeron de Estados Unidos, que se mandaron a fabricar. Sí ha habido, pero ha sido consignado a la Conferencia Episcopal. Como le digo, esta información es pública, la pueden solicitar a la SUNAT, les van a dar la información.

**I: De acuerdo. Usted ya me ha comentado, por ejemplo, acerca de… estos… estas dificultades que se tenían a nivel de la infraestructura en los establecimientos de salud, como para la instalación de las plantas. Pero ¿era ese el único problema? ¿No sé si había personal suficiente, especialistas en el manejo de las plantas? O sea, ¿había alguna otra… carencia de recursos en este sistema que se trataba de establecer para dotar de plantas de oxígeno a los establecimientos de salud?**

E: Sí, porque la parte de la implementación, el equipamiento estaba a cargo de Respira Perú, pero la operatividad de esa planta estaba a cargo del Ministerio de Salud. Entonces, era el ministerio el que debía

establecer el lugar donde se iba a colocar la planta, y son ellos los que administran la planta y contratan el personal y el presupuesto para la operatividad, mantenimiento y personal, ¿no? Entonces, el… es ahí donde se presenta el problema porque muchas veces nosotros hemos traído equipos médicos, lo hemos entregado al ministerio… Pero, cuando alguna vez nos ha tocado supervisar esto con el ministerio de salud, la contraloría o la policía fiscal, que son los que controlan los productos, ¿no? Pero…

**I: Perdón. Le perdí. Le perdí el audio.**

E: En alguna ocasión, nosotros hemos ido a supervisar los productos que entregamos en calidad de consignación porque, lógicamente, nosotros somos una institución de ayuda humanitaria, pero no tenemos el personal ni la autorización para operar esos equipos, ¿no? y lo entregamos a los establecimientos de salud. Y, como le comentaba, todas las donaciones que vienen o ingresan por el canal formal son fiscalizadas por la SUNAT, la contraloría, la Agencia Peruana de Cooperación y la policía fiscal. Entonces, cuando en alguna ocasión ha logrado… hemos tenido que ir a hacer una supervisión y ver qué pasó con ciertos equipos, hemos encontrado que los equipos están inoperativos o están guardados en un almacén o los utilizan para guardar papeles. Por ejemplo, una vez trajimos una incubadora y lo encontramos de calentador de papel para las fotocopias porque decían que no tenían personal para operarlo y que lo utilizaban para calentar el papel para utilizar la fotocopiadora. Entonces, bueno, ya escapa a nuestra responsabilidad también, sino de ellos, ¿no? Pero sí ocurre que el… como le digo, la parte de equipamiento era nuestra responsabilidad: traer el equipo, desaduanarlo, entregarlo. Ahora, la parte de operativizar eso está a cargo del Ministerio, ¿no? tiene que tener, como usted mencionó, personal, la infraestructura, las autorizaciones; y eso es lo que este ha dificultado bastante, ¿no?

En Huánuco… Huánuco hizo una campaña del arzobispado de Huánuco. Hizo una campaña con toda la población para llevar oxígeno a Huánuco. Ellos utilizaron, hicieron una rifa con la población de Huánuco, juntaron recursos, “Vamos a traer una planta de oxígeno para Huánuco”. Nunca se pudo traer porque la dirección regional de Huánuco empezó a poner trabas para traer esa planta para su hospital, decía que no, que no es la planta que nosotros queremos, mejor nos dan el dinero a nosotros y nosotros lo administramos. Y el grupo que se conformó no quería soltar el dinero, y así pasó y la gente seguía muriendo y seguíamos discutiendo. ¿Y quién va a traer el equipo? ¿Quién lo va a administrar? En Huánuco pasó ese problema, y la Dirección de Salud de Huánuco debe tener información sobre esa planta que se gestionó con el… se trabó, ¿no? nunca se llegó a concretar esa ayuda para Huánuco.

**I: ¿Y, por ejemplo, qué ocurrió con los fondos que se recaudaron? Porque ya me dice usted: se recaudaron los fondos, tenían el recurso para la compra, pero no se viabilizó por la negativa del Gobierno—de la Dirección Regional.**

E: Sí, la verdad es que no sé cuál ha sido el destino final porque yo no interviene en la importación, nunca llegué a importar estos equipos. Estaba entrampado en esas 2 organizaciones, porque en otras regiones, por ejemplo, en Cusco, en Cajamarca se establecieron sus campañas de cada lugar, ¿no? porque todos veían en Arequipa han llevado una planta, ¿cómo han logrado? “No, la gente ha colaborado y la autoridad, nosotros también queremos nuestra planta”. En Huánuco, en Chimbote, en diferentes sitios del Perú se establecieron las campañas locales para juntar recursos y traer una planta de oxígeno. Seguro… ¿en el estudio ha visto eso, ha escuchado?

**I: Estamos empezando. Así es.**

E: Ya, han hecho en Cusco, en Cajamarca, en Chimbote… después en la selva también hicieron este tipo de campañas locales porque la gente veía que traían plantas de oxígeno y querían traerlas. Pero el tema estaba justo en lo que usted señala en la pregunta, ¿no? “Quién va a manejar la planta y quién le va a dar

mantenimiento y quién va a contratar el personaje". Porque el tema de traerlo lo solucionaban con nosotros, bueno, a través de Cáritas lo vamos a traer, el equipo viene, lo desaduanamos... El tema estaba en ahora quién lo va a administrar, ¿no? Incluso en algunos lugares, bajo este procedimiento de donaciones, estaba el tema de… nosotros traemos una donación para un fin establecido, ¿no? Cuando hacemos la importación, esta planta de oxígeno está destinada a la población, por ejemplo, de Chota en Cajamarca; un ejemplo, ¿no? Yo tengo Cajamarca. Entonces, ¿qué sucede? Como la emergencia no estaba en Chota, sino la emergencia estaba en Cajamarca, ¿qué hacía la Dirección regional de salud de Cajamarca? "No, esta planta está mal ubicada en Chota, hay que llevarla a Cajamarca". Entonces, la gente empezó a decir "No, pero nosotros hemos traído para Chota, ¿no? No hemos traído para Cajamarca, que Cajamarca compre su planta, ¿no? o haga a su campaña". Entonces, empezó un tema de entre nosotros a… pelearnos entre peruanos, ¿no? porque usted se da cuenta… si Cajamarca tiene presupuesto y no ha comprado su planta, ¿por qué ahora quieren agarrarnos nuestra planta de Chota y llevarla a la otra provincia? Y empezó una disputa entre nosotros, porque el gobierno no hizo bien su trabajo porque no le explicó a la gente o había una cultura de ayuda entre nosotros mismos, ¿no? En una situación de emergencia es eso, ¿no? O sea, hay que sobrevivir como sea, el más fuerte va a vivir, ¿no? el que mejor se organiza… Y cuando empieza este tipo de movimientos dentro del gobierno de querer llevarse un producto a otro lugar, empieza el problema, ¿no? la gente de forma extraña y a veces, ¿no? cuando estamos en una situación de emergencia.

**I: Así es. Entiendo que su labor no es supervisar, no es acompañar más allá de la implementación, o sea, de las plantas. Pero ¿sabe usted si las plantas de oxígeno o el equipamiento que ustedes entregaron están en funcionamiento en este momento, están siendo usados o no?**

E: No tengo información de eso, pero sí le recomendé, por ejemplo, en la… a Respira Perú y a la Conferencia que establezcan un área de supervisión y control post COVID, ¿no? ¿Esto por qué? Por experiencia. el gobierno, sobre todo el congreso, cada vez que ocurre una situación de emergencia o desastre, después de 4 o 5 años, hace siempre una supervisión o control. Entonces, las instituciones que hemos participado dentro de la ayuda humanitaria tenemos que tener esa información para pasar esos controles, que sí nos corresponde porque la ley establece ese procedimiento de control por 4 años. Entonces, el Estado tiene una… tiene un rango de 4 años para hacer la supervisión o control. Entonces, esos suele venir después de 4 años, 3 años, ¿no? Si sea este por parte mía, yo he hecho la recomendación a ellos porque nosotros sólo participamos en la importación, no hacemos seguimiento, control, monitoreo, eso, ¿no? eso no está dentro de mi competencia. Solo yo veo el tema de importar, conseguir la documentación, hacer los pagos y retirar, en el menor tiempo posible, los equipos. Es lo que yo estoy a cargo.

**I: ¿Y sabe usted si su recomendación está siendo viabilizada, efectiva?**

E: Bueno, hace poco me han pedido alguna documentación, como la que usted me solicitó- el detalle, algunos documentos-. que sí veo que lo están haciendo porque siempre, en una situación de emergencia, hay instituciones que aprovechan el pánico, ¿no? Nosotros tenemos una institución acreditada que recibe donaciones; pero, en situaciones de emergencia, como por ejemplo el terremoto en Ica, se empezaron a crear organizaciones, municipios, todos quieren traer ayuda. No todos los que participan cumplen los requisitos o todos tienen el mismo fin, ¿no? algunos tienen otro fin de aprovechamiento. Y, cuando ocurre la emergencia, suele ocurrir denuncias sobre mal manejo de la ayuda; y es ahí donde el Congreso empieza a notificar a todas las instituciones acciones de control y supervisión, ¿no? Entonces, ahí es donde sale todo. El que le ha hecho bien el trabajo tiene toda la documentación, tiene las actas, todo; los que no han participado bien, suelen desaparecer, ¿no? Cuando la SUNAT va a supervisar, ya no existe, ya desaparecieron, ya se constituyeron, pero solo para la emergencia. Entonces, cuando pasa la emergencia,

ya no hay nadie a quien notificar, no hay información. Entonces, eso suele ocurrir siempre. Mire, yo tengo 23 años acá, en este trabajo, y en cada emergencia suele ocurrir eso. En cambio, la Iglesia católica está siempre… tenemos un precedente, un antecedente, en la SUNAT tienen todos los requisitos, las acciones de control siempre han sido levantadas satisfactoriamente. Nunca hemos tenido inconvenientes porque ya tenemos personal capacitado y preparado para este tipo de acciones.

**I: Ok. Si, por ejemplo… usted me ha hablado, por un lado, del Congreso, también de los responsables, digamos, de las de los entes rectores, ya sea de MINSA, la DIGEMIT, de SUNAT, y me mencionó también al Presidente. Pero en estos… entre estos actores, ¿quiénes son o quiénes fueron los responsables de encaminar o fueron los actores claves como para… orientar los esfuerzos para que el tema de las plantas del oxígeno se concreten, se viabilicen, lleguen a las zonas donde eran necesarios?**

E: Mire, el Ministerio de Salud es el ente rector máximo, ¿no? el que… en una situación de salud, ¿no? porque cuando tenemos otro tema, por ejemplo, de desastres naturales, interviene el Ministerio de Transporte, ¿no? Pero en un tema de salud, es el Ministerio de Salud el ente rector máximo. Y es ahí donde se requiere que la persona que está a cargo esté preparada o tenga conocimiento o esté apoyada con gente capaz, ¿no? De lo contrario, sucede lo que ha pasado en el Perú, ¿no? donde vemos que los funcionarios eran los primeros en vacunarse, en beneficiarse y son ellos los que estaban conduciendo… el mejor, digamos, mecanismo o procedimiento para que la ayuda llegue. Si ponemos gente de ese tipo, entonces nunca va a funcionar, siempre vamos a estar en este procedimiento, ¿no? porque… Quisiera comentarle que todo fue exitoso, sacamos todo fácil, pero no fue así. Fue todo complicado, todo cuesta arriba, trabajando contra todos porque utilizamos un procedimiento como si estuviésemos en una situación normal, asumiendo costos, como si estuviéramos en una situación normal. Nunca existió la pandemia para la SUNAT ni el Ministerio de Salud. O sea, yo no lo estoy hablando del Ministerio de Producción u otro ministerio, el mismo Ministerio de Salud, donde ellos no querían modificar el procedimiento y que se dé las facilidades, "Hay que modificar este procedimiento, esta ley que no habla de la emergencia sanitaria, pero ya existe, es una realidad". No es un tema de 15 días, 10 días. En su momento, ellos no tenían personal para salir a supervisar, su personal se empieza a contagiar, empiezan a estar restringidos, no tenían recursos porque no existía un presupuesto para la atención de emergencia, ¿no? Entonces todo eso… ¿y quién está a cargo de todo eso? el Ministerio de Salud. Es el… nos deja una situación como esta, surge un tema de análisis como el que ustedes están haciendo porque sí es necesario recalcar y observar todo lo que ha funcionado mal, ¿no? Y, en verdad, en la pandemia todo ha funcionado mal, ¿no? todo, todo, porque es inexplicable… Yo me indigné bastante cuando, como le digo, las plantas entraban fácilmente para los importadores, pero era complicado para nosotros que traíamos para ayudarlos. Entonces, era increíble. Si usted esto lo pone al público y le explica, seguro que se van a indignar tanto como yo porque era increíble que ellos saquen rápido las cosas para venderlo y beneficiar a alguien y nosotros que traemos para gratuitamente ayudar a la población nos pongan tantas trabas. Era increíble, ¿no?

**I: Si usted pudiera identificar, digamos… entiendo que hubo cuellos de botella de diversos… de diversas intensidades y tendría que decirlo, ¿no? una, la parte normativa, los equipos técnicos o los funcionarios que tenían esa dificultad para dar pase al tema de las plantas del oxígeno, a la parte también de la infraestructura. Pero si usted pudiera identificar, en todos estos actores, ¿qué ha sido lo más difícil? ¿Cuál ha sido el mayor de los retos que tuvieron que afrontar ustedes? ¿Cuál diría que es?**

E: Mire, el convencer a la gente que nosotros… a los funcionarios, ¿no? que están involucrados, convencerlos de que lo que estaban viendo en pantallas o escuchaban no era una mentira, no era una película, no era algo que no existiera, era una realidad. Entonces, cambiar la mentalidad del funcionario público es bien difícil en el Perú, o sea tiene procedimientos a rajatabla y no quiere saltarse una situación

donde tienen que aplicar el criterio. El procedimiento está establecido de una forma, pero querer modificarlo ante una situación como esta, el funcionario público dice “No tengo un respaldo, a mí me van a auditar y me van a decir por qué dejes salir esta ayuda; y me van a sancionar, ¿no? Y nadie me va a reconocer o decir ´Tú hiciste bien porque no le dijiste tal requisito ante una situación’”, y no existe. Ellos simplemente dicen “No tengo un procedimiento que me respalde para darles la ayuda que necesitamos. Sé que esta planta de oxígeno va a salvar la vida de mi madre, pero no lo puedo dejar salir porque el procedimiento me dice tal cosa”. Entonces, no había ningún tipo de criterio, ¿no? Por ejemplo, ahorita, nosotros peleábamos porque el procedimiento de donaciones establece que debemos esperar 7 días para poder retirar una mercadería… Como en este caso, por ejemplo, si ahorita llega un concentrador de oxígeno, yo tengo que presentar un expediente al Ministerio de Salud y esperar 7 días -como mínimo- para poder utilizar los procedimientos que me permiten poder retirar este equipo de la aduana. Entonces, tengo que esperar 7 días hábiles, ¿no? bueno, en una situación normal, como estamos viviendo ahora, puedo esperar, ¿no? Pero ¿qué sucede si yo traigo, como ahora le comentaba al inicio, traigo Tylenol para Trujillo, donde no hay Paracetamol en ninguna farmacia y la gente se está muriendo por la fiebre? Entonces, ¿qué me dice la aduana? “Espera los 7 días para que puedas retirarlo utilizando el procedimiento normal”. “Pero estamos en una situación de emergencia, señores, no puedo ayudar”. Y llamo a los funcionarios de DIGEMIT, y no tenemos funcionarios, tienes que esperar que te nombre… Entonces, es indignante, ¿no? porque, como al principio dije, no hemos aprendido nada, hemos pasado la peor emergencia o peor desastre… ningún terremoto, ninguna inundación ni un Fenómeno del niño ha matado a tanta gente en el Perú, y no nos hemos dado cuenta de que hay que corregir las cosas, los procedimientos tienen que ser más ágiles, expeditivos; si existe algo que no está normado, tenemos que corregirlo inmediatamente, no ser tan burocráticos, ¿no? Como yo le comenté, mandamos cartas al Presidente a avistar, al Presidente del Congreso y nunca tuvimos respuesta, nadie creyó que esto iba a ser tan devastador. Y a veces, cuando veo por televisión que los están acusando, yo dijo “Está bien, están presos por ser tan inhumanos, ¿no? O sea, no quisieron nada, no hicieron nada”. Nosotros le dijimos, estuvimos ahí tocándoles la puerta y llamándolos, explicando lo que estaba sucediendo, y no quisieron oírlo, nunca quisieron escucharnos.

**I: Entiendo su sensación de disconfort, de impotencia por lo que ocurrió. La pregunta es: ¿no se ha aprendido absolutamente nada? ¿O cree usted que ha habido algún cambio? ¿Hay alguna apertura? Si, digamos, ocurriese, tuviésemos un tema nuevamente de una pandemia, si tuviéramos la necesidad, por ejemplo, de tener nuevamente oxígeno, ¿estamos preparados? ¿No? ¿Podríamos afrontar o no?**

E: Mira, hace poco tuvimos una reunión con el PMA, que juntaba varias…

**I: ¿El PMA es qué?**

E: … Y… Programa Mundial de Alimentos, ellos ayudan en el Perú al traslado de la ayuda humanitaria, apoyan económicamente financiando fletes de traslado de mercadería. Hicieron una reunión que tuvimos. “¿Qué pasa si en Lima hay un terremoto ahorita? ¿Qué ocurriría? ¿No?” Entonces, me convocaron a una reunión donde estaba el Ministerio de Salud, Ministerio de Defensa, organizaciones que participó... Entonces, les decía un ejemplo, ¿no? “¿Qué pasaría?” Y no se ocurrió que el aeropuerto se paralizó por un accidente de los bomberos, que se estrelló una ambulancia con un avión. El aeropuerto inmediato que tiene que activarse es el de Pisco, inmediatamente. Pero ¿qué sucedía? No… O sea, yo les di un ejemplo, pero la realidad o el momento me dio la razón, porque inmediatamente quisieron derivar los vuelos del aeropuerto a Pisco, pero Pisco no estaba preparado, no tenía personal, el personal que estaba a cargo no estaba presente, la pista no estaba preparada… Entonces, se habla hace mucho tiempo que el Perú, Lima está esperando un terremoto de gran envergadura, hace años venimos trabajando eso, que hay que prepararnos, los simulacros y todo. Pero cuando sucede, no estamos preparados y nos damos

cuenta que… yo estoy seguro, como ocurrió el 2007, que llegaron los perros de rescate para buscar a la gente y no los dejaron entrar, no los dejaron entrar porque no tenían el registro de las vacunas de los perros, o sea, vinieron de otros países a ayudarnos con los perros a rescatar a la gente, a buscarlo, pero no los dejaron entrar porque no tenían su registro de vacunas que exigía sanidad animal en el aeropuerto. O sea, imagínense, ya estamos en una situación difícil. ¿Qué ha pasado? ¿Se ha modificado algo de ese procedimiento? No, todo sigue igual, igualito, no ha habido ninguna modificación. Entonces, yo estoy seguro que, si mañana hay un terremoto en Lima, después de 5 años, estaremos lamentando no haber hecho nada, porque es así. Y ahorita estamos viendo la fiebre del dengue en el norte, en Lima, en todo el sur, y el procedimiento sigue igual, no hay ninguna modificación, todos siguen protegiendo no sé a quién, ¿no? Algún día quisiera saber a quién cuida tanto DIGEMIT, ¿no? o el Ministerio de Salud, ¿no? ¿Por qué hay tanta traba? ¿Por qué tanto a las donaciones?

Yo digo "Si estamos trayendo para ayudar la deficiencia que existe, ¿por qué hay tanta traba?" No sé… quisiera tener esa respuesta de parte de los funcionarios, que me den la razón por la cual es tan difícil traer ayuda humanitaria en el Perú. Mire, yo he traído… todos los productos que he traído, hasta órganos para trasplante, ropa, zapatos, juguetes, he traído semen de bovino para mejorar... he traído en este tema de la pandemia, hemos tenido plantas de oxígeno, concentradores, ventiladores, de todo, ¿no? Pero no he encontrado nunca una facilitación de parte de las autoridades cuando ocurre una emergencia o desastre, donde tienen que ponerse ellos en el lugar de que estamos en una situación tan difícil que… Por ejemplo, yo no tenía personal, mi personal estaba contagiado. Entonces, ¿cómo hago para seguir todo el procedimiento que ellos establecían o exigían? Ni ellos mismos podían ir a hacer la verificación, pero, sin embargo, exigían algo que nos entorpecía el trámite, ¿no? es complicado, la situación de estos funcionarios tiene que cambiar la mentalidad de todos… y prepararlos. Como le digo, si hay una emergencia, vamos a lamentar mucho no haber hecho nada.

**I: Entiendo la dificultad, por lo que usted me ha ido comentando, en las instituciones públicas, pero ¿ustedes, qué lecciones han aprendido, qué aprendizajes han tenido como una institución que facilita el tema de las donaciones, viabiliza las donaciones? Y también como parte de Respira Perú, en este tema concreto del oxígeno.**

E: Sí, mire, hemos entendido—bueno, hemos aprendido a adaptarnos muy rápido, o sea, la experiencia nos ha servido a adaptarnos porque fuimos la primera institución en adaptarse al procedimiento virtual, por ejemplo, cuando la emergencia lo ameritaba, ¿no? O sea, Cáritas estaba preparado, si usted se acuerda—usted se pregunta qué institución está preparada para participar dentro de una situación de emergencia, no hay ninguna. Yo he estado en varias reuniones donde les he preguntado a los funcionarios "¿Cuántos de ustedes han estado en 4 o 5 emergencias, dirigiendo la emergencia?" Nadie, todos cambian, cambian; viene el gobierno, cambian, ¿no? Si el 2007 ocurrió un terremoto en Arequipa, a los 4 o 5 años por otro, ya los funcionarios son diferentes. Son diferentes, tienen otra... no tienen ese presente, ese enojado de todo lo que ha ocurrido. Sin embargo, nosotros seguimos manteniendo el personal, la experiencia; y lo que hemos aprendido en estos años es a ser más proactivos, nos ha abierto…

Si bien es cierto, la emergencia me ha permitido, por ejemplo, en mis veintitantos años de experiencia, nunca tuve la oportunidad de reunirme con todos los funcionarios de aduana, con toditos, todas las intendencias de aduanas nos abrieron la puerta. Por ejemplo, eso sí fue increíble, que la SUNAT ponga a disposición todas las intendencias de aduana a nivel nacional a apoyar, ¿no? Pero, como le digo, me decían "Ya, te vamos a brindar ayuda, pero que DIGEMIT dé la autorización". Era increíble, es como siempre escucho un chiste cruel que dice "Mamá, quiero ver la televisión" "Ya, pero no la prendas", ¿no? Tenía mi televisor ahí, pero no lo podía prender, ¿no? Entonces, era así porque yo tenía el televisor, tenía todo, pero DIGEMIT decía "No, no tengo funcionarios, tiene que tramitar el permiso. Me falta esto, lo otro"

“Oiga, estamos en una situación difícil, ¿no?” Nunca cambió, ¿no? pero los funcionarios de SUNAT sí fueron muy proactivos. Como le digo, de todos mis compañeros que hacen comercio internacional, estoy seguro que soy el único que debe haberse reunido con todos los intendentes de SUNAT a nivel nacional; y eso nos ha permitido ser… siendo Cáritas una institución líder con bastante prestigio y es lo que cuidamos nosotros, ¿no? Por ejemplo, porque como le comento, somos supervisados por la Contraloría, la SUNAT, el Ministerio de Salud, el Congreso, y nunca hemos tenido inconvenientes, ¿no? Y espero que la institución siga, que yo pase de esta institución ya algún día no esté, pero que mis compañeros sigan con este legado que tiene Cáritas sobre la solvencia y la credibilidad que tiene Cáritas.

**I: Ya como para ir cerrando, hemos conversado acerca de la labor de ustedes y también de todo lo que ocurrió a nivel de las instituciones públicas. Pero, ¿qué pasó con las empresas? Me gustaría saber porque, en ese entonces, estaba Lindley, estaba alguna otra empresa también. ¿Cómo fue el trabajo? Al principio, usted me comentó que ellos tenían como facilidades, podían importar. Pero ¿cómo fue su…? ¿Cuál fue su rol en ese tiempo de pandemia por el tema del oxígeno de manera concreta?**

E: Hubo una controversia. No sé si usted se da cuenta, la norma del oxígeno medicinal exigía un documento, entonces este nivel de pureza del oxígeno sólo lo cumplían 2 empresas, nada más; en concreto, Lindley y otra empresa.

**I: ¿Lindley y quién?**

E: (Ininteligible, MINUTO 1:08:37) eran dos los que tenían el… digamos, el monopolio del oxígeno en el Perú. Eran 2, nada más, y el resto, ninguno podía llegar. Si usted… en el estudio va a darse cuenta que todas las plantas de oxígeno que han ingresado en la pandemia solo brindan un nivel de pureza del 93%. No llega a ninguno al 99 ni el 100% puro. Lo cual era un tema político porque el elevar el nivel de pureza del oxígeno estaba siendo direccionado a 2 empresas que sí cumplían ese requisito, ¿no? era tan… Está claro lo que estaba ocurriendo porque era como darle el camino solo a ellos 2 porque el resto no iba a cumplir, pero sí el oxígeno al 93%, sí sirve para el tema medicinal, ¿no? Como se ha utilizado, ha servido para tanta gente, poder pasar el COVID, ¿no? porque, si no hubiera llegado una de estas plantas, si no hubieran abastecido de ese oxígeno al menos al 93%, que estaba exigido, permitió ingresar, no hubiese salvado a tanta gente. Seguro las cifras serían pero alarmantes, por no decir que, digamos, direccionada a 2 empresas por hacerles el favor, en otras palabras, iban a perjudicar a todos, ¿no? Entonces, DIGEMIT sacó un comunicado indicando que iban a permitir el ingreso de plantas de oxígeno que no llegaban al nivel de pureza y era excepcional y todo, ¿no? pero, ante tanta presión con el gobierno…

Seguramente en el estudio van a encontrar un tema que hubo con el obispo de Huancayo con la planta de oxígeno en La Oroya, ¿no? Seguro eso que debe estar entre uno de sus puntos en un capítulo, pienso en ponerle porque ahí marcó el delito, ¿no? del cual salió el tema de Respira Perú, ¿no? ante la indignación de querer utilizar esa planta inoperativa en La Oroya porque había una planta inoperativa en La Oroya y el obispo es el que empezó -un señor Pedro Barreto- empezó a alzar la voz y decir que cómo es posible, ¿no? que exista otras plantas y no tengo operatividad, estén operativas, ¿no? Incluso ellos pedían la administración de esas plantas, “Si no lo van a utilizar, la gente con sus balones ahí, durmiendo en la calle, y era increíble que no quieran o no permitir que ingresen otras marcas, otras plantas de oxígeno que podían darnos ese oxígeno que necesitábamos”.

**I: ¿Diría usted que hubo, por detrás de toda esta normativa, un interés económico, una presión fundamentada en lo económico, para que las autoridades no den pace o… sí, pues, no den pase a la posibilidad de recibir estas plantas de oxígeno con un nivel de pureza menor al que supuestamente debería ser?**

E: Sí, sí, pienso que sí. Por ejemplo, usted, por ejemplo, cuando entra…

**I: Discúlpeme, no le estoy escuchando bien.**

E: Si, por ejemplo, cuando… hoy en día, nosotros podemos entrar al computador y comprar cualquier producto en el mundo, ¿no? Tengo un familiar, por ejemplo, que sufrió COVID, y hoy en día no puede salir a la calle porque necesita un concentrador portátil para salir a la calle y tener… la SUNAT pone un requisito, por ejemplo, ese producto sigue siendo restringido. Necesita una autorización de DIGEMIT. Entonces, tú lo puedes comprar acá en cualquier lugar y lo vas a adquirir a un precio mucho mayor del que puedes conseguir en las plataformas de hoy en día de compras online. Entonces… pero no se te permite importarlo directamente. Tú no lo puedes traer, tienes que tramitar un requisito, que es una autorización sanitaria a DIGEMIT. Entonces, en una situación, digamos, que vivimos ahora, bueno, es un procedimiento normal, o sea controlado y todo. En una situación de emergencia, ¿por qué no cambia esto? ¿Por qué no nos damos cuenta que el hospital está lleno, abarrotado, está… no hay capacidad para él; la población empieza a salir a la calle a buscar oxígeno, no encuentra? Pero sí podríamos comprarlo y traerlo de cualquier parte del mundo, podemos comprar y traernos un concentrador que va a servirnos en la casa, pero no lo podemos traer tampoco por un tema de procedimiento.

Entonces, es ilógico, o sea el mercado se va abriendo al mundo y tenemos que ser más proactivos, ¿no? tenemos que modificar nuestros procedimientos, permitir que la gente pueda autodefenderse, ¿no? porque si el Estado no lo va a poder defender, entonces yo no puedo abastecer a todos de concentrador de oxígeno, ¿no? Pero ¿qué tengo que hacer? permitirle a la gente que lo pueda traer, y eso que le va a servir Estado, que lo va a aliviar de la presión que ellos tienen sobre eso, ¿no? Entonces, esto es… es cada día, pan de cada día. Mucha gente que ha padecido del COVID está con problemas de respiración, y van a necesitar estos concentradores portátiles para poder tener una mejor calidad de vida, ¿no? Entonces, se debe mejorar eso, pensar en esa gente y permitirle que puedan traerlo porque, de esa forma, vamos a darle mejor calidad de vida a todas esas personas que han sido contagiadas por el COVID, ¿no?

**I: A ver… usted… es como evidente el problema que hay en la actualidad, incluso post-COVID para las personas, ¿no? Pero ¿cuál cree usted que sea la razón por la que todavía esta normativa o hay estas barreras, estas dificultades para que, digamos, la población pueda beneficiarse o tener acceso a poder comprar, importar de manera directa ese equipo, que es tan básico y fundamental para sostener su vida?**

E: Sí es un tema económico, o sea, de aquí… detrás de esto, hay una protección a alguien, a un empresario, a un importador que tiene el monopolio de algo y no se permite, ¿no? O sea, siendo tan… digamos, en una situación como le digo, normal, bueno, ya, se permite, todo; pero en una situación de emergencia, sale esto a hacer tan evidente que se está protegiendo a algún empresario y darle la… digamos, el aprovechamiento de una situación tan complicada. Pero, no se dan cuenta de que finalmente se evidencia tanto que indigna a la población. Vamos a estar en una situación donde ya no es unos cuantos los que se enferman, sino masivo, conseguían del este proveedor, no va a poder abastecer a todos. Y si ya no puede abastecer a todos, quiero seguirlo protegiendo, estableciendo requisitos o inventándome, como en el caso de las plantas de oxígeno que le comenté, donde arancelariamente ese producto no era restringido, pero cuando empieza a complicarse y la población se levanta y empieza a agruparse y quiere traer las plantas, ya se convierte en un producto restringido ya. ¿Qué pasó acá? Cómo puede ser posible que, de un momento a otro, cambió las reglas del juego, cuando nosotros quisimos ayudar a que esto se solucione, ¿no?

Entonces es tan evidente que le digo que el mismo importador que nos empezó a proveer de las plantas solicitó una reunión con DIGEMIT, dijo "Oye, yo he traído para tal empresa hace un mes, una planta, y no

me has pedido nada. Y ahora que traigo para Respira Perú, le estás exigiendo esto, ¿no?". Dijo "Llevo plantas de un proveedor de Colombia. ¿Qué pasa acá en el Perú?" "No- me decía-, están locos acá porque si hace poco ha traído para 2 clínicas, algo así, trajo plantas y no pasó nada, ingresó y ahora está aquí. Y ahora que tú la traes como donación, no la puedes sacar". Es jalado de los cabellos. ¿A quién están cuidando? ¿A quién están protegiendo? Pero sí es más un tema económico, yo pienso que eso no debe permitirse en una situación de emergencia y debe ser sancionada, pero drásticamente porque están afectando a mucha población que podría ser tu familia, mi familia y no debe permitirse… la ley no contempla eso. Solo de los funcionarios públicos, la ley 28905 habla de sanciones drásticas con los funcionarios públicos que entorpezcan el ingreso de donaciones, pero, como le digo, el COVID no está reconocido dentro de la ley y ninguna emergencia por… sanitaria, no existe; solamente en el Perú hablamos de emergencia y desastre natural. Y, entonces… ¿pero el COVID no es una emergencia? sí, pero no es natural, es una emergencia sanitaria. Entonces… digamos, ellos se lavan las manos y a seguir el procedimiento normal.

**I: Ok. No tengo más preguntas. ¿No sé si usted quiere hacer algún comentario final sobre el estudio, algún aporte o alguna… algún aspecto que yo no le haya preguntado y que usted considere que es importante señalar para el estudio?**

E: Bueno por experiencia, espero que les vaya muy bien. He participado en este tipo de estudios, como le comenté, con la Cruz Roja, con otras organizaciones, importante hacerle ver a la opinión pública, a las autoridades lo que ocurre, ¿no? Y no esperar que venga otra pandemia, otro desastre tan terrible para darnos cuenta que no hicimos nada, ¿no? Me acuerdo mucho de un mensaje de Confucio que decía, ¿no?

**I: Perdón.**

E: Hay un sabio chino, Confucio, que decía, ¿no? "Si ya sabes lo que estás haciendo mal y no haces nada, entonces estás peor que antes", ¿no? Y eso es lo que nos ocurre, ¿no? Y espero que no nos pase. Estamos viviendo ahorita el tema del dengue y nos encontramos con la misma pared ahí, pero que uno dice "¿Que pasó acá?", ¿no? que cuántos quieren que mueran, más gente que el (Ininteligible, MINUTO 1:20:47) para recién darse cuenta. Todos los días vemos las noticias y es indignante que no hagamos nada, todo siga igual. "¿Y a quién protege?", yo digo, ¿no? porque no estamos trayendo productos para hacer competencia, ¿no? sino para ayudar a la gente que más lo necesita, que ahorita no tiene acceso a un medicamento para la fiebre porque se agotaron. En el Perú ocurre eso, ¿no? muchos de nosotros… va a haber fiebre, hay que comprarnos Paracetamol, hay que compramos Tylenol, hay que guardarlo, lo guardamos. No lo necesitamos, pero ya lo tenemos, por si acaso. ¿Y qué ocurre con eso? Hemos ocasionado una escasez de ese producto. Entonces, nosotros lo traemos para ayudar a esa gente, no para competir comercial con ninguna empresa, una farmacia, ¿no? Si no, traemos eso para distribuir a la gente que no tiene acceso o está… en este caso, tengo dinero, quiero ir a comprar, en Trujillo, un Paracetamol, y no hay. Entonces, lo van a recibir gratuitamente por parte de la Iglesia. Entonces, no hay competencia, no hay nada, ¿no? entonces entender eso, ¿no? Y ayudar a las organizaciones, no solo a la Iglesia, sino las otras organizaciones que participan en la ayuda humanitaria, darle las facilidades para que puedan hacer su trabajo, y de la mejor manera, ¿no? Eso, más que nada, y mucha suerte en su estudio. Y gracias por considerarme en esta parte de este tema que me apasiona mucho.

**I: Ok, muchas gracias. Señor E, una pregunta, si nosotros quisiéramos conversar con alguien más de este equipo que se formó en Respira Perú, ¿con quién podríamos conversar además? ¿Tal vez con la… con alguien de la Sociedad Peruana de Empresas? ¿A quién nos podría sugerir usted con el que pudiéramos conversar sobre estos mismos aspectos? ¿No? que hubiera visto un poco los cuellos de botella, las dificultades, mirar las lecciones aprendidas…**

E: Sí, hay una persona justo en la Sociedad Nacional de industrias que estuvo a cargo del tema casi de coordinación, era Carlos García Jerito. Él trabaja en la Sociedad Nacional de Industrias, y él es el—era el principal coordinador entre todas las instituciones. Él estaba en las reuniones con el ministerio, con la sociedad, con los empresarios, y él es el que sí le puede dar mucha información respecto a este tema porque ha estado ahí en el, como se dice, el campo de batalla. E incluso me acuerdo que en una reunión que tuvimos, les decía… porque al final de la campaña, casi hubo una reunión donde todos los involucrados participamos y les decía "No nos despidamos porque esto no va a acabar por 4 años, no, porque ahora viene lo más difícil, cuando… después de haber hecho tu trabajo de la mejor manera, empiezan a cuestionarlo, empiezan a dudar de lo que tú has hecho" "Pero si he hecho bien mi trabajo" "Sí, pero otra organización no hizo bien su trabajo, aprovechó el momento, y por eso todos los que participaron van a ser supervisados". Y uno dijo "¿Y yo qué tengo que ver en la municipalidad de tal distrito que fue denunciada?" "Pero como ellos fueron, ahora todas las organizaciones van a entrar dentro del proceso de control". Entonces les decía esto: "Por 4 años no van a estar tranquilos. Después de 4 años, ya pueden… ya podemos hacer un acto de clausura y despedirnos, pero antes no. Si esto sí es parte del trabajo que realizamos y tenemos que estar ahí, ¿no? es parte del procedimiento y ya estamos acostumbrados a ese progreso".

**I: ¿Y usted… es posible que usted me pueda proporcionar su contacto como para tratar de comunicarme con él y ver si acepta también participar del estudio? ¿Es posible para usted?**

E: Sí, le voy a mandar por WhatsApp su correo electrónico para que le escriba y pueda contactar con él.

**I: Ya, perfecto. Bueno, muchas gracias. Gracias por su tiempo, su disposición para colaborar con el estudio.**

**FIN DEL AUDIO.**

**Entrevista Estudio Oxígeno Medicinal. UPCH 22-06-2023**

Meeting started: 22/6/2023, 18:44:52
Participants:
I: Investigador
E: Entrevistado

## Transcript

**I: Para ir conociendo su labor y también dejarlo registrado aquí, me gustaría que nos comente labor que tuvo durante la pandemia con el tema del oxígeno medicinal. No sé si como representante de la OPS o tal vez por alguna otra institución, como para darle un contexto a la conversación que vamos a tener.**

**E**: Ajá. Bueno, si yo trabajo en el programa subregional para América del Sur de la OPS y durante la pandemia me desempeñé como consultor de este programa, nuestras actividades, que son básicamente… que estaban muy vinculadas a reuniones y trabajos entre países como comprenderás de vio truncado porque se suspendieron todos los viajes y todas las reuniones presenciales. Sin embargo, empezamos a desarrollar las actividades virtuales en las cuales se empezaron a dar interacciones entre países. Nuestro programa, por su naturaleza, también trabaja en actividades de cooperación Sur-Sur. Por otro lado, si bien es cierto, en nuestra oficina todo se hizo por teletrabajo, estuve muy de cerca siguiendo las actividades en el ministerio, porque mi esposa trabaja en el Ministerio de Salud, en esa época estuvo… ella nunca hizo teletrabajo, estuvo trabajando en (ininteligible) como comprenderá también estuvo un poco al tanto de lo que pasaba… comentaba los distintos desafíos, problemas, así como también alternativas que estaban desarrollando. Por esa vía tengo éstas dos aproximaciones, el tema del oxígeno en el Perú.

**I: Usted me dice que estuvo como consultor para el Programa en ése momento.**

**E**: Así es.

**I: Y cuál fue la intervención propiamente de la OPS en el tema puntual del oxígeno medicinal, hubo alguna intervención, no sé**

**E:** Bueno, sí. Primero hubo muchos pedidos sobre cuáles son las especificaciones técnicas del oxígeno que se debe usar. Entonces la organización, así como en otros temas, acuérdese que la pandemia fueron muchas cosas nuevas, rápidamente sacó guías, guías que fueron usadas por los países para orientar en el uso desde medicamentos, el tema de la vacuna y también se incluye el tema del oxígeno. Desde la Organización Panamericana de la Salud se trató muy rápidamente de brindar guías y orientaciones en todos aquellos temas que estuvieran vinculados al manejo de la pandemia, y eso incluía, evidentemente, el tema del oxígeno, entonces eso lo hacía la sede Central, la oficina central, a través de sus diversos técnicos, que daban estas orientaciones, que los países utilizaban. En el Perú, la interlocución directa de la organización estaba hecha a través del representante de la OPS, que en ese momento estaba a cargo del doctor Rubén Mayorga, que fue además el responsable del programa Subregional para América del Sur. Entonces, en un momento dado, él estaba como jefe del programa sur regional y también como representante. Como comprenderá, las labores de representantes lo absorbieron mucho más, era evidentemente mayor la demanda y el trabajo … ese era el rol, el acompañamiento técnico al Ministerio, permanentemente. Salía con a dar conferencias de prensa en compañía, al costado del representante, y el representante, pues daba la perspectiva de la organización, esto un poco para dar también mayor respaldo a las acciones que el Ministerio estaba haciendo.

I: **Éstas guías de manera puntual, porque por ejemplo me habla usted de medicamentos, de vacunas, de oxígeno, pero estás que están vinculadas al oxígeno, qué contemplaban está guías, porque hubo todo un debate las exigencias, que si tenía que ser al 96%, todo ese tema, pero no sé si es lo que incluían esos temas, qué es lo que se había considerado en este documento.**

**E**: Si, como comprenderá nosotros no entrábamos dentro de las… digamos, debates nacionales, que si bien tenían algún un componente técnico, había también algún trasfondo político. Pero las guías fueron, digamos, muy generales. Se utilizaron... yo te voy a compartir acá la guía que se sacó en 2021, para que la puedas ver, voy a pasarte el link en realidad para que no pese demasiado, y bueno, salí esta guía publicada y el representante entregaba estas guías a las autoridades y cuando había alguna consulta específico, puntual, normalmente la representación ponía en contacto a las autoridades con el experto en el tema, desde Washington o desde el país donde se encontrara. Este era más o menos el funcionamiento de cómo se hacían las cosas. Como se comprenderá, no había misiones en terreno, estaban suspendidas, los viajes, pero había algunas situaciones en las que, por excepción se hacía esto, pero en otros países, no fue el caso del Perú. Entonces, eso era lo que se hacía, pero, repito, no se… no se tuvo mucho cuidado en polémicas que tuvieran algún tinte político... era eminentemente técnico

**I: ¿Esta guía que se presentó en el Perú fue la misma que se manejó en la región?**

**E:** Claro, las guías no son para un país en particular, son guías para toda la región y cada país la da, no? cada país la da, entonces se daban estas guías y que intentaban justamente responder a las principales inquietudes o dudas que pudieran haber en relación a estos temas, no? Pero, cuando eso ocurría, se apelaba a hacer el contacto con el experto. Normalmente los expertos no necesariamente estaban en el Perú, podían estar en otro país y se hacía en contacto con los expertos, cuando era requerido. En la mayor parte de veces no era requerido, porque el Perú tiene suficientes expertos para saber cómo hacer uso del oxígeno. Entonces, no era el caso tanto para el Perú, que tiene muchos expertos, entonces, no fue el caso para el Perú. Pero, estas guías fueron las que se usaron. Y hay una orientación especial? Sí, acá están las guías, pero no lo usaba como para decir qué es lo que tengo que hacer, simplemente para "miren lo que estoy haciendo está acorde, a lo que dice, la Organización Panamericana de la Salud". Para eso lo usaba. No, porque tuviera que venir alguien a enseñarle. El misterio tiene funcionarios y personal con suficientes conocimiento y capacidad para para saber lo que tiene que hacer.

I: **Digamos que ésta guía sirvió como respaldo para los especialistas y expertos para respaldar su trabajo, que venían haciendo.**

**E**: Así es. Habitualmente ese era el caso, no. Muy pocos países necesitaban tener algún experto que les enseñe. En las Américas, hay países con mucho rezago donde tenía que venir alguien a enseñar. No ha sido el caso de Perú, como en otros países, no hay, no había que venir acá alguien enseñar, teníamos expertos que sabían cómo usarlos, así que no fue ese el caso peruano.

**I: Y ésta guía fue la única o luego se revisó, evaluada, revisada?**

**E:** Normalmente las guías se hacen después de una muy minuciosa revisión bibliográfica, entonces no es el caso de medicación o vacuna que había que estar actualizando, porque salían nuevas cosas, el oxígeno es un recurso que se conoce desde hace mucho tiempo, así que se alineaba, no más con lo que decía la OMS y se publica. Entonces, no es que haya tenido que tener muchas actualizaciones. El oxígeno es un recurso que se usa desde hace muchos años, y el uso puntual y especial en cada paciente eso ya es un tema clínico y allí no entra la OPS, en los temas clínicos ya es un tema netamente científico, lo hacen los especialistas. Ahí no se mete la OPS. Las guías clínicas las maneja cada gobierno, cada país, con sus expertos, sus clínicos, sus sociedades científicas, etc. Entonces no es que haya que, a diferencia del uso

de vacunas o el uso de mascarillas o el uso de … que se actualizaban porque eran cosas nuevas. En este caso no, no hay, no hay mucha mayor ciencia en el uso del oxígeno muy, bien, muy antiguo y muy y muy claro.

I: **Durante la pandemia surgieron diferentes debates a cerca de, digamos, estas empresas que tenían como el monopolio de la producción de los balones de oxígeno, la compra, la adquisición de plantas, en fin todo un tema allí. Me remite a pensar… si bien es cierto la guía fue como suficiente en ese momento, como una pauta para establecer la parte técnica, pero fue como suficiente para el momento en que estábamos, que se veía al principio, tan compleja y complicada, con la falta de oxígeno en todo el país o siente que se habría necesitado algo más. Entiendo que su labor es más de dar las pautas, entendiendo también de la información con la que contaba, estando más cerca de la información a través de su esposa, por el trabajo que ella realiza. Siente usted que fue suficiente, que se podría haber hecho más a través de la OPS o tal vez de alguna otra organización.**

**E**: En realidad no, porque el problema que tuvo el Perú con otros países era abastecimiento y generación de oxígeno. Entonces era un problema netamente interno y de carácter más logístico. El oxígeno no está dentro de la lista de productos que se incluyen en algún fondo rotatorio, porque la OPS tiene fondos rotatorios para comprar vacunas, medicamentos, en fin, el oxígeno, no. Entonces no estaba en nuestra… en nuestra, digamos, capacidad de acción, decirles cómo fabricar oxígeno, cómo producir oxígeno o de dónde conseguir oxígeno. Y entonces no, no se dio allí mucho allí. En un momento dado, ante la falta de oxígeno hubo el interés de traer oxígeno de fuera y, en concreto de Bolivia, entonces por allí se hizo algunas articulaciones, pero como esto era un tema de gobierno, gobierno, la OPS sólo funcionó como puente, no porque eran directamente acciones de gobierno. Entonces, en algún momento inicial, ante la falta de oxígeno en la zona sur se transportó oxígeno en camiones desde Bolivia, pasando por Chile para el sur peruano, pero repito, eso fue una acción directa y entre gobierno a gobierno, entre gobiernos, igual también hubo la gestión con Chile porque claro tenía que pasar por Chile, también Chile en algún momento ofreció donar al oxígeno. Entonces, Perú mandó camiones o cisternas y regresaban cargadas de éxito, pero esas eran acciones directas de gobierno a gobierno. Ahí, no es que la OPS vendiera oxígeno o transportara el oxígeno. Eran acciones que se hacían directamente de gobierno a gobierno.

I: **Cómo ve el manejo que se dio tanto a la producción como a la adquisición que se realizó en ese entonces o lo que se pudo hacer en ese entonces por el tema del oxígeno.**

**E**: Claro. Bueno, en el caso si registramos …si recordamos el Perú, de un momento a otro, empezó a hacer convenios para montar plantas generadoras de oxígeno y hubieron desde iniciativas privadas como el Vicariato de Iquitos que compró e importó directamente una planta de oxígeno, y así algunas iniciativas privadas y el gobierno lo que hizo es hacer un convenio con la UNI para tratar de que la UNI o la Católica fabricarán plantas de oxígeno y se las entregaran, para que el gobierno las distribuya entre los principales hospitales del país, de las principales ciudades. Entonces, claro empezar de 0, eso demoró porque no se fabrican plantas de oxígeno de un día para otro y fueron acciones concretas y directas del gobierno. Se hizo lo que se pudo, eso era lo que había que hacer y mientras tanto, apelar al oxígeno que hubiera. También, el Misterio compró gran cantidad de concentradores, éstas máquinas que lo que hacen es filtrar el aire y concentrar el oxígeno. Entonces, la estrategia del Ministerio estuvo, por un lado, proveer de estas de estos concentradores de oxígeno a los establecimientos de salud, donde hubiera electricidad, y simultáneamente montar plantas de oxígeno en las principales ciudades y hospitales para que abastecieran de oxígeno a los servicios de salud y a la población. Estas fueron las dos, pero no fue suficiente. Entonces la gente misma buscaba comprar oxígeno porque… eso también fue vendido como bien adicional en plantas privadas. Y eso, obviamente, fue disminuyendo en la medida que se fueron montando las plantas productoras de oxígeno, fue esa la estrategia. Recordemos que el oxígeno era un

recurso vital porque, como parte del tratamiento, sobre todo en los casos COVID moderado o grave, el principal instrumento terapéutico era el oxígeno.

I: **He venido ya conversando con algunos representantes de instituciones y organizaciones que estuvieron vinculadas al tema y por lo que me han comentado hubo una dificultad importante, por ejemplo, para importar las platas de oxígeno, había toda una dificultad, por un lado, sobre todo, por las exigencias técnicas que se expresaron, exigencias que antes de la pandemia y antes de generarse toda esta situación tan complicada de la necesidad de contar con oxígeno no eran requeridas por el MINSA. Entonces, hubo todo un tema. No sé si usted ha tenido información acerca de esto, no sé si podría comentarnos algo al respecto.**

**E**: Eh... no. No tenía mucha información al respecto, pero en lo que sabemos en general, cualquier producto que vaya a ser usado en personas, seres humanos, debe tener una aprobación o registro, registro que en este caso nos da la autoridad rectora, el Ministerio de salud, pero hubo mucha informalidad en ese momento o sea hay muchas plantas que se montaron y empezaron a funcionar sin tener ningún tipo de autorización ni nada, porque simplemente la gente se moría. Cuando la pandemia empezó y digamos, avanzó un poco más, empezaron a hacerse procesos más este… se trató de regularizarse esto y ahí es donde se presentaron las dificultades … porque también venía cualquier persona y decía que podía montar cualquier planta oxígeno sin… necesariamente tenía que cumplir algunos requisitos. Acuérdese que hay un oxígeno industrial y uno medicinal, que son diferentes. Entonces eso era… y eso que no éramos, decíamos, el órgano técnico competente y el ministerio tiene esa responsabilidad y, por lo tanto, yo creo que al inicio la verdad es que nadie buscó ni miró si tenía o no, las montaron y era una cosa de vida o muerte y ya cuando después estuvo más regularizado y empezó a intentar hacerse una comercialización de eso para empezar a tener plantas, venderlas, comercializarlas… ahí es donde ya el gobierno se puso un poco más firme. Ahora sí vamos a ordenar esto, necesitamos que esto cumpla algunos criterios y si no cumples estos criterios, no estás autorizado a poder vender y menos montar estas máquinas. Pero al inicio, no fue así. Al inicio, era que haya oxígeno. Lo primero era que llegara sí o sí oxígeno y en su momento. Recuerde que después empezaron a aparecer privados queriendo traer máquinas productoras de oxígeno, y ahí es donde se tuvo que poner cierto orden, por ejemplo, también hacer cumplir unas especificaciones técnicas. Y, claro, se las vendían instituciones privadas o públicas, no? desde municipios, instituciones privadas que los ofrecían para darlos. Ahí es donde se empezó a tener buscar, mantener un orden. Qué es lo que tiene que hacer el ministerio para garantizar también la seguridad de las personas y de los lugares donde se montaban esas plantas que no son totalmente inocuos, tienen sus riesgos si no se manipulo o se maneja bien o no está bien, y más criterios básicos de seguridad.

**I: Entiendo que la labor de la OPS es más orientada a dar éstas pautas, éstas guías para el manejo, el uso, pero en algún momento ustedes habrán tenido, no sé si la tarea, la labor o la intensión de mirar cómo estaba la supervisión de cómo se iban implementando las plantas, los concentradores de oxígeno. O sea, no sé si hay una labor que va más allá de la normativa, de establecer pautas técnicas.**

**E**: No, no, no, no, ahí no la OPS habitualmente no se mete en esos temas.

**I: Ni acompañaron el proceso para implementar.**

**E:** Lo más que hubo, porque la OPS también logró movilizar fondos, compró concentradores de oxígeno y las entregó al ministerio para que el Ministerio les diera uso, eso fue lo más que hizo, compró concentradores, eventualmente balones de oxígeno, pero que yo sepa no donó plantas de oxígeno. No, no es parte… no ha sido parte del trabajo ni tampoco entró en la parte en esta con estos elementos regulatorios. En eso, el gobierno no es un tema técnico en el que tampoco haya pedido apoyo o asistencia

técnica a la autoridad. No, ellos tienen todo eso muy bien estructurado y no necesitan … por allí en algún momento de repente 'bueno, oye cómo están manejando este tema en otros países'. Y por ahí la OPS, se dijo, miren tal país está haciendo así, eso sí impulsó muchos seminarios, muchas reuniones con autoridades sanitarias para intercambiar qué es lo que estaban haciendo y allí el Perú, junto con otros países, supieron cómo estaban haciendo otros países para el tema del oxígeno, pero no es que la OPS lo diera, era promovido el intercambio de información entre los mismos países, era información de estos países y cada país decía, mira yo el oxígeno manejo así, yo estoy consiguiendo esto, esto. Y, claro, el Perú aprovechaba esas otras experiencias y permitía luego establecer relaciones bilaterales directamente el Gobierno peruano establecía contacto con tal o cual país para tener mayor detalle de algunos elementos, pero no es que la OPS se haya superpuesto como el único que diga cómo, no, no suele hacer eso.

**I**: **De los concentradores, que la OPS donó sabe usted cuál fue el paradero final, a dónde los llevaron.**

**E**: Bueno, desde que se entregaron al gobierno y el gobierno las distribuyó dependiendo de sus ámbitos, las distribuyó a los establecimientos de salud. Todos todos.

**I**: **Pero ustedes no saben a dónde, si funcionaron bien.**

**E**: Si, si claro, se hacía entrega el ministerio y el ministerio generalmente decía estos son para tal región. Por ejemplo, se tuvo un proyecto en Ancash, entonces, a través del proyecto se hizo muchas cosas, también hubo otro proyecto para Tumbes. Entonces, donde la OPS se tuviera trabajo, entregaba estos equipos e iban a allá, a través de las autoridades sanitarias. Y claro, siempre iban hacia establecimientos de salud y cuando se hacía las entregas, pues había, generalmente, actos oficiales de entrega, con actas de recepción, o sea, eso es siempre que se hace eso, hay un acta de donación y de recepción, que garantiza que el producto el bien va a ser rastreado hasta el destino final. En fin, eso es solo eso lo maneja bien el ministerio, pero no es que estuviéramos detrás para ver dónde está, no… allí hay mucha confianza con el ministerio.

**I**: **Y usted sabe si esos concentradores todavía están siendo usados.**

**E**: Bueno, bueno con la mayor parte de estos equipos o concentradores que llegaron a los establecimientos, se usaron intensamente en la pandemia, pero después su uso se ha reducido, no… no sabemos… o sea, si me preguntas siguen ahí, están función. No sé, no sé, tampoco la OPS se ha puesto a mirar y a investigar a ver dónde están, qué están haciendo, lo más probable que estén en los servicios incluido dentro de sus bienes, algunos deben haberse deteriorado malogrado porque tienen una vida media determinada y hay que cambiar filtros, hay que este… no son productos así eternos y algunos deben haberse dado de baja, también porque su vida es media, y otros deben estar funcionando y, digamos, entra ya dentro de cualquier equipo que llega al Ministerio, no? como el tensiómetro, el ecógrafo, un equipo rayos X, ya el Ministerio se encarga. Muchos de ellos funcionan, otros les han de baja, y otros pueden estar sin uso por falta de mantenimiento, pero ya no es un algo que… en el que estemos nosotros de detrás, no es que hayamos hecho algún estudio, no.

**I**: **La compra de vacunas y medicamentos por parte de la OPS está como presupuestado, establecida como necesidad, pero la pandemia nos trajo esta nueva necesidad, que fue la del oxígeno. Ustedes compraron éstos equipos, los balones. Entonces, cómo se hizo para tener el financiamiento y comprar éstos equipos, todo lo que logró dar para aliviar la necesidad de oxígeno en el país, porque fue como algo nuevo, surgido a raíz de la pandemia, no es que ustedes tengan un presupuesto destinado para el oxígeno, anualmente.**

**E**: Bueno, primero, normalmente, la OPS no compra vacunas o insumos por su cuenta. Los 5 productos que compramos siempre se hacen por encargos de países, o sea, primero el país dice: yo requiero 1000

vacunas, de este tipo de vacuna. Entonces la OPS lo que hace es recoger las demandas, junta un solo pedido y negocia con un proveedor precio y luego dice: compra a nombre del país, hace la compra global y luego la distribuye, dependiendo de cada pedido que haya hecho el país. Entonces, no es que nosotros tengamos un presupuesto para comprar, el gobierno dice: quiero que me compres tanto, te doy la plata, acá hay, luego te doy la plata junta… y así va juntando y con ese dinero hace la compra conjunta, aprovechando economías cercanas. Pero no es que tengo un presupuesto especial para comprar y luego ofrece y saber quién quiere comprar esto, no, eso no es. Así no funciona… lo hace todo por encargo del país. Entonces, un país dice: ahora quiero que me compres este antimalaricos. Ya! entonces cada país dice: quiero que me compres este antimalárico, toma la plata, junta. Quién más quiere comprar? Otro país más quiere, entonces junta la plata de los países y compra a nombre de los países y luego les entrega. La idea es que al hacer compras en volumen consigue precios más… más a mercado, entonces así se ha hecho, y el oxígeno no es parte de eso, nunca. Y los equipos que pueda comprar, el concentrador o cualquier otro equipo, se hacen siempre en el marco de proyectos, proyectos que son concordados con el gobierno. Es decir, el Gobierno dice: mira, tengo estos fondos que he conseguido de este donante. ¿Qué cosas quieres que te ayude? Y e; gobierno dice: mira, yo quiero que me consigas concentradores, entonces concentradores. Quiero que me consigas tensiómetros, quiero que me consigas mascarillas. Entonces, el gobierno decide, dice: esto es lo que quiero que me ayudes con este proyecto. Los fondos normalmente son de algún proyecto y son proyectos cortos, son proyectos que tienen vida media y que tienen actuaciones muy concretas. En muchas ocasiones el donante dice: mira, te voy a dar este dinero, pero es solamente para tal región, entonces el gobierno dice que destine a esto, pero no es que tengamos un presupuesto que destinemos para comprar oxígeno o concentradores, no, es a demanda y de acuerdo a cada proyecto. Cuando se pone… se negocia con el donante, con el país beneficiario y en un acuerdo se dice: bueno, esto es lo que quieren ya están estos ítems, no es parte de una actividad regular comprar concentradores y entregarles. En pandemia, por la circunstancia, eso se hizo porque era lo que los países necesitaban. Ellos mismos decían, eso es lo que quiero, por favor, y conseguimos donantes y se consigue y es entregado en una actividad regular. Así es.

**I**: **Como una cuestión excepcional por el tema de la pandemia. Cuáles considera que son las áreas centrales en las que se debe centrar cualquier nueva política del oxígeno. La pandemia del COVID-19 nos ha mostrado, a todo el mundo, que tenemos el riesgo de que pueda ocurrir cualquier otra pandemia, en cualquier otro momento. El COVID-19 ya la tuvimos, hemos pasado ese momento, pero podría surgir otra pandemia o esta misma, de manera distinta, en fin. En función a eso es como irnos preparando, la pregunta es si nosotros en este momento tenemos como una reglamentación como país, que esté acorde con lo que hemos vivido o hay la necesidad de establecer nuevas normas, no sé teniendo en cuenta talvez enfoques técnicos, tal vez las necesidades que se observaron durante la pandemia.**

**E**: Lo que habitualmente sobre en el Perú son normas y tiene suficientes normas, tiene suficiente. La sacó en su momento, creo que hubo una respuesta muy ágil, porque rápidamente cubrió vacíos formativos. Es lo primero que hace el Ministerio para garantizar de que lo que se vende en el país esté dentro de algunos parámetros, así que creo que por ahí no va la cosa. Las normas están. Y las normas permanentemente se revisan, entonces el ministerio… creo que hace un buen papel. El gran problema es que a veces hay un exceso de normas y las normas para que sean efectivas, tienen que ser conocidas y ser cumplidas. Entonces, yo creo que ahí, básicamente, racionalizar probablemente las normas. Hay algunas que pueden haber ya caducado, ser obsoletas y hay otras que pueden seguro mantenerse vigentes y permanentemente las están actualizando y revisando. Yo creo que, en ese lado… si algo hacen en el Ministerio de Salud es que saben hacer normas, a veces en exceso. Entonces creo que no es un problema

de falta de normas, es más bien tratar de simplificarlas, sistematizarlas. Entonces yo creo que la cosa va por ahí.

**I: Ok. Cuál diría usted que fueron los retos más importantes respecto del tema del oxígeno durante la pandemia, sé que fueron diversos aspectos, pero desde su mirada si podría señalar uno o dos que usted considere como los más importantes.**

**E**: Bueno, el problema del oxígeno fue el inicio de la pandemia, cuando nos dimos cuenta que la capacidad de producción de oxígeno no se dio abasto para cubrir la demanda, pero nadie sabía no que se venía, entonces era muy difícil tener un país que haya estado preparado, no? y ahí nos dimos cuenta que los hospitales no tenían plantas de oxígeno, muy pocos servicios tenían plantas de oxígeno y se hizo algo que ya va a servir para siempre, se montaron más de 400 plantas de oxígeno en todo el país, entonces ahora oxígeno no nos debe faltar. Probablemente lo que haya que hacer es dar mantenimiento a esas plantas, porque en algún momento se va a utilizar, no solamente para una eventual pandemia en general el oxígeno es un producto necesario, entonces yo creo que es un poco complicado decirle que fallamos porque en realidad fue una falla de todo el sistema. Nadie sabía, no estábamos preparados. Y esto cayó por dentro de la precariedad que tiene sistemas, nos faltaban muchas cosas, también el oxígeno. No era algo… culpa de ese momento de las autoridades, es algo que venía de antes y nos abastecíamos con oxígeno medicinal que se compraba de proveedores. Con eso bastaba, antes bastaba, suficiente era con eso. Probablemente no necesitábamos más. Nadie se puso a pensar porque no tenemos una planta de oxígeno por cada hospital, porque no era necesario, era suficiente comprar. Entonces, llega la pandemia, ee instala con una velocidad tremenda y en pocos meses se cerraron la frontera y no había espacio ni tiempo, no hubo espacio en el tiempo, y cuando se empezaron a hacer los primeros acuerdos o convenios para producción de platos oxígeno con la UNI se hizo un convenio grande, con la UNI, el gobierno dándole fondos para fabricar, también se dieron cuenta pues que no es tan fácil como fabricar zapatos, no. Tuvieron problemas serios, problemas en la producción. Y luego después se vivieron intermediaciones que dieron sospechas de corrupción. Entonces se enlenteció el proceso, en fin, lo típico que ocurre aquí cuando hay estas cosas y se tiende a hacer muy rápido, pero yo diría que tuvimos el mismo problema que tuvieron otros países, estuvimos menos preparados por la precariedad del sistema y, sin embargo, la respuesta también fue, creo yo, bastante rápida, un año, un poco menos de un año de tener menos de 10 plantas a más de 400. O sea, eso fue un esfuerzo descomunal, enorme y también hay que reconocer que se pudo tener 400. De repente dicen, no, pero debieron ser 1 000. Pero tener 400 en el lapso de un año y en pandemia, con todo el cierre de fronteras y todo, no es algo sencillo y ahora tenemos hasta un exceso de plantas, que podrían estar ya malográndose, porque ya no se usan. Entonces, hay que cuidar de que esas plantas les estén haciendo mantenimiento.

**I**: **Pero este tema pasa por no solamente con los recursos, sino también por contar con recursos humanos para el mantenimiento, por tener las capacidades para hacer este tipo de trabajo. Usted que conoce de cerca lo que ocurre en el Ministerio, usted ve que eso se está encaminando o no se está haciendo nada, cómo ve usted el tema**

**E**: Bueno, el tema del mantenimiento de los equipos es un tema... es un problema en general con todos los equipos. El Estado compra, adquiere equipos y una de las dificultades es que no se asigna suficiente presupuesto para darles el mantenimiento. No es una cosa reciente, es una cosa general. Entonces yo creo que ahí radica uno de los principales desafíos. Tenemos una Dirección General de Equipamiento y Mantenimiento que tienen un inventario de todo el equipamiento del Ministerio de Salud, en sus diferentes redes y establecimientos, y ellos deben ser los responsables de garantizar que estos equipos tengan el mantenimiento necesario, pero, lamentablemente, los recursos suelen ser escasos, entonces no tienen la suficiente cantidad de recursos para (ininteligible) … muchas instituciones públicas son

autónomas administrativamente, eso lo tiene que programar cada institución, no lo tiene que hacer el Ministerio del Ministerio en su sede Central, cada Dirección Regional de Salud, cada hospital, cada Instituto, cada red y micro, en su presupuesto debe asignar un rubro, una cantidad para mantenimiento y lo concreto es que no lo hacen. En la última rueda del coche, entonces… y el Ministerio no tiene … no puede presupuestar eso porque le corresponde a cada unidad ejecutora, es así la descentralización. Eso no está centralizado. No, eso no está centralizado. Esto depende de ellos.

**I**: **Si, de acuerdo, pero ahí tal vez pueda entrar la parte normativa, es posible**

**E**: Lamentablemente, por normas las unidades ejecutoras no trabajan, no lo hacen. La norma está, pero la decisión de… en qué presupuestario asignar, son decisiones de gerentes, gestores, donde la prioridad no suele ser el mantenimiento, son otras … son la última rueda del coche y solo se preocupan cuando se malogra el equipo y ahora cómo lo arreglamos. Por supuesto, no hay, peor… y ahí queda el equipo abandonado. Es también un tema de capacidad de gestión, que depende de las capacidades que tengan.

**I: Cómo diría usted que está la situación del oxígeno hoy en el país, se habrá aprendido algo, habrá cambiado algo… los gestores, las autoridades habrán aprendido**

**E**: Bueno, el COVID ha sido un hecho traumático. ¿No? Pero no te tengo que explicar a ti que, a pesar de toda la pandemia, hemos tenido crisis y turbulencia política, sacamos un presidente, sacamos otro presidente, este… entonces eso hace muy difícil hacer gestión y están en cacería de brujas por temas políticos de quien estuvo a cargo de la pandemia al inicio, buscando por cualquier medio responsabilizar a funcionarios que estuvieron en su momento liderando la respuesta. Entonces, el tema es más político que técnico. Las instituciones y los funcionarios… hemos, todos los que han estado allí, han adquirido una enorme experiencia, pero lamentablemente dependemos de los políticos, la estabilidad política del gobierno, los actores gubernamentales del Ministerio y eso lamentablemente, no permite hacer un apropiado aprendizaje, porque en estamos en una inestabilidad política que no permite procesar absolutamente nada, eso no es prioridad. Ahorita la prioridad es como mantener o concentrar el poder, le van a hacer revancha contra mis enemigos políticos, y eso se tumba cualquier intento de sacar aprendizajes y tratar de … ahí hay un tema que genera un ruido enorme y que no permite hacer eso. En general, no estoy hablando sólo sobre el oxígeno, en general. Obviamente es también existe, pero es un tema ya más más político.

**I: Ok. Ya para ir cerrando, si tuviéramos una nueva pandemia cuál sería la situación del oxígeno en este momento.**

**E**: Depende la pandemia, ¿no? O sea, nadie sabe, no las necesidades. Bueno, lo que ahora vemos es que tenemos una autosuficiencia oxígeno, ya no dependemos del oxígeno ni siquiera de privados, ya hay una capacidad instalada que va a servir, si es que necesitáramos oxígeno. Esa capacidad instalada se puede deteriorar? sí se puede deteriorar, pero está, existe. Así que falta de oxígeno en los próximos años lo dudo que pueda haber, hasta que sus equipos se oxiden y se malogren por falta de uso. Pero tenemos la experiencia y sabemos ya cómo hacerlo, entonces, si fuera necesario requerimiento de oxígeno a mediano plazo por cualquier situación o contingencia, creo que estamos mejor preparados para responder eso, a como estamos en 2020.

**I: Qué lecciones clave podría compartirnos a cerca de lo que se vivió en el país, de esta manera en que se reaccionó, las acciones que se tomaron, de todo en general, lo que ocurrió en torno a la problemática del oxígeno en el país**

**E**: Yo creo que el equipo o la respuesta que tuvimos el primer año de la pandemia, con sus vaivenes, incluyendo el segundo año, me parece que fue, bueno el Gobierno Vizcarra y luego Sagasti, que fueron

los que tuvieron en pleno proceso, creo que fue una respuesta institucional que logró salvar muchas vidas, a pesar de la cantidad de muertes que tuvimos. Si no se hubiera respondido, la pérdida de vida serían muchas. Y eso incluye el tema del oxígeno, se hicieron cosas que antes se pensaba en imposibles. Y se logró hacer muchas cosas, el equipo que trabajó para esto hizo un esfuerzo sumamente grande. Probablemente no tienen los créditos de todo el trabajo que se hizo, pero fue, creo yo, un equipo muy profesional, muy consistente, salvo la primera respuesta de un equipo ministerial que no sabía mucho, no estaba al tanto, que estaba más perdido en otras cosas. El equipo que luego se instaló en el Ministerio ya para enfrentar la pandemia, cuando esta ya había empezado, creo que hizo todo lo que pudo, todo lo que humanamente se pudo hacer y logró mover un elefante que… que era... que nadie pensaba que se podía mover con cierta agilidad. Realmente se hicieron cosas, muy importantes y eso, claro, nadie lo reconoce, nadie lo visualiza. Se dice que somos el país que tuvo la mayor cantidad de muertes y que esas muertes sin culpables, pero yo creo que, si ese equipo no hubiera hecho lo que hizo, el número de muertes subiría muchísimo y si tenemos la muestra que tenemos es porque ese equipo justamente sincero las cifras, teníamos un sub registro. Y una de las cosas que hizo el gobierno, en su momento, dijo, no. tenemos que sincerar la cifra. No todos los países lo han hecho, no todos los países han sincerados la cifra de muertos. El Perú fue uno de los primeros países en sincerar el número de muertos y finalmente tener una sola cifra, en base al criterio más fiable que teníamos, el SINADEF. Eso ha tenido costos políticos, sí, claro porque cuando te comparas con otros países que tienen sistemas de información más precarios donde no se registran… entonces, yo creo que sí, hay una lesión aprendida, diría que más adelante cuando pase todo el ruido político y está lucha encarnizada por el poder permitirá recién visualizar el esfuerzo que se hizo en ese momento, ahora es muy difícil, hay mucho ruido político, lamentablemente el momento no es muy apropiado para hacer balances objetivos y neutrales. Hay mucha ansiedad y mucho revanchismo.

**I: Bueno, no tengo más preguntas, pero no sé si usted quiere hacer un comentario final como para cerrar ya la entrevista.**

**E**: No, simplemente espero qué te puede haber sido útil este testimonio, es un testimonio más de parte, pero estaría dentro de los resultados del estudio, así que apenas esté eso me avisas y me la compartes.

# 004 Entrevista

Meeting started: 26/6/2023, 3PM

Participantes: E, I

I: Investigador

E: Entrevistado

**I: Cómo para comenzar y tener la referencia de con quién estamos hablando, de qué se trata su participación.¿Cuál era su labor, su actividad en relación a este tema específico del oxígeno medicinal a través de la empresa minera?¿alguna organización? poco para contextualizar por favor.**

**E:** Sí, yo tengo un amigo de trabajo que… él aperturó una empresa con otro socio y… él conmigo abrió otra empresa. La empresa que él aperturó con su otro socio la hizo también coordinando con la Universidad Pontificia Católica del Perú ¿para qué? para que cuando inició este problema de la pandemia, ellos tenía un proyecto de fabricar plantas de oxígeno, plantas de oxígeno. Ellos habían investigado rápidamente en Lima quiénes tenían plantas, averiguaron y cuándo empezaron ellos a diseñar la planta, ahí es donde ellos me invitan a participar en este…

**I: Equipo digamos**

**E:** Equipo ¿no? para poder desarrollar la parte de las plantas de oxígeno… que en Perú eran bastante escasas,obviamente y también la falta de oxígeno que se veía.

**I:¿Usted se dedica a qué? Usted es ingeniero qué?**

**E:**Yo soy ingeniero civil, trabajé muchos años en empresa japonesa T. que es una empresa que se dedica a la venta de maquinaria de construcción y la minería; yo trabajé en sector minero. Y un compañero de trabajo ,que también trabajó conmigo, salimos casi a la misma vez por motivos de jubilación, prácticamente. Y nos encontramos en esta empresa, él me llamó y me pidió que participara en este proyecto en la Universidad Católica. La Universidad Católica nos cedió la parte de los talleres, como no habían alumnado, no habían asistencia, estaban ocupados. Entonces nos facilitó esa infraestructura donde también tenían obviamente conocimiento la organización la dirección de la Universidad Católica. Ellos estaban interesados porque de una forma… el padre Barreto… las este…organizaciones de la iglesia en coordinación con la con la sociedad en los lugares juntaban fondos para comprar plantas de oxígeno para que sea un evento mucho más rápido porque si se hacía a través del Estado, eso iba a tomar mucho tiempo,las licitaciones, los procesos. Entonces yo me involucré..

**I:Más en la parte técnica, o en la parte administrativa, de gestión..**

**E:**Inicialmente en la parte técnica no tanto, pero si apoyaba para conseguir los insumos para la fabricación y también la planta de oxígeno, una vez que estaban fabricadas había que llevarlas al lugar e instalarlas ¿no?y ponerlas en funcionamiento,entregarselas ¿no? eso se llama una entrega técnica, y de ahí darle a todo el soporte técnico post venta de la máquina. Entonces yo me encargué de en realidad esa parte, yo era el gerente de aftermarket o de post venta. Claro que me tenía que involucrar desde el proceso de fabricación para ayudar también al control de calidad para cuando yo tuviera que entregar la máquina desarmada y a volverla a montar no tenga…

**I: Dificultades**

**E**: Ningún inconveniente, entonces esa fue mi participación ahí en la…este…digamos en este proyecto que… tuvieron muchos problemas; primero por la parte de la dirección, una de las direcciones de ministerio salud que no quería otorgar el permiso o la licencia para poder fabricar las plantas de oxígeno. Este fue un proceso largo.

**I:¿Cuáles eran los reparos,observaciones?de estas dirección del ministerio ¿Cuál era la observación que les hicieron?**

**E:** Primero que… era el porcentaje de oxígeno, que no estaba claro, ni ellos mismos lo tenían claro.Pero había un problema con el porcentaje del oxígeno. Tuvieron que modificar inclusive una norma para que en el área sanitaria de la salud pública, se utilice oxígeno en un un porcentaje entre 91 y 93 lo que antes era hasta 97 creo era el porcentaje. Y eso era un problema porque decían que la planta debería de generar 93% de oxígeno permanentemente, pero la norma escrita en letra chiquita decía de 91 a 93. Entonces eso era uno de los problemas que digamos, que ocasionaba el tema. Y lo otro era un poco en el diseño. El diseño¿no? que ellos sospechaban que no sé contaba con todos los filtros, ¿no es cierto?, para poder asegurar que el oxígeno que se iba a entregar estaba libre de toda partícula, de impureza, de bacterias, partículas pequeñas. Entonces ahí entonces tuvimos que demostrarle a los ingenieros de la DIGESA creo que es, que nosotros utilizamos 3 filtros, 3 tipos de filtros; para pureza,bacteriológico y uno para retirar la humedad. Eso era la…Pero al fin nos dieron un permiso temporal.

**I:¿Cuánto tiempo les tomó a ustedes conseguir o superar esa dificultad que me menciona por el tema de las exigencias?**

**E:**Eso… eso fue como tres meses mas o menos que empezamos creo que en mayo,Julio; mayo fue y en Julio nos dieron un permiso temporal

**I:¿Hablamos del año 20 o 21?**

**E:**Del año 20, cuando empezaba recién la pandemia y habíae escasez de oxígeno. Y ese fue uno de los primeros este problemas. después el otro problema ya era un problema más este… operativo y desesperación de los

encargados,de las plantas que las plantas las querían para ayer entonces hubo, había una presión pero muy grande.

**I:En las regiones?**

**E:** Sí, que viajaba el padre ,me acuerdo el padre Barreto llamaba, mandaba una persona o venía el mismo alcalde ¿no es cierto? que había juntado dinero con la comunidad y así venían personas a… proque faltaba oxígeno ,no había y había mucha presión. Entonces a veces plantas se entregaban sin probarlas en el taller, entonces teníamos que ir al campo, armarlas y probarlas allá , terminar el proceso allá, y eso nos tomaba un tiempo más.

Y otro problema, que creo que bastante serio era que los lugares donde llevábamos la planta de oxígeno inclusive eran hospitales o centros de salud, o donde ellos indicaban,pero la mayoría eran hospitales eh… no había infraestructura para poder instalar la plata. Entonces, porque porque la planta necesita unos requisitos de estructura, un ambiente cerrado, ventilado, techado y una con energía electrica. Entonces ,tiene que ser energía eléctrica trifásica; y nosotros pedíamos o 220,380,440 la energia para poder instalarla, y nos decían que sí, que sí había, y cuando llegabamos no había techo, no había loza, no había nada.Y las plantas quedaban almacenadas, quedaban almacenadas por un buen tiempo. Entonces inicialmente inclusive yo me encargue justamente de eso , a las entidades que pedían una planta yo les mandaba unr requisito de infraestructura, hacía el plano, todo lo que debería de tener mínimo para poder instalar la planta, inclusive hasta las dimensiones mínimas del lugar donde deberían tener una planta o las razones mínimas que les explicaba, ventilado, lejos del agua, protegido de la lluvia y punto de energía, eléctrica. Y no era un punto de energía un par de alambres, tiene que venir un tablero de control o sea con todas las seguridades del caso,para evitar que funcione correctamente la planta y evitar que hubiere algún accidente producto de esta infraestructura.

**I:¿Cuántas plantas llegaron a instalar ustedes?**

**E:** Fabricaron cerca de 60 plantas

**I:¿Y todas gracias a (Ininteligible, MINUTO 11:27)?**

**E:** Todas

**I:¿En qué lugares del país? ¿Alguna zona más focalizada?**

**E:** Creo que en todas las regiones del país, menos …creo que en Puno fue una, una o dos.Después en Arequipa, Tarma, Chanchamayo eh… Iquitos, Iquitos creo que fueron cinco plantas, al mimo Iquitos¿no?, en todas partes, en la sierra, en la costa, en la selva

**I:En todas partes**

**E:**En todas partes

**I:Ok.De esas 60 plantas usted diría ¿ en qué lugares tuvieron mayores dificultades para la instalación?**

**E:**En todas

**I:En todas las regiones**

**E:** En todas las regiones, hasta el mismo Huaral,en Huaral, en Chancay también instalamos.

**I:¿Y todas fueron así, por cuestiones técnicas?**

**E:** Por cuestiones técnicas,a pesar de que les adelantabamos ,les pedíamos. Creo que hubieron algunas pequeñas excepciones. Creo que la de Tarma o la de Chanchamayo en la Merced , ese creo que sí…era ya un almacén que lo habían adecuado y ya tenía energía.

**I: Y eso cuánto significaba en tiempo, para que ¡Ok llegó la planta, no tenemos las condiciones hay que ver cómo se hace'**

**E:** Han habido casos en que han demorado entre… 30 días a 90 días.

**I:La planta en el establecimiento de salud sin funcionamiento**

**E:** Sin funcionamiento

**I: De acuerdo. Y otra cosa, a ver**

**E:**Y otra cosa más que, para que quieren la planta esa es la otra, o sea, para que quieren la planta. Para oxígeno, pero¿oxígeno para qué ? para llenar balones, para llenar balones o llenar un recipiente y tener conectado a las cama directamente, a las camas con paciente todavía no en situación grave, para eso o también para UCI. Eso es otra cosa,o sea ellos no lo tenían claro a veces; entonces llegaban y decían 'Hola quiero cargar balones' pero para cargar balones se tiene que… han debido de pedir un mantenimiento… se llama 'buster' en realidad pero es un compresor de alta presión para poder cargar los balones a 2000, 3000 libras de presión, y a veces eso no lo habían considerado. Entonces…y esa era otra dificultad también.

**I:¿Y eso era un desconocimiento de las jefaturas de los establecimientos o usted ve que eso era más a un nivel de MINSA de la gestión más propiamente de la institución como ministerio? Porque una cosa es el requerimiento del hospital y otra el requerimiento de Minsa como tal ¿no?**

**E:**Si.Sí claro, era de los dos. Porque a veces nos han pedido que en los hospitales que ellos coordinaban con que iba a comprar la planta y no les decían estos detalles. Yo mandaba, recuerdo mandabamos específicamente tiene que tener esa secuencia. Decían que sí, a veces decían que no, o que lo traigan y aquí solucionamos el problema pero el problema era bastante serio. Entonces eso atrasaba el uso de la instalación y poder producir el oxígeno. Y después ,ahí nos dimos cuenta que ningún hospital tiene plantas de oxígeno.Entonces todos estan supeditados a una logística básicamente de transporte

**I:Externo, de contratación externa**

**E:** Claro,logistica de transporte de qué, de balones,que van al hospital Central ya sea por ejemplo en Arequipa que tampoco tiene planta de oxígeno, sino que ahí a esa planta a ese hospital le entrega y le vende las compañías que venden oxígeno, les llevan ¿no? y les llenan el sistema que tienen ahí de tanques que tienen de alta presión y de ahí ellos llenan sus balones de oxígeno ,alimentan a la red del hospital y los otros hospitales vienen con sus camiones a recoger a recoger aceite en sus balones de oxígeno

**I: Más como un insumo**

**E:** Sí, eso creaba una dependencia increíble, una logística impresionante cuando ellos mismos podían tener la planta de oxígeno que no es nada del otro mundo. Son…para mí son equipos bastante bastante confiable una vez que estén funcionando y el mantenimiento no es caro, tampoco es un mantenimiento caro. Solo hay que tener cuidado nada más, y ese era el otro problema

**I:Hablando de ese tema, ¿cuánto puede costar una planta de oxígeno? de estas que se instalaron en los hospitales.**

**E:**Se fabricaron,nos autorizaron a fabricar plantas de 5 m3,de 10 m3 y de 20 m3. A ver si mal no recuerdo la de 5 creo que costaba en dólares… 200mil dólares o 180mil dólares incluyendo el 'Buster' que es un elemento bastante caro, la de 10 algo de 350 ,320 mil dólares; y la de 20 estaba alrededor de 400mil dólares aproximadamente. Con el 'Buster' incluido

**I: Y eso incluía el equipo mismo, la instalación, la capacitación, algún seguimiento posterior, mantenimiento o algo así o no**

**E:**A veces no incluía el mantenimiento posterior pero ofrecíamos el mantenimiento …

**I: On line**

**E:** No, no por 5 meses, 6 meses. En ese mantenimiento nuestro personal técnico iba y revisaba la planta cada 2 ,3 meses o cada cierta cantidad de horas. Muy pocos fueron los que aceptaron inicialmente

**I:¿Significaba un costo adicional o no?**

**E:**Sí, sí. Podía costar un costo adicional. A veces nos decían ‘ no quiero que en el precio me pongas el mantenimiento. De 400 mil le cobrabamos 410 mil. Eso incluía cambio de filtros ajustes regulaciones y chequeos. Pero en realidad el mantenimiento de las plantas son muy simple. Y ahí nos dimos cuenta ,como te dije, que los hospitales no tenían ,ni uno, ni uno tenía una planta de oxígeno, ni una.

**I: Hasta ahora me habló de la parte técnica, de la infraestructura. Pero que tal el recurso humano, el personal capacitado para el manejo…**

**E:** Ese es el otro problema, que no había personas capacitadas. Y había mucha rotación también en los empleados mismos de los lugares mismos donde dejábamos las plantas. Porque al final las plantas no se las entregabamos al hospitallas entregabamos al hospital , pero se las entregábamos al dueño; que era a quien compraba. Que era la parroquia…

**I:Episcopado, cáritas**

**E:**Episcopal, cáritas o no sé quién , que eran los que pagaban. Salvo cuando empezaron a comprar las municipalidades, salvo cuando empezaron a comprar las municipalidades con recursos de ellos y recursos de gente que había donado dinero,las personas que donaban donaban dinero eso era. Pero no había suficiente personal capacitado para operar las plantas

**I:Y ustedes incluyeron dentro de la venta del producto las capacitaciones al personal**

**E:**Sí, generamos capacitación, pero cuando íbamos rotaban a la gente o habían problemas, por mal funcionamiento porque cometían errores, la dejaban prendida, no la apagaban,no purgaban, habían cosas que hacer, un proceso que se quedaban también por escrito ¿no? Entonces también un problema en el personal

**I:¿Qué tan común ,frecuente fue de que el personal no estuviera capacitado? diría usted que fue el 100% de casos, 80**

**E:**Un 80,aprox

**I:Tuvieron dificultades una vez que se instalaron como con cuantas plantas ?**

**E:** En realidad no teníamos problemas con ninguno.Lo que sí, queremos ser honestos, en las primeras plantas producto del diseño original hubieron algunas este… mejoras. Que eso fue hecha por la compañía sin costo

para ninguno de los clientes, a veces no entregaba la pureza. Entonces tuvimos que llevar y cambiarle la ‘Ceolita’por otro tipo de ‘ceolita’ mejor calidad para entregar la pureza

**I:Porque era la primera vez que se producía**

**E:**Claro, y después , habían también al comienzo había problemas de fisuras con los tanques,que eran de acero inoxidable y habían fisuras. Y nosotros no es que llevabamos un soldador, les llevabamos un tanque y lo reemplazamos y nos traíamos el otro. Estos son problemas operativos nuestros, que fue al comienzo ,después ya pudimos mejorar esos procesos y las plantas funcionaron perfectamente, perfectamente.

**I:Entiendo que ustedes, dentro de su contrato no sé si está, hacer una supervisión, evaluación de cuál es el funcinamiento de las plantas de oxígeno como para saber si están funcionando ahora o no están ¿le han hecho algún tipo de seguimiento?**

**E:** Sí, justamente desde el año pasado ,desde el año pasado que ya se desapareció el covid. Todas las entidades que tenían estas plantas nos han estado llamando para que le hagamos mantenimiento porque estuvieron paradas varios meses¿no? eso a sido el año pasado. Hemos ido a hacerles mantenimiento, a hacerle el arranque o las máquinas que no arrancaban hemos ido y hemos hecho arrancar las plantas y entregar el oxígeno que ellos solicitaban.

**I:Y estas dificultades en los equipos era porque ya no necesitaban el oxígeno o porque de pronto se malogró y no le hicieron más mantenimiento**

**E:**Mantenimiento, se quedaron sin operación, sin mantenimiento se quedaron paradas, paradas. Hemos vuelto a ir, hemos hecho creo que de las 60 por lo menos a 30, la mitad le hemos hecho mantenimiento. Inclusive aquí en Lima que se vendieron a varios; a la Municipalidad de Lima que se vendieron 2, distrito de San Isidro, San Miguel ,4 y 2 más; creo que aca en Lima habían como 5-6 plantas que estan funcionando perfectamente. Y todas se activaron a raíz del dengue.

**I:A raíz del dengue se han vuelto a activar**

**E**: Sí, comenzaron a. El año pasado algunas querían hacer mantenimiento, ya tenían presupuestos seguramente ya se había hecho la transferencia formal de que lo compraron al hospital, ya imagino ellos lo incluyeron en el presupuesto y comenzaron a hacerle mantenimiento a las plantas .Unos llamaban para que arranquemos la planta y le enseñemos a las personas que estaban a cargo el funcionamiento de la planta y verificar el funcionamiento de oxígeno que ellos requieren

**I:¿Como de cuántas plantas estamos hablando que ustedes han empezado a hacer el mantenimiento?**

**E:**Han sido como 30

**I:A ya , no a todas**

**E:** No a todas, como a 30 que hemos echo el mantenimiento, y gente nueva que no conocía el manejo, el concepto para cómo funciona qué es lo que hay que hacerle. Y bueno ahí aprovechamos, qué es lo que quieres hacer con la planta; quieres llenar balones, quieres tenerla en la linea de la red de las camas de los hospitales o los dos.

**I:Ahora ellos tienen más claridad?**

**E:**Sí, ahora ellos tienen más claridad. Ya funcionan las plantas sin ningún problema

**I:qué han optado por hacer? que les han pedido , que funcionen para balones o para la red**

**E:** Para las dos, ahora, hay algunas que no tenían el 'Buster' para balones y nosotros creo que les hemos vendido, han sido un par de casos. Pero casi la mayoría se les ha ido entregando con 'buster' para que puedan cargar balones. Porque eso es justamente lo que los hospitales debería de tener, dependiendo la capacidad deberían de tener dos plantas: dos de 5, dos de 10 o dos de 40,dos de 20 m3, dos en paralelo, dos en línea.Que las dos juntas entregan 40, las dos juntas entregan 20 y las dos de cinco entregan 10m3 en paralelo. Entonces, así puedan ellos tener con su 'buster' pueden tener los balones llenos y los pueden poner en serie y pueden también ser el pulmón para alimentar las camas o llevarlos al mismo cuarto o poderlas llevar a un centro médico cerca¿no?transportarla y llevarla

**I: ¿Y eso están optando por hacer los establecimientos?**

**E:**Eso le hemos recomendado, pero no no lo han hecho

**I:Qué prefieren, qué estan optando ?**

**E:** No han dicho nada, pero eso les dijimos desde hace mucho, desde la pandemia. Tan útil es tener dos.

**I: ¿Y el MINSA interviene?**

**E:** Pero por qué dos, las dos tienen los mismos componentes, entonces en caso de emergencia si una está en mantenimiento pero tengo una funcionando,a la mitad pero funcionando, tengo oxígeno.Si tuviera una sola o un solo sistema de 40, no es cierto, se malogra ,falla, se para y no tengo oxígeno para nadie. En cambio si tengo dos en paralelo me facilita el mantenimiento; mientras una este operando a la otra le puedo hacer mantenimiento .Si una se daña, se daña algo tengo la otra que por lo menos tengo oxígeno

**I:Y a nivel del MINSA usted ha notado algún  cambio, algún interés adicional**

**E:** Nada, nada. Absolutamente nada

**I: Y en la parte normativa usted me dijo que le dieron de manera provisional la autorización para la producción. ¿Qué pasó después? ¿hubo algún cambio?¿ajustaron la norma?**

**E:**No, fue provisional

**I:¿Por cuánto tiempo fue provisional digamos?**

**E:** Fue hasta… hasta el tiempo que, que duró la emergencia,el año pasado

**I:Hasta noviembre**

**E:** Noviembre

**I:Hasta Octubre¿no?, porque Noviembre 1 ya termino. O sea ¿ustedes en este momento no pueden producir más?**

**Entrevistado:** No, no podemos producir. Solamente hacemos mantenimiento.Creo que están gestionando para que le den el…

**I:La autorización**

**E:**La autorización ,ya…permanente

**I:Ustedes tienen infraestructura ya establecida para la producción**

**E:**Sí, sí.Todo

**I:Esta empresa de la que usted me hablo que se constituyó  para esta situación ¿todavía está en actividad?**

**E:** Sí ,sí está en actividad; hace otras cosas también ¿no?. Ahora están haciendo proyectos para retirar los metales pesados del agua, el cambio

**I: Siempre con la Universidad Católica**

**E:** No, ya no ya. Este proyecto ya no.

**I:Pero el tema del oxígeno sí. ¿Se mantiene?**

**E:**El tema del oxígeno sí se mantuvo hasta este año, hasta fin de año.

**I:Ahora ustedes trabajan de manera independiente como empresarios**

**E:**Sí,exacto, exacto y obviamente la Universidad Católica con la llegada que tenía a los ministros, los ministerios hacía todas las recomendaciones que yo te he mencionado¿no? que deben tener dos en paralelo, que deben tener infraestructura, que deben tener personal capacitado, no depender de solo un proveedor de oxígeno.

**I:Si en este momento hubiera alguna necesidad de producción de plantas de oxigeno, ¿recurrirán a ustedes como empresa o a la Universidad Católica ?**

**E:**A nosotros

**I:A ustedes**

**E:**Sí, de repente pueden acudir a la Católica, pero la Católica nos va a llamar a nosotros.

**I: Ustedes tienen la infraestructura, todo el equipamiento, toda la parte…**

**E:** Todo,los planos, el diseño.

**I: Esta cuestión normativa entonces, además de esa dificultad del porcentaje, los filtros ,la calidad. ¿Hubo alguna otra condición, requerimiento adicional o era básicamente eso ?**

**E:** Básicamente era eso, no, no había. Que recuerde, que recuerde…no había otra. Pero solo podíamos producir de 5, de 10 y de 20

**I: Las empresas, entiendo que hay dos empresas importantes que producen plantas y también tienen todo el tema del oxígeno,de balones y todo esto. Ellas producen plantas más grandes?**

**E:** No, no

**I:Todas hasta 20**

**E:**Claro, estoy hablando de oxígeno industrial. No era una producción industrial como la hace Linde. Ellos son, producen, tienen la planta produce, no sé , 500 creo m3 diarios. Esos son impresionantes, tienen otro sistema de frío, alta presión ;eso otra ,otra cosa. Muy grande,para producir grandes cantidades de oxígeno industrial que va al 98% al 99% que se usa en la industria para sobretodo para soldadura o para hornos ,la industria en general. Y el medicinal que tiene otras características,que tiene una pureza no mayor a 93% de oxígeno. Entre 91 y 93% de pureza de oxígeno

**I:En ese entonces cuando hubo el tema de…digamos la crisis propiamente del oxígeno netamente,porque no fue durante toda la pandemia al inicio no surgio, fue más adelante**

**E:** La primera ola, la segunda, la tercera ola. Era terrible

**I:Sí, exacto. En ese momento digamos, solo estas dos empresas estaban como…tenían las autorizaciones para producir, vender, distribuir en fin. Usted , no sé si sea el caso, usted ha percibido que haya habido tal vez algún tema de interés económico, detrás de estas dificultades o barreras que se puso para la producción de plantas en el país**

**E:**Seguro, porque habían 2 empresas que importaban equipos, para…equipos de producción de oxígeno medicinal¿no?. No sé si ellos, pusieron trabas para que no se fabrique normalmente ,sino se importe. Entendiendo que una planta importada, ya sea de México, Estados Unidos similar a la nuestra vala el doble ,dos millones de dólares, millón y medio, de dólares una planta y entregaba lo mismo. Salvo la de China, que costaban igual que nosotros. Nos traían desde China

**I:Pero en ese momento no se podía traer**

**E:**Era imposible, trajimos una. Por una emergencia, trajimos una planta de China , una sola planta trajimos que también se demoró como 90 días en llegar, pero 'llegó rápido' ,entre comillas,pero llegó. Y fue la única¿no?. Y depués el tema de los insumos, la parte de limpieza nunca tuvimos problemas de conseguir para conseguir

**I:Todo se hizo acá**

**E:**Se ensambló acá , acá lo unico que se hacía eran los tanques , de acero inoxidable. El resto era traído de afuera o comprado normalmente ,los tableros, las valvulas

**I:Pero en ese rato eran difícil… los tralsados, todo se paralizó, se cerró**

**E:**Sí, todo era súper complicado. En esa época era súper cumplicado

**I:Si usted tendría que sugerir algún cambio en la normativa, un cambio en la política como para que esta dificultad que ustedes tuvieron. ¿Qué sugeriría?**

**E:**Bueno sugeriría que… un poco de flexibilidad en las normas, en las normas. Y comprobar con una planta piloto los valores que son adecuados para el uso medicinal, porque eso a las finales no comprobaron. Nosotros lo comprobamos en la línea; con la Universidad Católica que ellos tenían también los instrumentos especiales para comprobar que además de la pureza del oxígeno, el oxígeno no vaya con otros elementos nocivos a la salud, para poder veificar que es un oxígeno medicinal

**I: Ok, ok. ¿Cómo ve usted el trabajo de esta dirección del MINSA? así en general en relación al tema del oxígeno medicinal, de la crisis de oxígeno medicinal que se tuvo en el país**

**En:** Muy burocrático y muy lentos los procesos. Muy, muy lentos; no se le ve involucramiento en la emergencia. Porque si hay un interés se tienen que involucrar. Porque si hay una planta, o una compañía que está fabricando, no hay que esperar que los busquen ,los llamen para que vayan. Ellos deberían tomar iniciativa de ir a ver y cómo ayudar a que esta iniciativa, este proyecto seguir adelante para dar una solución a los ciudadanos¿no?. Eso era,no había involucramiento.

**I: ¿Y a qué cree que se debe eso? Usted que estuvo en contacto con ellos**

**E:** Sí, no era… es creo que…no sé si es cultural en el sector público. Sobre todo los de carrera, no los que llegan a cargos este…

**I:Directivos**

**E:**Directivos de confianza ,sino a los de carrera¿no? No son gente comprometida con lo que tienen que hacer, no se involuran al final, no ponen ellos 'está mal esto, hay que hacer esto, ¿no? No ,está mal', hagan esto , modifiquen su proceso, su ficha técnica hay que corregirla, hay que hacerla así o asá. Si no es,iban sí o no, mal o bueno; no había un asesoramiento proactivo para tener una mejora continua, permanente. Eso era lo que llamaba la aención de estos organismos, que no no… más son fiscalizadores,van 'ta malo y te condenan defrente; no participan del problema. Como debe ser, el espíritu de todo. Yo le digo porque he trabajado en sector privado y también tenía clientes ,no es cierto, clientes sumamente exigentes que yo les vendía las máquinas ;las máquinas tenían problemas ,claro, al final era responsabilidad del que le vendió y ellos se involucraban también; es tu problema ,también es el mío vamos a solucionarlo juntos no vamos a ver qué ha pasado

**I:Y a qué eso ¿es más de cultura o una cuestión de temor a la también a la responsabilidad?**

**E:** Cultura, una educación también enfocada en valores quizá ¿no?, compromisos trabajo en equipo eso creo que va más por ahí que por otra cosa

**I: ¿Y eso usted lo percibe a nivel de gestores del minsa nada más o también a nivel más regional, municipio o gobiernos regionales?**

**E:** Sí

**I: Ha sido igual para usted o digamos …**

**E:** Sí,claro. Más son los del sector salud, las municipalidades son un poco más este… abiertos algunos, más receptivos, más comunicativos o…unos más que otros pero creo que ayudaban mejor.Pero los del sector salud o las direcciones de los ministerios son demasiado parametrados y duros ¿no? hay mucha falta de involucramiento y los temas que ellos también les interesa. Yo veía eso, a mí eso sí me disgustaba

**I: Y luego de que ustedes han hecho o han instalado las plantas, entiendo que ustedes como empresa no lo vendían directamente al sector salud sino a las instituciones u organizaciones qué…**

**E:** religiosas o algunas municipalidades que compraban y ellos después se las han transferido al…

**I: Los establecimientos de salud**

**E:** Establecimiento de salud

**I: Pero ustedes tenían algún tipo de ah… no sé si el término es fiscalización, de seguimiento o verificación por parte del MINSA**

**E:** ¿A nosotros?

**I: a ustedes como empresa que vende este servicio, no me refiero al trabajo que ustedes hacen sino a los a los equipos**

**E:** Mmmmm, no

**I: Las exigencias que ellos tenían, ¿alguna vez fueron a verificar si realmente correspondía el 93% de pureza?**

**E:**Sí,una vez fueron …y creo que porque en la ficha técnica no decía no sé qué cosa ,estaba mal…

**I: Quiero entender eso mejor, a ver a ustedes les observaban el documento que usted me decía las normas chiquita que es ustedes les exigían 93**

**E:** Nos pedían una ficha técnica y ya habíamos entregado la ficha técnica.A veces esa máquina ya la habíamos cambiado, mejorado en algo que no había sido modificada en la ficha técnica. Entonces eso es una mejora o sea está bien no es igual pero esto de acá es mejor.. OK pero no era nada que no dejaba de funcionar era una válvula… así en la válvula en bola por una válvula con puerta o porque el filtro le pusimos un filtro adicional o le quitamos un filtro y le pusimos un filtro más grande. Pero al final era la misma función

**I: Yo me refería a que por ejemplo,a ustedes les pidieron que las plantas de oxígeno tuvieran , produjeran el oxígeno al 93% .OK, luego ya en campo el minsa a través de alguna dirección,algun personal, que se yo verificó que suceda así**

**E:** Sí, sí en realidad no sé si el minsa pero los usuarios eran los que verificaban porque que la planta tiene…

**I: Estas exigencias**

**E:** No. Tienen el tablero dónde indica la cantidad de oxígeno, también la presión hay dos, dos ,este… el doble ,o sea lo ves acá y lo ves acá y también se puede llevar a un un instrumento y medirlo al final de la línea ¿no? o la línea que ellos quieran el porcentaje de oxígeno. También se podía hacer eso y en eso eran rigurosos todos , todos son rigurosos y todos exigían que esté en ese rango de entrega de oxígeno ;eso sí eran los usuarios básicamente

**I: Entiendo que ustedes han trabajado de manera más privada con, digamos… las negociaciones que han tenido más han sido con organizaciones que han colectado a los fondos o gobiernos regionales pero usted habrá percibido en algún momento que ha habido algún interés político, de alguna personalidad ,de algún actor para empujar que esto de las plantas funcionen o tal vez que no funcionen**

**E:** Los que estuvieron más preocupados fueron los… todas las entidades episcopales ,ellos fueron los que empujaron para que esto sea un éxito, a través de la Católica. Y la Católica es quién que ayudaba mucho y ellos algunas veces hablaban con el ministerio de salud básicamente o con primer ministro para ayudar a que esto… este proyecto sea mucho más… avance mucho más rápido y que las entidades que necesitaban plantas de oxígeno estábamos ahí dispuestos a entregarselas. Más ha sido por ese lado, más ha sido por ese lado

**I:** Ningún digamos actor, congresista ,ministro, autoridad, NN

**E:** Bueno aunque hubo un…creo que congresista , ahora es congresista. En esa época era gerente general creo ,de la municipalidad de Huaral ,ahora es congresista ,fue ministro de Castillo; ah no sé cómo se apellida. Él fue el que cuando no era autoridad electa ayudó en este tema

**I: Ayudó cuando ustedes tenían que instalar**

**E:**Sí

**-Entró una llamada y se pausó la grabación-**

**I: A ver, si usted tuviera que resumir ¿cuál fue el mayor obstáculo que tuvieron para la producción?**

**E:** ¿Y la puesta en operación?

**I: así es y la puesta en operación de las plantas**

**E:** La falta de infraestructura

**I: Infraestructura**

**E:** Eso. Infraestructura, energía eléctrica e infraestructura del local.

**I: Espacio, digamos. Ahora que ustedes han empezado hacer mantenimiento cómo está el tema de la infraestructura**

**E:** Todos han tenido que adecuarse a las infraestructuras que nosotros les recomendamos

**I: ¿Ya la tienen?**

**E:** Ya la tienen.

**I: Todos**

**E:**Todos. Han puesto energía ,han puesto el techo , las han puesto en lugares cerrados, ventilados ¿no? protegidos del ambiente y todo.

**I: ¿Cuál considera usted que ha sido el aprendizaje de las personas que ,digamos en ese momento vieron el tema del oxígeno medicinal? no sé si se ha vuelto a contactar con ellos, se ha vuelto a encontrar o han rotado todo si no están más. ¿O en el proceso usted vio que tuvieran algún aprendizaje?**

**E:** yo creo que aprendizaje…

**I: De ellos**

**E:** De ellos sí… que creo que han podido detectar que es un tema simple ¿no? el tema de las plantas de oxígeno no son complicados ,con una…un pequeño entrenamiento… claro la persona tiene que tener un nivel educación ,no digo superior pero sí técnico ¿no?

**I: Conocimientos básicos**

**E:** Conocimientos básicos ,con eso es suficiente para que uno le puede explicar y puede entender cómo funciona, que hay que hacer y qué es lo que no hay que hacer. A las finales prender y apagar y ver que esté el oxígeno ,lo entreguen y si no ‘ por eso ah, por eso por eso por voy reviso’ y los filtros que estén correctamente, no esten saturados ,hay una línea roja que se prende, en el tablero también hay señales que ayudan a eso ¿no? A identificar cualquier falla. Creo que eso es un dado cuenta que no es nada difícil.

**I: Si tuviéramos una pandemia en la que tuviéramos nuevamente la necesidad de oxígeno, como la que tuvimos ,igual o peor¿cuál sería la situación en este momento?¿Podríamos afrontar una situación similar o no estamos en la capacidad de hacerlo?**

**E:** Nosotros creo que sí estamos en la capacidad de hacerlo. Tenemos el ‘know how’, tenemos el conocimiento y todo para poder este…ensamblar plantas y entregarlas, tal cual hemos querido, hemos visto la necesidad que hay

**I: Ustedes como empresa ,pero ¿y el país? porque ya hay plantas**

**E:** Sí, sí hay bastantes.No solamente vendidas por nosotros

**I: Sí ,sí lo sé**

**E:** Otras que les han donado han traído de afuera. Inclusive hemos hecho mantenimiento a las plantas que nosotros no hemos vendido, eso también hemos hecho,creo que más de 8 plantas creo que no las hemos vendido. Hemos hecho mantenimiento y las hemos puesto en funcionamiento acá adentro porque no funcionaban, tenían problemas. Incluso arrancar ¿no?y lo hemos hecho eso.

**I: Con todo lo que hay instalado las condiciones en las que están ahora podríamos afrontar una situación así**

**E:** Yo creo que sí ,sí ,yo creo que sí .Por lo menos inicialmente. Inicialmente ya no va a estar desbordado el sistema sanitario de oxígeno ,ya no va a estar desbordado pero sí se puede aliar y dependiendo de eso uno puede ir incrementando plantas de oxígeno para evitar esto

**I: Desde su mirada como empresario ,como actor que estuvo involucrado en todo el proceso de las plantas cuáles son las lecciones clave que usted diría son aprendizajes**

**E:** Que todos los hospitales deben tener plantas de oxígeno. Todos los hospitales, plantas de oxígeno y 2 en paralelo. Calcular la capacidad máxima de porcentaje y poner 2 de ellas

**I: A mí me queda la inquietud …eso es digamos cuando hay una necesidad de oxígeno …digamos constante ¿no? pero sabemos que…**

**E:** Hay, la hay constante.Esa fue otra sugerencia que hicimos, '¿qué vamos a hacer con el oxígeno ahora que pase la pandemia?', asilos de ancianos, todos los asilos ancianos .La mitad de las personas necesitan oxígeno o tienen deficiencia ,y ahí es un lugar Donde uno pueda volver a entregar

**I: Intervenir**

**E:** A intervenir

**I: Pero eso entraría a tallar cortando la compra a estas empresas ,que son importantes ,que son las que proveen ahora.¿Como ve eso usted?**

**E:** Mmmm…no le entendí esa parte

**I: O sea en este momento hay muchas instituciones que dependen o compran a estas 2 empresas el oxígeno**

**E**: Sí correcto

**I: Si nosotros empezamos ,pienso , en que un establecimiento de salud empieza a proveer de Oxígeno digamos ,a los asilos como usted lo dice eso indicaría que ya no se compraría oxígeno a estas empresas ¿como usted ve eso?**

**E:** Yo creo que ahí tienen que ver también el costo beneficio. Cuál es la Inversión y el beneficio que voy a tener,y cuánto me puede costar a mí ese oxígeno ¿no es cierto? el metro cúbico de oxígeno versus lo que me puede vender la planta, estos distribuidores, estos fabricantes, vería eso primero. Y lo segundo que está ya por encima de lo que pueda costar es la salud y asegurar que los pacientes tengan oxígeno ¿no? entonces qué… voy a Depender estas personas sí-no o puedo usar las 2. Tanto la…Una parte le compro a ellos para asegurar los tanques e infraestructura que ya tengo montados y mis plantas que tengo instaladas puedo llenar balones de oxígeno y entregarlas a los cuartos cuando no tengan el sistema dentro o llevarlas a otros establecimientos de menor categoría que no tienen plantas de oxígeno. Eso podría ser, Un poco combinar ambas.No dejar de lado

**I:** Y**o no tengo más preguntas ahora pero no sé si usted quiere comentar algo más que es importante que sepamos para el estudio, información ,alguna pregunta que yo no he hecho que usted diga 'tengo que decir esto'**

**E:** No, creo que ya todo lo …creo haberlo dicho todo.En la experiencia que tuvimos, con el tema del oxígeno que fue bastante crítico .Al margen ,obviamente …no solamente creo que… no solamente hay que depender de las plantas oxígeno que nosotros proveíamos,ni los proveedores fabricantes de oxigeno que venden. Pero también hay pequeños este… pequeños equipos que producen oxígeno

**I: Así es**

**E:** Que son duales …y producen cierta cantidad para la…

**I: Para las personas que han quedado afectadas por ejemplo¿no?**

**E:** Afectadas,puede tener su aparato con energía eléctrica y tienen… pueden tener también… este…

**I: Ustedes producen esos respiradores**

**E:** Sí ,bueno les llaman el respiradores. Sí, trajimos unos cuantos

**I: Ya, pero ¿no los producen? ¿no los elaboran?**

**E:** No,no, no. Eso la mayoría son chinos o europeos¿no?. Son unos respiradores pequeños. El único problema ,son que tienen mucho ruido, hacen mucho ruido; sobre todo en las noches que baja un poco el ruido

**I: El ruido de la ciudad**

**E:** El ruido de la ciudad ,eso se escucha terriblemente. Entonces hay que alejarlo un poco de los pacientes y creo que es bastante molesto ¿no? es solamente para una emergencia seguramente.

**I: Bien,ok. Gracias**

# 005 Entrevista

Meeting started: 27/6/2023, 11 AM

Participantes: E, I

I: Investigador

E: Entrevistado

**I: bueno vamos a comenzar .Y para tener de manera más o menos contexto con quien estamos conversando, sin necesidad de mencionar su nombre podría, por favor comentarnos su labor ¿Cuál es su actividad?¿Hace cuánto tiempo está usted trabajando en este tema? y su vínculo en tema concreto del oxígeno medicinal**

**E:** Mire yo soy médico, médico cirujano tengo 2 especialidades soy médico internista y también soy médico ginecobstetra.Trabajo nombrado para el ministerio de salud pero en diversas ocasiones he ocupado cargos de asesoría en políticas públicas en el congreso de la república. He sido varias veces asesor principal de la comisión de salud del congreso de la república y he trabajado con varios partidos políticos los temas representativos a la salud pública. Además he liderado el colegio médico en algunas…2 ,3 oportunidades y también dirigido una institución denominada xxxxx que es una institución médica netamente de estudios estudios de lo que… es desarrollo de la propia profesión médica.

**I: De acuerdo**

**E:** El tema vinculado al oxígeno se basa en mis estudios que hago sobre salud pública en personales. Yo no he seguido el oxígeno medicinal sólo a partir de esta pandemia,sino que yo venía desde atrás estudiando el tema del oxígeno medicinal inclusive desde el año 2003 o 2004 cuando ya hubo unas denuncias por oligopolio y concertación de precios ,y distribución del territorio nacional en dos grandes empresas; en el norte una y otra en el sur para la venta del oxígeno medicinal para Essalud salud. Ahí inclusive hubo una participación de Indecopi donde la primera instancia Indecopi, señala una sanción a estas empresas pero curiosamente,posteriormente estas acciones son levantadas. Entonces, desde ahí ya se observó en paralelo otra acción que también yo he señalado en mis estudios de esa época ,era que en modo sistemático todos los cables del Perú son públicos, iban perdiendo progresivamente perdiendo capacidades de producción de su propio. Teníamos plantas de oxígeno propias que tenían los hospitales que ya nunca se reparaba, se dañaba y no se reparaban y siempre era más fácil comprar los balones de oxígeno o sino comprar los tanques… los iso tanques para poder almacenar lo que compraban de estas grandes empresas.

Progresivamente, el tema llevó a que el año 2010 a raíz precisamente del conflicto que había en Essalud …y había esa controversia en torno a si era o no, se había originado o no oligopolio ;es que el ministerio de salud emitie una resolución ministerial ,cuando estaba de ministro Óscar Ugarte,en el gobierno del presidente Alan García. Sacó una resolución ministerial que señala que el oxígeno medicinal para fines del petitorio nacional de medicamentos sea un oxígeno que tiene una concentración al 99% . Esto permite precisamente ,también, que como colateral que el proceso ,distorcione el proceso que viene indecopi, en cuanto a estas empresas, porque de alguna manera ya el ministerio les había dicho que ese es el oxigeno que se necesita y ya no había forma de ver el tema del oxígeno superior al 96% de concentración. Luego los sucesivos cambios en el petitorio nacional que ha habido hasta la época de la pandemia se mantenían el oxígeno medicinal al 99% y ese era el oxigeno que se

utilizaba en el país. Esto que llevo también, qué se desincentive el mercado de oxígeno en el país o sea no había forma de producir 93% oxígeno medicinal porque nadie te lo compraba ,entonces el sector privado no tuvo señales en el mercado para invertir en este sector ,tampoco la producción de concentradores y menos aún había intenciones de que los hospitales puedan invertir ,porque ya invertir en un oxígeno medicinal al 99% ,las inversiones eran mayores.

**I: Así es**

**E:** Entonces, inicialmente hubo un desmantelamiento y después se le quita el incentivo para participar en este en este tema. Esto era grave porque había problemas sobre todo en los servicios de neonatología, donde se requería oxígeno y como todos sabemos nosotros el oxígeno al 99% usado en recién nacidos produce la … produce la retinopatía que lleve a la… a que los niños se vuelvan ciegos. Entonces había que disminuir la cantidad de concentración de oxígeno que comprabas al 99% para bajarla, digamos para disminuir esto.Y es donde más se usa el oxígeno, uno de los lugares donde más se usa el oxígeno son los lugares, las salas de parto

**I: Salas pediátricas y…**

**E:** tendría que colocar lo que llaman campanas... Que para poder disminuir el oxígeno de 99% al nivel de oxígeno que necesitaban estas… puedes parar un ratito voy a responder esta llamada.

-Se pausó grabación-

**E:** Cuando todo ese tema de cómo se fue destruyendo prácticamente la producción de oxígeno en los hospital públicos,…los directores de hospitales,los funcionarios y presupuestos del sector salud iba a la compra ya del oxígeno ya en balones o también a la compra de los isotanques para ya comprar a estas empresas grandes

**I: Que eran dos**

**E:** Dos, dos únicas empresas

**I: ¿Y ellas tenían ese nivel de concentración 99%?**

**E:** Solamente eran las únicas que producían el 99%

**I: Ok**

**E:** Entonces ,qué ha pasado entonces, que a partir de esa fecha… estas empresas como señala Indecopi, se habían especializado, unas para el norte y otras para el sur del país. Entonces si tú querías comprar oxígeno en el sur era una empresa y Essalud tenía ese problema porque Essalud tenía que saber a quién compraba en el norte, a quién compraba en el sur.

**I: No puede ser una compra masiva a nivel nacional**

**E:** No puede ser una compra masiva a nivel nacional. Entonces esos temas llevaron a Indecopi a a ver toda esta discusión. El otro problema que había es que no había a nivel nacional una posibilidad de saber qué hospitales tenían o no operativo el oxígeno, no había una notificación obligatoria como cualquier medicamento a pesar de haber estado en el PENUME ,a pesar de estar en el petitorio nacional único de medicamentos esenciales ,era el único medicamento que nadie informaba cómo estaba tu stock.

**I: Nadie reportaba.OK**

**Entrevistado:**Si puedes saber cómo va el estado de una penicilina pero el oxígeno medicinal no había, entonces no había un sistema de notificación nacional ,por tanto en el momento en que ocurre la pandemia no se sabía nuestra capacidad de respuesta en oxígeno frente a las necesidades de la pandemia.

**I: Así es. Se reportaba la necesidad…**

**E:** Pero no podía cuantificarse ,ni nada de eso. Por otro lado también hay que tomar en cuenta que estas grandes empresas… te daban todo el servicio, te llevaban el oxígeno, te lo dejaban en el hospital, te recargaban tu balón o sea no había ninguna necesidad de que los hospitales pudieran crear un servicio propio para hacer eso. Entonces cuando llega la pandemia,las empresas, estas dos empresas ven sobrepasada su capacidad de producción de oxígeno a tal punto que ellas no podían cubrir la demanda nacional. Y entonces ahí lejos de… crear, abrir el mercado para que participen otras empresas;lo qué hace el gobierno, de Martín Vizcarra en ese momento, es insistir en la compra de balones, comprar del extranjero, comprar de otros países ¿no? Porque se acuerda cuando hay la polémica del oxígeno que podían dar las empresas mineras, etcétera, no solamente era un tema de concentración, sino también de pureza, o sea, de que no tenga contaminantes digamos eso que es distinto, pero también era en el fondo una forma de permitir que ingrese un oxígeno menor a 99%. Entonces se da todo un debate normativo y yo participo en ese momento, con una congresista , con la doctora Tania Rodas; yo le elabore un proyecto de ley para disminuir la concentración de oxígeno al 93%. Eso solo eso no era esencial , para nosotros era esencial crear el sistema de suministro, el tema de los balones también es otro problema,el tema de los manómetros también es otro mercado, son mercados que van ligados al oxígeno

**I: Y que son muy especializados**

**E:** Son especializados entonces la ley, señala no sé si ustedes han tenido tiempo de revisar la ley, es una ley que me tocó a mí (Ininteligible, MINUTO 10:01) , toda la problemática del oxígeno… tuvimos tuvimos la opción de reunirnos con… el colegio de ingenieros para ver el tema de…

**I: la parte técnica**

**E:** La parte técnica, nos reunimos con las dos empresas ,nos reunimos con ellos, nos reunimos con directores de hospitales ¿no? y además vimos la parte normativa … y entonces se hizo el proyecto de ley días antes de que el proyecto de ley, ya cuando se había aprobado el proyecto de ley para remitir al ejecutivo, el ejecutivo emite un decreto de urgencia 062 que en su artículo segundo dice que el artículo baja de 99% al 93%. Pero quiero

advertirte que ese no es el tema esencial, es importante ,es digamos la base pero no es lo esencial ;lo otro es lo esencial, crear el sistema porque si tú no creas un sistema de suministros, no creas un sistema de control de calidad del oxígeno,y no creas un control de ver los stock de suministro ,el informe de la cantidad y la obligatoriedad

**I: No se llega a monitorear**

**E:** Y de la obligatoriedad de que cada hospital tenga un tema de seguridad en oxígeno ,etcétera ,y cada centro de salud eso no… no iba a funcionar. Entonces nuestra ley es compleja, a tal punto fue tanto el debate interno , y ahí obviamente se vieron los intereses económicos que tuvimos 3 posiciones, 3 alternativas: la del presidente Vizcarra que solo bajaba del 99 al 93, la del partido Alianza Para el Progreso que señalaba en ese momento una propuesta de ley que hizo el congresista Valdez ,qué señalaba que había que declarar de interés comprar oxígeno nacional e internacional ,y la otra propuesta que también hizo una congresista de Alianza Para el Progreso que es ,con la cual yo había trabajado, que era crear todo un sistema de suministros. Que finalmente, o sea hay 3 normas, 3 normas hay en ese periodo sobre el tema del oxígeno. Pero finalmente recién es en diciembre de ese año del 2020 es que se puede introducir ya el oxígeno al 93%

**I: Una pausa en ese momento. Usted comenta que alianza para el progreso tenía como 2 propuestas ¿cómo se explica que un mismo grupo parlamentario o un mismo partido haya 2 propuestas distintas¿no? Una para la compra…**

**E:** El asesor, el asesor era yo que estaba detrás. Cuando yo vi que el proyecto de ley había presentado el congresista Valdez con Alianza Para el Progreso ,yo le dije que han hecho un mal proyecto, y entonces la congresista que estaba ahí atenta me dijo 'doctor y cuál es su proyecto', esto es el proyecto. Entonces me dijo 'lo hago, lo asumo'

**I: Ya ella de manera individual**

**E:** De manera individual

**I: No como partido**

**E:** Individual.No, Finalmente como partido, porque tienen que firmar la bancada pues

**I:** Ya, ya, ya

**E:** Entonces ahí tenía 2 proyectos. Que al fondo, en el fondo uno era mantener las cosas, declarar de interés la compra ,que era solamente para asignar fondos, para seguir haciendo lo mismo. Yo pedí un cambio total del sistema

**I: ¿Y ahí más allá de una cuestión partidaria habría habido algún… trasfondo económico de algún interés económico del congresista?**

**E**: Bueno la política en general se mueve en base intereses

**I: Lo sé, lo sé**

**E:** No es extraño que hayan en un mismo partido político 2, 3 proyectos de ley distintos porque hay conflictos siempre de diversos intereses sobre todo

**I: ¿Y en este caso?**

**E:** En este caso claro que sí, había una dura oposición porque no necesitabas ley. No necesitabas ni decreto de urgencia . No necesitabas ni ley de que declare interés ,no necesitabas ni siquiera una ley que cree el sistema nacional de suministro de oxígeno ,no necesitabas porque con el simple hecho con una resolución ministerial, Víctor Zamora o de la ministra Pilar Mazzetti pudieron haber cambiado la resolución ministerial, que aprobaba el petitorio nacional de medicamentos y pasar de 99 a 93% .Por qué no lo hacían Por qué es obvio, que sabían que iban a chocar con algunos intereses y preferían no hacerlo .O pensaban que la pandemia ya pasaba y que esto iba a ser un pico

**I: Y no había necesidad de…**

**E:** No iba a haber necesidad de romper un monopolio un oligopolio. Lo cierto es que se prolongaba la pandemia y eso no ocurría.Pero al margen o no de la pandemia, sí había una necesidad de crear un sistema nacional de oxígeno porque no es posible que con un sistema de salud como el nuestro que es sumamente fragmentado y que cada sector ,o sea cada ministerio ,tiene sus propias actividades de salud ¿no? O un sistema… segmentado donde cada grupo social Resuelve su sistema de financiamiento de su salud a su manera. Entonces es muy difícil cuando no tienes un sistema de notificación universal y tú no aseguras un sistema…

**I: Un sistema de información consolidada**

**E:** Información consolidada y tienes un sistema de fiscalización general de todo este tema. Es muy difícil que tú puedas generar una seguridad en el tema del oxígeno medicinal. Y encima la política fue terrible porque… el presidente Vizcarra con Víctor Zamora a pesar de esas limitantes que estamos conversando ,la primera el que no hay un tema del oxígeno pero tampoco había un sistema de notificación general ,ni vigilancia epidemiológica ;no hay hasta la fecha, no formado lo que es la inteligencia Este… sanitaria para saber cuál es la capacidad de respuesta del país frente a una epidemia o una catástrofe. No tenemos todavía ese sistema montado.El Perú entra a un encierro y condenas a los loretanos a que resuelvan sus enfermedades de la pandemia encerrados, con 5 unidades de cuidados intensivos y sin oxígeno. Entonces, o sea esa gente estaba como en una prisión ,estaba con una presión y un incendio donde el prisionero no puede escapar en ningún sitio y solamente puede rezar para que el incendio no le llegue a su casa ,a su celda. Eso fue lo que vivió Loreto. Eso, eso fue una muy mala decisión . Tú tendrías que haber tenido la capacidad de sacar a los pacientes graves de Loreto, traerlos a Lima

con los cuidados correspondientes pero no podías hacer por el miedo de que Lima no se contagie ;que ya sabemos ahora de que ya estaba. Porque la tasa de mortalidad había aumentado frente a los promedio histórico había aumentado en el mes febrero ,ya desde la quincena de febrero las muertes estaban en alza. Sin embargo entonces los encierros en Loreto, entonces en loreto es donde se ve dramáticamente el tema del oxígeno. A tal punto que la parroquia de loreto tuvo que hacer colectas para poder comprar una planta luego que su autorizó Que el oxígeno podría ser utilizado a partir el 93%

**I: 93%**

**E:** Eso fue lo que pasó con esa norma. Entonces ahí… hay una comisión investigadora en congreso de la república del cual yo soy miembro de la comisión ,el jefe de esa de asesores de esa comisión que no puedo dar detalles ,no puedo referirme a detalles concretos ni de empresas ni de personas; pero que es una línea de investigación que actualmente está en esa comisión para determinar responsabilidades este… civiles penales e inclusive de infracción constitucional en ese acápite.Porque eso es, el tema del oxígeno lo ha visto también la Contraloría ha hecho un informe en el proceso de adquisición de concentradores por ejemplo donde

**I: ese informe ya es público**

**E:** Sí ya es público ,claro que sí. Hay un informe de contraloría donde va donde incluso detecta responsables de quiénes son los responsables, de quienes son los que están involucrados de compra de concentradores ,hay todo el tema de plantas de oxígeno , hay también el proyecto Legado que también hace compras; todo en ese tema el oxígeno ha sido muy opaco. Si bien cierto el oxígeno medicinal es una oxígeno que está en el petitorio nacional, es un recurso estratégico de salud ,debería comprarlos Cenares ,sin embargo eso no ocurría, lo compraban los propios hospitales. Luego cuando viene el tema de la epidemia lo compra Perú compras, lo compra Legado, todo el mundo compra el oxígeno pero al final se compran y se importan balones de oxígeno del Ecuador de una empresa ecuatoriana también que ya… se va a ver luego este es todo un consorcio latinoamericano, no es solamente peruano. Y entonces compran los balones que no tenían los estándares de calidad peruano, no tenían los nanómetros estándar de calidad peruanos y encima estos balones lo compra legado ,legado ,no lo compra el ministerio de salud .Y curiosamente el ministerio de salud le cede los derechos para que ellos compren 'sí que ellos compren, nosotros estamos estresados con la pandemia y no podemos comprar nosotros compren ustedes' y así el ministerio de salud se fue deshaciendo en algunas actividades específicamente médicas que requerían una profesionalización para esas adquisiciones. Fueron pasando a sectores que no tenían nada que ver con eso. A tal punto que… ay más de 1000 balones ,creo que son 14000 balones que están en Legado todavía que nadie los quiere recibir porque no reúnen los estándares de calidad que requiere para ser introducidas en el mercado. Según se han comprado plantas de oxígeno que son tan grandes que no pueden ir a un hospital pequeño como el caso de Pacasmayo. O sea, todo eso es lo que se ha traducido en este periodo de la pandemia. El tema no es solamente el oligopolio , sino el tema no es solamente romper el oligopolio…porque si no cambia la red de juego en el mercado, el oligopolio va a volver a aparecer… porque están dadas las condiciones para que eso vuelva a ocurrir , tú necesitas cambiar esas reglas y esas reglas se cambiaron con la ley que nosotros trabajamos. La idea era poner nuevas reglas ,un poco como el objetivo de tu trabajos de investigación. Qué es lo que hicimos nosotros en ese momento,o sea eso queremos hacer crear un sistema de tal manera que nunca de aquí a futuro un establecimiento de salud por más pequeño que sea tenga estrés por falta de oxígeno

**I: Ok. La norma está creada y ya fue aprobada… el tema es ya está implementada, porque el papel aguanta todo. ¿Que ocurre ahora con establecimientos de salud¿ ¿Qué están implementando? ¿qué pasa con el minsa? ¿qué pasa en los gobiernos regionales?**

**E:** …Esto es un proceso idéntico que el inicial. Nosotros empezamos nuestra conversación señalando que hubo un proceso de desmontaje de oxígeno en todo el país. Ha habido mucha resistencia en aprobar la ley ,primero decían ya hay una norma que se ha dado declara de interés el oxígeno ya para qué van a hacer otra ley, ya el ejecutivo ya dijo que era del 99 el 93% para que otra ley

**I: Pero eso fue como un decreto de urgencia provisional**

**E:** Un decreto de urgencia ¿no? pero igual, pero igual. Ya tiene valor, ya es legal .Además no necesitaba como decía ,con una simple resolución ministerial de aprobar el petitorio lo resolvías. Todo era para decir que está preocupado el presidente en hacer eso. Lo cierto es que cuando se dio la ley, ya la ley grande de sistema ,digamos, de suministro y todo esto; esta ley debería implementarse toda.Pero ahí …es el tema pues ,creo yo, que los ministros o los que están en el cargo han tenido dificultades para siquiera entender la problemática ,siquiera para revisar la ley En su conjunto y decir esto es lo que aporta. Si quiera para buscar el proyecto de ley y del congreso y mirar la exposición de motivos y decir cuál fue la necesidad de esta ley y ahí evidentemente que la comisión de salud del congreso debería de hacer un proceso de fiscalización para el cumplimiento de esa norma.

**I: Que no está haciendo**

**E:** Que no está haciendo. Nosotros, yo le comento el periodo pasado, yo he sido asesor principal de la comisión de salud o sea no este el 2021-2022

**I: No de la comisión investigadora sino de la comisión de salud del congreso**

**E:** Así es ahora estoy en la comisión de investigación del 2022 al 2023 de la comisión investigadora. Pero cuando estuve en la comisión de salud ,nosotros sí hacíamos el seguimiento a ver qué está pasando ;y los avances eran muy bajos ¿no? Eh no sé, no creo que sea solo normativo ya ahora. Ahora tiene más que ver con el tema de que la …sociedad civil, los mismos médicos, los usuarios de los sistemas reclamen. Ahí me queda claro por ejemplo

**I: Pero eso de quién depende ¿Es una cuestión más de gestión institucional, del minsa,o es una cuestión más del recurso humano que está ya en los establecimientos de salud?**

**E:** Es el MINSA, es el MINSA .Por qué motivo , porque el oxígeno está dentro del petitorio de DIGEMID. Quién debería estar interesado en que ese sistema funcione sería DIGEMID. El oxígeno es un recurso estratégico ,quien más debería estar interesado en que funcione sería Cenares. Luego quien ,quien ,quien debería ver que el oxígeno esté su sistema creado debería ser la dirección general de operaciones, todo es MINSA. Los gobiernos regionales aquí no tiene nada que ver, porque ellos tendrían que ejecutar las pautas que les dé la sede central del MINSA. Yo creo que ahí hay un tema central de desorganización o …en algunos debe ser también falta de conocimiento, pero en otros también deben ser intereses creados de no poder manejar este tema del oxígeno que es de gran importancia

**I: En el MINSA**

**E:** En el MINSA

**I: Se hablaba mucho de… a nivel del minsa el tema de asumir responsabilidades 'por esto de no firmo este documento porque no lo entiendo o no lo sé, luego viene fiscalización y soy yo quien tiene que asumir la responsabilidad', es una cuestión más cultural de 'mejor no para prevenir' ¿usted cree que es una cuestión de cultura organizacional, va más vinculado a una cuestión de algún interés aparte que no tenga que ver con esto necesariamente?**

**E:** No. Lo que sucede lo que sucede del ministerio es falta de liderazgo ;te explico… simplemente si tú tienes un ministro que entra a gobernar ,que tiene antecedentes según la prensa ,no digo que sean reales digo según la prensa, 19 procesos judiciales o 19 acusaciones, tiene denuncias por haber presidido una organización criminal en una región¿no? Ha asesorado para que se publiquen normas que van en contra el desarrollo de la salud pública, como por ejemplo quitar etiquetado a los sucedáneos de la leche materna y todavía sobre los… dietas para regímenes especiales, quitar el etiquetado y los octógonos… o que te saca una…una ley este… de residentado médico que por ejemplo va en contra de los jóvenes porque quiere favorecer a grupos de presión que hay en los hospitales ,etcétera. Tú llegas a una conclusión pues, ¿qué firmo? Yo firmo eso de que me pide que firme el ministro, tú tienes muchas dudas de firmar y probablemente piensas 2 veces antes de firmar. Primero yo pensaría 2 veces de asumir el cargo con él, pero además ya estás adentro entonces firmo o no firmo. Entonces qué haces pasas asesoría legal, asesoría jurídica ,haces un informe, otro informe a otra asesoría legal, un informe privado y te llenas de sellos en la resolución solamente para… yo quisiera… no puedo referirme a las investigaciones que hacemos el día de hoy por obvias razones este…

**I: Sí lo entiendo lo entiendo**

**E:** Los casos que tenemos son como los que… como los que ocurría en la época de La Independencia del Perú. Cuando… los caudillos militares, era un patrón ,el caudillo patrón, su grupo de militares que lo rodeaban y empresarios amigos que trabajaban lo que… lo que llama Quirós, el patronazgo ¿no?... El patronazgo militar civil¿no? Que era. Y así entonces 'tú quieres' le decían a Domingo Elías, que era el empresario de ica, 'ya te doy más Tierra, ¿que te falta abono? te doy las islas guaneras, te las entrego para que las veas, ¿qué más quieres? no tienes que trabaje porque porque voy a independizar a los negros, traigo chinos te doy un subsidio por cada chino que traigas, te doy un subsidio de 30 pesos' entonces eso que ha ocurrido en el Perú ,no ha cambiado. Lo que puede cambiar es que no son militares, son políticos ,digamos, los grupos que existen

**I: Los empresarios siguen siendo los mismos**

**E:** Los grupos de empresarios… existen .Y entonces ese es un sistema de gestión corrupta que empezó, según relata Quirós, desde el momento de la Independencia nacional, desde que José de San Martín vino ,porque según Quiroz también fue corrupto. José de la riva agüero, nuestro primer presidente de la república, hizo una empresa igual con el mismo sistema ,corrupto. Vino Bolívar ha sido lo mismo, ojo que había pena de muerte para los

contrabandistas sí se hacía contrabando. O sea,todos estos temas, a raíz de esto , de este seguimiento que ustedes están haciendo acerca del oxígeno se van a dar cuenta. La estructura de gobierno y de congreso

**I: Hablando del tema de empresarios,cuál fue digamos, entiendo que ustedes se reunieron con ellos, ¿cuál fue la postura de ellos respecto a esta nueva normativa que se aprobó?**

**E:** Bueno, nosotros no los consultamos para la normativa. Pero sí les consultamos para tener opiniones para hacer la normativa, porque obviamente no sabíamos si la normativa les iba a gustar o no¿no?

**I: ¿Pero cuál fue la postura que ellos tenían?**

**E:** La postura de ellos fue, que nos dieron la información, nos dijeron que su capacidad está sobrepasada de producción. Sí les parecía bien que se cambie el tema de la concentración porque en ese momento era necesario, porque ellos no lo podían hacer. Fueron muy proactivos, nos dieron muchísima información. Nos explicaron el mercado, porque no es fácil hay mucho… nosotros estamos viendo el mercado formal pero en el oxígeno hay un mercado informal, también hay balones de oxígeno que eso sería, es un tema que hay que investigar también. Hay un mercado informal muy serio, hay contrabando de balones vacíos de oxígeno que vienen de Bolivia ,que vienen de… Ecuador, hay oxígeno… hay balones que son rellenados empíricamente, o sea es todo un problema serio que la industria formal te señala ¿no? te dice 'oigan ustedes nos quieren penalizar a nosotros pero miren esta informalidad' la ley, por eso es que crea un comité de calidad también para verificar esas cosas que dicho sea de paso hasta la fecha no veo que se haya implementado .Por qué… porque uno de los problemas es también… la calidad de la producción del oxígeno. Nada asegura que tu compres un balón de oxígeno en la esquina y en realidad tenga medio balón, o de lo contrario tenga el oxígeno adulterado. Todos esos temas están en la norma y nos expresaron los los propios empresarios. Nuestra relación en ese momento, yo conversaba con ellos a través de la congresista que hacíamos la ley… fue muy…mucha información nos dieron en ese momento que nos permitieron ver un concepto mayor de todo el suministro de oxígeno desde la producción hasta que llega al hospital. Es lo que hemos visto nosotros

**I: A ver ustedes entonces vieron toda la parte… de esta cuestión normativa política. ¿En algún momento habrán visto, o usted habrá tenido la oportunidad por lo menos de ir mirando qué pasaba con la… las plantas de oxígeno que se elaboraron aquí en el país, luego fueron llevados a los hospitales , a los establecimientos de salud?**

**E:** No, no, ahí hay varios temas. Estos temas que usted me pregunta son parte de la investigación que estamos llevando nosotros con responsabilidades. No te puedo dar detalles, te voy a dar solamente en macro. Se importaron máquinas ,se importaron plantas, ese es un tema; ahí ha habido varios problemas en importación de máquina que tiene que ver con la utilidad, con la necesidad con el volumen de producción de oxígeno, la capacidad, todo. Ya es un tema técnico que… que quizá este… cuando salga el informe, que ya sabemos que va a salir, ustedes ¿hasta cuando tiene el trabajo de investigación?

**I: Debemos terminarlo como que ya, porque es un trabajo en conjunto con otros países.**

**E:** Ya, nuestro estudio. Nosotros lo presentamos el 3 de agosto, o sea el 4 ya esta colgado el tema. Pero este… ese es un tema. El segundo tema, el segundo tema es que el Perú quiso producir plantas de oxígeno propias con la universidad de la UNI

**I: Y la Católica también por su lado**

**E:** No, no. La Uni fue, los otros eran proyectos. Igual hicieron lo de la marina, otras empresas pero no. El proyecto formal con una inversión importante fue el proyecto de la UNI que fracasó ¿no es cierto? No pudo cumplir con las máquinas completas y a raíz de que ese fracaso es que el Perú ,ese dinero lo pasan al Legado para que compre del Ecuador. Ya, ese es un tema ya que hay comprometidos en este tema porque finalmente no resolvieron el problema, fue un gasto inútil. Ese es el tema de las plantas. Ahora, yo he visitado plantas… y… algunas plantas ya no funcionan porque no tienen necesidad y les y les es más costoso hoy en día

**I: Hacer el mantenimiento**

**E:** Hacer el mantenimiento que comprar los balones de oxígeno. O sea ese es un tema que va a ocurrir ,mucho me temo que pasado un tiempo logremos estar como estuvimos antes de la pandemia, porque es obvio que mantener un sistema de suministro de oxígeno tiene un costo que debe estar presupuestado en la ley de presupuesto. Si eso no logras, en esta ley de presupuesto que se va a aprobar este año, es imposible que tengas el mantenimiento de todas las plantas de oxígeno a nivel nacional… hay hospitales que tienen dos plantas de oxígeno y solamente están trabajando con uno, con la más chica… y la otra obviamente se va a deteriorar por…

**I: Por el no uso**

**E:** Por el ya no hay necesidad de uso del oxígeno. Luego… también el diseño hospitalario, antes los hospitales se diseñaban con el oxígeno empotrado¿no es cierto? Con el oxígeno empotrado, como el gas en las casas. Pero luego eso ha cambiado y ya no se incendia el oxígeno. Entonces ahora creo que va a tener que cambiar en el diseño hospitalario; que tiene que ver ya con el diseño e infraestructura del ministerio. O sea muchos son los cambios que se tienen que hacer si es que queremos que se cumpla la ley, y asegurar que hasta el centro de salud más chiquito tenga oxígeno. Les voy a poner ejemplos… si usted se va a Huancaya, de turismo, Huancaya queda de Lunahuaná más arriba, como para irte a Huancayo,es un lugar precioso turístico pero que está casi 4000 m de altura. Es posible que un niño o una niña tengan mal de altura, tú te vas al centro de salud y no hay oxígeno, ese niño se va a morir salvo que bajó inmediatamente a la costa . Bueno le comento que ahí ha habido una muerte hace poco, precisamente por falta de oxígeno. Entonces, eso a pesar de nosotros era un país de altura, que sufrimos de mal de altura ,etcétera; no tenemos asegurado en estos establecimientos de salud del ande oxígeno. Yo he viajado a Coracora me he ido al, como yo siempre he estado investigando el tema de oxígeno, me he ido al hospital de Coracora y del MINSA, oxígeno no hay, me fui a essalud ,no hay oxígeno. El único balón que había de oxígeno era el que tenía la ambulancia, era para que el paciente que ya esta mal lo subas a la ambulancia y lo bajes Nazca, a la costa para que ya respire. Entonces esos temas no se resuelven de la noche a la mañana, eso requiere una gestión ministerial que tenga la idea fija en crear ese sistema de seguridad de oxígeno medicinal en el país. Y esto tenemos que esperar y esperar hasta que tengamos gente preparada que ocupe el cargo del ministerio de salud

**I: ¿Qué otras dificultades ve usted en la implementación de esta norma? Además de la que ya mencionado una cuestión más de estructura de los establecimientos de salud, recursos humanos, financiamiento…**

**E:** Los temas son, realmente son cuatro los temas. El primero el fraccionamiento del sistema ,que hace que cada… cada ministerio elabore sus propias normas y no haya posibilidad de articular una política en conjunto ;que lo dice la norma pero una cosa es lo que dice la norma y otra es que lo hagan en realidad. El segundo tema es sistema de información, nosotros necesitamos la notificación obligatoria de cada cada balón de oxígeno que hay, tengo que hacer una trazabilidad del oxígeno, el consumo histórico del oxígeno en cada uno de los lugares y eso tiene que estar una notificación al... a Digemid ,a Cenares y sobre todo al CDC; porque el CDC debe tener, que no tiene hoy día… le he planteado una norma que estamos proponiendo estos días, que ojalá la norma… la ley se apruebe… estamos pretendiendo fortalecer el CDC pero agregando una nueva competencia que es en la competencia que monitorea la capacidad de respuesta del estado como país frente a una epidemia, que eso no tenemos. Entonces ya ahí podría centralizar esta información, en modo obligatorio. Y hacer que funcione el Renace que es el registro nacional de… los epidemiólogos, epidemiología a nivel nacional, que vayan a todos los sectores.Eso se sería un segundo punto, entonces el tema de la.

Tercer punto es de que esto esté incluso incluido en la ley de presupuesto, no solamente la compra del oxígeno, sino también el mantenimiento de los equipos y todo el sistema que tiene que estar monitorizado… y cuarto y último punto, debe estar muy bien claro, estandarizado el uso de oxígeno medicinal en todos los establecimientos de salud. O sea, que tenga noción la gente en qué caso se debe usar oxígeno y cuando es importante. Por ejemplo, si a usted la nombran jefa de un establecimiento de salud como Huancaya que ya hemos conversado, usted no debería estar ni 1 minuto sin que usted no tiene oxígeno en su establecimiento de salud. Y hablemos de aquí nomás, de Matucana, hablemos de aquí nomas en la parte de altura; o sea no podría usted aceptar eso. Esto ya es parte de las funciones que debería tener cada jefe de cada establecimiento de salud en ese tema y eso no está tampoco trabajado. O sea el tema… es es muy complejo¿no? ese es en el tema sanitario pero hay un tema de la industria que también eso tiene que ser este... Trabajado¿no?. Nosotros tenemos que propender a que no haya oligopolio. Según, como dice la constitución tenemos que abrir el mercado y que haya más competidores y es probable que aquí haya incluso posibilidades de que participe la pequeña industria… que pueda producirse en mayor cantidad del oxígeno. Lo cierto es que… en la panademia hemos tenido casos como el siguiente¿no? Que hay gente que si tenía plata se compraba un concentrador en Estados Unidos no podían traerlo al Perú

**I: No podían traer al Perú**

**E:** Por qué porque no estaba homologado el…el producto en el país y, DIGEMID tenía que darle el visto bueno. Y para ingresarlo… pero no podía darle el visto bueno porque no había la norma que diga que es correcto o no, tenía que pedirle al propio comprador,al propio paciente y decirle ‘dame tu prospecto’ y compararlo con estándares de Estados Unidos y ver si es que ese prospecto reunía el estándar de Estados Unidos luego emitir acá una licencia. Sacaron una licencia especial de importación ,solo para el paciente, o sea tenías que ir… era increíble ,le pedían el médico que receta concentrador, le decían al médico ‘recetame la palabra concentrador de oxígeno’ ¿por qué? porque sólo así puedo meter mi producto, un producto. No podías traer 10 productos ‘para mis vecinos de la cuadra’ no podías traer; porque no estaba homologado el producto en el país. Entonces por otro lado se han traído equipos que nunca se han utilizado también es cierto, que la gente también tratando de apurar todo eso… esto ha ocurrido. Hay gente que en … que han venido de España con su concentrador , venían por si acaso han traído su concentrador al Perú y cuando han llegado a la aduana no los han dejado entrar. Y así ha quedado, habría que investigar cuántos concentradores habrá pues retenidos en la aduana del país

**I: Hasta ahora**

**E:** Hasta ahora ¿no? Porque este… eso era recurrente. Entonces ese tema requiere un trabajo de parte del ministerio de la producción en colaboración con el ministerio de salud para tratar de promover la competencia, de promover la competencia en este mercado de oxígeno. Y el otro tema que hay que promover es la autoproducción de oxígeno en los establecimientos de salud, o sea que…

**I: Que no dependan de la compra**

**E:** que no dependen de la compra y venta de esto. Y la última lección que hay que sacar es que toda epidemia o pandemia es un negocio¿no? Para unos es un negocio; y obviamente ahí no se puede hacer nada porque eso que ocurre ,ese negocio hace que la gente ayude en la epidemia. Aunque no necesariamente sea altruista pero resuelve los problemas de la gente en ese momento. Si no hay agua y vendes el agua 5 soles resuelves para la gente que puede comprar el agua ¿no?. Entonces,y el estado debería intervenir ya, el gobierno debería intervenir ya para que pueda palear esto. ¿Por qué digo esto? porque hubo la intención durante la pandemia de querer estatizar cosas ¿no? dicen'hay abuso , hay que estatizar las clínicas, hay que estatizar las fábricas estas de oxígeno porque hay abuso' , no se resuelven así las cosas, las cosas se resuelven cuando hay una sobredemanda de algo es obvio que va a ver gente que entra al negocio y cuando entra al negocio los precios van a bajar porque hay más competidores. O sea no es un tema de mercado, pero hay picos donde sí genera algún desajuste y se puede prestar a el enriquecimiento rápido de la gente, de algunos empresarios

**I: El estado debería intervenir como para regular esas situaciones**

**E:** Exacto el estado debería intervenir; no, más que regular,modular ¿no? Es decir oiga ,en caso del oxígeno cada balón estaba a 2500, 3500, hay gente que ha pagado hasta 7000. Esta el caso de la señora Capira, que corría en Arequipa detrás del presidente Vizcarra por un balón de oxígeno. Entonces en esos casos claro el precio sube a 7000 y habría que pagarlo,no tienes otra opción. Por qué por qué para que otros actores se metan al mercado y digan '¡oh 7000! yo también voy a vender oxígeno' entonces eso va a ir abaratando más los costos. El problema es si tú cierra las fronteras,como pasó en Loreto,si cierras entonces ¿qué haces?. Eso, esa medida del confinamiento obligatorio que fue la del Perú una de las más drásticas del mundo, fue totalmente terrible para la lucha contra la epidemia

**I: Hablando de Iquitos, entiendo que allá digamos... Se implementaron 5 plantas de oxígeno.¿Sabe usted si están en funcionamiento?**

**E:** No, no, no están en funcionamiento. Yo he estado, he visitado Iquitos. He ido en Iquitos y solamente del hospital regional que tiene 2 grandes plantas la más chica funciona y la otra no funciona. La donada tampoco funciona, por la parroquia. Ya en el tema de…

**I: Pero no funciona por una cuestión de que no hay la necesidad o porque está malograda**

**E:** No hay necesidad, no hay necesidad. O sea… de ahí decía de que mantener lo que casi no necesitas mucho, no va a ser prioritario.Y va a pasar ,va a pasar lo que me temo; como dice el dicho 'de lo que temes no escapas' va a pasar lo que me temo. ¿Cuál es el temor que tengo? que volvamos al sistema anterior en el que como no se usa mucho oxígeno y es difícil de tener el mantenimiento de las plantas ,etc este… vamos comprando mejor

balones o ponemos isotanques y ya necesito oxígeno como el gas, gas te trae el gas y ya esta,más fácil que producir tu gas

**I: Pero por ejemplo qué pasaría si de aquí a unos meses, o que se yo de aquí unas semanas nada más , como ocurrió con la pandemia tuviésemos una nueva situación crítica como la que tuvimos en el 2020 por el tema del oxígeno ,de la crisis**

**E:** Claro esa es la hipótesis que trabaja la ley por eso es que la ley crea el sistema, y hay un tema de mantenimiento y un tema de todo dentro de la ley. Pero este… no piensan así los políticos, no piensan así los gestores, no… no piensan en el largo plazo, no piensan en mantener eso como una estructura en funcionamiento. Yo le voy a contar una anécdota, que ya la he pasado y también la he investigado. Que es el tema de los grupos electrógenos.Cuando vivíamos la época del terrorismo, la subversión, o como la quiera usted llamar, la guerra interna como, le quiera usted llamar según la ideología que uno tiene, tiene el nombre, ¿no es cierto?, habían apagones a cada rato. Entonces los hospitales compraban grupos electrógenos, es más los donantes, los cooperantes los regalaban. Grupos electrógenos para los hospitales, que eran grupos conectados a la red ,de tal manera que cuando había un apagón automáticamente entraba el grupo electrógeno. Pero pasó el tema de la subversión, entonces este… ya no había necesidad de grupos electrógenos, entonces había una directiva en la que cada 15 días tenía que…

**I: Usarse**

**E:** Comprar el petróleo, echarle su petróleo, poner a funcionar el motor durante unas 4 horas que funciona el motor ininterrumpidamente y lo detenías. Y eso cada 15 días, y así sucesivamente.Y había que gastar de caja chica, comprar el petróleo y así sucesivamente. Bueno cuanto más tiempo pasa después que no habían apagones ya no funcionan… ya no funcionan, tienen los grupos electrógenos abandonados.Punto dos, en las salas de operaciones, hoy día la sala de operaciones se usan cialíticas que tienen baterías incorporadas.Entonces se va la luz ,entra la batería;esa batería requiere mantenimiento permanentemente. Pero qué ocurre que como no se va la luz, la batería no se mantiene. Cuando realmente ocurre un apagón

**I: No funciona**

**E:** Ya no funciona la cialítica y tienen que terminar la operación con linterna. Entonces, todo ese tema ocurre porque no existe un programa nacional de mantenimiento que obligue a esas cosas. En el caso de los grupos electrógenos no hubo una ley para esto, pero que sí la tenemos en el tema del oxígeno que deberíamos aprovecharla y exigir que la ley de presupuestos tengamos eso y que los funcionarios tengan en perfecto estado estos sistemas de oxígeno. Ahora el tema del oxígeno es grave porque hay muchas enfermedades respiratorias, requiere nebulizaciones, o sea si hay necesidad de oxígeno. Aunque es menor la cantidad de oxígeno, sí se necesita por lo tanto es totalmente inexplicable por qué en las postas y centros de salud ,de provincia sobre todo, no se fijan en su seguridad de existencia del oxígeno.

**I: Yo no tengo más preguntas pero no sé si quiere comentar algo más, algo que tal vez no hayamos conversado aquí en este momento que considere que es importante de conocer para el estudio**

**E:** Creo que si sería importante que revisen los documentos emitidos por instituciones como defensoría, contraloría… informes que… preliminar que hay en el congreso sobre algunas cosas. No por nada también mi bibliografía lo que yo he escrito sobre el tema y… de repente con eso podemos armar otras preguntas y estaría (Ininteligible, MINUTO 49:05) a responder porque a veces uno expresa todo lo que tiene yo porque no hay un plan establecido para eso, con eso yo creo que sería todo. Gracias

006 Entrevista

Meeting started: 30/6/2023

Participantes: E, I

I: Investigador

E: Entrevistado

**I:Hoy es 30 de junio del 2023. Mi nombre es Lizzete Najarro, y voy a conversar con un informante clave para el estudio 'economía política del sistema de oxígeno medicinal en el Perú' que está siendo llevado a cabo por la Universidad Peruana Cayetano Heredia. Sólo para una cuestión de registro,como para tener el contexto de con quién estamos conversando, por favor nos podría comentar cuál es digamos su tarea, su labor a qué se dedica usted**

**E:** Soy gerente general (Ininteligible, MINUTO 1:00)

**I: No le entendí, no le escuché bien**

**E:** Me desempeño como gerente general de un gremio empresarial líder en el país

**I: OK. su relación con el tema del oxígeno medicinal en qué momento es que, usted Empieza a trabajar en ese tema, digamos a colaborar con ese tema porque no es propio una actividad de un gremio empresarial**

**E:** Así es el que en el marco de la pandemia nuestra institución,junto con otras 2 instituciones decidieron hacer una iniciativa de apoyo al a la a la mitigación de la pandemia a través del sector privado y la academia, y de esta forma generamos un movimiento que logró recaudar fondos del sector privado para atender la necesidad. Entre las necesidades que pudimos apoyar una decisión conjunta con los principales actores que estaban actuando en el (Ininteligible, MINUTO 2:10) salud ,Essalud ,entidades (Ininteligible, MINUTO 2:17),universidades y demás …entramos con diferentes iniciativas como fabricación importación, por un lado de plantas de oxígeno, luego motivación de producción nacional de plantas de oxígeno y su respectiva adquisición para donación a hospitales en el país. Y en paralelo también algunos equipos importantes como respiradores mecánicos de un solo uso que se usaron en otras iniciativas a nivel Sudamérica con bastante éxito ¿no? y algún apoyo en EPP (Ininteligible, MINUTO 2:52-2:57) bueno y en mi caso, dentro de esa dinámica yo he (Ininteligible, MINUTO 3:0) entre las funciones involucradas nos dividimos funciones administrativas, de comunicaciones(Ininteligible, MINUTO 3:07) y en mi caso (Ininteligible, MINUTO 3:09) yo decidía a que planta comprar con un grupo especializado de médicos decidíamos a dónde debe ir destinadas esas plantas, con un análisis técnico y me tocaba ver también la situación de los hospitales (Ininteligible, MINUTO 3:28)

**I: Sí, discúlpeme le escucho entrecortado por momentos**

**E:**Yo te escucho, yo te escucho muy bien. No sé por eso te comentaba que (Ininteligible, MINUTO 3:43) he cerrado la ventana por eso. Pero no sé si ha mejorado

**I: Ya ok. Entonces entiendo que ustedes como que se organizan pero ¿cuál fue la motivación que los, que hizo que se organicen para precisamente orientarse a esta actividad, a este apoyo?**

**E:** Bueno dos cosas¿no? Primero, la convicción de que las entidades privadas como las nuestras debían tener una participación más activa en los problemas de la sociedad civil. Venimos trabajando, ya veníamos usando iniciativas vinculadas a salud, educación y con esto la pandemia obviamente buscamos tener un rol activo… y por otro lado también¿no? Como una…sabiendo de las, primero las deficiencias y dificultades que tiene el Estado para atender la pandemia; decidimos actuar con el sector privado que evidentemente nuestros mecanismos de acción son más dinámicos que el del Estado.

**I: Hablando de temas del estado. Ustedes para ese entonces tenían información acerca de las políticas que se venían, se venían usando, implementándose destinadas a este tema del oxígeno medicinal.¿Tuvieron alguna dificultad?¿Había alguna norma institucional que ustedes conozcan o la que apelen para el trabajo que hicieron?**

**E:**Lo bueno fue que hicimos un equipo técnico conformado también por autoridades¿no? Autoridades, le llamábamos nuestro 'consejo médico' en el cual estaba el ministerio de salud, estaba Essalud , estaban doctores notables que en ese momento estaban liderando la lucha contra el covid. Así que ellos eran los nos daban la pauta; entonces de esa manera teníamos a través de ellos acceso a información técnica de dónde estaba ubicada los focos en el momento que… por ejemplo digamos, no sé estamos viendo adquirir una planta de cierta capacidad , entonces el consejo médico era quien decidía la ubicación de la planta. Entonces obviamente todo estaba enmarcado en, en estos procesos. También es cierto que tuvimos mucho apoyo lo que es Aduanas para facilitar el ingreso de plantas con exoneraciones propias del estado de emergencia¿no? Todos los equipos que traíamos entraban,lograban, tuvimos el apoyo máximo. Es más,solo como anécdota un embarque de respiradores médicos de un solo uso que aterrizó a las 6:00 de la mañana; a la 1:00 de la tarde ya estaba ingresando en los almacenes de salud por ejemplo¿no? Así de dinámico logramos hacer las cosas.

**I: Eso fue digamos ¿por una cuestión de seguimiento, de aplicación de una norma o un reglamento? O fue más por una cuestión más de interés personal de todos los implicados que hicieron que de pronto vamos a poner esto en marcha, vamos a hacer que todo…**

**E:** Fue una aplicación máxima de una normativa existente, o sea sin saltarnos la normativa pero por ejemplo hicimos… importación anticipada, o sea ya desde antes la aduana ya sabía que iba a llegar el embarque, en que vuelo todo destinado , coordinado para que haya un supervisor. Entonces todo el proceso se hizo muy rápido, pero digamos… siguiéndo los requisitos de oficio¿no? Sin romper el procedimiento pero dándole la máxima prioridad

**I: Y eso fue así desde el inicio del trabajo de ustedes o digamos se regularizó…**

**E:** Mira, dependía mucho de las personas lamentablemente¿no? En todo este proceso si algo aprendimos es finalmente depender muchísimo de las personas. Porque con los cambios que empezaron a haber, sobre todo en la segunda mitad de la pandemia, ya se perdieron varios procedimientos, varias cosas que hacíamos muy ágiles se dejaron de hacer. O sea sí dependía mucho de la buena voluntad y la disposición de las personas que estaban a cargo de todo. De los diferentes procesos que involucraban cualquier cosa… que involucre instalar una planta, ubicación de las plantas, adquisiciones que tienen que hacer los hospitales para recibir las plantas , admisiones, contrataciones que tienen que tener. Ahí dependía mucho de las capacidades de los profesionales encargados de cada uno de sus temas

**I: Me comentaron que alguna institución tuvo dificultades, por ejemplo para el tema de desaduanar equipos que habían sido donados en el marco de la, de la ley que tiene que ver con las situaciones de emergencia o desastres naturales. Porque el tema de la pandemia del covid no estaba dentro de lo que era considerado un desastre o una emergencia¿eso también se aplicó ustedes o…?**

**E:** No, en lo absoluto. Yo Lo que siento es que hubo mucha desorganización de los que importaron. A ver, es cierto que hay una ventaja adicional que no te he comentado. Mi línea de desarrollo ha sido comercio internacional o sea yo antes de ser gerente general era gerente de comercio exterior de la Cámara de Comercio de Lima y antes de eso trabajé en Prompex y Promperú entonces la parte de comercio exterior la conozco bastante bien; es más soy profesor en una universidad entonces conozco muy bien los procedimientos y hice una… o sea el estar bien informado pude hacer una red y hacer todas las anticipaciones posibles, todas las

conversaciones previas. Entonces cuando ya llegaban las cosas ya no había nada que concretar, ya estaba todo conversado, todo allanado. Y noté, sabía de casos de gente que me decía 'me ha pasado esto'pero podía escarbar un poquito y la verdad me había dado cuenta que había unos errores casi por inexperiencia, por no decir otra cosa, muchos errores por inexperiencia, de pasos que se habían querido saltar y la gente pensaba que 'no,como es emergencia, todo se arregla'. Y no, hay cosas que lamentablemente igual tienen que seguir los procedimientos. Entonces yo creo que es la inexperiencia y las ganas de hacer ese activismo que nos apresuraba este… sin tener en cuenta todos los aspectos y procedimientos de importación. Porque sí había cosas que no podían dejar de ver. En lo que sí a veces sentíamos… que era unas instituciones se requería permisos obligatoriamente a pesar de que nosotros sí teníamos que pasar el control posterior este… sí nos complicaba un poco. Básicamente una institución, que fue DIGEMID.

**I: ¿Como en qué casos?**

**E:** Por ejemplo la planta no podía salir de almacén mientras ellos no la vayan a inspeccionar. Entonces en algunos casos la inspección tardaba… tampoco es que fuera mucho pero tardaba un par de días cuando ya el hospital obviamente nos llamaba cada hora para que le despachen la planta para ver cómo iba, porque están desesperados de tener la planta y tenían todo listo y no se despachaba porque faltaba que un funcionario de campo se de el tiempo de ir a ver la planta, entonces... Claro, estaban igual con muchas cosas pero igual este, había esa limitación. Pero después,pero igual se solucionaba; o sea igual llegaban ,igual despachabamos; era un cuello de botella más que una falla procedimental. Pero por procedimiento no, te digo no hemos tenido en ningún caso ningún problema. Pero claro, como te digo habiendo hecho las coordinaciones previas bien hechas

**I: ¿Usted sabe si se revisan o evalúan las directrices, las política, la normativa en relación al tema del oxígeno medicinal? Para facilitarles la importación, para facilitar el desaduanaje, qué sé yo en todo este periodo que tuvimos tan difícil por la carencia del oxígeno.¿Se dio alguna de estas cosas? ¿Se hizo alguna revisión de las normas o algo parecido?**

**E:** A ver a ver si me puedes… no entendí la naturaleza de la pregunta

**I: Sí. Le preguntaba si usted tiene información acerca de si ¿estas directrices, estas normas que rigen la el tema de las donaciones ,el tema de las importaciones fue revisado, fue observado, fue mejorado que sé yo en el periodo de la pandemia?**

**E:** Sí,claro. El estado de la pandemia facilitó porque… mejor dicho había facilidades para todos los procesos de importación de productos, no recuerdo si hubo una norma en particular pero sí la normativa de emergencia incluían tener prioridad a los casos vinculados a operaciones de comercio exterior. Lo cual evidentemente, la importación de productos estaba de… para la pandemia estaba haciendo parte de¿no?

**I: OK. ¿Usted considera o sabe si son suficientes las políticas para el oxígeno medicinal? Las que tenemos en el país suponiendo que se pase o se tenga una situación similar en el futuro**

**E:** A ver estamos hablando de 2 cosas diferentes¿me estás hablando del provisionamiento?

**I: Sí.También de las facilidades que ustedes tuvieron o tendrían sí se tuviese en el futuro una situación similar y se tuviera que intervenir nuevamente**

**E:** Mhmmm. Sí hay problemas serios¿Cuál es el problema? A ver pero es un tema de política, no de provisionamiento por eso te preguntaba. Como provisionamiento existen ,los hospitales tienen dos formas de proveerse. No hay más, no existen más. Por planta o generación de oxígeno ,dentro de los cual es hay dos sistemas principales, uno es la planta de oxígeno que nosotros trajimos y conseguimos que se fabriquen en el Perú que es digamos… una planta que toma oxígeno del ambiente, lo filtra, lo concentra y lo lanza a través de una tubería al interior de un establecimiento médico. Dentro de esa rama el segundo proceso usual ,es lo que se llama 'oxi-tanques', viene más o menos como un sistema de gas que tú ves en los restaurantes viene un camión o una cisterna , llena con oxígeno líquido un tanque y luego la máquina lo que hace es licuar ese, esa, ese líquido para que se convierta en gas¿no? Listo, entonces ya tenemos esas dos este… esas dos formas de proveer oxígeno.

Y por el otro lado está el sistema de los tanques individuales, que es el que se hizo más famoso porque veíamos a toda la gente andando con su cilindro¿no es así? Ya, esa es la otra forma. Según tengo entendido porque no he profundizado en la investigación tal vez sería un tema para ustedes que sea complementario para ustedes, en los hospitales a nivel mundial cualquiera de los dos primeros sistemas sea el de plantas de oxígeno o el de oxi-tanques son los más usuales. Por qué, porque significa que cada cama tiene un terminal¿no? Como si fuera un enchufe igual uno de los terminales es un terminal de oxígeno y simplemente conectan a una red y listo, se acabó. Eso fue lo que nosotros hacíamos, pedíamos y eso es parte de lo que te decía que nos cedieron las contrataciones que querían hacer los hospitales porque no tenían redes para oxígeno, entonces ya tienen la red y simplemente llevan un tanque principal y listo. Pero en el Perú, este por motivos que toca investigar ,porque la verdad es que he escuchado diferentes versiones del origen, se utiliza mucho el cilindro individual. Lo cual es más costoso ,más ineficiente… pero también se presta mucho más fácil las prácticas … deshonestas digamos lo de alguna manera ¿ok?. Entonces… dicen que esa es la razón principal. No, no me consta pero lo que sí puedo decir es que hoy en día yo sé que hay hospitales que están dejando de usar las plantas de oxígeno para regresar al sistema de balones de oxígeno¿no? Entonces el problema va a ser igual, o sea cuando y la planta la están dejando malograr, entonces… cuando mañana más tarde vuelva a ocurrir una experiencia de este tema, lo primero que va a pasar, ocurrió en la panderia, ustedes seguramente no sabían esto pero, lo primero que se hizo fue tratar de activar alguna de las 13 creo que habían plantas a nivel nacional, solo 13 este…y menos creo que 11, 11 o 13, una de las dos. Y no había cómo repararla, porque claro estamos en pandemia, no habían repuestos o si no te cobran un ojo de la cara pero era simplemente que las habían dejado morir y habían regresado a los sistemas tradicionales. Estamos hablando de hospitales nuevos, uno de ellos era el hospital del niño. Que digamos bueno no es de ayer pero es relativamente uno de los últimos, tenía planta de oxígeno en su diseño y le habían dejado morir la planta. Estaba fallando, estaba en 10, 20% de capacidad, o sea no servía para lo que se necesitaba pero estaba, no había cómo repararla. Y así como ese, otros casos a nivel nacional de hospitales grandes que eran los que tenían plantas. Entonces eso es parte del problema, si Perú tuviera una red instaurada que los hospitales ,no sé la parte técnica pero entiendo que los hospitales grandes deben tener plantas de oxígeno y es más 2 plantas oxígeno, cosa que en el caso falle una bueno se van alternando como cualquier sistema ¿no? Sistema con backup, con 2 plantas de oxígeno que van turnándose para operar en la operatividad normal y en caso una falle, la otra entra en pleno funcionamiento mientras la otra se arregla. Que es el usual en cualquier sistema ; y adicionalmente ya durante la pandemia cuando ya las plantas estaban funcionando sí algunos hospitales tenían un stock de balones de emergencia, ya ,ya. Tienes ahí 50,60, 20 lotes ,fuera de balones en caso falla jalas de ahí pero es un sistema de respaldo no es el sistema de todos los días. Entonces por eso te decía, el provisionamiento de oxígeno va a tener problemas si es que las plantas de oxígeno se dejan morir, no se dejan de utilizar y no se incorporan como parte del abastecimiento regular de un hospital. Porque mientras se, en un caso como la pandemia que se disparó la demanda ,se disparó porque el problema no es que no fabricaramos, el tema es que de necesitar 100 el día se necesitaba 1000 al día. Claro pues, materialmente una planta de oxígeno, la planta ,la fabricante que llenan cilindros estaban al 100%, 110% de capacidad y simplemente no había cómo abastecer. Entonces ahí es donde empieza el problema, si vuelve a ocurrir y se sigue apoyándose solamente en la las los cilindros sí va a haber problemas. Las plantas oxígeno que hay en el país ,que ya hay más de 100 plantas que se han donado. Estamos hablando de en promedio son 25 más o menos tienes 25000 $m^3$ de producción que podrías a partir, no perdón qué hablo, 2500 $m^3$ al día, es un montón ,mucho más de lo que se necesita entonces no puedes perder esa capacidad instalada por desidia o mala administración de parte de quien administra el hospital. Porque recuerden que acá hay 3 actores; en orden de eficiencia Essalud es el más eficiente, segundo el MINSA , tercero los hospitales regionales. Ese es otro tema, los hospitales regionales tienen serias, serias falencias para poder administrar; por lo menos en lo que a mí me concierne en el sistema de salud lo he notado es bien complicado, bien complicado lo que ocurre en los hospitales administrados por los gobierno regionales. Creo que me he saltado temas.

**I: Sí, luego les vamos a ir revisando. Pero volviendo con el tema de la parte normativa ,ya usted me ha hablado del tema de provisionamiento. Y para el tema de la importación, de las facilidades que puedan tener para agilizar los procesos para traer, para que las plantas que se han importado puedan salir rápidamente de aduanas para que todos los procedimientos sean como más ligeros**

**E:** No no hay problemas, no hay problemas. (Ininteligible, MINUTO 20:26) es con conocimiento y por mal procedimiento.Y te lo digo con (Ininteligible, MINUTO 20:31) causa, las plantas de la UNI tuvieron retrasos por temas aduaneros que lo solucionamos con una llamada. Una(Ininteligible, MINUTO 20:38) una llamada es que solucionó el problema. Es simplemente, lamentablemente es desconocimiento. No… yo no veo

sinceramente que sea otra cosa; y bueno el cuello de botella en DIGEMID porque hay algunas inspecciones obligatorias que bueno dentro de todo hace sentido. Finalmente ellos son la autoridad que regula el ingreso que son para contacto con el cuerpo ¿no? Medicinas, cremas, etcétera, cosmética. Entonces ellos son los responsables que hagan una inspección mínima, sí de repente se podría variar un poco el procedimiento para que en algunos casos que se haga ex post y no ex ante pero fuera de eso no, en realidad no hay. Es más Perú tiene uno de los sistemas de aduanas más rápido de Sudamérica, o sea de hecho si lo haces bien no debes tener problema, pero el problema es ese. Entran actores que no son operadores de comercio exterior y ahí es donde empiezan los problemas.

**I: ¿A quienes se refiere con con que entran operadores?**

**E:** Importadores, empresas que a veces quieren… hay 2 casos, los que quieren aprovechar la oportunidad comercial y los que (Ininteligible, MINUTO 21:53) simplemente son operadores que no suelen hacer importación; quieren entrar al tema de la importación y no se separan adecuadamente

**I: ¿Usted observó esa situación durante la pandemia durante la pandemia? hablando solamente del tema del oxígeno**

**E:** Sí, claro. Porque nos llamaban, como sabían que nosotros estábamos ágiles. (Ininteligible, MINUTO 22:12) Muchas llamadas,'oye cómo has hecho con esto, como has hecho con lo otro ,hablo con fulanito, hablo con menganito'(Ininteligible, MINUTO 22:20) y ya estaba. Todo eso pasaba todos los días, por lo menos una vez por semana nos llamaban para hacer alguna consulta.

**I: Entiendo que Respira Perú, que fue digamos la organización por la cual ustedes canalizaron el tema de las donaciones…**

**E:** No, fue un colectivo creado por nosotros. Fue creado por la sociedad nacional(Ininteligible, MINUTO 22:45) y la conferencia episcopal.

**I: Así es ,así es. Fue un, digamos una organización que canalizó todas las donaciones del país de donantes tanto de empresas privadas como de personas comunes, instituciones y personas comunes…**

**E:** Una precisión, respira a Perú fue un colectivo. Digamos una institución aparte, fue un colectivo. Y eso es lo que te decía cuando nos dividimos la responsabilidad. El dinero ingresaba a través de la conferencia episcopal para que dé las garantías del caso, las donaciones sí lo veíamos(Ininteligible, MINUTO 23:27) la sociedad nacional de industrias y la parte de comunicaciones era que le correspondía a USIL

**I: De acuerdo¿cuáles fueron las dificultades o los retos para ustedes al momento de precisamente trabajar? porque entiendo que, por lo que me dice también, el episcopado era quien digamos tenía o recibía los fondos y ustedes eran quienes debían canalizar estos fondos¿cuál fue la dificultad? si es que las hubo o no mayores retos para digamos cumplir con esa tarea**

**E:** Digamos en ese momento las empresas estuvieron muy dispuestas a apoyar. O sea, si bien es cierto sobre todo las que pudieron mantenerse en operaciones, las que estuvieron fuera de operaciones ya obviamente por motivos económicos y de presupuesto les fue más difícil aportar pero sí recibimos muy buena receptividad de las empresas para poder este…hacer donaciones ¿no? Recibimos alrededor de 16 millones de soles y de todo tamaño de empresa; algunas corporaciones grandes nos dieron millones y otras este… con otras medianas trabajamos el esquema por planta. O sea 'dóname media planta' que estaba valorizada en tanto. Y eso era así, como que te dan el 'brandeo' o la visibilidad adecuada. Pero básicamente era un tema de disponibilidad de recursos, algunas estaban muy golpeadas por el cierre de operaciones, otras no tanto, pero por ahí vinieron las limitaciones.

**I: Usted me ha hablado hace un rato de que había más dificultades para una vez teniendo la planta implementar… las plantas ya en las regiones por ejemplo, en los hospitales, en los establecimientos de**

**salud de las regiones¿cuál es la dificultad que usted observaba a nivel de equipos, infraestructura recursos humanos?¿Cuál, cuál era?¿ qué pasaba?**

**E:** Todas las anteriores. A ver, sí a ver… a ver para ser, cuál habrá sido lo más notable. Te cuento un poco el proceso para que me entiendas por dónde iban los problemas. Por ejemplo teníamos un… a ver, a ver, a ver… Ya,llegábamos después de la decisión de la compra de las plantas de oxígeno, nosotros nos tocaba ya ir a ver este… se decidía a qué región iba a ir y ya luego yo me contactaba personalemente con el director del hospital o con el director regional de salud ¿no? Y le decíamos ya, que se había decididoq ue una planta vaya para ellos así que había que hacer la preparación, OK. Muchas veces ahí empezaban los problemas, porque a veces la parte de los recursos técnicos que tenían los gobiernos regionales, personal técnico no era el idóneo, o no entendían bien lo que tenían que hacer para subsanar la situación, no entendían bien la parte técnica porque la planta tenía requerimientos de infraestructura vinculados a energía, vinculados a… a parte física ¿no? Necesitaban una losa para poder ser instaladas; entonces se requería de parte de ellos que tomen ciertas decisiones internas. Ya ahí, es como te digo empezamos nosotros a notar que no sabían bien lo que se necesitaba. Tanto así que en gobierno regional, un hospital, lamentablemente nos dijeron que estaban avanzando todo y cuando llegó a la planta este… no la tenían, no la tenían lista de la planta, el espacio, no tenían listo el espacio. Y tuvimos que reprogramar, un poco a las corridas, tuvimos que reprogramar la llegada de la planta¿no? Fue un tema complicado ahí que tuvimos que resolver también. Sí pero. Entonces el otro tema era que se demoraban en ejecutar ; o sea por ejemplo necesitaban el, necesitaban vamos que… que pongan una losa nueva, una losa donde poner la planta ,o que instalen la red de puntos de oxígeno que te contaba que no tenían, ya. Ahí es donde empezaban los problemas, porque les costaba obtener las aprobaciones internas, decían que no tenían presupuesto, que no tenían a, b o c. Entonces nosotros les pedíamos ,bueno sí pero resuélvanlo ¿no? porque si no hay losa o si no hay (Ininteligible, MINUTO 28:27) a red interno, simplemente lo destinó a otro hospital.Y claro, cuando les decías eso te decían 'no ,no.Sí de todas maneras lo vamos a hacer' pero la hora de la hora nunca lo terminaban haciendo, así que teníamos problemas internos con ellos de parte de la disponibilidad de su presupuesto, del entendimiento de las necesidades ,de recuperación de las plantas de oxígeno y este… también de aprobaciones internas. A veces se demoran mucho en todo el proceso de aprobaciones.

**I: Una cuestión más de gestión del mismo personal que trabaja en la región**

**E:** Sí…sí,sí. No sé si será de gestión de verdad, yo creo que es de capacidades. O sea, no sé si sabes pero un director regional de salud me enteré en esa época podía ganar (Ininteligible, MINUTO 29:16) soles. Entonces la disponibilidad de profesionales que tengan todas las herramientas para solucionar una situación de esta naturaleza no era la mejor

**I: ¿Y usted notó que hubieran mayores dificultades en alguna región en particular o diría usted que fue en todas las regiones la misma situación?**

**E:** En mayor o menor medida era en todas. En mayor o menor medida era en todas, eso sí. No era que te dijera esta región…Bueno a ver las regiones más grandes con mayor presupuesto solían tener mejores equipos, eso sí debo decir. Digamos qué te digo Arequipa, Piura, Ica, pero…que pues un hospital en Huánuco, la región de La Libertad también nos sorprendió pensamos que iba a estar mejor, tuvimos bastantes problemas con Huanuco, Pucallpa, Madre de Dios era ya, era de terror.

**I: OK. Y a nivel de gestión del MINSA ¿Usted notó dificultades para la implementación, recepción de las plantas o tal vez la misma… él mismo no sé manejo del trabajo que se iba haciendo? Una cuestión no sé si de gestión, de recursos humanos, o de alguna institución particular no sé SUNAT, DIGEMID o el mismo MINSA**

E: Perdón ,perdón estaba hablando con el micro cerrado. Te decía, dependió mucho del viceministro a cargo. A ver con el primer viceministro que atendió la pandemia realmente trabajamos muy muy bien; lamentablemente en este tema del 'vacuna gate' estuvo involucrado y dejó el cargo. Pero realmente con él pudimos trabajar bastante bien, con los siguientes viceministros ya no tanto. Dependía nuevamente mucho de las personas. Dependía demasiado de las personas que estaban a cargo, bueno era un proceso humano¿no? Definitivamente eran procesos, eran…gestiones que se hacían todo el tiempo. Así que ahí también venía la limitación principal,

algunos eran buenos gestores y otros no tanto. Igual en las regiones, pasaba exactamente lo mismo, dependía mucho de la capacidad de gestión que tuviera quien estuviera a cargo del área¿no?

**I: Ok. Me gustaría entender cómo era el proceso de priorización de la entrega de las plantas, era como a pedido , era por orden de llegada, por la necesidad de una región que ustedes observan que era mayor que en otra¿ cómo así destinaban o decidían cuál era el destino de las plantas de los respiradores en sí de todo lo que se obtuvo, se gestionó en este periodo?**

**E:** A ver, ahí ¿me copias? Ahí, a ver cómo fue.Ahí, un segundito que me han pedido algo.

**I: No se preocupe**

**E:** Ya, ahora sí. Como te comentaba, como la decisión de decidir dónde iba cada planta era una decisión ultra, ultrasensible. Lo que hicimos fue este… tomar una… cómo se llama, una... creamos ¿cómo le llamamos? Consejo médico, consejo médico de alto nivel en el cual estuvieron los actores, los actores principales de la pandemia¿no? Todas las instituciones que acabamos de mencionar MINSA, Essalud, etcétera más doctores reconocidos que estaban liderando y que eran líderes de opinión de este asunto . Entonces ellos decidían. Nosotros poníamos, decíamos 'sabes que conseguimos'…claro también imaginarás que nos peleábamos a nivel internacional por las plantas ¿no? Entonces conseguíamos una planta y decíamos 'ya la segunda semana de Julio va a llegar una planta de estas características técnicas¿donde debe ir?' Entonces con la información estadística de dónde estaban los casos más grandes, cuáles eran hospitales que tenían las capacidades técnicas para poder recibir la planta se iban asignando. Al principio era bastante obvias las decisiones¿no? Almenara ,Almenara tuvo hasta 3 plantas de Respira Perú, tal vez recuerdas que almenara era el hospital con mayor cantidad de camas destinadas a covid. Almenara tenía la prioridad, enviamos plantas, que está en Grau de Essalud, cómo se llama Almenara y Grau, Grau , Almenara, el hospital este…varios de Essalud, varios del callao también empezaron a recibir plantas. Y luego también ya empezamos a ver el tema de regiones, vimos plantas más pequeñas para Pucallpa, para Iquitos, para diferentes regiones pero eran ellos los que decidían porque sí ,como yo estaba a cargo de la parte técnica, que eso fue una solicitud mía, yo le dije 'miren, discúlpenme pero como está la situación yo no pienso decidir este a dónde va a ir la planta de oxígeno, es demasiada responsabilidad' entonces creamos este comité , lo activé, tuvo sus asesorías técnicas, tenía sus actas y de esta manera llevábamos un registro, el equipo , el grupo decidía dónde. El ministerio de salud tenía una persona de análisis estadístico bastante buena que era ella la que nos daba la pauta¿no? Nos daba una presentación, cómo están las cosa, los mapas, donde estaban los problemas y en base a eso más lo que entendían los doctores ya se tomaba la decisión.

**I: Se tuvieron situaciones críticas, bueno en todas las regiones, pero en algunas fueron como que mucho más evidentes ,como el caso de Iquitos por ejemplo, fue sumamente complicado. Yo tenía la oportunidad de viajar allá un par de veces después y las cosas que he escuchado y he podido informarme han sido realmente muy difíciles,muy muy difíciles. Por ejemplo en este caso de Iquitos, cómo así es que se empieza a trabajar en la zona. Entendiendo que es como un punto aislado entre todas las regiones¿no?** (Ininteligible, MINUTO 36:45) **Una situación diferente más compleja ¿o también un tema de gestión tal vez? No sé si ese fue el caso también allá**

**E:** El problema de Iquitos es la energía, Iquitos no tiene trifásica. Entonces no podíamos enviar plantas de gran capacidad a Iquitos; creo que es más creo que nunca pudimos llevar planta a Iquitos. Justamente por los problemas que tuvo el gobierno ,de las primeras plantas que enviaron fue para allá pero es más fue un sistema y creo que enviaron una planta de llenado de cilindros ,ni siquiera una planta de red que era lo que nosotros proponíamos siempre. Entonces había ese tema¿no? Y a Iquitos enviamos equipos, enviamos insumos, respiradores ese tipo de cosas. Pero ,como te digo, acuérdate también que respira Perú es cierto nos activamos por ahí por mayo… todo estaba con 2,3 meses de anticipación, de espera. Compras no había nada disponible de inmediato, es más los respiradores de un solo uso ,que es un equipo fabricado en Estados Unidos, que supuestamente están en almacenes nos tardaron casi un mes en despachar; y ni qué decirte de las plantas de oxígeno, los repuestos estaban imposibles de conseguir. Entonces lo que conseguíamos, al principio conseguimos empresas que habían pensado en comprar y las comprábamos en el barco inclusive, ya en camino a Perú; esas fueron las primeras. La primera planta que enviamos fue a Arequipa justamente, a unos hospitales grandes de allá, fue la primera que despachamos una planta grande de 60 m3 al día. Entonces ahí teníamos, muchísima capacidad, muchísimas digamos limitaciones para poder despachar. A Iquitos yo no recuerdo que

hayamos enviado planta, creo que al final las limitaciones técnicas de energía impidieron que pueda enviarse una planta, si la memoria no me falla.

**I:Ya,ok. No sabía que ustedes no había intervenido en Iquitos, desconocía eso. Bueno, entiendo que su labor ha sido como más de hacer la compra, la gestión de la compra, facilitar, llevar los equipos ,ya implementar en zona. Y no tienen como una ,digamos labor de supervisión, fiscalización posterior; pero no sé si hay un seguimiento que ustedes están haciendo respecto de las plantas que fueron implementadas, fueron entregadas al minsa, a los establecimientos de las regiones¿saben cuál es la situación de esos equipos ahora? ¿En qué está, cómo están? ¿Aló?no le escucho si me está hablando**

**E:** Ay perdón. Te decía que sí, efectivamente estamos haciendo una labor de seguimiento, es más este… hace unos meses contratamos un servicio de inspección de todas las plantas que habíamos donado. Sabemos el estado, por eso te digo que algunas las están dejando malograr. O sea tenemos un reporte ,el modelo al principio fue un modelo de gestión de uso en la cual las plantas eran de la conferencia episcopal¿no? Obviamente ellos son los que manejaban la parte administrativa. Perdón 1 segundo me está llamando mi jefe, deme 1 segundo y regreso contigo.

**I: Sí, sí. Voy a pausar la grabación, no hay problema**

**--Se pausó por una llamada—**

**I:Ok. Sí me comentaba sobre el tema de seguimiento ,de supervisión**

**E:** Como te digo, como las plantas en la última etapa, lo que estamos haciendo es… hacer que la… cómo se llama… pasar de gestión en uso a donación definitiva.La conferencia episcopal está en pleno proceso proceso y está haciendo ya la donación definitiva a los hospitales, para que ya puedan hacer suya el mantenimiento y todos los diferentes aspectos vinculados a la operación del ¿cómo se llama? La operación de las plantas.

**I: ¿Y para eso están haciendo como una identificación del estado en que se encuentran en este momento?**

**E:** Claro, justamente lo que te decía habíamos contratado un este… una empresa especializada ,que era una de las que nos instaló incluso alguna de las plantas, la contratamos para que pudiera visitar planta por planta, viajaron a nivel nacional y nos hicieron un reporte detallado del estado de cada uno de ellos.

**I: ¿y cuál ha sido el resultado de esta evaluación?**

**E:** Mira, en líneas generales, varias de ellas están han entrado en desuso; o sea han regresado al sistema de cilindros, otras ya sea por ,pero están operativas, otras han tenido fallas y no las han reparado, y era parte del convenio , el convenio establecía que el hospital debía darles el mantenimiento preventivo y correctivo necesario; entonces no lo , no lo hicieron así. Luego hubo este…otras este… y en otros casos, que es la mitad más o menos, todavía la seguían utilizando. Hay de todo, hay de todo. Pero en varios casos estaban en desuso¿no? Un número demasiado alto para nuestro gusto, pero bueno. (Ininteligible, MINUTO 42:32) el tema es que no podemos quedarnos con esos activos ¿no?, como la conferencia episcopal no se puede quedar con esos activos, valen millones. Entonces no…también para ellos... Aparte no tendrían dónde almacenarlos, no. Acá lo importante es que ya queda en el sistema hospitalario, que al final ese es el beneficio; que queden en el sistema, que haya la mayor cantidad de beneficiarios y que puedan tener una vida útil y lo más larga posible.

**I: De las 100 plantas que ustedes hicieron entrega entrega…**

**E:** No, no, no. Motivamos la donación de 100 plantas, de manera directa hicimos 26.

**I: Entonces la supervisión es solamente de estas 26**

**E:** Claro, las que son propiedad de la conferencia episcopal a través de respira Perú

**I: De acuerdo. Y de estas 26 ¿Cuántas están operativas? Digamos en perfecto estado de funcionamiento**

**E:** Mira, queda a cuenta compartir los datos del informe, no tengo los datos en la cabeza pero sí te puedo ,creo que puedo sin ningún problema pasarte las conclusiones de la investigación. Luego ya este… por la tarde

**I: Oh, perfecto se lo agradecería para tener una referencia más precisa de cuántos equipos, plantas están en funcionamiento. Respecto del mantenimiento usted me decía de que, los hospitales deberían hacer cargo del mantenimiento tanto preventivo como correctivo¿conoce las razones por las que no se ha realizado ese mantenimiento?**

**E:** No. En realidad básicamente es por temas de presupuesto y demás.

**I: Presupuesto de los hospitales**

**E:** Sí, o sea no han asignado presupuesto para el, la reparación y aparte algunos hospitales también decían que era por un problema técnico. Desde el punto de vista que como no era de ellos no podían darle reparación, porque no pueden reparar algo que no es suyo. Entonces ese fue otro motivo para iniciar el proceso de donación definitiva, en algunos casos y ya se concretó y en otros casos está en proceso. Pero la conferencia ya desea cerrar ese proceso a la brevedad posible.

**I: De acuerdo. ¿Cuáles usted diría que son las áreas claves que uno debería o deberían concentrarse las políticas sobre el oxígeno? Tanto digamos, considerando todo este… todo lo que ocurrió en realidad. Por un lado está el tema de las dificultades que se tuvieron por otro lado están también, como crisis sanitaria propiamente pero también las dificultades en la gestión, no sé si solamente las regiones sino también tal vez otras instituciones como para afrontar de mejor manera o de una manera adecuada las posibles situaciones similar ,que no sea necesariamente una pandemia puede ser un desastre natural o que se yo, de alguna situación difícil para el país¿no?**

**E:** Pero digamos ¿te refieres a los hospitales en qué situación están para atenderlo?

**I: no sólo los hospitales, me refiero más a nivel de gestión como ,como un institución. Sé que tiene que ver diversos aspectos porque Minsa es como que regula y los hospitales ,los establecimientos son los que operativizan pero en ese marco hay todo entramado de situaciones que como usted ya lo pudo ver…**

**E:** Claro, lo que pasa es que, realmente yo he tenido la oportunidad de levantar la alfombra y y la verdad que encuentras muchísimas ,muchísimas deficiencias. ¿Qué es lo que ocurre? El Minsa, como tú bien dices, regula, da las políticas nacionales de salud. Pero en lo que es hospitales sólo tiene gestión sobre unos pocos hospitales en el país, y principalmente Lima. No ,perdón no, no, de Lima metropolitana, en algunos hospitales de Lima metropolitana; eso lo que tiene gestión directa, que son los menos. El principal gestor de hospitales en Lima, por lo menos, es Essalud. Ellos sí tienen, Essalud tú sabes, un sistema público-privado en el cual a través de los aportes de los empleadores y de los trabajadores, se genera un presupuesto millonario qué es el que se gestionan a través de Essalud y por eso tiene sus hospitales para los trabajadores y sus familias. Y dentro de todo, con todo y sus deficiencias, es el más eficaz de los 2 ejecutores. Entonces… y por otro lado tienes la gestión de los hospitales de regiones a cargo de los gobiernos regionales. Entonces,si bien es cierto tú puedes dar la política pero de ahí para que la política llega la realidad ya implica de los gestores, ya pasa por los gestores; entonces ahí esta es la principal deficiencia, porque tienes como gestores a quienes no necesariamente saben cómo gestionar adecuadamente. Entonces ahí, ahí está la debilidad más gruesa

**I: Qué se podría hacer para resolver esto, este impase, esta dificultad**

**E:** Yo sigo pensando que la descentralización no debería incluir temas cruciales como educación y salud; e infraestructura. Nosotros como institución somos convencidos de que la descentralización tiene que tener una gestión unificada porque tú tienes demasiada dispersión de criterios, de capacidades a nivel nacional, entonces esa es la principal debilidad para nosotros. En educación y salud ,y infraestructura tenemos que tener autoridades o gestores únicos, que sean los responsables a nivel nacional y puedan llevar estándares a todos los hospitales

para que (Ininteligible, MINUTO 48:36) tener un nivel de acceso¿no? Y obviamente con una gestión profesional que puede hacer que se eleve el nivel. Porque realmente en regiones, por lo menos, la brecha es gigantesca y es de terror.

**I: OK. Ya de manera general, por toda la experiencia que ha tenido en este tiempo en el que han venido trabajando el tema¿cuáles diría usted que fueron los mayores retos con respecto al trabajo que realizaron con el tema del oxígeno?**

**E:** ¿Me repite la pregunta? Hay un poco de ruido

**I**: Sí. Le decía que, ya como haciendo un resumen. Por toda la experiencia que usted ha tenido en ese trabajo que ha involucrado el tema del oxígeno¿cuál diría usted que han sido los mayores retos que han tenido que enfrentar?

**E:** La falta de capacidades, la falta de capacidad para atenderla emergencia, la falta de capacidades técnicas para poder implementar las soluciones que se proponían, ese de hecho es la más grande que hemos tenido. Y bueno, también el acceso a recursos; evidentemente, no en todos los casos logramos el nivel de donaciones que hubiéramos querido¿no? Pero es por un tema de coyuntura, por diversos motivos

**I:Ok, cuando me habla usted falta de capacidades es , ¿hablamos nuevamente de el tema de las instituciones en la región o de ustedes también como equipo? Porque es la primera vez creo que tienen que trabajar también en este tema**

**--Se desconectó—Fin de la primera parte**

**I: OK ahora sí vamos a retomar con la segunda parte de la entrevista de hoy que es 30 de junio del 2023. Entonces, habíamos estado conversando acerca de diversos aspectos pero en este momento me gustaría ya como para ir resumiendo ¿cuáles diría usted que fueron los principales retos al respecto del oxígeno medicinal durante la pandemia? Desde su labor, desde la labor que realizó el gremio al que usted pertenece**

**E:** A ver, desde la labor que hicimos nosotros de manera directa… la articulación con los hospitales en regiones. La articulación con los hospitales en regiones fue para mí el reto más importante ,por la baja capacidad técnica y de ejecución de los profesionales de la salud y de los gobiernos regionales con quienes nos tocó coordinar. Después, sentíamos que había una lucha contra el sistema de plantas de oxígeno en general porque en realidad nosotros en algún momento dado compramos cilindros también; que al principio se entendía que era la mejor solución pero luego nos quedó claro que el cilindro no significa nada si no hay oxígeno. El problema era la proveeduría de oxígeno y de esa forma las únicas formas de compensar era a través de sistemas de producción que era algunos de los 2 que te mencionaba, o plantas de oxígeno o el oxi-tanque. Pero el oxi-tanque que también depende de un proveedor que te genere el oxígeno líquido, la ventaja de la planta de oxígeno es que jala aire del medio ambiente, entonces obviamente no tiene un costo el insumo base como tal,que es básicamente el costo de mantenimiento y operaciones de una planta generadora. Pero había mucha resistencia como te digo a ese sistema, entiendo que médicamente no es el sistema más perfecto o en todo caso tienes que tener un excelente sistema de control y demás de la producción para que tengas el producto con la calidad óptima para uso médico. Pero igual sigue siendo una opción este… que cumple con los requerimientos mínimos necesarios si se le hace un mantenimiento adecuado a la planta, eso no está en discusión

**I: ¿Esta resistencia de la que me habla la encontró, la observó usted en los establecimientos de salud, en los gestores o en empresas que producen oxígeno?**

**E:** Yo lo sentía que era un poco, a ver hay un tema que luego de que colgamos me acordé que no te había hablado de tema super importante. Perú en el... Gobierno de Alan García aprobó una norma con una, con un… exigiendo que el límite de pureza sea por encima del estándar internacional, lo cual era un despropósito¿no? Nuevamente, una ley con un estándar por arriba del estándar mundial. Entonces si la memoria no me falla era 93% del estándar mundial y Perú pedía 95, entonces eso había obligado a que no se puedan utilizar plantas de

oxígeno, porque las plantas de oxígeno tienen una producción o sea no hay ninguna planta de oxígeno que te diga 95 te llevan todos a 93 porcentaje de pureza. Entonces eso obligaba a que solamente hubiera 2 proveedores en todo el Perú que pudieran proveer y encima esos 2 proveedores después nos enteramos que habían dividido el mercado nacional , unos atendida en el norte y otros atendían en el sur (Ininteligible, MINUTO 4:16) eso que te digo (Ininteligible, MINUTO 4:20) de oxígeno ¿no?. Entonces no, no había una proveeduría, no había otra forma de proveer al mercado nacional porque no había o sea no había, en el momento en que empezó la pandemia no había cómo proveerle al mercado nacional así como (Ininteligible, MINUTO 4:34) de oxígeno no iba a pasar por las autorizaciones de DIGEMID ni de nadie porque la ley, una ley exigía un nivel de pureza por encima del mercado. Claro , cuando no había pandemia me imagino que todo el mundo contento pero igual aún era un nivel de pureza por encima de los estándares internacionales. O sea, no es la primera vez que pasa esto en el Perú pero en este caso de la pandemia costaron vidas ese error. Entonces, porque la demanda estaba cubierta, no había problemas de… perdón no había problema de oferta. Entonces en el momento en que se dispara la oferta, tuvo que salir una norma especial que durante pandemia se permitía que la concentración baje a 93, lo cual se alineaba con el estándar internacional y de esa manera las plantas pudieran entrar al Perú porque la primera semana no pudieron entrar, entonces este fue un primer cambio que luego entiendo ,te soy sincero habría que revisar, pero entiendo que ya se corrigió esa, ese exceso ¿no? Digamos de… de normativa nacional de la ley, es lo que ya se corrigió que ya hoy en día ya se permiten porcentajes de pureza de 93%¿no? Entonces hay una mejora en regulación que se logró o que obligó en todo caso la pandemia a corregir ese error. Y lo más paradójico era que el ministro que firmó la norma en el gobierno de Alan García, fue el mismo ministro que estuvo después derogarla¿no? Y dijo que lo habían sorprendido. Bueno.

**I:Para entender eso, sabemos, estamos informados de que estas 2 empresas eran las que mantenían el monopolio nacional…**

**E:** Pero el monopolio lo creo la ley, el monopolio lo creo la ley

**I: Sí, sí ,sí yo entiendo, yo entiendo. Con razones que probablemente no conocemos detrás pero en fin. ¿Ustedes en algún momento tuvieron algún tipo de conflicto, no sé con estas empresas por esta apertura, por este… por la intervención que se estaba teniendo para precisamente proveer al país de mayor número de plantas y resolver o digamos apoyar en resolver el tema de la escasez del oxígeno?**

**E:** No, no voy a dar detalles pero digamos que sí hubo en algún momento dado conversaciones al respecto, en las cual es expresamos nuestro desacuerdo con, con la normativa.

**I: Con estas empresas**

**E:** Por lo menos con una de ellas, sí.

**I: De acuerdo, de acuerdo. Quería entender yo sé que ustedes representan a un gremio de empresarios y estas son también empresas, entonces ahí como que podría haber discrepancia, no sé si nivel de diferencia , confrontación pero quería como entender si había habido una situación de esa no**

**E:** Sí, si hubo una conversación en la cual nos pidieron informe creo, nos pidieron información sobre lo que estamos queriendo ser porque evidentemente ,ellos estan… lo que pasó, cuál fue el problema, cuando empezó la pandemia ellos hicieron rápidamente inversiones para ampliar la capacidad de oferta pero nosotros en paralelo estábamos pidiendo que se cambie la normativa para que baje al 93% con lo cual se abría el mercado de plantas de oxígeno, entonces ellos entraban en una dinámica que digamos ponía en riesgo, querían saber hasta dónde ibamos a llegar porque obviamente se ponía en riesgo… el retorno, no se ponía en riesgo he usado mal la palabra, alargaba, iba a ser más difícil recuperar la inversión que habían hecho última hora y como justamente le habían hecho a última hora les había este… le iba a generar… un problema interno pues¿No? Por qué evidentemente habían pedido una suma importante para inversión, habían ampliado capacidad y en ese momento se estaba cambiando la norma, entonces querían saber hasta dónde pensábamos llegar con este apoyo porque evidentemente nos necesitaban como insumo para ver su retorno de la inversión.A las final eso una empresa, no son una… ellos tienen que, finalmente tienen una responsabilidad con sus inversionistas. Entonces ahí sí hubo una conversación en la cual les explicamos el alcance y nuestra opinión sobre lo que hubo en la ley, evidentemente hubo un tema de… todos queríamos que se mejore la pandemia pero evidentemente las acciones

que se tomaron no necesariamente eran las que eran pertinentemente para el negocio pero quedó claro nuestra posición en este tema, el tema era apoyar la lucha contra la pandemia y debimos hacer todo lo que fuera necesario. Y hemos tomado más medidas necesarias, hemos tenido discusiones con más de una empresa porque en su momento dado también propusimos que se priorice la producción nacional de artículos en base a lo que se llama tela no tejida, la tela no tejida es la base de las mascarillas. Entonces si revisas surgió una norma que aún está vigente, que dicho sea de paso estamos pidiendo que la deroguen porque ya no tiene sentido; en el cual la exportación de tela no tejida tenga una priorización para la producción nacional, pensando en atender la necesidad en ese momento que había de la urgencia de contar con ese tipo de productos. (Ininteligible, MINUTO 10:12) la mascarilla, la ropa médica, etcétera,etcétera. Pero, y eso bueno, sí pues generó también algunas discusiones con algunas empresas porque obviamente habían empresas que habían nacido pensando en atender el mercado peruano y otros países de Sudamérica; entonces los obligó¿no? a priorizar, a dejar de atender algunos clientes en el exterior , pero nada al final las empresas estuvieron de acuerdo y apoyaron la iniciativa. Pero sí hemos, ante la decisión tomada si hubieron algunos intereses en los cuales tuvimos que conversar y explicar la posición ¿no? Y discutir lo que había que discutir.

**I:Ok. Pensando en una hipotética, digamos ocurrencia de una situación parecida, similar con la que hemos tenido con la pandemia. Ustedes tomarían las mismas decisiones, en el sentido de 'OK vamos a apostar por el tema de apoyar, para el tema del oxígeno'u esto que me está comentando por ejemplo por las empresas que elaboran los tejidos y elaboran los materiales para el cuidado personal. Lo digo porque ustedes siguen siendo un gremio empresarial Y por otro lado también ya me lo ha comentado están las empresas que quieren todo este trabajo, con la experiencia que han tenido en este tiempo de pandemia ¿cómo se vería , cómo afrontarían, cuál cree que sería la posición de su gremio?**

**E:** Ya, lo que pasa. Lo que tenemos que tener en cuenta ahí, hay una… entiendo la posición, yo también estaría parecido a ti. Pero hay una variable que tienes que considerar, finalmente cualquier posición que tome cualquier gremio empresarial, cualquier iniciativa que impulse un gremio empresarial siempre va a haber alguien que gane y alguien que pierda; por economía simple, si promueves una ley que reduzca la anemia en niños, las empresas de golosinas van a verse afectadas. O sea cualquier norma que tú quieras impulsar por más buena que sea va a haber gente que va a ganar y gente que va a perder, o va a a ver a tener que reacomodar su negocio para que adecuarse a las nuevas normativas, es normal. Pero no por eso las instituciones dejamos de impulsar iniciativas que consideramos son beneficiosas para el país. En general ese es ética, que consideramos el actuar correcto, hay que tomar las decisiones que sean correctas para el país en el momento que tengan que hacerse y evidentemente va a haber empresas que tengan, es más, con esas decisiones que tomamos hay empresas que fabricaban mascarillas y han crecido 8 veces. O sea, me entiendes al final no podemos, no podemos hacer que nuestras decisiones dependen de quién gana o quién pierde, eso sería un error. Entonces como gremios empresariales tenemos claro que tenemos que impulsar principios, tenemos que impulsar medidas que sean a favor del país en el tema de desarrollo económico, desarrollo social y en este caso de temas de salud o educación¿no? Y sabemos, somos conscientes que hay empresas que se van a beneficiar y otras que no tanto, y con las que no se van a beneficiar bueno les tenemos que explicar la situación y más bien ver cómo manejamos con ellos el tema de minimizar, ayudarlos a que minimicen el impacto pero no por eso vamos a cambiar decisiones necesariamente, o sea no. Y escuchamos a todos y podemos hacer ajustes para que la propuesta sea lo más beneficiosa posible en todos los aspectos, pero somos conscientes nuevamente de que cualquier iniciativa va a haber empresas que se van a beneficiar y otras que no.

**I:OK. Como usted ve a los diversos actores involucrados MINSA, Sunat, gobiernos regionales, Respecto de las dificultades que se tuvieron para afrontar todo es este, digamos no solo la implementación de las plantas sino también desde el inicio de la importación hasta ya propiamente este momento en el que deberían estar haciendo mantenimiento, deberían estarse poniendo en funcionamiento o habilitándose la infraestructura de las instituciones para que se sigue dando uso a las plantas. Entonces ¿usted percibe que las instituciones involucradas o las entidades involucradas han mejorado, han cambiado en algo, han aprendido algo?**

**E:** Qué difícil tu pregunta, qué difícil tu pregunta estoy pensando al final que te respondo.

**I: Dejé las preguntas más difíciles para el final**

**E:** Mira, a ver te soy sincero. Yo ya evidentemente, ya no estamos viendo el tema de oxígeno de manera directa porque fuimos una solución temporal, un apoyo temporal… pero digamos, a ver durante los 18, 20 meses que hemos estado operando este tema a tiempo completo, lo que sí me ha preocupado es la rotación de la gente; entonces no se ha generado, o sea si la gente hubiera estado (Ininteligible, MINUTO 15:40) de la pandemia y se hubieran quedado yo tendría la tranquilidad qué esta gente ya sabe cómo actuar en caso ocurra nuevamente. O sea a mí me ha tocado hablar con gente, para instalar una sola planta, a hablar con 3 diferentes gerentes de desarrollo de salud, directores perdón, directores de desarrollo de salud en un gobierno regional para la misma planta; o sea te estoy hablando de 1 año para instalar la planta que eran 90 días, de 60 a 90 días. Entonces eso sí me preocupaba demasiado , porque significa que la gente que hay ahora estoy seguro si es un análisis, un porcentaje mínimo de la gente que esta hoy día cargo de las responsabilidades del sector salud en las regiones ha estado en la época de la pandemia y tiene una idea más o menos cercana de que es todo lo que implica. Entonces es nuevamente, en donde puede ser un poco mejor la situación es en las reguladoras¿no? En Digemid, sí debe estar la misma gente. En Essalud también ya cambió, la gente con la que más trabajamos fue en la época de Fiorella Molinelli, bueno independientemente de lo que ocurra en el caso particular de ella como persona era un excelente gestora; Entonces resolvimos todo muy rápido, cuando ella salió sí notamos algunos cambios porque ya no había la misma dinámica pero igual sobre todo hay un equipo técnico que en cierta medida se mantiene. Sobre todo en la parte de los hospitales, esa parte no cambia mucho. Pero, o sea la pérdida de espacios, la pérdida de conocimiento adquirido, tan poco tiempo, es terrible. Y a veces me ha pasado, hace poco tuvimos, tenemos todavía una planta que tenemos que reubicar porque la persona,la institución que la iba a tomar por diversos motivos ya no lo estaba tomando y nos dimos cuenta que ya no conocíamos a nadie, o sea llamábamos a todas las personas con las que habíamos tenido contacto y ya nadie estaba en donde estaba. Entonces fue muy, fue un trabajo adicional antes era muy rápido porque estábamos en pandemia, levantabas el teléfono, hablabas con fulano, coordinaban, sacabas adelante y seguías avanzando. Ahora ya no podíamos hacer eso, entonces eso sí me preocupó ya como persona, como profesional porque evidentemente formar a una persona y que sepa lo que tiene que hacer es lo más compleja en estas situaciones¿no? Es lo más complejo en estas situaciones. Así que nada pues, entonces, eso sí preocupa, preocupa bastante

**I: Usted me comentó acerca del informe de supervisión del estado de las plantas con las que han, digamos han estado entregando o instalando en las regiones; pero entiendo que han habido más, más plantas. Si se diera una pandemia ahora¿cuál sería la situación del oxígeno en este momento?**

**E:** No sabría decirte, sinceramente. Es una respuesta que no ,no tengo las herramientas pero lo que sí hemos sentenido ,lo que sí te puedo decir es todo lo que te comentaba hace un tiempo ¿no? Un retroceso en el uso de plantas de oxígeno y regreso a los cilindros con lo cual se configuraría, si todas las plantas nuevamente se malogran ,ahora ya no serían 11 sino 100 plantas malogradas en los hospitales del Perú. Entonces sí, sí habría un retroceso en la política de uso de plantas de oxígeno, lo que va a terminar pasando es eso. Entonces vamos a podríamos nuevamente ante un pico de demanda como ocurrió en la pandemia, nuevamente podríamos tener un desabastecimiento que fue lo que generó la mayor, bueno no la mayor pérdida pero una buena parte de las muertes fue justamente por él desabastecimiento de oxígeno medicinal en las cantidades necesarias. (Ininteligible, MINUTO 19:26)

**I:Usted como digamos cabeza, como persona que impulsó este tema o estuvo más presente en el trabajo de coordinación y gestión en el tema de oxígeno por parte de su gremio¿qué lecciones claves podría compartir con respecto al trabajo que realizaron como gremio, como institución y también como grupo, como parte de este consorcio, de este grupo que trabajo precisamente para respira a Perú?**

**E:** A ver… primero me pareció muy positivo el hecho de contar con, con un equipo… con digamos… con la iglesia, con la conferencia episcopal, cáritas que también fueron las 2 entidades de la iglesia católica que operaron con nosotros en una institución como la nuestra y el lado educativo. O sea es, de manera tripartita y cubriendo diferentes frentes, realmente el trabajo que se pudo hacer fue de primer nivel. Cubrimos muchas de las necesidades pendientes que tuvo el país en ese momento, la iglesia católica no es por nada pero no por gusto es una institución con más de 1000 años de existencia realmente, con todo y sus procedimientos, y sus controles¿no? Que toda organización grande tiene pero realmente bastante eficaz y realmente fue súper importante el poder contar con ellos como parte de la estructura de organización. Luego, el tema de las empresas realmente muchas respondieron positivamente de una u otra forma para poder este apoyar , o sea me quedó claro cuando las empresas ven una necesidad real salen a apoyar o también, no era la primera vez ¿no? También ha

habido momentos de desastres naturales de gran envergadura en la cual también hemos sentido una participación activa de parte del sector empresarial. Pero en este caso en particular fue bastante activo o sea fueron 16 millones de soles, no es un monto menor y en realidad, el otro tema también es… el costo de ineficiencia del estado, o sea nosotros con 16 millones de soles, que es una (Ininteligible, MINUTO 21:56) para lo que es el estado y para los miles de millones que se invirtieron en las pandemia, logramos mucho más en ese tema que otras instituciones con mucho más dinero. O sea después nosotros con horror veíamos que algún gobierno regional estaba comprando una planta que nosotros sabíamos que no costaba más de 200 mil , 240 mil dólares la compraban en 1millón. Y te digo son casos reales, o sea no te contesto pero inclusive llevamos, llevamos la inquietud a niveles de contraloría para que revisen esos procesos porque en realidad ya era un escándalo, eran cosas que no tenía nombre. Pero es la ineficacia del estado para poder ejecutar¿no? Nosotros éramos 2 personas del SNI, o sea digamos yo que dediqué gran parte de mi tiempo a este tema más una personas que era mi coordinador que estaba 24 por 7 en ese tema, por ahí 2 personas más de logística que las activábamos cuando era necesario, alguien de la conferencia episcopal y listo, vas parando ,vas parando de contar. Y fuimos los que vimos la compra de las plantas, la (Ininteligible, MINUTO 23:07) de los equipos, la importación, hubo una persona de cáritas también del área logística de cáritas extremadamente eficaz. O sea con poca gente y gente clave que sabía pudimos hacer muchísimo para apoyar esto, este tema de Respira Perú en diferentes partes del país. Ahí nos dimos, fue una prueba clara de que el estado no, lamentablemente tiene una ineficacia muy alta y que es un costo que pagamos finalmente todos los peruanos; eso fue también otra lección. Y también,y por último entender que aún en situaciones extremas, la parte de decisiones en el estado es demasiado lenta y a veces hasta indolente con los procesos. O sea, el tema de cómo se han hecho tan rígidos los procedimientos de compra, adquisiciones hacen que la gente no quiere firmar nada, o sea el tema del control…

**I: ¿Se refiere a los funcionarios públicos?**

**E:** Así es, no quieren firmar nada, no se quieren comprometer a nada por miedo a las auditorías posteriores. Eso también es un costo, un costo bien alto que estamos pagando como país¿no? Al tener un sistema que ha hecho que se paralicen; y yo, yo he sido funcionario público, yo también tenía algunos millones de presupuesto anual y tenía que colocar pero en realidad a lo que es hoy día es mucho más complejo. Y yo ah, que era inocuo porque era una institucion de promoción, yo trabajaba en Promperú hasta 2 años después se recibían consultas sobre un cóctel que habíamos hecho trayendo compradores internacionales a un evento en Perú, un evento para artesanías y auditoría me preguntaba si yo había contado cuántos bocaditos habían salido de la cocina. O sea a ese nivel, entonces imagínate cómo sería para otros procesos más, más complejos. Entonces sí, hay un costo de yo le llamo el costo de la desconfianza que se ha generado en el país; entonces es un tema álgido que no podemos dejar de tener en cuenta como país para, pensando en el largo, en el desarrollo a largo plazo.

**I:Ok, bueno yo no tengo más preguntas en este momento pero no sé si por ahí hay algún comentario más que quiera hacer que yo no le haya preguntado y que usted considere que es como importante para el estudio**

**E:** Yo creo que está… bueno, el tema de las compras públicas. Hay un tema que tú me preguntaste pero con el comité médico que te conté que habíamos creado había una preocupación permanente de los doctores , que era como lograr que haya producción nacional de estos productos que consideraban de vital importancia para el país, que no podíamos dejar de tener. Pero como el Perú ha tenido desde hace unos años una política de apertura comercial muy grande,este… la prioridad ahora es, solamente es el precio entonces lamentablemente no necesariamente tenemos el precio más bajo como país porque pagamos, tenemos nuestra mano de obra es de las más caras que hay en la región, nuestro sistema de infraestructura logística es terrible, entonces nunca lo vamos, no vamos a lograr tener los precios muy bajo. Pero sí tenemos una buena relación calidad precio, pero no te compran por calidad precio, te compran por precio. Entonces apenas pudieron, el sector público este… dejó de comprar mascarillas, hoy en día las fábricas de mascarillas venden en Bolivia, venden en Puerto Rico, venden en Estados Unidos, venden en Colombia pero no venden ni una mascarilla de Perú. Entonces los médicos me decían¿cómo hacemos para que haya esta producción?¿Que hay que hacer?¿Por qué no invierten en plantas para que haya producción nacional? Yo les digo el problema no es que inviertan, el problema es que hay un mínimo de compra asegurada porque si van a invertir en una planta para que acá en el Perú no les vendas nada, no tiene sentido. Entonces, lo que hay que hacer es motivar algunos sistemas para que algunos productos, algunos servicios, que sean considerados sensibles, que sean considerados estratégicos para la salud pública se asegure un mínimo de compra nacional; o sea no te digo que compren mal, pero que compren, que compren, que exija

a un nivel de calidad y si el producto cumple ese producto a un precio competitivo; bueno proveete localmente. Entonces… era donde yo le pasaba un poco la pelota a las personas del Minsa y de Essalud que estaban ahí, les digo o sea ustedes tienen que proponer esto está bien, si quieren que haya una industria local que provea EPPs , etc¿no? Las líneas de protección personal, que (Ininteligible, MINUTO 28:05) que haya ciertos tipos de equipos, fármacos, maquinaria; tiene que haber un mercado porque si no a quién le van a vender. Si no(Ininteligible, MINUTO 28:14) se tiene que trabajar en ambos sentidos. Entonces sí, nos decían 'sí bueno tenían razón, es importante, ya vemos cómo hacemos' pero nunca se movió nada de eso. Entonces yo creo que si ustedes hablan con el sector médico que es quien está en la cancha, ellos sí consideran que debe ser estratégico poder contar con oferta local para poder atender emergencias cuando sea necesario¿no? Cosa que es algo que (Ininteligible, MINUTO 28:40) no tienen ¿no?

**I:Ok, le entiendo. Bueno muchas gracias por su disponibilidad, sé que tiene una agenda súper súper compleja pero muchísimas gracias por apoyar en el estudio.**

**E:** Gracias más bien a ti por la paciencia

**I: No ,no.Entiendo no se preocupe, voy a dejar de grabar en este momento entonces.**

**007 Entrevista**

**Meeting started: 01/07/2023, 11:05**

**Participantes: E, I**

**I: Investigador**

**E: Entrevistado**

**E:** Ok, respecto a las políticas pertinentes. En cuanto a los servicios de oxígeno medicinal, **¿hay alguna pública orientada hacia eso?**

**E:** Bueno, nosotros, en principio, como dirección de gestión de riesgo, desde nuestro campo que era el velar por necesidades que pudieran presentarse en emergencias y desastres, y, de hecho, en el plan nacional de gestión de riesgos están las heladas y los friajes propusimos dentro de la política nacional, el cambio del porcentaje bajo el cual 1 podía obtener como fuente de oxígeno principal, no 99, sino 93%, porque no todos los pacientes requieren 99% porque a nivel nacional estas heladas y friajes generaban un impacto importante en los extremos de la vida el impacto del manejo la neumonía y la distribución de oxígeno en los establecimientos de primer nivel era por demás, deficiente.

Entonces, la política de cambiar este porcentaje de 99 a 93, que luego fue una vedette en la pandemia lo propusimos nosotros desde la dirección de gestión de riesgo en el año 2018 propusimos el cambio en virtud de poder adquirir generadores de oxígeno. Concentradores que pudieran apalear la necesidad de ese oxígeno para los pacientes que se complicaran y poderlo referir con seguridad en ambulancias con equipos portátiles hacia los establecimientos de mayor nivel, porque nuestros establecimientos, primer nivel no tenían ni el horario ni el personal para manejar lamentablemente aún sean casos no tan complicados a estos pacientes.

Entonces, la primera política que nosotros propusimos cambiar y nos costó muchísimo, la DIGEMID puso miles de obstáculos, por decir, una manera: sustentamos que hasta en los documentos de la OPS establecían que el funcionamiento de estos concentradores podía ir inclusive con un rango de 88-90 porque no todos los pacientes necesitan 100% de oxígeno de hecho, el oxígeno es tóxico cuando lo había más de 60% por más de 6 h.

Entonces, con ese sustento de la OPS con el sustento de que queríamos manejar pacientes de neumonía grado inicial no complicados, no en uci, nace el concepto de sustentar lo que en el 2017 la gestión había solicitado y no se concretó que fue los concentradores de oxígeno ya el 2018 no se enfrentamos a otro gran problema para implementarlo, para poder implementar una política, tiene que estar escrito digamos, a grandes rasgos y en las políticas de salud en aquel entonces no había esclarecido una política relacionada específicamente para emergencias y desastres. Lo único que teníamos era la política que se firmó en el 2017 de **hospitales seguros** que luego encima fue cuestionada porque a nivel de CEPLAN el formato de la

política fue cambiada y esa política que hablaba de reducir la vulnerabilidad de los hospitales y específicamente el tener provisiones de oxígeno prácticamente se quedó.

Nosotros la seguimos difundiendo, desde luego, pero desde el punto de vista del más alto nivel, fue sujeta a cuestionamiento y nace en el medio de esto casi para el 2020 recién la política **Perú país saludable** en donde incorpora el concepto para emergencias y desastres, pero no de la manera tan específica como lo habíamos propuesto nosotros en el 2017 política aprobada, por supuesto, de hospital seguro no entonces en esa política hospital seguro si habla que los hospitales tienen que tener un índice de oxígeno obligatorio medido anual y tiene que velarse por la cantidad de oxígeno y una cantidad específica para las primeras horas llegaba un desastre.

Por lo menos teníamos el sustento y ya después, en el año 2 018 tuvimos aparte de la DIGEMID que no quería que compráramos a nivel particular por ese quiebre del 99, 93% que proponemos. El segundo, los equipos que se pidieron en el 2017 como parte de la operatividad de reducir la vulnerabilidad en heladas y friajes no estuvo en el plan del 2 018 no estuvo por qué justo yo un periodo salgo del ministerio, retorno a mi hospital. Y en ese pequeño periodo lo sacan del plan, lo que habíamos planificado para gestionar, concentradores de oxígeno lo retiran y por qué lo retiran. Lo retiran. Porque ¿cuál fue el sustento técnico que dio la gente de la dirección que dirigía ese tiempo? Lo que pasó fue que, como en el 2017, no se logró y fueron sujetos de bueno, cuando no haces algo te juzgan, cuando haces porque lo haces en este caso, hicieron una serie de documentos desde la defensoría desde la contraloría porque el MINSA no cumplió con la compra de generadores en 2017 y como todos se asustaron en 2018. no lo colocaron. Entonces, eso fue una segunda.

Tenemos la primera traba de que no podía comprar siendo competente para comprar por mis funciones de ROF lamentablemente, también tuve la segunda traba que el colega que estuvo tan solo 3 meses en el cargo lo quitó de la planificación del 2018. Entonces, y ya estando aprobado un plan con resolución ministerial como usted comprenderá, era difícil, pero sí lo logramos hacer, hicimos un informe técnico. Se contrató un ingeniero especialista y con el equipo de la DIGER, se retomó el tema, se presentó porque si nosotros seguimos dilatando ya usted sabe que del estado no está 1 o 2 años y se pierde la oportunidad, se hizo la gestión se incorporó para el siguiente y se comenzó la gestión de los generadores.

Para eso, la DIGEMID nos aprobó esa subsanación de tener 93% para estos generadores y para el 2019 el MINSA a través de la dirección ya tenía concentradores, ni siquiera de 10 de 20, porque yo, como intencionista, sabía que 10 era nada y que 20 tenía una mayor capacidad.

Entonces eso fue la gestión que se hizo en cuanto a la política de mejora de la oferta de oxígeno, pero pensando más en heladas y friajes con una cantidad de requerimiento menor de oxígeno lamentablemente cuando vino la pandemia se malinterpretó el concepto y se pensó que los concentradores pues eran la panacea. No, lo fue más para casos leves y paliativos, porque los pacientes en sí requerían estar en un área hospitalaria o acondicionada para tal como una cantidad una fuente de oxígeno permanente Entonces ahí. Tuvimos un problema de concepto y para el 2020 que yo todavía seguía en la dirección de gestión de riesgo, se propuso un plan, un plan de fortalecimiento basado en que en lo que le comencé a decir la política de hospital seguro todos los años teníamos un registro de ese índice de vulnerabilidad basándome en ello, se hizo un plan que también solo se ha alcanzado la distribución de plantas de oxígeno, concentradores, balones, insumos en general, para la dotación de oxígeno esto fue trabajado por el equipo técnico y liderado por mi persona en la calidad ya de especialista de manejo de pacientes.

E hicimos ese plan, no este con la data que teníamos al momento y seleccionando hospitales que tenían en ese momento con tanta capacidad, porque no todos tenían la capacidad ni los recursos humanos ni el

espacio ni nada pero prometo 0, sabíamos muy bien qué hospitales les podían dar la talla basándonos en eso hicimos este plan y también pusimos establecimientos de primer nivel, aquellos a los cuales les habíamos distribuido estos generadores de oxígeno y que de alguna manera podían servir de apaleamiento principal en el momento cero. Bueno, todo este plan no fue tomado en cuenta lamentablemente, este plan lo presentamos de manera ya este virtual en aquella época ya comenzó, pues el tema de la virtualidad y a pesar de que este teníamos ese problema nosotros cumplimos con dárselo a la dirección de operaciones del MIONSA que tuvo 2 momentos tuvo 2 directores también en aquel momento está un determinado director luego cambió.

A los 2 les presentamos el plan. El plan está costeado más o menos en cerca 200 millones de lo que salió en aquel momento en el MINSA en paralelo sin haber sido presentado a la dirección de riesgo, porque todo lo hacía el comando, nosotros no sabíamos de esa plata yo me entere por las redes. Ese plan y en paralelo, yo ya había presentado unas semanas antes el plan de nosotros. Entonces yo, cuando vi, dije: dudo que en una semana haya leído mi plan y hayan sacado algo de ahí y efectivamente, ese esa resolución de oxígeno salió solo para balones ni siquiera insumos y presupuestado en 80 millones y como comprenderá, mi equipo y yo sentimos una gran frustración porque todo el trabajo y toda la evidencia que teníamos de cómo manejar de una manera técnica este asunto fue prácticamente desestimado y en esas reuniones relacionadas a concentración de oxígeno tampoco fuimos convocados. Entonces eso digamos fue para la primera ola.

**I:** Doctora, por lo que usted me va diciendo entiendo que ha habido como momentos en los que digamos, ustedes tienen propuestas, han revisado, han observado las dificultades que hay en campo y en función a eso hacen propuestas; propuestas no solamente para para una normativa tienen también desarrollo en todo un plan, pero no se aceptan o se postergan**. ¿Cuál es la, no sé cuál sería la motivación por la que esto no se resuelve?** Estoy como tratando de entender el **por qué pasan esas cosas es una cuestión más de personas de desconocimiento o no sé qué cuál que está detrás de todo esto.**

**E:** Pues, vayamos primero a la estructura. A me gusta en cuestiones de emergencias y, sobre todo, desastre de esta magnitud, tratar de estructurarlo de la siguiente forma no, yo considero que tienen que haber sin 5 sistemas operativos eso es, digamos, algo de mi experiencia, que tienen que estar coordinados todos para poder manejar de acuerdo una emergencia.

El primer punto es el sistema, ¿no? Y el sistema aquí estaba, por decirlo una manera era un sistema había sistemas paralelos. No había un orden en el mando, estaba el comando, por un lado, estaban las direcciones, quizá, por otro lado, estaban los asesores con el ministro por otro lado, y había en la coyuntura política de la PCM, no había una verticalidad, no se sentía una verticalidad, yo he pasado emergencias en el MINSA, digamos el fenómeno del niño 2017, síndrome Guillem Barré, que también fue una emergencia que tuvimos, que se prendió y tuvimos que manejar rápidamente que fueron las 2 grandes digamos que yo podría decir; una a nivel de sobre todo poblacional y más de salud pública, y la otra más a nivel de hospital y en ambas. yo sentí que había verticalidad, que había un mando que había alguien que tomaba decisiones y que todo estaba de alguna manera orquestado, sin embargo, en la pandemia no se sintió eso. Es decir, no se enteramos de las decisiones por correo o por la resolución que salía y las mismas direcciones se quejaban, la verdad, ¿Y cómo nos damos cuenta? Porque nosotros, en la dirección de gestión de riesgo, tenemos la responsabilidad de justamente asesorar en temas de emergencias y desastres a la alta dirección y hubo un quiebre en la comunicación no se nos permitía eso tenemos que tratar con otras personas, personas que no tenían el perfil, claramente no tenían el perfil, ¿

Y a pesar de que el Ministro de aquella época, el doctor Zamora, las veces que pudimos acceder a él, por una parte, decía: sí, necesitamos que la DIGER participe a la hora que nos juntamos con el equipo que él había nombrado no había una escucha activa.

Entonces ese era un quiebre importante entonces, en el sistema, había, como le digo, se percibían los sistemas en paralelo y no verticalidad. La PCM, por un lado, los asesores del ministro por el otro, el tema del comando y las direcciones, por otro.

Las mismas direcciones acudían a mi persona, los equipos técnicos, sobre todo porque ahí había otro problema. ese es un primer nivel del sistema y dentro del sistema los subsistemas, que es la dirección las direcciones consumen con sus propios equipos técnicos, es decir, muchos directores, sentían la presión de sus equipos y digamos, a veces se percibía que a sus espaldas de los equipos técnicos, tomaban las decisiones con algunas personas probablemente, porque los equipos técnicos acudían a mí comentándome que no sabían nada de lo que había efectuado o firmado su director entonces ese era había un quiebre en el sistema, el sistema de comando de incidentes, que debería implementarse de acuerdo a la ISO 2320 pues lamentablemente no tenía una verticalidad como debería haber tenido el. Segundo problema en cuestiones de organización ah bueno, y dentro del sistema el subsistema tiene que tener un seguimiento informático o sea todos debemos manejar la misma información. Otro quiebre. El comando utilizó la data de SUSALUD para la gestión de camas, también. Y también, por otro lado, DIGEMID generó en el tiempo ya un sistema para lo que es específicamente el oxígeno, que hasta ahora es muy bueno, pero en esa época todavía no se tenía se estaba construyendo solo entendía la gestión de camas esta información era de SUSALUID o sea les dio la malla informática para que la utilizara el MINSA, pero esta información no la tenía la directora personal de salud, ¿la dirección de gestión de riesgo y una serie de actores? no la tenían.

Entonces fue complicado manejar en ese aspecto esa parte importante de la gestión de información, entonces en nosotros, por otro lado, manejamos el Centro de Operaciones de Emergencia, a mí me traía información los Gobiernos Regionales, directamente, los atendidos los y claramente teníamos una información que no era la información del Ministerio, nuestras cifras eran sobradamente mayores, insistimos de manera directa, acudimos al comité donde estaba el comando el comando de la Dra. Mazzetti a través de correos, a través de documentos remitimos información, como DIGER, DICOE estableciendo que no había una concordancia entre lo publicado la información pública de lo que nosotros recibíamos de regiones hicimos reuniones ZOOM con el equipo comando para explicarles esta divergencia y que se pusieran de acuerdo porque no teníamos una información única y la población, evidentemente lo percibía. Tampoco se nos tomó en cuenta tampoco hubo una, digamos, un seguimiento de ello y quedó, pues, eso, digamos, por decirlo empaquetado, no fue tomado en cuenta entonces esos son los problemas que tuvimos a nivel del sistema.

El segundo problema a nivel de gestión del espacio a nivel de gestión en lo que compete específicamente oxígeno, para poder tener oxígeno teníamos que tener ciertas condiciones para empezar, energía eléctrica, condiciones de limpieza, condiciones de seguridad y condiciones de lugares establecidos que se suponen tanto simulacro los hospitales tenían que tener. Obviamente, esto nos agarró en mal momento, porque pocos hospitales eran los que tenían esa información, pero la forma de sistematizar esa información nosotros sí la teníamos a través ISH. Y esa información en lo que es la gestión del espacio, tampoco fue utilizada por más que también se les dijo, nosotros cumplimos con remitir planos de hospitales seleccionados para manejo del covid en el momento cero explicando que ahí podían ir modulares o tiendas porque era la necesidad en ese momento y cómo poder este resolver el problema del espacio. Entonces problema del espacio fue un problema serio, no un problema que se tenía información, pero no se utilizó de la mejor manera y fue difícil también porque aquí en la gestión del

espacio que es parte de la logística intervino legado debido a la falta de respuesta inmediata que pasó, el Seguro social propone los modulares que ustedes conocen y fue asumido por la PCM como la hoja de Ruta, para lo que es la construcción de estas infraestructuras que sabíamos además, tenían un tiempo de estancia corta.

Ya prácticamente el Ministerio, lo que tenía que hacer y lo que se ordenó en aquel momento era que el ministerio trabajara con ese equipo de ESSALUD y Legado ya los temas ya prácticamente se delegó en otras instancias, el tema de la gestión del espacio.

El tercer punto era el tema de la gestión de los insumos. También tuvimos problemas porque no se tenía el concepto escalonado de planta, concentrador, valor, medidor de presión y los diferentes equipos e insumos que se tienen para dar oxígeno llámese canona, máscara de reservorio, entonces cuando se pedían las cosas se pedían de manera asimétrica, es decir, solo balones, como vimos en la en la propuesta, que realmente es a mí me daba vergüenza, porque yo decía mis colegas que son intensivistas y médicos, dirán, caramba, tú estás en el ministerio, y haz avalado esto, no pero yo ni siquiera había participado de eso no entonces esas deficiencias se vieron en cuanto a la gestión de los insumos. Ya luego esto pasó a ser un requerimiento a nivel de PCM con el gran logístico que fue Legado y ya nosotros pasamos un paso al costado y éramos simplemente dadores de opinión, bueno, la DIGER no, los demás integrantes del ministerio.

El cuarto punto era la gestión de recurso humano, otro error acá sólo se pedía médicos y a las justas duras, penas enfermeras cuando necesitamos ingenieros y técnicos biomédicos, a pastos era la oportunidad de oro, de poner tas con tas las deficiencias de los hospitales en estos especialistas. Es decir, hay hospitales del tercer nivel de que no tienen especialistas, que no tienen ni siquiera un técnico en las 24 h que solucionen los problemas de la planta de oxígeno la casa fuerza, se va en la presión de oxígeno y bueno, se fue pues no, o sea, no se tiene. Y cuando se hizo la gestión de los recursos humanos, tuvieron omisiones de batallones que debieron estar aquí para tener información y equipos operativos, ingenieros biomédicos técnicos biomédicos, ingenieros de sistemas hasta bachilleres porque, así como entraron a taller, estudiantes y residentes tenían que entrar esta gente para ayudarnos a manejar la información en tiempo cero del oxígeno no se sabía cómo se estaba manejando, la cantidad algunas regiones lo lograron hacer otras no.

Algunos, el sistema alguno con Excel otro a mano a lápiz y papel. Es decir, ahí nos faltó esa visión de que el Staff es completo no es solamente que el médico va a solucionarlo todo y es una falta de visión de la necesidad de la logística que se requiere en la parte de emergencias en la parte de plantas también hubo un problema porque por alguna razón eso lo digo porque yo asistía a las regiones había una gran desesperación porque vinieron las plantas y las plantas de iban a demorar 3, 4 meses, 3, 4 meses que ya nos agarró la segunda ola y toda la primera ola fue con balones que se cargaban por vuelos en eso sí puedo dar fe que hubo de alguna manera una respuesta inmediata eso sí me consta que un equipo del MINSA, por lo menos por lo menos, se organizó para poder tener esos vuelos de apoyo con las idas y venidas de los balones hasta que se pusieron las plantas pero cuál fue el problema ahí que no se pensó en alternativas o si se pensó no había porque había escasez de todo

Si nosotros hubiéramos hecho caso a la compra de concentradores de oxígeno y aquí aparto un momento no en esto de la que la gestión de los insumos, si hubiéramos hecho caso en la gestión aprobada por el MEF de presupuesto que nos dio a nosotros adicional ni siquiera presupuesto ordinario, el MEF me aprobó un presupuesto para adquirir 80 concentradores de oxígeno, ya no de 20, de 30 porque ya teníamos otra visión para heladas y friaje, pero eso lo anularon lo anularon para comprar ventiladores mecánicos. Bueno, otro error que hubo por ahí, ¿no?

**I:** Error. Perdón, error previo a la pandemia.

**E:** Así es, en plena pandemia eso fue como yo lo gestioné el 2019, la plata que iba a tener para el 2020 enero. febrero, marzo, el 31 de enero sale el primer plan de preparación para el Covid y en febrero del 2020 enero, perdón del 2020 ya ese presupuesto que el MEF me había aprobado a mí para comprar concentradores automáticamente pasó para adquisición de ventiladores mecánicos.

A pesar que se defendió de que necesitaba, porque la idea era tener el degradé de oferta de oxígeno, plantas de oxígeno, concentradores de 300 litros que se hacían en grandes campos y concentradores de 30 ya ni siquiera de 10 y 20 para realmente dar la talla en ese grueso de pacientes de moderada complejidad y leve y evitar que vaya, pues, a la parte crítica y lo que se hizo fue fortalecer los extremos, fortalecer plantas de oxígeno en hospitales y fortalecer 10 litros que era para pacientes leves y todo el batallón del medio todo el batallón del medio se quedó en nada, entonces eso fue digamos en la gestión de insumos los problemas que se tuvieron otra cosa en las plantas de oxígeno.

**I:** Sí perdón. Antes de pasar a esto, una me queda una inquietud: **¿Cuál considera usted que ha sido las razones que llevaron a tomar esas decisiones?**, por ejemplo, hacer este cambio en la compra, en fin, todo esto que usted me está relatando es como decisiones, pero que nos lleva a tomar esas decisiones o que llevo a eso en esos momentos a las autoridades a hacerlo porque la información entiendo que la tenían.

**E:** Bueno, de manera no lo podría decir de manera enfática, porque puede ser presunciones nada más. ¿No? En primer lugar, como le dije, la falta de tener un sistema vertical, no tener la data correspondiente ni la información correspondiente de lo que se estaba trabajando a pesar que esto que lo he comentado usted lo comenté a todas las y de las cuales este tenía o estaban a mi cargo, el segundo es no tener la confianza en el equipo técnico probablemente la visión que tienen en general la política peruana y cuando hablo de política, habla de la política buena, porque la política debe influir para hacer bien en la población, no los conceptos errados que tenemos nosotros pero la política, lamentablemente, se fía de personas que de pronto pintan más canas o pueden tener más títulos o de pronto, pueden ser más reconocidas a nivel, digamos, del país o hasta se podría decir internacional pero había poca confianza en sus equipos técnicos que para eso estábamos entonces no hubo esa confianza.

Yo percibía como que haya muy bien déjalo gracias, pero por otro lado, se tomaban decisiones con otros equipo como lo dije, no en la parte de los sistemas. Por otro lado, toda emergencia grande, las decisiones no las toman ministerio, las decisiones las comenzaron a tomar al más alto nivel de la PCM y eran comunicados al comando para su ejecución eso también pude percibir entonces como usted comprenderá ya ni siquiera dependíamos de que en el ministerio hubiera gente que pudiera tener alcance técnico a ese nivel, sino que además a nivel de PCM había también ese hándicap,

Ahora de manera personal la gente que tomaba decisiones se podía reducir en el MINSA probablemente a 5 nombres y todos ellos están en la parte del comando y puedo decir que en ese comando no lo conformaba ni un representante del centro nacional de Epidemiología, ni un representante de la Dirección General de Gestión de Riesgos, ni un representante de la Dirección del personal de salud; 3 direcciones claves que siempre nos movemos juntas en lo que es emergencia y cuando lo propusimos para que lo colocara ya era muy tarde, ya se había difundido las normas del comando ya se habían hecho todo lo que

se tenía que hacer y, desde mi punto de vista, tampoco era necesario, porque lo único que queríamos era ser escuchados, y la comunicación se rompió como le comenté, ¿no? De hecho, el equipo de comando se fue al MINDIS es decir, ya ni siquiera estaba en el MINSA para conversar con ellos teníamos que ir. Hay una barrera física había una barrera de no escucha activa, y yo diría que estos puntos serán claves para poder tomar las decisiones más asertivas, entonces falta confianza, falta comunicación, escucha no activa, personal que quizás tomaban decisiones que no tenía el perfil de gestión de riego ni manejo de emergencias y desastres y obviamente, la intervención de la PCM que confían en alguno de ellos y no este y el otro es no dentro de un Ministerio falta comunicación interna entre los propios directores y sus equipos técnicos, si fue un caos de organización, un caos de toma de decisiones por no verticalidad y esto, pues se da en muchas emergencias. No es algo sólo exclusivo del Covid no hubo esa oportunidad, tampoco de congeniar es decir, el mejor equipo en respuesta de emergencia, tiene que conocerse cada uno tiene, sabe su función y lamentablemente con tantos cambios que hubo en el MINSA esa alta rotación, impedía que seamos un equipo, no éramos ni siquiera grupo, entonces y mucho menos equipo, de alto rendimiento o sea para manejar una emergencia se necesita un equipo, de alto rendimiento que trabajen en sincronía y eso ni siquiera grupo llevábamos porque ni nos juntábamos por último, entonces eso medio mucho en lo que es Staff y los recursos humanos encima de que no teníamos los suficientes a nivel operativo a nivel estratégico pues no había las conversaciones, como deberían hacerlo.

**I:** Y esto **¿podría verse debido a las condiciones propias que la pandemia del Covid que generó el tema?** por ejemplo, de empezar el distanciamiento social yo sé que tal vez no tenga relación, pero muchos de los vínculos que se habían establecido se lograron establecer cambiaron muchísimo por la pandemia.

**E:** Es cierto, quizás en un primer momento, pero no más de 2 semanas, porque le soy sincera a las 2 semanas después de aquel 16 fatídico de marzo que dijeron que ya no era nada presencial todas las actividades pasaron a ser virtuales y uno inclusive tenía reuniones en simultáneo y al mes nuestra conclusión era, somos más efectivos en virtual, porque antes no podemos ni alcanzamos una reunión y resulta que ahora estoy con 3, 4 reuniones, no había forma y todas las lleva a cabo claro hasta las 7 de la noche, porque ya después todo el mundo cerraba su Zoom. Entonces, pero eso yo no diría que haya sido la principal barrera.

Quizás las primeras 2 semanas porque obviamente, todos tuvieron que acostumbrarse al zoom y todos tuvieron que acostumbrarse a la virtualidad, pero después de 2 semanas eso echó a andar y todos nos reunimos por zoom eso era la normalidad. Entonces, por un zoom no era porque podían poner. Ok, no estaba el titular, pero podía ir un segundo, un tercero entonces, no creo que eso haya sido, de hecho, nosotros continuamos haciendo las reuniones que hacíamos de manera virtual y convocamos al comando, comando que solo fue en 2 de las 7 creo que llegamos a tener este y mi función era, por lo menos comunicar a todos los equipos técnicos lo que yo me enteraba, que se estaba haciendo y lo que yo hacía y escucharlos a ellos para transmitir esa información al comando de las inquietudes y tratar de juntar a todos para tratar siquiera de enrumbar al mismo MINSA.

**I:** Tener una comunicación y tener un mismo mensaje y comunicación para todos.

**E:** Así es. No porque estábamos en plena elaboración del plan de preparación para la segunda ola, porque ya la primera hora había sido un desastre entonces teníamos que construir el plan de la segunda ola desempeñando la segunda ola, tuvieron algunas dificultades en el tema de la decante de presupuesto y ya se estaba hablando de las vacunas y ya se estaba hablando, en ese plan estaba estipulado toda la parte compleja del oxígeno, nuevamente entramos a tallar con el plancito que teníamos, pero también estamos a tallar con el tema de las vacunas, tema que en ese momento no estaba todavía 100% dilucidado y por lo tanto, demoramos en la ejecución de ese plan pero los lineamientos estaban las direcciones participaron y un tanto que tratamos de a ver a ver mejorar ese primer plan que se lanzó el 31 que era plan de preparación para el no ingreso del virus así se llamaba no así se llamaba no, en este plan tenemos que lucirnos en el sentido de todo lo que nos faltó. Por eso comenzó a tomar mucho más tiempo y queríamos poner todo, que ya no puede concluir ese plan, ya le tocó a la siguiente directora

que asumió ese plan, pero si se tomaban las reuniones para que por lo menos el equipo técnico aportara las mejoras correspondientes que se informará su Dirección General, algunos directores participaban y ahí en estas reuniones, curiosamente, se enteraban todo lo que hacía DIGEMID lo que estaba haciendo persona de salud, lo que estaba haciendo "SI hemos hecho el vídeo hoy, mira, no sabía, pues pasarme la información" ósea sirvió de una ventana alivio para poder compartir información, de lo que estaban haciendo otras direcciones que no se sabían no entonces esto ya después ya no se siguió efectuando se logró terminar el plan pero ya no se concretaron al final esas reuniones con los equipos técnicos, entonces eso es lo que digamos esos fueron los que afectaron yo no diría que por no tener presencial. Eso es un factor, pero quizás los últimos puestos.

**I:** Doctora en cuanto a la parte normativa, usted diría que en este momento **¿son suficientes las políticas que tenemos para el tema del oxígeno médico? ¿Sabe usted si se han revisado las directrices, las políticas? ¿Cómo es que si se dieron?**

**E:** Tenemos vigente a través de DIGEMID aspectos importantes, SI se tuvo una directiva fuerte del manejo del oxígeno, pero debemos actuar de cara a la nueva reorganización de los servicios de salud post covid es decir, la directiva que tenemos están relacionadas a Covid y ya estamos post covid entonces deberíamos tener una reorganización porque en el periodo pico hemos alcanzado hasta más de 5000 camas UCI, pero camas UCI que no cumplen todas las condiciones que han tenido recursos malos entre Covid y no Covid hemos llegado a 5000 de lo que se ha reportado en el sistema de SUSALUD.

La gran pregunta es 5000 con oxígeno, Ok, ¿qué tipo de oxígeno no tenemos mapeado? Eso por digamos área crítica, por establecimiento, y por región, tenemos de manera importante otro avance entonces en ese en ese contexto de las normas hay que actualizar la directiva es muy buena, es bastante sólida la que sacó la DIGEMID en cuanto a las indicaciones el mantenimiento de las plantas, los tipos de plantas eso sí, pero tiene que actualizarse de cara a la nueva realidad.

Lo segundo, por si acaso venga otra emergencia. Lo segundo es el tema de la información que hemos crecido, porque hay un sistema de DIGEMID que permite conocer el estado semaforizado de las plantas de oxígeno y la cantidad de oxígeno para los últimos semanas o 15 días eso también hemos crecido porque ese es un reporte obligado lo que tiene que mejorarse al respecto de ese sistema es el control de la calidad de la información, porque ¿qué pasa? yo podía haber llenado como hospital y lo lleno hoy que estamos este primero en julio, pero de pronto pasan 3 meses y no lo lleno más, entonces el último día aquí no primero julio viene la emergencia de septiembre, y en MINSA tiende la información de julio puede decir,

no, pero el Hospital X está muy bien porque aquí está reportado. ¿Oye, pero esa información de cuándo es eso? Eso no sale en el sistema entonces el sistema tiene que tener la capacidad de poder tener control calidad. Por eso digo que tenemos que tener también recursos humanos informáticos que pueda haber, y eso mejorar los sistemas de control de calidad este segundo punto de gestión de información muy importante para tomar decisiones, se tiene, pero hay que hacerle control de calidad. Ahí está, ¿Cuántas plantas están en funcionamiento? ¿Qué regiones están en rojo, amarillo, en verde? ósea eso es muy bueno que lo tiene DIGEMID a través de su página.

Y lo tercero es muchos de los equipamientos adquiridos han sido adquiridos por la parte privada y ahí el ministerio de salud y las entidades, digamos públicas no se han hecho cargo porque no las adquirieron ellos, entonces cuando tuvimos el plan de preparación de tercera ola particularmente insistí de manera enérgica de que el Ministerio de salud tiene que tener una política para recoger ese mantenimiento de todo lo que es privado porque no podemos decir simplemente a porque privado ya no me encargo de eso tengo que hacer un nuevo inventario y hacer mío esas plantas de oxígeno, definir con las empresas que lo donaron este sí que van a colaborar con el mantenimiento porque no es cuestión de comprarnos más la planta sino tengo que hacerme cargo o hacer convenios o lo que fuera. Ese mapa no lo tenemos y lo exigimos para el plan de tercera ola la respuesta fue “No es competencia en la parte pública, porque eso fue comprado por privado, y yo no puedo invertir en algo privado porque si no es malversación de fondos” entonces aquí vamos al cuarto y punto, importantísimo estamos amarrados, de manos con las mismas normativas que existen en el país por el miedo a la corrupción, por el miedo a la corrupción lamentablemente no puedo desertificar presupuesto si no he cumplido un objetivo determinado, y así me está muriendo en otra necesidad, no puedo, porque si no me denuncian como malversación de fondos y ese miedo hasta lo ha manifestado actualmente para el fenómeno para lo que fue este esta tormenta que tuvimos Yaku con la misma Ministra cuando asumió, dijo, “no tenemos ahorita, porque eso es malversación de función” como un representante del estado va a decir eso ya el más alto nivel tendrá que ver si está ahí el presupuesto no tiene que certificar sacará una resolución presidencial si es necesario para hacerlo, para eso está el consejo del Ministro, entonces que un ministro diga eso públicamente refleja la mediocridad en la capacidad de gestión y cómo nos hemos amarrado tanto en el miedo de no firmar documentos y ahí a la prueba me remito el ejemplo que le di del oxígeno, ¿Cómo es posible que en 3 meses lo hayan sacado de planificación del 2018?, yo tenía 2 caminos o jalaba flecha y seguía mi vida tranquila y feliz o me hacía miles de problemas y lo sacaba.

Decidí la segunda y por decir, la segunda recibí una denuncia justamente por negociación incompatible por comprar algo que no estaba en el plan del 2018 por más que estaba en el plan del 2017 y que lo regularicé en el 2019 y sigo en el proceso entonces obviamente son pocas las personas que se van a cargar procesos de esa naturaleza penales y administrativos por culpa bendita de que no acá hay corrupción inclusive han habido intentos fallidos de ministros que ante este miedo han decidido hacerse de buenas colaboraciones con las de contraloría y decirles hasta que participen en los procesos realmente de compra lo cual no puede ser, porque no puede ser juez y parte y posibilita también las tomas de decisiones adecuadas, tras eso, las adquisiciones del Estado son obsoletas, porque pretenden hacer una compra al más bajo costo y lo que piden es competencia con especificaciones técnicas que tengan algo semejante y cuando uno pide algo diferente, dice que está direccionado y eso es incorrecto, es una apreciación inexacta porque actualmente las ofertas que hay de equipos tienen diferente tecnología entonces yo no puedo pretender si yo tengo en el mercado 5 tecnologías no puedo pretender que el Estado tenga 5 fichas técnicas semejantes y que opte por la más bajo costo, porque las 5 tecnologías van a ser diferentes, entonces una va a criticar a la otra y eso es lo que pasa en la realidad que no estoy direccionando porque quiero esto quiero la otra.

Entonces hacer un sustento técnico se vuelve un Frankenstein ¿no? Y en el MINSA los ingenieros que ven esta parte logística lo cambian, lo acomodan para que pueda calzar en estos reglamentos que son obsoletos reitero cuando deberíamos comprar de acuerdo a lo que un equipo técnico que no se ha nombrado en el MINSA , y esto lo reitero, lo queríamos hacer en 2017, pero no hubo tiempo; un equipo técnico conformado por lo menos 5 direcciones, como lo tiene EsSalud y que ese equipo sea responsable de sacar digamos las mejores especificaciones técnicas al margen del más bajo costo, entonces en este corto punto de problemas legales, por tener miedo a la corrupción y la forma en cómo adquieren las cosas del Estado, es también una gran limitante que impide que las operativos, los estratégicos a nivel de MINSA y CENARES compren lo mejor eso es una gran debilidad y si compran, para no meterse en problemas, no necesariamente están comprando lo mejor para el país, porque se tiene la absurda idea que, si es un presupuesto mayor de la habitual, no acá hay algo no acá hay algo que no es adecuado, y si seguimos en esa roca, el Perú va a seguir como ahora tristemente puedo definir en un ejemplo tan simple en mi UCI los tubos endotraquiales de los pacientes que hemos hecho prácticas y evaluaciones, revisa nomás para revisarlo a ver este tubo ni siquiera describe ahí en su empaque de qué material está hecho cuando sabemos que lo materiales son importantes para reducir las neumonías asociadas al ventilador no lo dicen, entonces eso es una clara muestra del nivel en el que estamos. Y si así estamos en un aspecto crítico de pacientes en el último escalón de la Salud Publica, pues vayamos a ver cómo están todos los demás.

**I:** Doctora en relación a estos temas de compras en general, la parte de los financiamientos **¿De donde estamos actualmente o como estamos actualmente financiando los servicios de oxígeno, médico tiene usted información al respecto?**

**E:** Bueno, podría dar cierta información ahí si tengo ciertas limitaciones porque no es la dirección que lo que sí puedo decir es a nivel de hospitales, en los hospitales efectúan convenios con empresas grandes como PRAXAIR y PRAXAIR es la que da el servicio, por eso es que hay cierta dependencia a estas entidades privadas dado a que ellos son los que nos surten del oxígeno, de hecho en pandemia en la segunda ola ya fuera del ministerio particularmente tuve que tener una conversación con ellos porque ni el director ni la directora del momento ya habían dado por sentado que hasta ahí nos podían dar oxígeno. Entonces tuve que llamarnos tener una conversación para poder de alguna manera, flexibilizar algunos aspectos técnicos de la dotación de oxígeno, pero entonces cuando uno adquiere estos servicios por convenio la parte técnica de cómo lo distribuyó y los horarios depende mucho ya de negociaciones internas, entonces no significa que cuando compre ya este compra con todas las indicaciones puede cambiar eso no está estipulado en el momento que uno compra como lo que estoy comentando y menos en condiciones de emergencia y desastre, ahora por otro lado los insumos que son los balones ya las los materiales que se utilizan para la conexión directa al pacientes, los medidores de presión y todo eso también se tienen limitaciones en aumento de las compras, por alguna razón, compran, o sea que pueden comprar de pronto 100 balones, pero no compran 100 medidores no sé por qué, o sea eso es una limitación. No podía entrar a tallar no es porque no se pida, es porque se hace priorización en las adquisiciones.

Entonces en el concepto de logística lamentablemente esas priorizaciones muchas veces no decantan en la jefatura para la toma de decisiones, ¿no? Entonces, y eso es todo un problema doble a veces los jefes no ven la importancia de ir a la logística para definir lo que se está comprando y, por otro lado, logística tampoco a veces lo llama porque ya es un caso perdido y ellos toman la decisión entonces el tema de adquisiciones.

**I: ¿Se refiere a que los equipos de logística son los que priorizan las compras?**

**E:** A veces porque uno lamentablemente pues tienen que ya tomar la decisión por presiones de las Direcciones y en otros, porque, a pesar de que llaman a los jefes los mismos jefes no tienen la conciencia de que esa participación de ellos, es vital para lo que va a venir para sus servicios, eso no lo he vivido lo he visto, he tenido que presionar al director por favor vea, revise, entonces considero que ahí el problema es doble. El presupuesto, por supuesto, puede venir de diferentes partidas, porque el presupuesto lo tienen en un hospital el departamento de emergencias a través de sus programas presupuestarios. El programa Presupuestal, 104 que es para emergencias y para todos los servicios ahí, tiene también una partida, pero no es la única, porque, lamentablemente ese presupuesto que yo debería poderlo incrementar con requerimiento de oxígeno porque para eso fue creado el 104 lamentablemente se encarga sólo de pagar al personal, es exactamente el 90% es para pagar recurso humano, entonces las emergencias del país no tienen techo para pedir ellas como decir de manera muy operativa, cambios que pudieran tener o mejoras en su emergencia, que están limitados a lo que pueda pedir la otra oficina que la oficina de servicios generales y las oficinas de servicios generales tienen que tener una visión amplia de la situación en condiciones regulares y en condición de emergencia para las adquisición ellos tienen también su presupuesto, entonces ahí es otra partida que tienen para la adquisición y finalmente, la tercera partida es el 068, pero a nosotros nos niega mucho, porque dicen, no para eso está la 104 y para eso están los hospitales, tú solamente pide para hospital de campaña.

Entonces, evidentemente, da pena decirlo, pero estamos entrampados de brazos cuando se trata de presupuesto y finalmente decanta el pedido en la Dirección General y la Dirección General lo mandan MINSA a la Dirección de operaciones y la Dirección de operaciones para aprobar el presupuesto de lo que viene ahí en lo que es: balones oxígeno, plantas Hace la última priorización entonces todo el trabajo que uno hace en hospital al final cantan porque dice no a este hospital no al otro hospital.

Entonces es muy exigente la manera en cómo a veces este parece Esto cuento ¿no? pero es así, el día que tengamos una política clara de prevención, de preparación, de hacer caso a esos índices de seguridad hospitalaria que me dicen estos hospitales están en rojo, por caer si necesitan eso el día que se haya un planeamiento de acá 20 años de cambiar vamos a parar pero ha tenido que venir el Covid para que se den cuenta que realmente estamos remal y la pregunta que me hago es, y eso que no ha venido el Sismo, viene el Sismo en lima, ahí sí no tenemos ya ni siquiera espacios ni hospitales porque los hospitales con estos modulares que se han refrescado pero no es la solución y la gran mayoría está tranquilo porque tienen los modulares todavía 3 años del Covid que se están destartalando pero esa no es la solución la pregunta es eso también lo plantee en el 2021 ¿ cuál es el plan de retomar los hospitales los destartalados que se están cayendo a pedazos? y que ahora tenemos un tercer problema, porque no solamente la estructura fallida de ese hospital, el segundo problema que tenemos son los modulares en el espacio donde iban a estar los nuevos hospitales el tercer problema es ahorita tengo toda la avalancha de gente que se ha descompensado post Covid sin Plan post Covid, entonces, ¿cómo vamos abordar esto? Esto es una política al más alto nivel que tiene que salir con ley a 20 años de priorización.

**I:** Doctora y la ley, **¿y la Ley para la Atención de Situaciones de Emergencia y desastres naturales no ayuda?**, sé que es bastante genérica, es como no sé si se aplica, esta desfasada, habría que modificar.

**E:** Lo que pasa es que no solamente las leyes decantan o tienen que estar en planes estratégicos, tuvimos la oportunidad de participar en plan estratégico del MINSA, pero no es el plan estratégico del MINSA, o sea, la que hemos pasado amerita una intervención de la Presidencia del Consejo de ministros a nivel del país y lamentablemente el CEPLAN no cree que esto sea una política, esto cree que está suscrito a hospital allá. O sea, no ha aprendido el CEPLAN que el problema de los hospitales no es MINSA, el CEPLAN no ha aprendido que el problema de los hospitales es todos los ministerios, porque para el hospital tengo agua, tengo que tener luz, tener el terreno, tengo que tener saneamiento, tener a Gobiernos regionales abiertos que hagan hospitales de verdad que los verifiquen no ha aprendido es como tampoco ha revisado el sustento que hemos remitido de las lecciones aprendidas de Japón, que ya van por la segunda del marco del 2015 de Yogo, del marco del 2015 Sendai, de cómo tenemos que tener hospitales seguros frente al desastre lo que implica.

Entonces tenemos actualmente una política del 2017 no reconocida por CEPLAN, porque dice que esa política es muy de servicio y política tiene que ser aquello que toque directamente al paciente, entonces tenemos una conceptualización pésima, porque si voy a esperar que la política es todo que toque salud pública al paciente estoy mal, o sea, porque la infraestructura no lo toca o que el oxígeno, digamos, no es quien lo va a seguir a su casa no puede ser política. Estamos mal, entonces esa política tenemos que mejorarla, resucitarla, involucrar, cómo se involucran los demás ministerios: energía, vivienda, porque se había involucrado y trazar un plan con esa política dentro de los planes a mira esa política en los planes estratégicos de cada ministerio que cada ministerio se comprometa. Ok, si estamos en rojo en Cajamarca, en Amazonas, en Loreto, Madre de Dios yo me comprometo a priorizar ahí la luz, el agua, que sé yo y específicamente para estos hospitales eso es lo que falta, entonces, no hay un esto lo miro una sincronización de capas de lo que hacen energía, energía, lo que hace vivienda, lo que hace salud, lo que hace educación, para desarrollar lo que se llama esto lo conocemos bien los gestores de riesgo, la reducción de riesgo por población y por comunidad, no de manera asincrónica, por pedacitos.

Entonces no es competencia del MIONSA a los hospitales de salud, es competencia del país de la PCM de alguien que orqueste y que ahorita se ponga a trabajar a 20 años qué vamos a hacer si los espacios quedan para los hospitales nuevos están ocupados por esos modulares que se están destartalando en el momento y que son usados por todas las contingencias porque valgan verdades antes del Covid teníamos necesidad por cualquier mercado ahora lo están usando, la pregunta es: ¿hasta cuándo vamos a seguir jalando flecha?

**I:** Bueno, si pensando en eso, precisamente **¿hay algún mecanismo de supervisión de los servicios de oxígeno medicinal?** Usted me habla de estos hospitales se implementaron, pero de alguna en aquellos establecimientos de salud en los que se implementaron las plantas, los concentradores sabe usted si se está haciendo no sé un seguimiento una supervisión.

**E:** Sí. Los equipos del MINSA, a través de la dirección de operaciones y justamente parte de la preparación del plan de la tercera ola, incluye equipos que pudieran acudir a esos establecimientos y hacer un mapeo, no la dirección de operaciones lo hizo con su equipo, pero no fue un mapeo alturado con ingenieros, con biomédicos, fue un mapeo de personas que delegaban para ir y hacer un Excel “ah cómo está bien o mal, número, cantidad” no de manera técnica, como debe ser, primero, porque no teníamos recursos humanos para ir por todas las regiones buenamente se hizo un mapeo y lo segundo como les digo es esa hoja que llena ese sistema que llena de 2 fuentes, la DIGEMID lo mapeo, pero ese mapeo no es alturado, no es frecuente; se hizo por la tercera ola desconozco si lo han vuelto a hacer, siempre recomendamos eso, y el

tercer punto teníamos un aliado que era SUSALUD, iba y supervisaba pero ese aliado le quitaron el presupuesto justo para la tercera ola y cuando yo pedí que fuera por cómo me apoyaban y todas las emergencias la respuesta del en ese momento funcionario fue: no puedo Mónica porque lamentablemente me han quitado mi presupuesto y no tengo, lamentablemente, gente que te puede ayudar en esta oportunidad.

Entonces estos 3, la respuesta es si hay un seguimiento, pero no es eficiente porque va personal que no necesariamente tiene la expertis técnica para definir en qué grado esté afectado porque no hay suficientes ingenieros, no hay una dirección general, no hay una dirección ejecutiva que es la DIEM que la que se encarga de eso insuficiente para todo el aparataje a nivel nacional y delegan en los gobiernos regionales, que tampoco tienen la competencia en hacer esa tarea. El segundo, el sistema de DIGEMID, que si es que es muy bueno lamentablemente no tiene control, ósea no sabemos a ciencia cierta que porcentaje de lo que está escrito ahí realmente es. Y lo tercero que SUSALUD, que debería fiscalizar hasta donde sabía, no sé si ahora ya lo tiene, pero no tenía suficiente recurso para poder enviar y contratar gente a todas las regiones y hacer un levantamiento de calidad de la data.

**I: ¿Hay algún aspecto que ya está, digamos, normado al respecto de la supervisión del seguimiento que se deben hacer?** Usted me hablaba de que había como una normativa que sí había establecido, pero que no tenía como el presupuesto o que no se venía implementando.

**E:** Las directivas que le digo de oxígeno que datan del 2020 y 2021 determinaban lo que el check list de cómo debe estar la planta y todo eso pero quienes hacían la supervisión siempre han sido de manera general DIRIS, DIRESA, GERESA no decantaba en aspectos específicos eso obviamente cada DIRIS, DIRESA, GERESA tiene que hacer su propio plan paralelo para ello, pero ¿qué va a hacer si no tiene recursos para ello? No tiene recursos ni humanos, ni logístico, ni presupuestal para eso es el gran problema, se crean normas, pero el presupuesto específico para ello no hay. Puede ser que la DIRESA tenga presupuesto, pero como no está estipulado, no puede utilizar el presupuesto entonces ese es otro problema que tenemos.

**I:** Ya, usted **¿podría asegurar que el oxígeno medicinal, que en este momento se está que produciendo las plantas que se han instalado tienen el requerimiento, el porcentaje, la calidad necesarios?** porque ya ha pasado 2 a 3 años probablemente de implementación de las algunas plantas si no se hace el mantenimiento o se hizo eventualmente se podría estar garantizando en este momento que se tenga oxígeno o que responda en la cantidad que se requiere.

**E:** Pues ahí tenemos un problema porque todo equipo que no se utiliza lamentablemente se valora y existe también otro concepto errado en el MINSA en donde lamentablemente difundieron me consta porque yo estuve en esas reuniones insistí en que eso no es así. Planta que no se utiliza porque no hay este, pero no hay este, digamos, pacientes, planta que ya no se ya hay nomás, o sea, prácticamente no se le hace la prueba no se le da luz, ¿no? Entonces ese concepto equivocado que nace desde hace mucho tiempo atrás en donde se decía los hospitales de campaña modulares, si no hay emergencia, no se mueven graso error porque si yo no lo muevo va a pasar lo que yo encontré en el 2017 equipos con las baterías totalmente digamos, malogradas equipo, sucio, equipos sin funcionar, tuve que contratar ingenieros, arquitectos para levantar todo lo que tenía por el craso error de decir no, esto no utiliza cuando

actualmente la este ya a nivel OMS, OPS por manejo de desastres ya se sabe que todo el equipamiento lo tengo que tener en funcionamiento ya este la necesidad del país es tan grande que necesita cerrar brechas en campañas de salud atención en todas las emergencias que hay yo puedo movilizar los equipos de la misma manera se debe hacer con las plantas me daría madera, pero esto ha decantado en que las plantas en donde no hay este paciente simplemente se cierre. Como les dije no hay calidad porque simplemente todas las plantas de a nivel privado no han sido asumidas por el Estado ni por los gobiernos porque los gobiernos regionales también debieron asumir esas plantas con su presupuesto esta realidad que le digo es punto de corte al 2022 noviembre ¿Por qué? Porque fue la fecha en donde ya dejé de participar en las reuniones con los viceministros y con los directores generales hasta donde yo tuve la oportunidad de exigir la política debería ser que el MINSA y los gobiernos regionales a través de una normativa vertical asumiera y en sus planes figuraran que adopten esas plantas para darle mantenimiento hasta esa fecha noviembre 2022 no se había tomado acción de ello, entonces, de pronto, ahora han cambiado las cosas no podía aseverar eso pero mi punto de corte estas últimas reuniones que estuve en noviembre del 2022.

**I:** De su perspectiva. **¿Cuáles deberían ser las áreas claves en las que, desde hace cualquier nueva política sobre el oxígeno?** En qué debería estar, como se centradas o enfocadas.

**E:** Bueno. Ahí tenemos 2 aristas; la primera arista es primer nivel de atención y atención itinerante o extrahospitalaria y en cuanto a los servicios que voy a brindar, y la segunda es la hospitalaria, pero todo esto se en una gestión de ¿Cuál es mi demanda actual? y yo creo que esto se tiene que basar en una gestión de información fuerte en una inteligencia de la data que me permita saber mi demanda en 3 dimensiones; nivel convencional, a nivel contingencial y a nivel de emergencias y desastres. Si yo no tengo mapeado estos 3 escenarios de cuál es mi demanda actual real, mi demanda contingencial, que es la que vivimos en todas las ciudades del Perú, no hablamos de Lima, todas las grandes ciudades, vivimos en sobredemanda, no podemos dar atención, a todos que hiciéramos no podemos esa es nuestra realidad y la situación tercera, que es la de desastres, entonces con toda la información de Covid ya se debe tener alguna información de cuál fue mi necesidad real de oxígeno máximo, la pregunta es: ¿eso esta Censado en algún análisis? la información la tiene DIGEMID esta parte se sustentarme en una inteligencia de la data que ya existe, que la tiene DIGEMIN y evaluarla con un Censo de pronto nacional de campo para verificar que lo que está escrito tenga calidad la información; una vez tenida esta información de lo que estoy gastando lo que gasté en el peor momento yo puedo construir estos 3 sobre estos vienen los servicios y los servicios es a dónde voy a ofertar, de dónde voy a ofertar, de pronto la información de DIGEMID es incompleta y no alcanza la parte pre hospitalaria o fuera del hospital que son todos los pacientes que normalmente requieren oxígeno, todos los pacientes que no llegan a un primer nivel siquiera esa data tampoco se levanta que es cuestión de verificar la data del CDC del Perú respecto a los pacientes que murieron por neumonía antes de llegar a un establecimiento de salud que en algún momento se tuvo como data también para la gestión de los generadores de oxígeno y una vez que tenemos todo eso viene ya esté en la inteligencia de data, qué servicio voy a dar, viene cómo voy a dar el servicio lo voy a dar con plantas, lo voy a dar con concentradores, lo voy a dar con balones o con qué lo voy a dar, entonces yo creo que esta visión que le estoy comentado está la segunda y la tercera en el plan pero la primera parte que es la eso que además, eso lo sabemos los gestores de riesgo no la he visto la propusimos durante todos los lunes que teníamos reunión en general, todo tenía que ser planificado de esa manera bajo ese esquema de 3 escenarios para no engañarnos y no tener cifras anecdóticas sino tener cifras reales y apostar a arreglar nuestro sistema informático a la brevedad posible, no la hay; yo pienso que eso es un gran error porque se trazan siempre y sobre todo en emergencias cifras de acuerdo

al equipo que esta de turno y no en una planificación alturada que permita ver lo que va a pasar después de la emergencia, entonces vivimos en una contingencia el día a día, contingencia que no está mapeada y este porque hay data oculta en todo nivel, Pre hospitalario, primer nivel de atención hospitalario y eso pues tiene que corregirse.

**I:** Esta visión que usted me comparte ahora **¿es compartida por los funcionarios del MINSA, los directivos de este momento o no?**, porque yo, después de conversar con ustedes, realmente me estoy quedando, como preocupada porque digo si es obvio para alguien que está involucrado en la gestión porque no lo es para las actuales autoridades o que se está haciendo al respecto.

**E:** Bueno, esta visión que yo tengo de la situación como abordo los problemas enfocada en el manejo de grandes emergencias y desastres, esto está publicado, esto es algo que lo he hecho público, en las reuniones y no lo veo en los planes, hay una visión general en qué vamos a pedir cuando hay un desastre decante en una emergencia sanitaria y ahí se pone o de alguna manera debería ir, pero no todas las direcciones tienen esa capacitación necesaria y esto es también un punto importante; se tiene que creer en esta visión y qué es lo que pasa realmente siempre la emergencia nos gana y hasta que las autoridades y que de pronto no tienen esta competencia clara se empapen de esto ya tienen que dar una respuesta. Entonces exige a sus directivos ¿Cuánto necesito dime cuánto compro dime qué hago, dame el plan? entonces se pierde esa oportunidad de tener un análisis este es el momento, estamos en un momento clave, porque estamos a la espera de otra gran emergencia sanitaria de hecho, ya hemos vivido una la del Dengue eso podría comentarla o el tendido de todo lo que se previno y lo que no se hizo, pero por cuestiones éticas no lo hare y lamentablemente podría decir que no todos tienen esta visión, ahora este forma que le digo yo lo he sistematizado así en función a lo que he revisado y como especialista en gestión de riesgo abocada a la parte de salud siempre ha sido mi preocupación manifestarlo a las autoridades correspondientes y brindar ese asesoramiento a la dirección que corresponde que es la dirección de operaciones. Si la dirección de operaciones no tiene este marco que se lo hemos dado en zoom, en presencial y con documentos siendo asesor al despacho de salud pública, se lo di con documento esta visión y tuvo una reunión con SUSALUD, con representantes desde GOZ, con representantes los Viceministerios para que lo tengan claro bueno yo cumplí con mi misión de informar, pero son competencias que considero al más alto nivel, tendrían que tener los funcionarios que asumen los puestos.

**I:** Doctora un entrevistado, me comentaba que, bueno, la percepción que tenía era de que en la ley sobre todo sobre desastres, emergencias y desastres naturales no se había considerado el tema de las emergencias sanitarias y por eso es que como que había algunas dificultades por ejemplo, por el tema de las donaciones y esto que usted ya me decía de que los establecimientos de salud no le dan mantenimiento porque asumen de que eso no es del Estado, es de un privado, porque ha sido donado, fue comprado por instituciones de fuera pero bueno, no es no es del Estado entonces había como toda una dificultad; esta persona me decía que era porque no se tenía la percepción de que, por ejemplo, el Covid o el Dengue o alguna emergencia sanitaria no estaba contemplada como parte de una emergencia o un desastre. Entonces me llamaba la atención por eso, por la visión, la percepción que esta persona tenía al respecto.

**E:** Ahí tenemos que aclarar 2 puntos. En primer punto: efectivamente la ley de SINAGERD actual está muy, muy abocada al tema relacionado a desastres de origen natural, pero eso no quita el concepto de riesgo y el concepto de peligro y el tema de la pandemia en sí ha sido tocado en la política es decir es una ley, pero la política de gestión de riesgo nacional si habla de pandemia quizás no le da el impulse necesario, la fuerza, los gráficos que se ha perdido la oportunidad porque lamentablemente la política que salió en el 2021 es una lástima yo casi lloro cuando veo la política, porque habiendo tenido en 2020 la oportunidad de oro, el Ministerio de Salud de intervenir ahí fuertemente en esa política no sé si los convocaron o no, no han hecho pues un despliegue fuerte de conceptos en la gran política de gestión de riesgo, pero si aparece pandemias sí aparece pandemias, entonces si me dice en aquella época teníamos el marco, tenemos el marco la ley de SINAGERD que teníamos el marco del estado de emergencia, el estado de emergencia se sustenta en la emergencia sanitaria que es otro contexto, entonces ¿por qué no se trató como tal? Eso ya son decisiones de los equipos que comandaron que políticamente no querían llamarle desastre a lo que hace rato era desastre pero las normas eran claras a tal punto a tal punto que la gente se olvida de una grabación del Presidente de aquel entonces, que era el doctor era el este bueno, Vizcarra, el Presidente en 1 de los primeros discursos de lo que comienza de manera rutinaria él activa y bueno, está mal decir porque todos los COES siempre están activados, pero de manera figurativa activa todos los COES lo dice públicamente no lo dice explícitamente, estamos en un desastre y activemos los coches, pero dice enfáticamente en un mensaje, lo recuerdo vivamente porque dije por fin, ahora si entremos a tallar con el MINDE y dijo "a partir de este momento se activan todos los coches a nivel nacional provincial por esta emergencia sanitaria" no dijo desastre después de ese esa reunión que debe estar grabada alguien de tener grabada porque fue publicación nacional nunca más volvió mencionar lo es COES que supongo que sus asesores le habrán dicho, yo intuyo no hoy ya no y lo otro que le digo porque estoy segura de eso uno de los doctores que eran los y para mí, digamos, el encargado de manejar este asunto, porque la jefe de comando sabemos quién era so tenía un conocimiento, una doctora de prestigio, sí total reconocimiento su labor, pero en cuanto a gestión de riesgo tenía limitaciones y se basó en los equipos que tenían y el que le seguía en el mando que era quien reportaba al presidente de la República, a mí así Face to Face me dijo "Mónica no pretendas manejar esto como un desastre, porque no lo es y lo que tú quieres presentar de tus indicadores sobre saturación y sobre demanda no le interesa al Presidente de la República" esas fueron las palabras textuales y la última reunión que tuve con él, porque, por supuesto que dije aquí de qué estamos hablando después de ese episodio funesto hablé con el ministro yo a punto de renunciar y claro, recibí por ahí asesoría de otros colegas porque en ese momento se veía mal tu renuncia.

Porque encima querían quitar la alerta roja yo determiné desde el MINSA que se dictara en abril la alerta roja que no la querían sacar la tenía encarpetada un mes, todo marzo, morían de miedo de que yo pidiera la alerta roja. Fue con el doctor Zamora, que se sacó la alerta roja a las 2 semanas que saqué la alerta roja puedes creer Lizzete que la jefa comando pidió que la retiraran.

Ahí fue otro punto de quiebre que fui a hablar con el ministro y dentro de mí dije si realmente con lo que le dije quiere retirar esta alerta roja, que es lo que ha permitido reabrir los establecimientos primer nivel que lo que me permite hacer triaje terceros y un montón de cosas más y si yo renuncio porque ya esto es demasiado y lo hago público además, una de las cosas buenas que tuvo Zamora fue ponernos Face to Face con el comando y ante el sustento técnico que le dimos Zamora dijo no, va la alerta roja y se queda la alerta roja entonces eso es algunas muestras de la situación y no es tan cierto de que el que no esté de manera explícita haya impedido, porque conocen la ley quien conoce los términos de emergencia en Italia y el Estado de emergencia por Dios, fue dictado tal cual se dictó en el 2017 y en otros eventos entonces tan solo con ese Estado emergencia era para activar todo, como bien lo hizo Vizcarra, pero que parece

que ahí pararon el discurso, se notó claramente porque en los siguientes que yo seguí de cerca ya no hablaba nada de COES nada.

Entonces yo que tenía todos mis amigos militares, entre comillas, amigos, les digo porque he trabajado tantos en emergencias decía, pero ya actuamos, ¿Qué pasa? me decía, pero Mónica. ¿Qué vamos a hacer si no si no nos dan explícitamente órdenes de eso? Entonces quien tenía que tomar la batuta era la PCM empoderando al Ministerio para la toma de decisiones, se empoderó al comendo, pero el comando no tuvo la rigidez de manejar al COES y yo conversé con ellos cuando retorné al MINSA y les dije por supuesto, sus verdades de que por qué no habían comandado y la respuesta fue: no podíamos conversar con efectivos militares que no tienen las cosas claras en salud, craso error, porque ellos nos podían haber ayudado en miles de cosas y subestimaron el nivel que tienen muchos militares a nivel de planeamiento estratégico que de hecho hay cursos que se dictan de planeamiento estratégicos por militares y la estrategia comienza por un nivel militar, entonces, otro error del MINSA fue subestimar a la gente que está en el MINTER.

**I:** Doctora ya para ir cerrando. **¿Cuáles diría usted que fueron los retos mayores?** O principales. Respecto del oxígeno durante la pandemia.

**E:** Bueno, el principal reto fue de la gestión de información no teníamos una información clara de la demanda de oxígeno y la semaforización en la primera ola actualmente lo tenemos, pero falta control de calidad y eso es un reto importante. Tenemos que saber dónde se necesita, cuánto se necesita el nivel y tienen que tener todos acceso a una base con Internet para poder estar comunicados en tiempo real lo primero es sin información no sabemos nada; el segundo punto es necesitamos un fuerte contingente de recursos humanos de lo que no hemos contratado ingenieros, técnicos, capacitaciones a ese nivel, porque son ellos los que van a hacer el mantenimiento; y eso no lo tenemos en los gobiernos regionales, no lo tenemos ni en la misma Lima y eso genera un déficit importante y tenemos que darle una retribución económica acorde con la exposición que ellos están dando la retribución económica que reciben es por lo demás irrisoria y eso pues no motiva a los profesionales de ingeniería en trabajar en salud para el pago que le dan prefieren irse la empresa privada, entonces necesitamos en segundo punto un contingente ingenieros técnicos bien pagados remunerados que permitan levantar toda esa información que se primer paso y poder decir: este es mantenimiento preventivo esto es correctivo esto es lo que me falta. Eso necesitamos eso.

Y en tercer lugar, necesitamos jefes, directores, ministros, entidades de tomadores de decisiones que conozcan cuál es el perfil de escenarios que hemos comentado si no conocemos los escenarios contingenciales y de desastres que siempre vamos a vivir van a seguir siempre cometiendo el mismo error pidiendo a última hora lo que se puede priorizar lo que alcanza el presupuesto y no lo que deberíamos proyectarnos y trabajar digamos a largo plazo todos los requerimientos.

**I:** Desde los diversos sectores, se hizo a algún. **¿Se tomó alguna acción para abordar estos retos?** Usted noto si se llegó a superar o no.

**E:** Bueno, como le digo en el tiempo, ya para la tercera ola teníamos fíjese para la segunda y tercera ola ya teníamos sistema de información a través de la DIGEMID, el sistema es RENOXI muy bueno que además,

además permitió que la base de gestión de cama de SUSALUD pasara ese sistema y ese primer reto digamos que se ha cumplido pero falta ver la gestión de calidad ampliarlo, mejorar; el segundo reto relacionado a los digamos, ingenieros y todo el personal del Staff no, eso no se ha mejorado eso es algo que sigue en el vacío que nadie lo toma porque claro el Ministerio de Salud vela por los médicos, enfermeras, profesionales, salud, pero quien vela por los profesionales, de ingeniería no tenemos eso te necesitamos ahí el levante información ahí estamos cero cero.

Y en el tercer punto, ya que tiene que ver con el conocimiento y las capacitaciones, pues ahí también tenemos un base importante hay limitaciones, este que tienen que ver también con que tienen ciertas que tienen que tener las personas para poder enseñar a los demás, lo pongo de manera explícita los que toman decisiones generalmente apuestan de diplomados para arriba diplomado maestrías y lo demás, pero no todos estamos, y yo no digo que sea la única, pero estos conceptos que he vertido lo abierto en función a lo que me he nutrido a lo largo de estos años a la experiencia personal en campo estratégica y lo puedo dar en curso de hecho actualmente estoy dando un curso que es de gestión de riesgos en grandes críticas a través de la universidad, porque siento que esto lo debe conocer más gente, pero es limitado, porque yo no pongo por ejemplo el momento dictar un curso en una maestría o en una en un doctorado o lo que fuera porque lamentablemente yo solo tengo egresada de una maestría y aunque tengo un máster en gestión de riesgo de una universidad española no está reconocida por SUNEDU yo no sé cuántos más de los que tenemos este conocimiento operativo técnico vivaz para manejar emergencias están en competencia para poder dictar esto al más alto nivel y estoy segura también cuando revisó los programas académicos de otras universidades que quienes dictan estos temas tienen el concepto teórico, pero no el concepto práctico.

Entonces nos ponemos trabas nosotros mismos. ¿No? Si quieres enseñar, tienes que tener esto el otro, y no todos tenemos esas competencias. Entonces hay que aprender de todos ahí tendríamos que abrir campo, a las especialidades a las universidades para que nos permitan compartir aspectos prácticos como ex funcionario, ex directivos y también dar nuestra verdad sobre lo que pasa y probablemente así se despierte un poco el conocimiento de los que actualmente toman decisiones.

**I:** Yo sé que los directivos, los funcionarios públicos que están en los cargos más altos rotan de manera anotado de manera constante, **¿Y cuál diría usted que la situación hoy en este momento a nivel de instituciones se habrá aprendido algo, algo habrá cambiado a raíz de la pandemia?**

**E:** Bueno cuando yo retorné a MINSA en el 2021 después de la haber pasado la segunda ola en mi hospital, ¿Qué aspectos positivos noté? Había preocupación por la gestión de información, preocupación se formó una unidad de inteligencia estratégica pero lamentablemente no se pudo continuar yo forme parte inclusive de esa unidad tratamos de unificar la información de todas las direcciones otro salvaje de dominar, pero pese a todos los esfuerzos que se tuvieron en algunas, digamos, como algunos líderes, no se pudo mantener, no se puede mantener porque no todos tienen el concepto de la inteligencia y data como base para toma decisión, entonces, si hubo un antes y después hay mucha preocupación por analizar pero a la hora de concretar se abruman con el día a día las autoridades ¿Por qué se abruman? porque tienen una visión operativa y no estratégica, es decir, quiere solucionar lo que sale en la televisión y no se preocupan por los planes estratégicos ni siquiera por medirlos cuando regrese estuve también un tiempo en la oficina planeamiento y presupuesto y me preocupé por verificar y hacer seguimiento del cumplimiento del plan estratégico del MINSA, desde luego que están muy, muy afectados y no era por la Covid yo demostré que no era por Covid que desde antes estábamos, así.

Entonces, uno de los grandes problemas es que no tenemos en las autoridades esa visión de mantener en el aumento de emergencia un equipo que siga viendo lo normal, lo regular, lo estratégico se pierde todo se pone modo emergencia y no hay nadie que siga esa parte que al final nos va a dar contra la cara, entonces que ha cambiado el tener data el querer tener unificada pero no ha cambiado el hecho de que se tenga que sostener eso de tener un equipo permanente bajo una resolución que no se cambien a ese equipo operativo por lo pronto no se debe cambiar debe mantenerse y debe nutrir la nueva autoridad de cómo tienen que hacerse las cosas y los planes estratégicos cuales son los indicadores que tiene que él manejar eso es un punto.

El otro punto es, hay preocupación por hacer note una preocupación para hacer planes ya por a cualquier emergencia que antes yo veía que si nosotros no lo presentamos estaban ahí pero ahora hay gran preocupación porque eso tiene una connotación desde luego política, entonces hay una preocupación ya al plano cualquier cosa, ya hablan de cualquier inmediatamente lo quieren en Las cosas positivas. Lo otro que note a pesar de los cambios era que siempre se trataba de velar por este tema del alcance a la población, eso no se ha perdido el problema son las estrategias para ello hay preocupación del Ministerio por la vacunación se dice que no, pero yo que he estado ahí, sí, o sea no hablo de la vacunación Covid hablo de la vacunación regular hemos ido a supervisar hemos conformado equipos, hemos visitado las regiones o sea si ha habido un movimiento durante el 2021, o retomar ese tema, pero lamentablemente, pues este a pesar de que eso ha surgido después del Covid y se ha notado el digamos como estamos tan mal en ello, pues este la parte no ha ido de la mano con la parte presupuestal entonces ahí tenemos un problema las cosas negativas, que se ven después de la pandemia y siguen viéndose es que no hemos mantenido esta equipo de soporte de data, a pesar de que ya tenemos una herramienta es triste porque ya tenemos la herramienta y no se le saca provecho es más, ya ni siquiera es público la gestión de camas antes hasta fines del 2022 tenemos público la gestión de camas, la ocupación ahora ya no, entonces eso es algo que hemos retrocedido de lo que habíamos avanzado en la transparencia de información, no lo en líneas generales.

**I:** Eso a un nivel más macro del país **¿usted ha notado si a nivel regional se ha habido algún aprendizaje, algún cambio no se si a nivel de la gestión de las autoridades regionales, o tal vez de las DIRIS en los mismos establecimientos de salud?** ha tenido usted la oportunidad.

**E:** Yo diría que si ha habido un cambio positivo en algunas regiones, en algunas, sobre todo las del norte y centro, porque con toda esta movilización de Covid saben que tienen un potencial ahí oculto que pueden movilizar ante cualquier emergencia, de hecho las regiones del Norte por el tema del dengue también tenían esa potencialidad que se incrementó con el Covid, pero sus limitantes de siempre es que es asimétrico el crecimiento no todos se ponen, como dicen, la camiseta, y ahí vienen los resultados que a veces no son tan buenos, pero en líneas generales cuando yo supervisaba después del Covid veía que ya había otra mirada cuando hay una emergencia un poco más activos, un poco más participativos pero siguen las mismas debilidades de gestionar planes no pueden hacer un plan, porque como cambian tanto su gente, la gente que hizo el plan para Covid ya no en la misma y está una persona nueva en la cual hay que empezar del ABC para la matriz del llenado del plan entonces no hemos cambiado en eso, no hemos cambiado en eso y por mucho documento que sacamos del MINSA, no cambien a los equipos operativos los han cambiado, entonces seguimos ahí un problema grande a pesar de que tenemos gente, hemos mejorado en gente entrenada, gente que se ha capacitado en condiciones realmente muy muy duras sobre todo en regiones si acá en Lima era difícil en regiones era el triple pero esa gente muchas veces no

está siendo recontratada, termina su periodo de contrato y se va, no hemos aprendido en realidad a valorar el recurso humano.

**I:** Ok. Con la última pregunta, si se viera una pandemia ahora**, ¿cuál sería la situación donde del oxigeno?**

**E:** Bueno, yo creo que definitivamente estaríamos un poco mejor de lo que hemos estado en la primera ola, pero estaríamos igual de afectados que la segunda por el tema de que no tenemos al momento un mapeo del mantenimiento preventivo y correctivo de no solo de las plantas sino un mapeo en general de requerimientos de otros equipos, como los concentradores y los insumos. Y el otro tema es que mal, porque no tenemos equipos de arquitectos, ingenieros, técnicos biomédicos que puedan ver este tema a nivel operativo y en campo no los tenemos y mientras no lo tengamos vamos a retrasar la gestión de información y retrasar el actuar en la respuesta rápida que se tiene que tener en estos casos. Y si hubiera otra pandemia, probablemente sea mayor que la del Covid en el contexto de la más que la segunda ola, por la sencilla razón que ya los modulares no existen, los modulares están deteriorados, los espacios han sido vueltos a ocupar por otro tipo de patologías y la verdad que los hospitales que estaban dando sus últimos respiros antes del Covid posterior al Covid ya esos hospitales ya no dan más en toda la parte de lo que es funcional y todas las conexiones porque se han hecho una serie de Frankenstein en la parte de cañerías y tubuladuras y eso nos expone a riesgos y ustedes habrán escuchado los riesgos que han habido en otros hospitales en el oriente de explosiones y todo lo demás, entonces tenemos riesgo, porque no tenemos seguridad este índice de seguridad hospitalaria no ha sido vuelto no ha sido retomado entonces tenemos que ver la situación de los hospitales Post Covid que desde luego están más riesgo de lo que tenían Pre Covid porque han forzado su funcionamiento y sus últimos aliento siendo hospitales ya caducos en su estructura, en su funcionamiento.

Entonces esta próxima pandemia probablemente haya más conocimiento, más me recursos humanos pero la forma de dar respuesta probablemente sea igual o más baja la que dimos en la segunda ola por este tema no va a haber más gente afectada y no tenemos todavía la organización a nivel de otros profesionales que no son de salud y que tienen que entrar a tallar de verdad.

**I: ¿Que lecciones claves le dejan a usted todo ese tema del Covid?,** pero en relación al tema del oxígeno.

**E:** Bueno, todos los simulacros y simulaciones, como siempre lo hemos propiciado deben contar con una valoración de cómo estamos en nuestra respuesta a los pacientes que demandan oxigeno no solamente Covid, pacientes que tienen que operarse, pacientes que van a requerir que van a sufrir traumatismos torácicos, pacientes que van a hacer síndrome secundario por cualquier patología, entonces yo tengo que tener yo tengo que tener ese mapeo tengo que tener una información clara, y actualmente, pues no, no se tiene. Entonces yo creo que la información es muy importante y ver la calidad de información al respecto de qué escenario me voy a encontrar entonces propiciamos el conocimiento de escenarios como lección aprendida contingencial y de emergencia, propiciamos el que se tengan prácticas en simulacros y simulaciones en donde realmente terminen con un detallado de cómo está mi aporte, mi oferta de oxígeno; el otro aprendizaje es que tenemos que seguir luchando, porque estas carreras no medicas de ingeniero de sistemas, ingenieros biomédicos, técnicos, tengan un sitial dentro del manejo de las emergencias Inclusive en su momento conversamos con el Colegio de Ingeniero, colegio de Arquitectos, pero su presencia debe ser más fuerte, debe ser más contundente debe ser más política en ese aspecto para poder trabajar de manera alturada esto no es un trabajo solo del médico, la enfermera es un trabajo

de muchas profesiones y de gestores de riesgo perce planificadores y eso este cuando se trabaja así se mejoran las cosas; el tercer punto es el Estado no es un buen logístico definitivamente no lo es por diferentes razones que aquí he expuesto y estuvo bien que se delegara en alguien más el problema fue de que ese alguien tiene que tener esa capacidad de poder bajar al llano y entender que tiene que escuchar a la parte operativa aquí muy bien se hizo entregar en un gran Legado a través del ministerio de transporte a través de una sola fuente de, digamos, de gestión de presupuestal pero se hizo mal en no ir de la mano de manera periódica de manera regular con los equipos operativos.

Se pensaba que estos equipos de Legado, al dejar un ingeniero o un personal que velara por su modular era suficiente y muchas veces probablemente este responsable no elevaba nuestras quejas nuestras recomendaciones al más alto nivel, pudieron reunirse con direcciones, pero a este nivel tan particular de pacientes críticos en requerimiento oxígeno tenían que hablar con quien maneja los pacientes tenía que hablar con los directores de hospitales tenían que hablar con los jefes de la sociedad emergencia, y eso no se hizo. La línea de comunicación era a alto nivel y evidentemente se perdía una comunicación directa y el más sencillo ejemplo es lo que comentó que en plena segunda ola ante las dificultades que tenemos con el sistema de oxígeno y qué sé yo personalmente no me aguanté y pedí quién es el teléfono del gerente general ahí me valí de mí expuesto al ex directora general e hice un un zoom con ellos, con las jefas enfermeras que eran encargada del oxígeno en el hospital y con un representante del hospital que felizmente tuve también la suerte tener el apoyo del hospital a través de uno de los funcionarios y mira este, por favor, necesitamos la reunión yo la dirijo si quieres yo conozco esta gente y hay que exigirle tal o cual cosa, porque si no acá nos vamos a morir todos.

Entonces me dio el pase y en esa reunión pudimos aclarar algunos aspectos de redistribución de oxígeno y conocimos algunas deficiencias que ellos también tenían que no la sabíamos y que conociéndola por lo menos reduces el estrés no de que esa situación esté en todos lados y pues tenemos que ajustarnos así, nos guste o no, y eso ayudó mucho, entonces eso no se hizo en ningún otro hospital entonces todos los colegas vivían el estrés perenne que no llega el oxígeno que Praxair es malo que estamos mal entonces yo creo que ahí la comunicación a nivel operativo es mucho más no sólo es que ya mi director fue a hablar, no sólo es que ya el legado se va a encargar, sino es donde están las papas quemando ahí al nivel más bajo operativo ahí tiene que decantar esas reuniones mensuales o bisemanales para poder este tener una tranquilidad, bajar el estrés, el burnout y trabajar de manera más orquestada y sabiendo que se está haciendo lo mejor posible, porque al tener ese vacío de conocimiento de yo no sé si están actuando bien eso me genera un deterioro psicológico y no me permite trabajar adecuadamente, No.

**I:** Ok, Bien, doctora, Yo no tengo más preguntas, pero no sé si usted tiene algún comentario final, tal vez alguna pregunta que no hice, que usted considera que es necesario de decir o aclarar.

**E:** Bueno, solo tendría que decir al respecto que este tema puntual en oxígeno generó muchos retos en la pandemia, pero desde antes de la pandemia quiero que quede claro que ya teníamos problemas de oxígeno ya teníamos problemas de oxígeno y que hubieron muchas trabas que no se solucionaron en su momento, como el permitir la dispensación de oxígeno al nivel 93%, como la adquisición de concentradores de oxígeno y apoyo a quienes hacíamos esa gestión, como el tema de tener planes aprobados que tuvieran estas otras disponibilidades que si se estuvieron en previos desde el 2016 y 17 pero que lamentablemente pues no fueron tomadas en cuenta, yo quiero que quede eso claro que no es que por el Covid despertamos sino que desde antes del Covid por un problema de las heladas y friajes ya teníamos una idea más o menos clara, panorámica de las debilidades de nuestro sistema de salud en

cuanto a la disponibilidad de oxígeno, ya lo teníamos por lo menos mapeado; lo segundo tenemos que alguna manera cambiar la forma en cómo gestionamos los equipos al interno de las entidades públicas, porque si seguimos con este tema de temor a la corrupción y que no puedo movilizar los presupuestos de una manera más ágil vamos a seguir teniendo debilidades y vamos a seguir teniendo demoras y vamos a seguir teniendo problemas de denuncias y médicos legales en aquellos que como quien habla, tuvieron de alguna manera, por decirlo así el atrevimiento de gestionar cosas por la necesidad y los sustentos autogenerados y no de la manera que me lo exige el Estado eso es lo que yo tendría que decir al respecto una mayor protección a los funcionarios y un seguimiento, porque valgan verdades uno puede actualmente existe lo que se llama, con esto sí termino, la declaración jurada de intereses, es decir, uno declara hasta el cuarto grado de consanguinidad quiénes son tus parientes y a qué se dedican hoy en día yo puedo dar una declaración diciendo que se levanta el secreto financiero, entonces hay por todos lados una transparencia en la cual un funcionario puede hacer esos cambios y caramba puede ser seguido ese nivel para verificar que no está cometiendo ningún delito, sin embargo, lejos de hacer uso de eso uno cae en denuncias, caen pérdidas de dinero contratación de abogados y todo lo demás, y no puede digamos seguir adelante y desanima a muchos, gente competente trabajar en el estado precisamente por esos puestos entonces quienes asumen los puestos, pues no necesariamente son los que tienen las competencias sino son los que siguen las reglas del juego, reglas de juego que tienen que cambiar en aras de tenerlo mejor.

**I:** Ok, doctora. Muchas gracias. Entonces, en este momento voy a parar la grabación aquí.

**008 Entrevista**

Meeting started: 07/7/2023, 09:20 AM

Participantes: E, I

**I: Investigador**

**E: Entrevistado**

**I: Para comenzar hoy es 7 de julio del 2023, esta es una entrevista parte del estudio que está realizando la Universidad Cayetano Heredia que se titula Economía Política del Oxigeno Medicinal en el Perú. Solo para dejar el registro, el participante ha aceptado que grabemos la conversación para comenzar de manera contextual y que se quede en registro por favor nos comenta un poco acerca de su función con servicios en los que trabaja que tienen que ver o que estén vinculados con el tema del del oxígeno medicinal.**

**I: ¿OK,? Cuéntame un poquito acerca de cómo es que ustedes, durante la pandemia han visto este tema del oxígeno, las dificultades Que ha pasado, si bien es cierto se conoce de un modo a través de las noticias a través de lo que se veía, pero ya alguien estando en contacto directo con otros profesionales de la salud y también su misma labor. ¿Cómo ha sido este tema para usted?**

**E:** Bueno, cuando llega la pandemia al país el año 2020 identificamos que es un problema netamente respiratorio, problema en la cual vamos a tener necesidades de fármaco, pero no creíamos pues que era a una demanda altísima, ¿no? En su momento se revisó que había una serie de fármacos que podrían tener algún control o beneficio sobre esta enfermedad, pero poco a poco se fueron descartando, quedando finalmente quizás el único que tenía algún beneficio importante y que cambia el curso de enfermedad era el oxígeno medicinal. Nosotros teníamos información pues de que a nivel nacional solo contábamos con el termina cantidad de cámaras cuidados intensivos y que sobre eso había una también una cantidad limitada de oxígeno en su momento entonces no podíamos excedernos de un margen importante porque el oxígeno no podía cubrir todas las necesidades que en su momento tenía que tenerse en cuenta para poder recuperar pacientes crecieron los números de pacientes los casos grabes también se hicieron bastante frecuentes, las camas hospitalarias fueron cubiertas, el 100% de pacientes hospitalizados necesitaban oxígeno y aproximadamente un 50 a 60% necesitan camas en cuidados intensivos.

Entonces le contaba de que al ser el diagnóstico situacional cuando empezó la pandemia, encontramos de que el fármaco más importante para poder tener algún beneficio sobre estos pacientes era el oxígeno medicinal, nosotros identificamos este beneficio a medida, pues, de que pasaba con la información que teníamos de otros países, sobre todo en Europa y China, no con quienes nos reunimos de manera virtual para ver como iba ver el impacto aquí en el país se supo que el beneficio era solamente de eso, lo demás era quizás fármacos que pudieran caminar un poco la inflamación, pero era el fármaco más importante, entonces proyectamos nuestras necesidades pues a la cantidad de pacientes que vamos a atender y nos dimos cuenta, bueno

que con lo que contábamos en su momento no iba a satisfacer en un porcentaje importante a una cantidad de pacientes que iban a llegar a un hospital.

Tenemos nosotros restricciones de oxígeno, digamos propios porque no tenemos redes instaladas de oxígeno en los principales hospitales y no se utiliza también este insumo que es el balón, entonces ese era también una falencia importante, porque pues los balones necesitan recargarse, necesitan digamos tenerlo al alcance, la cantidad de balones también se necesitaba muchísimo ya incontables. Pero creo que lo más resaltante fue que no teníamos redes de oxígeno instalada suficiente en los hospitales es por eso, pues de que al acabarse el balón no había donde trasegarlos. Este paciente se veía afectado, este paciente desplazaba a otro se complicaba, necesitaba UCI, UCI estaba lleno, se incrementaba más cama UCI, necesitan más oxígeno, o sea, se creó un problema una gran bola de nieve que en su momento le informamos, pero pues el Estado insistía en que digamos necesitábamos más camas UCI, eso también fue un tema que nosotros Informamos en su momento, dijimos que tenía muchas limitantes, ya que el principal combustible para que trabaje un ventilador mecánico o trabaje un dispositivo que entre oxígeno es el oxígeno medicinal.

Entonces eso fue una limitante importante. Todo está documentado, pues los años que hemos vivido en pandemia, hora este necesitamos tener también alternativas que eran necesarias para salir de este gran problema que teníamos y se hablaron, pues de una serie de dispositivos portátiles, se habló de equipos que en su momento no eran de uso hospitalario pero se instalaron en los hospitales y pues que muy poco o nada suplieron las carencias de oxígeno lo más importante es que no teníamos, digamos, tanques de oxígeno medicinal en los hospitales lo suficiente como para satisfacer la demanda de tanta cantidad de pacientes. Creo que por ahí va las primeras impresiones en este problema si tuvieras de repente conversando se recuerda algunas cosas más que son importantes para ustedes.

**I: Doctor, pero este tema es como, o sea, si bien es cierto, se evidenció durante la pandemia esto damos la falta, la carencia de oxígeno era como una constante antes de o es que como no había necesidad, no se evidenciaba.**

**E:** Bueno, es cierto, ¿no? Creo que con lo que teníamos en su momento era suficiente para los pacientes que llegan al hospital. Por lo menos dale grandes acá en Lima me pareció que era ya estamos en una cantidad correcta. En los hospitales de periferia, en los hospitales regionales todavía no hay tanque de oxígeno medicinal líquido se trabajan con balones con redes un poco, digamos carentes de todas las condiciones de bioseguridad. Una UCI en una región o una provincia, trabaja con balones de oxígeno y cada cuatro o seis horas hay que estar reemplazando se hizo costumbre en ese momento y así han trabajado. Otra cosa era que las emergencias también trabajan con balones de oxígeno, quizás los únicos que tenían redes de oxígeno instalas eran las salas de operaciones que eran importantes y cuidados intensivos, emergencia trabajaban con balones eso era la situación inicial y con eso vivían, con eso era suficiente o se habían acostumbrado a un ritmo así de trabajo tal cual. Pero al ingresar una patología que era eminentemente respiratoria y con alta demanda de oxígeno obviamente esto cayó pues no,

entonces ya nadie te cambiaba un balón de oxígeno cada 6 u 8 horas sino cada 4 horas con lo que no tenían era insuficiente, estoy hablando de las zonas donde se trabajaban con balones y en los hospitales nacionales también en áreas de hospitalización en las cuales no había redes de oxígeno también se instalaron balones ahí también hubo problemas complejos, grandes. ¿Eh? ¿Cuánto aumentó la demanda de oxígeno? Se calcula que aproximadamente 10 veces de lo que teníamos en ese momento, de 5 a 10 veces y los rangos pasaban por todas las reservas que teníamos en el país.

**I: ¿Esta manera de trabajar respondía a una cuestión más normativa, más de parte del sistema mismo, una política o era más bien una cuestión que en ese de los trabajadores de salud de la forma en que se manejaba el establecimiento?**

E: Mire, creo que existe una mala costumbre de haber trabajado así, yo creo que tenemos todos, la normatividad necesaria como para trabajar bajo redes de oxígeno, o sea, pongo un tanque de oxígeno líquido y contentar a todos los servicios existe, ¿no? pero pues la inversión, las necesidades de cómo se llama de poder recargar esto. Eso también era un problema que en su momento cuando se habló con las empresas privadas en que provienen pues este oxígeno líquido les era muy complejo pues digamos ir a una zona muy distante con su camión de oxígeno y hacer la recarga y todo; por eso que quizás por costumbre lo único que se hacía era cargar los balones de 10 M3 y enviarlos a las zonas más distantes. Por ejemplo, creo que Iquitos trabaja en su mayoría con balones de oxígeno de 10 m3, no había una red importante de que no líquido y eso, pues este eso fue un punto importante como para para con nosotros caer en grandes problemas cuando se dio y vino en crecimiento las olas. Aparte que también me parece que otro punto que se dejó, pues mucha libertad fue que el oxígeno medicinal salga los domicilios, ¿no? se liberó el oxígeno, el oxígeno es un fármaco es un medicamento que requiere una prescripción médica que requiere una dosis requiere este titularlo dependiendo pues de la necesidad del paciente y no liberarlo, un poco más y lo vendían en un bazar, el oxígeno en plena pandemia y eso nos trajo problemas grandísimos también porque el oxígeno también tiene efectos adversos cuando se da una concentración muy alta.

**I: Entonces, por lo que me dice, era una cuestión más de costumbre. ¿Conoce usted si hay alguna o se publicó alguna norma, no sé si nacional o institucional que digamos oriente o describa cómo es que debería ser el trabajo por él que se tenía que hacer en los establecimientos de salud con el tema del oxígeno medicinal?**

**E:** Dentro de lo que es la infraestructura hospitalaria, la ingeniería hospitalaria, la provisión de oxígeno está indicado que se debe dar a través de redes, redes de oxígeno, ¿no? con un gran tanque de oxígeno líquido, porque es lo más limpio y puro que hay ese es el oxígeno medicinal que digamos está bajo medidas de bioseguridad, los otros tanques de oxígeno son medidas digamos que no cubren las expectativas de bioseguridad de poder brindar un paciente y este fármaco pues tiene que estar puro y tiene que pasar todas las digamos medidas adecuadas para evitar que este paciente a través de este producto adquiera alguna enfermedad o alguna

toxicidad, entonces no hay otro medio mejor que el oxígeno líquido, que es el digamos la forma más importante de poder dar oxígeno a un paciente que lo requiera.

**I: Usted sabe si las normas respecto de las políticas de oxígeno medicinal se revisan, se evalúan, se han ajustado o se vienen ajustando en este en este momento.**

**E:** Creo que todo a raíz de la pandemia nuevamente se está retomando recién nos hemos dado cuenta que el oxígeno es uno de los medicamentos más importante. Políticas claras no las tenemos, no, no hay este digamos formas de poder manejarlo, DIGEMID creo que solo titula la pureza y entiendo que esté por encima de 99% según la normatividad que creo también ha sido un error importante o para limitar la producción por otros medios con la producción del 93% es importante para nosotros por encima de 90, trabajamos muy bien con nosotros en UCI en emergencia, es una limitante que sea digamos de más alta concentración porque obviamente restringes a otros proveedores, ahora este creo que debería haber un punto de corte ahora más que necesario para hacer una política clara de del uso de este fármaco, ¿En qué momento deberíamos utilizarlo?. ¿Y cuándo? y ¿quién debería administrarlo? ahora uno va una a un centro donde hay oxígeno y te lo dan no sin ninguna receta médica y eso también es un tema que quería regulara raíz de la pandemia hoy día estamos viendo las consecuencias de utilizar oxígeno en casa son muchos pacientes que han ingresado con complicaciones severas pulmonares por altas concentraciones de oxígeno y eso es consecuencia de la pandemia de haber liberado este fármaco para un segundo domiciliar.

**I: ¿cuáles son como él o con qué complicaciones vienen los pacientes en este tiempo a consecuencia de esta esté uso inadecuado?**

**E:** Mire, cuando el oxígeno se da por encima de una concentración del 60%, se habla de que produce fibrosis pulmonar, entonces nosotros tenemos que tener indicaciones para que el requerimiento de de porcentaje de oxígeno sea menor a esto y si lo requiere por encima de 60, sea por un corto plazo. Entonces cuando nos hemos encontrado con pacientes en casa y han estado necesitando oxígeno, han estado en concentraciones del 100% en casa y sin titular no había ningún médico que lo titule o el personal de salud que iba veía que saturaba más de 90, 95% lo dejaba tal cual no lo regulaba no lo titulaba.

Están llegando hoy en día a los hospitales pacientes con fibrosis pulmonar como consecuencia de la de la inflamación ocasionada por el COVID y por la alta concentración de óxido que recibió en su momento y eso lo indicamos, no se podía liberar la presión de oxígeno a nivel domiciliario tenía que ver alguna indicación de titulación para tratar de que haya complicaciones posteriores.

**I: Usted me dice que eso lo indicaron, lo indicaron como colegio, como institución o establecimiento salud**

**E:** Como sociedad cuando se habló acerca de que se estaban usando oxígeno en casa, de que había grandes colas para la venta de balones de oxígeno, que se prácticamente los costos de un balón de oxígeno se elevaron 10 veces en su valor normal y pues se creía que colocando un balón

de oxígeno acostado y sin titular era más que suficiente para que el paciente se establezca. Entonces sí se habló acerca de las consecuencias de utilizar de manera libre este fármaco.

**I: ¿Cuándo es que dieron estas opiniones recomendaciones?**

**E:** Cuando el Gobierno prácticamente el libro, o sea, no hubo ningún control acerca de la situación del oxígeno medicinal, cuando había esas grandes colas en las cuales se compraba su balón uno, bueno también en ese momento no había camas hospitalarias como para atender a todos y tenían que ir a su casa.

**I: ¿Qué otras recomendaciones dieron ustedes? saben más de este tema del cuidado**

E: Las recomendaciones eran pues que también este si se administra en domicilio tenía que ser bajo control médico y nunca hubo un control médico al respecto, no había luego se habló de que tendría que tener también vigilancia de parte de un personal de salud itinerante que vaya por ahí para regularlo y se habló también de que era mejor tenerlos los suministros de oxígeno a nivel de establecimiento de salud ósea en un primer nivel en un segundo nivel para tener un mejor control que eran los únicos lugares para dar este este fármaco a través de concentradores o balones porque era la forma más correcta me parece, no liberarlo a domicilio no. Pero en su momento no había ni primer ni segundo nivel, o sea, también estábamos y no había una manera en la pandemia pasó todo nuestro nivel de control.

**I: Ya ahora, viendo en perspectiva lo que ocurrió y lo que en ese momento se tenía como una norma ¿usted ve que son suficientes las políticas que tenemos para el oxígeno en este momento? ¿Que haría falta? ¿cómo se tendría que ajustar?**

**E:** Ciertamente lo que yo percibo del Ministerio de Salud fue que en su momento los que se sientan en las mesas de trabajo son personas eminentemente que te traduce en papeles o copian de otras directivas lo que ellos creen por conveniente, no recogen la experiencia de la persona que está al lado del paciente y cuando se da recomendaciones de manera técnica ellos lo orientan a una política o a decisiones políticas más que decisiones técnicas eso es lo que yo percibí, porque si hablamos de otras cosas, por ejemplo, de ahí la Ivermectina también se utilizó de manera indiscriminada y se formó un comité de expertos, las sociedades científicas que eran las que en su momento manejaban o estaban al lado del paciente nos pronunciamos para que este producto no se no se deba dar, sin embargo, el Ministerio de Salud lo liberó y eso también incrementó más la cantidad de pacientes que llegaban a los hospitales.

**I: Entonces por lo que entiendo usted mira como las normas son como un resultado más de una decisión política o es más bien una reflexión técnica**

**E:** No, es una decisión política, no nos podemos salir del marco que ellos creen porque o sea siempre están a la defensiva, uno trata de poner digamos normatividades y que vayan con lo que sea a nivel internacional, pero dice hay que ver la realidad del país no está para eso, nos van a denunciar se están cuidando sus espaldas, definitivamente ahí prima decisiones políticas que técnicas.

**I: No sé si usted en algún momento como parte de su trabajo en los establecimientos de salud o en la sociedad ha mirado un poco lo que es el tema del financiamiento del tema del oxígeno médico.**

**E:** Me enteré acerca de cómo se manejaba esto porque nos metimos de plano para decidir qué más podíamos hacer tengo entendido que el costo de 1 M cúbico de oxígeno el precio real es 8 soles, 8 a 10 soles y que en su momento lo vendían el balón de 10 m3 hasta 3000 Soles, o sea el metro cúbico estaba en 300 soles una cantidad importante en cuanto a costos a pesar de que el oxígeno medicinal líquido podría tener un costo un poco más alto es digamos, inocuo y saludable para el paciente, instalar una red de oxígeno hospital no es muy caro a largo plazo le va a dar muchísimo beneficio porque vas a tener justo de donde puedas administrar y te olvidas del balón de oxígeno, el balón de oxígeno en un hospital requiere, piden una recarga que denota transporte, que un técnico lo lleve, los costos que no los vemos están muy por encima de instalar un tanque de oxígeno líquido y sus redes y nos indican más que recargarlo cada cierto tiempo y eso no requiere otro tipo de asistencia. Eso fue lo digamos, el costo beneficio se planteó que debía haber oxígeno por lo menos en centro de salud que tenía más 100 camas para arriba que era lo necesario, para evitarte pues recargas innecesarias con los balones y otra cosa. En su momento mi tema era más que toda asistencia y médico quizás me entere de algunas cosas por la información que nos llegaba.

**I: En cuanto a la sociedad, ¿ustedes en algún momento no sé si como grupo, tal vez alguna persona más interesada en el tema digamos, ven un poco la parte de la rendición de cuentas sobre la seguridad del oxígeno médico a nivel del sistema de salud?**

**E:** No, como sociedades o fines son otros, son académicos, equipos científicos, no vemos ese tipo de cosas

**I: ¿Quiénes son los responsables de regular el tema del oxígeno medicinal?**

**E:** La DIGEMID

**I: ¿Solo lo DIGEMID? pero a través de, por ejemplo, el suministro clínico propiamente los establecimientos de salud ¿hay algún seguimiento que hace la DIGEMID también allí?**

**E:** No, no, lo que pasa es que recién se han dado cuenta que el oxígeno es un fármaco antes manejaban o era como un insumo más hospitalario, o sea, como una red de agua, como una red de otro servicio básico que se le da al hospital y pues cualquiera lo podía usar o manejar, pero la pandemia se enseñan muchísimas cosas, debería haber una dosificación, la forma de administración, equipos para poder administrarlo. Entonces cada hospital ya se maneja de acuerdo a la parte clínica no hay digamos una oficina que controle, el oxígeno llega a un hospital lo trae la cisterna, lo recargan al tanque de oxígeno líquido y de ahí el que prescribe el médico, la enfermedad también lo maneja, pero no hay un ente regulatorio dentro del hospital que lo pueda manejar o controlar o supervisar.

**I: En su opinión, ¿cuáles serían las áreas claves en las que debería centrarse cualquier nueva política para el tema del oxígeno? ¿Y en qué debería fundamentarse? No sé si más en la cuestión técnica tecnológica, más en el tema de reforzar todo el sistema de salud.**

**E:** Bueno, miren con la pandemia se han improvisado muchas redes de oxígeno creo que ahora es el momento de hacer unas redes formales del oxígeno, tú vas a los hospitales y ves que estás saliendo tubos de un cuarto esperando otro han instalado red, pero me parece que ha sido de la manera más. en su momento se necesitaban hacerlo de manera rápida, pero ahora pues ya estamos en un momento en la cual debemos modificar nuestros hospitales y hacer redes formales de oxígeno, las emergencias deben tener puntos de oxígeno deberíamos tener puntos de contención del oxígeno, olvidémonos del balón, por favor, el balón nos ha traído demasiados problemas porque si bien es cierto se vio como una forma de administrar, el tema de la recarga, el tema de estar viendo trasegaba en situaciones de desastres nos ha traído demasiado problema , no sirve para para conectar un ventilador porque también te demandan mucha muchos recursos Humanos y te complica el paciente porque al hacer el cambio del paciente deja de recibir oxígeno y entonces ahí se nos han muerto varios pacientes.

Creo que ya deberíamos modernizar todo lo que es nuestro sistema de administración de oxigenoterapia por red a nivel hospitalario, hay hospitales que tienen más de 100 años y veo redes que están, digamos al aire libre están por ahí entonces Imagínate eso no debería existir en el hospital nivel 3 debería ser una red confiable, una red segura, porque también puede ocurrir accidentes.

**I: De lo que está hasta ahora, digamos, de la forma en la que se está trabajando con el oxígeno que ¿cree usted que debería mantenerse? Bueno, usted ya me ha comentado que debería la parte de la instalación es como que hay que cambiarlas, pero ¿qué si funciona? En todo el proceso de llevar, de hacer llegar el oxígeno a la paciente.**

**E:** Qué funciona bien, tiempos en los cuales no estamos en situación de desastre todo parecía que caminara normal ahorita estamos tranquilos, creo que la modernidad tiene que ir a todos los hospitales no podemos seguir así. Me parece que el oxígeno también viene de una empresa privada que proveen a nivel nacional, eso también para mí fue una sorpresa porque pensé que por ahí había alguna institución nacional que podía también darnos la mano, pero no fue así. Creo que, por ejemplo, debería establecerse una normatividad hospitalaria que por encima de las 50 camas hospitalarias que va a tener cualquier centro de salud debería tener un tanque de oxígeno líquido, debería tener ya de diferentes metrajes no se si de 20000 m3 o 10000 pero ya instalado, donde hay un camión que lo pueda recargar.

Te vas a regiones y provincias y todavía siguen con balones; otra cosa es establecer protocolos de manejo de oxigenoterapia en pacientes ese también es un tema que no se maneja hasta ahora, que es muy libre y que si se da a nivel domiciliario porque actualmente hay pacientes en casa que reciben oxígeno, hay pacientes por temas de enfermedad crónica, pero están bajo control médico, eso está muy bien. Debería de haber una política clara también de

administración de oxígeno domiciliario, cuáles son los beneficios, los riesgos y todo eso debería salir en normativas. Creo que es lo que se me ocurre en este momento.

**I: ¿Cuáles fueron los retos para usted desde ya? Yo sé que el tiempo nos permite ver cosas diversas que en su momento por la el tema mismo de la urgencia de todo lo que se vio durante la pandemia, los momentos más críticos de la pandemia nos muestra una perspectiva más amplia ¿cuáles podría usted decir que fueron los retos con respecto del oxígeno durante la pandemia?**

**E:** Bueno tratar de que ningún paciente se muera por falta de oxígeno eso fue un reto importante por todo, Oxígeno líquido digamos que se prohibió a través de estos salones de otros equipos que también prohibió y concentradores. Mi preocupación fue siempre mirar alrededor del Perú y yo preguntaba de Ecuador a Chile a Colombia porque teníamos amigos ahí que trabajaban en UCI y su principal problema era oxígeno y ellos nunca tuvieron problemas y cuando se habló de sus redes y sus redes eran pues tenían más allá de 5 o 10 años, habían sido renovadas tenían provisión de oxígeno hasta en el último rincón de tu país y si tenían necesidades de balón era una cosa extraordinaria, el balón ya es un insumo que esta retirado de los hospitales de primer mundo solo se permiten a nivel domiciliario. ¿Qué es lo que deberíamos hacer? ver pues recoger las experiencias de los países como llegado a esto es importante saberlo porque hasta ahora pasó la pandemia en el país no ha cambiado mucho las cosas, seguimos igual que antes en la pandemia

**I: Durante la pandemia se hizo la instalación de plantas en diversos establecimientos de salud gracias a donaciones en fin a compras que también se hicieron ¿sabe usted cuál es la condición, la situación en la que están estas plantas de oxígeno? ¿están en funcionamiento? ¿se llegaron todas a implementar o no? ¿Que en qué situación estará?**

**E:** Y eso es un tema que estuvimos en contra, las plantas del oxígeno no son para hospitales grandes las plantas son para el Centro de Salud para lugares donde no puede llegar el oxígeno líquido, fundamentalmente a ese lugar. En mi hospital han instaló una planta de oxígeno está apagado no se necesita una red distinta a la red de oxígeno líquido, ósea no sentido instalar una red paralela porque ya la red de oxígeno líquido es suficiente. Digamos, fueron donaciones pues producto del desastre que estamos viviendo se aceptó y todo, pero perspectiva no tienen utilidad a nivel hospitalario, creo que la ubicación más exacta es un hospital nivel uno, en Centro de Salud ahí si deben de estar porque ahí sí va muy bien ya que no llegan la recarga de oxígeno a través del tanque , creo que lo que pasa es que la desinformación también están grande que en su momento, pues creían que esto reemplazaba un tanque de oxígeno líquido, no es un oxígeno puro y el tanque producto de esto, es un oxígeno, digamos que no pasa todos los criterios de bioseguridad que lo instalaron y nunca funcionó.

**I: Para entender mejor está usted diciéndome que las plantas que se instalaron en los diversos establecimientos de salud no llegaron a funcionar y no eran los digamos los que se necesitaban en ese momento**

**E:** A lo mucho se hizo que era una planta de oxígeno que proveía oxígeno solo para 10 pacientes, para 10, nada más.

**I: ¿Aunque la planta de oxígeno tengo una mayor cantidad de producción de oxígeno?**

**E:** Sí, depende

**I: porque hay como de varios decibeles**

**E:** Sí, pero el que nos habían instalado era promedio de 20 y era para 10 pacientes nada más y hacía una bulla inmensa, parecía una fábrica que estaba instalado dentro de un hospital y el sonido no era muy cómodo para nada, absolutamente, no te daba tranquilidad, los pacientes que estaban recibiendo oxígeno se quejaban de la bucha como te digo no es para para un hospital nivel tres, no va.

**I: ¿A partir de qué niveles de establecimiento y para dónde servirían esto estas plantas? Porque fueron muchísimas tengo entendido que son casi 400 plantas que están en todo el país.**

**E:** Sí preguntáramos si están operativas en este momento, no están operativas.

**I: Y no están operativas porque precisamente por el tema de la infraestructura o hay alguna otra razón por la que no están operativas.**

**E:** Porque prefieren los que tenían plata y han utilizado el balón de oxígeno prefieren el balón de oxígeno en este momento.

**I: ¿Y eso a que se debe?**

**E:** Se debe a que de repente están acostumbrados a un ritmo de trabajo en la cual no haya bulla, el ruido es ensordecedor, es terrible y no da comodidad en el trabajo entonces de repente lo tiene como una reserva en caso de ser otra vez este tipo de desastre, pero más nada creo yo si se estratifica a dónde debería ir, debería ir a un Centro de salud nivel uno, muy periférico, ahí debería estar ubicado en el uno o dos a lo mucho, en los hospitales regionales o hospitales nacionales tienen una red ya establecida de oxígeno líquido no van con este sistema de administración de oxígeno, de planta.

**I: OK, entonces eso nos lleva a la necesidad de tener oxígeno líquido, pero ¿qué tan probable ve usted que eso sea posible? ósea entiendo que puede estar como la necesidad, pero una situación en un país o un contexto en el que vivimos como es del del Perú, ¿qué tan probable es que esto se pueda instalar?**

**E:** A la larga es mucho más económico que otra forma de instalar oxigeno yo como le digo hay que recoger las experiencias de los países vecinos que tiene la misma geografía nuestra, que en los hospitales más distantes tienen oxigeno liquido las plantas, como lo dijeron, los tienen en un lugar muy recóndito donde no llegan el sistema. Esa es una forma de política de poder administrar oxígeno a nivel nacional.

**I: Ok, pero en un contexto de pandemia, un contexto de desastre natural y una crisis que presente ¿cuáles serían las mejores opciones o alternativas para responder a una necesidad como la que tuvimos durante la pandemia del COVID-19? Si digamos hay una necesidad también de equipos o de oxígeno por una cuestión de no sé otra epidemia que tenga complicaciones respiratorias, en fin.**

**E:** Bueno, este yo creo que los que proveen oxígeno ya deberían, estoy hablando de las productoras como helth products y el otro ya deberían tener su reserva de producción, deberían tener unas plantas digamos produzcan más oxigeno líquido como una forma de reserva creo que ahí sería una forma de solucionarlo o el Estado quizás que pueda tener una planta de oxígeno líquido en la cual solo en situaciones quizás podría ser la una alternativa. Yo creo que deberíamos mejorar todas las redes es una forma más limpia de entregar oxígeno a un paciente es una forma que te demanda menos recurso humano, es una forma también de tener segura nuestros equipos biomédicos, también es porque hay mucha pureza ahí y creo que a la larga de repente pareciera que fuera un poco más caro pero nos va a dar mucha tranquilidad a nosotros que trabajamos con esto, de no estar en la expectativa que se me acaba el balón, que la planta ya dejó de producir más, se necesitada para el paciente número 11 o 21 y ya no hay y eso es un tema, deberíamos tener esta reserva de oxígeno a nivel nacional luego de lo que ha pasado con la pandemia.

**I: Para tema de la generación de oxígeno, ya sea por estar por el oxígeno líquido o por el tema de las plantas se requiere además de infraestructura, además de personal de salud que digamos controle, verifique, administren a un equipo para el mantenimiento, ¿para el mantenimiento yo no sé de qué tan complejo puede ser hacer el mantenimiento para este tema del oxígeno líquido a con respecto del oxígeno de la producción por las plantas, por las plantas, entonces? ¿Cuál es más complejo, donde se requerirían personal más especializado o un poco para mirar también toda esta parte del recurso que se requiere para contar con uno u otro sistema?**

**E:** No, definitivamente lo oxigeno líquido no te demanda Recursos Humanos, solo viene el tanque lo recarga y te olvidas. Este la planta de oxígeno, sí, pues, necesita que haya una persona esté permanentemente de ahí porque hay subidas y bajadas en concentración de oxígeno, es un motor que está prendido permanentemente y que extrae el oxígeno del ambiente y es un proceso ahí dentro de sus equipos y concentrarlo en unas especies de balones para los pacientes eso demanda pues recurso humano, demanda mucho mantenimiento me parece que por ese lado que pareciera económico se complica la situación. Las normas internacionales indican que la habitación de oxígeno liquido es mucho mejor que cualquier otra forma.

**I: OK, ahora de lo que usted ha podido ver desde de los diversos sectores implicados en el tema, no solamente de manera funcional en el establecimiento de salud sino toda la infraestructura a nivel de las instituciones que se armó para responder a esta necesidad de oxígeno que había en el país ¿qué funcionó? ¿que no funciono? ¿cómo se hizo eso? ¿Cuáles fueron los obstáculos que se tuvieron? Porque se vieron implicados diversos actores, no solamente MINSA, DIGEMID**

**estuvo la SUNAT por el tema de las importaciones, estuvo también en algún momento PCM todos los sectores involucrados.**

**E:** Bueno la empresa privada contribuyo bastante, muchísimo, mucho creo que es más importante y fue más rápido que el mismo Estado las trabas burocráticas también un poco que se limpiaron en el camino, pues porque había presión, pero este definitivamente tiene que ver una persona que lidere esto no o una institución o una oficina, que yo no sé si el Ministerio de salud se hubiera informado luego de esto. Funciona siempre dentro de una situación de desastres por eso me pareció importantísima labor de la empresa privada porque no solo dio oxígeno sino también dio equipos para poder trabajar en pacientes graves y eso fue mucho más rápido que el Estado. Mire le comento estamos 2023 hasta hace 6 meses han estado llegando ventiladores mecánicos que fueron encontrados en 2020 entonces

**I: Se refiere a que recién están llegando**

**E:** Si recién la última cantidad de ventiladores han llegado en diciembre del 2022 a mi hospital y los 5 restantes porque llegaron en simultaneo. Han llegado 50 ventiladores que fueron comprados por motivo de la pandemia, es una traba inmensa en cambio cuando se habló con empresa privada para que nos doten de ciertos equipos no pasa ni dos ni tres meses y ya estaban llegando las donaciones de ellos, ahí uno ve alcance pues ya burocracia que se da y el tema de digamos de compra de gobierno al gobierno de año pasa por una serie de filtros, creo dentro de los operadores del Estado de control y unos y otros digamos del control sobre otros, lo que hay muchas oficinas en vez de ser ayuda era una traba importante para nosotros también habían equipos que no salieran de ADUANAS que estaban ahí.

Y pacientes seguían muriendo un tema complicado, pero creo que lo bueno, yo trabajo dentro del Estado y trabajo en la parte privada y siempre he visto a la parte privada como una forma mucho más eficaz de poder resolver problemas y se demostró la pandemia. ¿La iglesia también jugó un papel interesante?, ¿no? La iglesia también tiene un peso entre los principales, digamos empresarios del país, también dentro de sus relaciones y manera impresionada al Estado también ayudaron, llamaban y decían que necesitábamos nosotros nos hemos reunido de manera virtual con mucho de ellos hemos tratado de orientar también porque en cierta manera, como no había un tratamiento específico, se compraba lo que te decía una otra persona y había también una forma de malgastar el dinero. Pero bueno, se concluyó que el oxígeno era lo más importante en ese momento.

**I: OK ¿Cuál es la situación hoy respecto del tema del oxígeno? usted ve que se haya aprendido algo, que ha cambiado algo. Sé que lo he ido como comentando durante la conversación que estamos teniendo, pero como para ir resumiendo.**

**E:** Bueno, mire, yo lo veo como el año 2019 el país ahorita, lo que pasamos el 2020 o 2021 no ha servido absolutamente nada hemos vuelto a lo anterior. No ha habido incremento de camas hospitalarias, camas UCI, yo veo siempre de tanque de oxígeno líquido ahí un hospital uno chiquito, que hubo en la pandemia, no ha habido un recambio de eso, yo creo que es hora

después de intercambiar todo esto, se utilizó muchísimo debe haber agotamiento de estos equipos ya debe haber pasado todo un proceso pues en la cual se tenga que renovar. Estoy hablando de oxígeno, luego renovar las redes de oxígeno que es improvisar en su momento o ponerlas de forma definitiva y bueno desinstalar estas plantas que tienen ocupando un espacio importante dentro de los hospitales. Creo que ayudar y llevarlos a otros centros o redistribuir u ordenar.

**I: Pero eso no le están tomando a nivel de la dirección de los establecimientos de salud, lo están como mapeando porque es evidente para ellos, los equipos, las plantas están allí.**

E: Pero no se utiliza.

**I: No, entiendo que no es utiliza, pero digamos a nivel directivo, a nivel de los gestores del hospital ven que no se utiliza, ven que se podría hacer algo o trasladarlo que no le van a dar uso. Sabe usted si están haciendo alguna gestión**

**E:** No, eso es un tema que debería manejar a nivel de Ministerio de Salud sincerar en este momento. ¿Cómo está la proyección de oxígeno a nivel nacional y donde se necesita? para distribuir estos equipos que están ahí. Veo que en el Ministerio de salud no hay quien lidere este tema tampoco son cosas que suceden, pues también con tanto cambio que hay en Ministerio de salud que se cambia cada rato los ministros y no creo que se pueda lograr una política de corto plazo ese un tema también pero bueno, nadie se hace problema recibió su donación está tu planta ahí mal que bien, habría que dar una mirada a todos los hospitales como están, en qué situaciones.

**I: Se lo había preguntado de una forma diferente, pero en este momento me gustaría saber si tuviéramos una pandemia ahora ¿cuál sería la situación del oxígeno medicinal en el país?**

**E:** Haber no tengo información de cómo están las reservas de oxígeno líquido si estas empresas privadas que nos venden han mejorado su producción o tienen alguna reserva o han empleado un plan en eso me parecería fundamental. Luego a niveles infraestructura hospitalaria no hemos crecido absolutamente nada, poco; le comento algo, no se acuerda el hospital de ATE que fue, digamos, destinado para COVID y se perturbó por la pandemia es un hospital fantasma, un hospital que estaba lleno de aparatos ahí y pues se abrió, pero cuando fuimos a revisarlo dijimos haber prueba este ventilador mecánico y nos dijeron que iban a trabajar con 200 ventiladores mecánicos, hay 200 ventiladores.

**I: ¿Cuándo hicieron esa visita?**

**E:** Eso fue en marzo, marzo de ese año 2020 ya cuando empezaba la pandemia. Mira hasta dónde llega la torpeza del Estado dijeron vamos a abrir 200 camas UCI y ahí se va a manejar todo el COVID que hay que tanta cosa, que los equipos, que el personal. Haber vamos a prender un ventilador, prendieron y la alarma de oxígeno sonaba, le digo, tu oxígeno ábrelo haber llamada por teléfono al estamos en mi tercero o quinto piso llamadas al sótano abre el oxígeno bueno, abrieron oxigeno yo le digo, y ¿cómo está tu sistema de oxígeno? no abajo tenemos 10 balones

en una red abajo y de ahí vamos a probar y veo alrededor porque estábamos en quinto piso y no había ningún tanque de oxígeno líquido que necesitan.

Cuando, pues nuestros políticos hablan y dicen cosas pues hay que tener bastante pie de plomo poder saber si las cosas que piensan o dicen van a correr con la realidad, no había un tanque de oxígeno líquido y le digo en ese momento estaba el viceministro esto no puede funcionar así, no funciona para nada, necesitamos por lo menos 2 tanques de oxígeno, por temas de no caer mal, creo dos semanas lo instalaron, pero sí no hubo una visión clara de cómo se manejan las cosas. Ahora si tenía red, tiene red, pero no tenía su tanque de oxígeno líquido. Solo como comentario era esto, pero lo demás es de que necesitamos, pues saber si tenemos reservas de oxígeno, mejorara lo que se implementó ya se desarmaron muchas áreas temporales también y carreras ciertamente políticas y ver cómo se va a manejar, cómo se va a redistribuir los recursos que se utilizaron para trabajar en hospital y que no se están utilizando en este momento.

**I: Usted pudiera y revisar también dos lecciones claves que podría compartirnos acerca de lo que vivimos, de lo que se fue en relación al tema del oxígeno medicinal como profesional de salud, como parte de la sociedad.**

**E:** Mire yo creo que hay que prepararnos de acá para adelante para situaciones como estas, hace muchísimo tiempo nosotros vivimos muchas alertas y de enfermedades respiratorias que vienen del Asia recuerda el SARS el MERS hablando de virus que se meten a los pulmones y ese tipo de problemas, pero nunca llegaron al país nunca y nosotros creíamos que esta vez no iba a pasar lo mismo iba quedar en Asia y no iba a llevar para acá. Pero bueno, yo creo que de acá para adelante tenemos que tomar muy seriamente las cosas creo que deberían. Mi primera reflexión es que tiene que cambiar todo el sistema de salud ya eso se ha dicho, eso lo dicen todos también y no se hace. Y que el otro punto es el recurso humano, el recurso humano si no hay recurso humano, por más que te instalen redes de oxígeno y tanta cosa, no hay quien pueda atentes a los pacientes. Tomar muy seriamente esto y deberíamos cambiar todo lo que es a nivel del sistema de salud y tener en cuenta muchos Recursos Humanos que creen pues que van a poner una cama UCI vamos a poner 5000 y no piensan en el recurso humano que debe haber para esta cama, creo que se toma muy a la ligera este concepto de opiniones acerca de cómo sabía manejar esto nunca consultan al persona que está trabajando el día a día en el campo como nosotros y que debe dejarse politizar, pues y más que todo y en base a la normatividad técnica mejorarse todos los procesos de interés en salud.

**I: OK, no tengo como más preguntas ahora pero no sé si usted tiene alguna otra reflexión acerca de lo que hemos conversado, algo que tal vez no le haya preguntado y que considere que es importante señalar aquí.**

**E:** Bueno, más bien le agradezco la oportunidad no me hace recordar muchas cosas críticas de hace como tres años y pues este tratando de revisar todos mis archivos que tengo ahí para ver acordarme de muchas cosas, pero las cosas que tengo en el 2019 antes de la pandemia estamos viendo hasta lo mismo ahora

**I: Ósea no ha cambiado nada**

**E:** No ha cambiado nada es una lástima.

**I: Ahí me queda una inquietud Y es algo que estoy como escuchando también de otros entrevistados, es una cuestión más de índole de estructura del sistema o es una cuestión que está más vinculada con las personas con el recurso humano, con las costumbres, con la forma de ver el trabajo que se hace porque o sea, para cambiar la casa no solamente se cambian las paredes y estructuras, sino también hay cambio personal y entonces me gustaría saber por dónde es que se debería ir o hay que ir de manera paralela o no sé cómo ve usted ese tema, porque su comentario lo escucho también en otras la persona**

**E:** Claro, yo creo que debería haber y tener en el paralelo y por lo mismo. Yo creo que la mayoría de mis colegas médicos que todos cambiaron sus conceptos acerca del manejo de lo que es el tema respiratorio del oxígeno ha cambiado muchísimo. Creo que van a salir nuevos conceptos acerca del manejo de esto, ya se han publicado muchas experiencias en el uso de oxígeno y la administración de oxígeno ya no es solo necesariamente el ventilador mecánico, sino las los dispositivos de alto flujo y otras cosas que nos han ayudado bastante bien.

Y en cuanto a la estructura, eso sí, es capaz de hacer control, pero bueno las sensaciones mías son que deberían dirigirnos enteste pensantes y que tengan una política clara de cómo se debe manejar esto un segundo virus u otra pandemia nos va arrasar nos va a complicar más las cosas y hay que estar preparados para esto también porque este mundo es bastante complicado hoy en día con las instrucciones por virus que están bastantes fuertes.

**I: Sí, una de las cosas que también se viene alertando hace bastante tiempo es la posibilidad de que en el Perú tengamos un gran terremoto, por ejemplo, que es una situación que crisis compleja ¿estaríamos como preparados para una situación de esta porque en este momento también habrá la necesidad de contar con equipos, contar con oxígeno para los pacientes que por alguna cosa tenga alguna complicación, o sea parte del mismo resultado del terremoto? ¿Cómo se trabajaría que se haría para estos casos?**

**E:** Esta pandemia ha renovado nuestros equipos, comentaba que hace unos 6 meses han llegado nuevos ventiladores, hoy en día la capacidad de ventiladores mecánicos a nivel nacional está perfecta, ha reemplazado los ventiladores que nos ha cambiado lo que pasa que ahora hay mucho ventilador que no se está utilizando es el momento de poder mejorar nuestra estructura hospitalaria, deberíamos no nos va a salir muy caro en el hospital, ya que los equipos están. Uno frente a un desastre pues como la que te mencionan y estar muy preparado y nuestros hospitales pues están bastante viejitos, están bastante, creo que no puedan resistir un sismo de esta magnitud eso también es una cosa que también pasa por la cabeza, porque cada rato nos dice acerca de qué preparados para un sismo o terremoto acá en Lima y veo pues de que la situación está bastante complicada si fuera acá, recuerdo que cada vez que desastre una región o provincia distante del país todo lo refieren a Lima, todo lo refieren a Lima, porque Lima obviamente está más preparados para enfrentar esto, pero si el terremoto se diera acá quien nos va ayudara a

nosotros Arequipa, Piura no creo, o sea no tenemos esa capacidad o una alternativa de que alguna región nos pueda ayudar a Lima, entonces Dios no quiere que suceda esto pero va a ser imposible predecirlo pero sí se viene pues un desastre que sí suceda un sismo o terremoto acá en Lima nos complicaría todo.

**I: OK doctor, muchas gracias por participar en este momento voy a apagar la grabación. Gracias.**

**009 Entrevista**

Meeting started: 7/7/2023, 20:22 pm
Participantes: E, I
**I: Investigador**
**E: Entrevistado**

**I: Muy bien, buenas noches padre, y antes de comenzar de manera general, tengo que decir que hoy es 7 de julio del 2023, esta es una entrevista como parte del estudio que la Universidad Peruana Cayetano, lamentablemente en la investigación que se llama economía política del sistema de oxígeno medicinal en el Perú. Debo señalar que el entrevistado ha aceptado que grabemos la conversación que vamos a tener y como para ir ya comenzando propiamente con la entrevista me gustaría que, por favor, nos comente, usted de manera general, como para tener una referencia de con quién estamos hablando ¿cuál era su función o su labor en el tema de los servicios de oxígeno que se tuvieron durante la pandemia? si usted había tenido la oportunidad de tener una actividad similar o papel antes si todavía está en este momento involucrado en el tema un poco para tener el contexto básicamente.**

**E:** Ya sí. Bueno, muy buenas noches. Solamente soy sacerdote ya con 28 años de servicio y trabajando en la DIOSIS de una región en el Peru y si hemos tenido alguna experiencia, no solamente con esto del oxígeno que tuvimos ahora por la emergencia y la pandemia sino también en otras comunidades pues de alguna manera buscando que por lo menos el gobierno se involucren las situaciones más álgidas y problemáticas de una población como, por ejemplo, el agua potable y también el ayudar en los caminos en las carreteras. De alguna manera hemos tenido cierta participación en este tipo ser más bien una ayuda para los gobiernos locales o regionales siempre hemos estado en esa en este rubro y ya con lo de la pandemia pues hemos tenido la posibilidad de ayudar muchísimo más sobre todo con el oxígeno aquí en Cajamarca, en la ciudad de Cajamarca y en la región eso se podría hacer un poco eso así en general.

**I: Ok, En qué momento usted, entiendo que su labor ha sido de varios años atrás interviniendo en labor social para que algunas obras se realicen ¿en qué momento usted se involucra propiamente con el tema del oxígeno medicinal a partir de cuándo diría usted que comenzó esta labor?**

**E:** Sí, eso fue bueno, empezando la pandemia aquí en Cajamarca.

**I: ¿Desde marzo?**

**E:** En primer lugar, tenemos que hacer un poco el contexto en Cajamarca estábamos a punto de recibir un premio por parte del Gobierno porque no había nada, todo estaba muy y las autoridades locales de ese momento tanto municipales como regionales pues estaban saltando en una pata porque creían que todo estaba bien y un día puede haber sido un 23 de junio o julio, que recibo una llamada por ahí. Yo quiero decir desde este momento que no fue la iglesia ni mi persona la que tuvimos la iniciativa si no una persona que tiene un nombre concreto, Gisela Ortiz Rodolfo Ortiz y otros más en este momento, pues ya algunos incluso han dejado el colectivo pero que fueron los primeros en que llamaron por teléfono a eso de las 11 de la noche, y me dijeron hermano. ¿Qué hacemos? se nos viene una ola fatal de la pandemia de la Covid-19 y vamos a necesitar oxígeno qué hacemos y le digo bueno yo mi parte pues qué podemos hacer yo no me puedo meter mucho en este asunto, investigué pregunté incluso a obispos oiga que me están pidiendo esto y normalmente, pues, me aconsejaron que no me metiera porque eso era muy difícil y muy costoso pero al final nos decidimos no gracias a estas personas que tuvieron la iniciativa de formar el colectivo Cajamarca respira aquí yo solamente me sumé a ellos. Luego, claro, ellos hay que recordar la idiosincrasia de nuestro pueblo y la cultura que la presencia del sacerdote todavía es considerada en

nuestra cultura aquí en Cajamarca de manera especial, por lo tanto, pues me pidieron que presidiera este colectivo y empezamos a preguntarnos qué hacemos y esto era un poco en la primera pregunta: ¿Qué hacer? sabemos que se necesita oxígeno, pero ¿qué hacemos?

**I: Ahora ustedes, ¿Cómo es que inician la labor? entiendo que fue una llamada viendo la necesidad, pero ¿qué fue lo que inmediatamente o qué acciones inmediatas tuvieron o iniciaron?**

E: Lo primero que fue decir, bueno, conseguir balones decíamos muy bien, tenemos los balones y cómo los llenamos. Porque aquí comenzó el oxígeno a costar demasiado esto es una de las cosas que se tiene que puntualizar y ver yo no sé desde ya desde ahora o cómo hacerlo, de que no se puede tener como prioridad la ganancia económica antes que la vida de las de los seres humanos, esto era una preocupación porque decíamos si tenemos balones pero cómo los llenamos y aquí comenzó a costar en esos momentos hasta 1000 soles, la llenada de un tanque de 10 litros o como llama de metros cúbicos, 1000 soles o 800 Entonces bueno, esa es la otra preocupación y comenzamos ya a reunirnos con los diferentes actores de la sociedad que nos acompañaron colegios profesionales que también enviaron a sus representantes y comenzamos a preguntarnos si no sería posible que podamos comprar una planta. Y la ilusión lo digo siempre, no la ilusión del colectivo de las personas involucradas era comprarnos una planta de 60 m cúbicos, pero ¿cuál era la esperanza? era que íbamos a golpear las puertas de las grandes empresas que estaban aquí, que están aquí en Cajamarca, que son las mineras y nosotros y también golpear la puerta del gobierno regional, golpear la puerta de la municipalidad provincial y decíamos si nos dan yo qué sé 20 soles cada uno o nos dan 50 con eso tenemos de sobra nosotros realmente, como a veces se hace, comimos la torta antes de hacerlo y fuimos a las grandes y todas nos cerraron las puertas nos dijeron que el gobernador regional y el alcalde habían dicho que hay oxígeno de sobra en Cajamarca eso fue la expresión de todos cuando ya veíamos que la gente se estaba muriendo ya aquí en ese momento pues al final dijimos, miren, bueno, no podemos comprar una de 60, pero y hagamos algo y nos pusimos allí tenemos a la única empresa minera que todavía no estaba en explotación estaba recién en estudios no sé qué estaba haciendo, era XXXX, la única que nos regaló así desinteresada 30 balones de oxígeno no nos dio dinero nos dio balones, como ya teníamos algo, pero decíamos ahora. ¿Cómo lo llenamos? Y bueno, ahí es donde nace como nadie nos dio dinero y no nos querían ayudar las grandes empresas dijimos ahora. ¿Qué? pues entonces ahí ya el grupo CCH aparte PN. LS a GO las damas de más o menos del colectivo y el resto de los varones OS, el doctor MB, RO y SS, que entró en la política y dejó el colectivo, pero sigue siendo parte de ello, es decir, comenzamos a hacer una colecta publica que yo lo digo siempre, desde el fondo de la vida del Pueblo, una colecta que lo hizo la gente realmente de a pie algunas empresas medianas empresas nos ayudaron la primera colecta pero era ver a esa gente campesina vendedores, ambulantes, inmigrantes que estaban por aquí que se acercaban a poner un sol, medio sol, 10 centavos a las alcancías que se hizo no gracias a la a un grupo de artesanos que nos regalaron. Pero íbamos por las calles, por la calle pidiendo limosna teniendo tantísimos recursos aquí en Cajamarca. Fuimos al gobernador regional, fuimos al alcalde, le decíamos, oigan, nosotros tenemos esto ustedes den el resto, pero realmente fue una voltear la espalda, le dieron la espalda al pueblo yo lo digo con todo respeto y cariño, pero le dieron la espalda al pueblo y el pueblo sacó la cara por eso he dominado yo por lo menos lo denomino a oxígeno de Cajamarca respira es la solidaridad de los humildes hizo posible no que trajéramos una planta de 10 m cúbicos no podíamos más con 300 000 soles que podíamos comprar cuando pedimos, como se llama las proformas de la UNI nos pidieron por esa planta 1 280 000 soles solo por la planta tiempo, ni hablar, o sea, esto ya es de bromas esto es de grandes entonces gracias a Dios, conectamos con alguien en Trujillo Don Adolfo Díaz, que en paz descanse y goce y él nos ayudó con la primera.

Nos cobró 61 000 dólares para traer la planta e instalarla a esa planta es móvil tiene su carreta, que también costó como 40 000 soles la carreta está equipada para salir a donde sea pero el problema era ya tenemos la planta ahora donde lo ponemos entonces desde el principio, el colectivo en general su sugerimos que la planta se pusiera en el Simón Bolívar es el hospital antiguo que le llamamos nosotros aquí de Cajamarca, porque era el que no tenía oxígeno el hospital general nuevo tenía una planta que había regalado la minera Yanacocha sino me equivoco, entonces decíamos ayudemos al hospital con la planta, pero usted no puede creer lo que hicieron para no recibir la planta.

**I: ¿Cómo?,**

**E:** Lo que hicieron las autoridades de ese momento habido y por haber para no recibir la planta.

**I: ¿pero y a qué se debe su rechazo?**

**E:** No sabemos por qué, o sea, tenemos idea, pero eso a veces ya no se públicamente no se puede decir porque digo no en el sentido con ustedes, no hay problema nosotros creemos que era todo el monopolio que había del oxígeno aquí en Cajamarca.

**I: Cuántas o qué empresas son las que digamos, comercian o ¿producen?**

E: Hay una empresa tanto a nivel de la dirección regional de salud, la DIRESA y es el único que vende. Yo digo, me habrá sido por eso.

**I: Pero perdón, Disculpe padre. Usted dice que la DIRESA vende oxigeno a eso se refiere**

**E:** No la empresa que se llama Oxicax, ella es la que vende el oxígeno al Ministerio de Salud de Cajamarca a toda la región y él y el dueño había dicho que garantizaba el oxígeno a todo el norte de Cajamarca a todo el norte de aquí Trujillo, pero claro es que es una mentira porque no había a nosotros nos llamaba la gente cada 3 min, nos llamaban a los sacerdotes, al padre JC de la catedral y al que le habla nos llamaban por oxígeno la gente. Y no querían que encendamos la planta porque no llegaba a 99% de pureza porque solamente llegaba 93 es decir, nos ponía un montón de pretextos hasta que un momento el mismo gobernador regional del momento dijo que era una planta de segunda reciclada, tuvimos que llamarle la atención personalmente ¿cómo puede usted criticar la caridad del pueblo? a mí. ¿Puede usted decirme lo que quiera, pero no puede usted ofender lo que ustedes no hicieron y no han hecho? Lo ha hecho el pueblo y encima usted está diciendo que es una donde una planta de segunda que usted está mal, al final ya nos tuvimos que discutir y pelear un poco porque no querían recibirnos entonces al final dijeron que teníamos que donarles la planta a la RIS , a la Red Integrada de Salud encima teníamos que regalarles la planta para que puedan ellos hacer el convenio y ellos pagaban ya la luz, la energía, los operarios y ya no pero sino no querían y tuvimos que hacerlo porque la gente se moría y el oxígeno encima querían que trabaje solamente 8 horas cuando en plena segunda ola aquí en Cajamarca esa planta ha trabajado casi es la única, porque, gracias a Dios en el colectivo tenemos un gran equipo técnico que lo hacíamos funcionar que también no podemos asegurarlo no podemos dar pruebas de eso y en 3 oportunidades malograron la planta había indicios que nos robaban el oxígeno, l indicios de encontrar arena dentro de los no arena en las válvulas y esa planta está en una carreta en una cabina ósea no está en el suelo pero luego otra vez malograron el ventilador encontramos un desarmador adentro que habían metido el desarmador para que se atasque no hay otra cosa, en 2 oportunidades tuvimos que ir con la Fiscalía, con la Defensoría del Pueblo, por lo menos para dar miedo porque nos estaban robando y vendían el oxígeno que era gratis para la gente ha habido cosas muy serias que no se pueden denunciar con pruebas pero nosotros lo hemos visto, nosotros sufriendo, llevando el oxígeno para arriba y para abajo, cargando al

hombro los 2 sacerdotes con los jóvenes de la parroquia, ¿verdad? Y gente que se estaba aprovechando de eso uno dice donde estamos.

**I: Sí. Padre, entiendo, o por lo que me ha comentado puedo entender que ha habido múltiples dificultades tanto para la instalación como para la compra para conseguir el financiamiento o reunir el dinero necesario para la compra, pero antes de hacer la compra ustedes tuvieron algún tipo de apoyo personal de salud como para determinar cuál era la mejor opción del equipo cuál iba a ser el uso en función a la necesidad. ¿Cómo? así ustedes lograron determinar esta es la planta más allá del precio, sino de las características técnicas.**

**E:** Claro, por eso le digo la idea era comprar nosotros de 60 porque, según el doctor Benítez, Gilmar Senedo, los técnicos que no solamente eran técnicos de piezas y de cosas mecánicas sino también técnicos en ver un poco la realidad analizar lo que se venía entonces ellos dijeron aquí vamos a necesitar oxígeno a como dé lugar porque se viene la ola y nos vamos a necesitar pero claro no logramos tener el dinero, cuando este señor que nos llama de Trujillo y nos dice que nos daba por 200 000 soles una planta que nos habían pedido en otros sitios un 1 280 000 de dijimos, bueno dijimos esto es un milagro o esto es un atraco teníamos que ver viajamos en la Comisión con la ayuda de los del general de ese momento, Cacho que estaba acá que nos dieron un pase para poder irnos a Trujillo y poder movernos y el sentir cuando llegábamos por los diferentes puestos de aquí hasta Trujillo de Cajamarca a Trujillo que son más o menos entre 4 a 5 horas y en cada puesto veían las camionetas y decían son las camionetas del oxígeno dejemos pasar ni siquiera nos pedían documentos sólo decía son la camioneta del oxígeno de Cajamarca eso fue grandioso porque entendíamos que estábamos haciendo algo útil entonces los médicos y todo el equipo técnico de Cajamarca respira ellos vieron esa necesidad. Y bueno, dijimos, no podemos comprar más no tenemos más dinero. Así es que la que tenemos se compra. Eso fue todo.

**I: Ok, Entonces ustedes solamente tuvieron financiamiento para la compra de las plantas por lo que se recaudaba en la población en ningún momento llegaron a tener apoyo del municipio, del gobierno regional o de alguna institución pública.**

**E:** No, Bueno en la primera colecta que hicimos los trabajadores del gobierno regional hicieron como decimos, una chanchita y dieron

**I: Pero no a nombre de la institución, de manera ya como personas individuales o en un grupo de trabajadores.**

**E:** No, pero no hubo entonces la empresa de alimentos, SAC se llama, ella también colaboró, pero como empresa era una empresa pequeña o sea no es una empresa grande, entonces pero como le digo tanto en la primera como en la segunda colecta es un poco eso el estilo que hemos hecho y tenemos que recordarle a la población y yo siempre lo digo: a mí no me gusta repetirlo, pero yo creo que es importante que lo sepa la población y que se lo sepa porque mucha gente siempre nos ha preguntado no porque hay que recordar que yo he dicho siempre en este colectivo no hay un individualismo de una persona somos un colectivo y todos hemos trabajado y todos metemos la mano y todos hacemos lo que tenemos que hacer entonces yo siempre digo, es un colectivo, no es una persona, es un colectivo, entonces el colectivo que ha hecho una cosa que deberíamos hacer en nuestro país no malgastar las cosas ya nosotros, con 600 000 soles hemos puesto en marcha 2 plantas de oxígeno aquí en Cajamarca una de 10 m cúbicos, y la otra de 20 metros cúbicos una está en Simón Bolívar y la otra está en la Universidad Nacional de Cajamarca para la segunda planta tenemos que agradecer a la universidad al rector actual y su gente que no nos pusieron ningún pero para que funcionara en la en la universidad, todo fue distinto a la primera vez

distinto, totalmente. Pero ya para la segunda colecta, también tuvimos ayuda de una señora en Canadá, Cajamarquina, la señora que nos ayudó de verdad y también de ayuda de Alemania, de un cajamarquino que está en Alemania y también ayuda de unas religiosas que están en Madrid que conozco que también hicieron una colecta, ya tuvimos una pequeña ayuda desde el extranjero que fue bastante también de Alemania de una asociación, una que tienen aquí su sede en Cajamarca que nos ayudó tuvimos, pero como le digo hemos recogimos más o menos eso 600 000 soles entre las 2 colectas y ver en la primera colecta de manera especial a un notario que con su martillo, rompiendo con los años que tenía y con todo el peligro de contagiarnos y de todo lo demás pero ahí estaba el rompiendo las alcancías para contar de moneda para ver cuántos habíamos recogido era una ilusión era una para nosotros una fiesta estar juntos aquí en de la parroquia de la iglesia la recoleta ahí todos con las linternas de los celulares porque no había mucha luz pero ver ese entusiasmo por ayudar a los demás y es eso ha habido dinero de sobra a nivel de las instituciones estatales pero no hicieron nada esto se tiene que decir y bueno, yo no sé, tuvieron algo no lo sé que tuvieron los monopolios, pero hay que decirlo que no se puede despreciar la vida de los seres humanos por una ganancia, ¿verdad? Y esto lo hemos visto en todos los sitios por la pandemia que también se habla y se dice en la pandemia nos ha hecho sacar lo mejor y lo peor de la humanidad eso también hay que decir.

**I: Y la empresa y la empresa privada más adelante entiendo que la primera colecta que hicieron no hubo como un aporte muy importante, pero en la segunda colecta ocurrió lo mismo o ya hubo una mayor participación de la empresa privada.**

**E:** No, otra cosa que también tengo que decirlo incluso los congresistas de ese momento cajamarquinos sólo 2 dieron su colaboración otros no dieron, solo 2 congresistas dieron su colaboración y los demás nunca ni siquiera se agradecieron ni siquiera dijeron oigan gracias porque están salvando vidas, nada, pero nada de nada yo llamé la atención a algunos de ellos, les dije oigan congresistas, ¿Qué están haciendo por el pueblo? Encima me parece que en esos momentos había en el Congreso que querían dar una ley que el oxígeno solo se podía usar si era de 99 punto por ciento de pureza que significaba que las plantas que se estaban comprando no se podían usar. A mí me avisaron y escribimos una carta, me acuerdo el colectivo escribió una carta al ministro de Salud en ese momento, diciendo: oigan, no pueden hacer porque esa ley tenía nombre propio solo una empresa puede producir eso.

**I: Y cuál era el, digamos, de la información que ustedes tenían, como que sustentaba el hecho de que podía ser del 93% digamos, el porcentaje del oxígeno.**

**E:** Porque, en primer lugar, ya lo nosotros lo estábamos usando, porque la primera planta que trajimos a veces llegaba a veces llegaba al 96 aquí por la presión los técnicos saben mejor que yo ese asunto, pero nunca bajó nunca bajó de 93,60 y la gente lo usaba aquí como porque no había más.

**I: Y el personal de salud los especialistas que decían al respecto**

**E:** De verdad, ellos decían que eso se podía usar, que no había problemas, o sea que, si bajaba a 80 eso sí, porque a mí me explicaron que es como el aceite en el motor, ¿verdad? la pureza, la fineza y si es más grueso ya le podía afectar la garganta, la traquea esas cosas las fosas nasales, pero de esa de esa pureza no había ningún problema y se usaba diario entonces dijimos oiga se está usando y la gente se está salvando por este oxígeno de 93.60 y la planta que hemos traído la otra segunda planta llega a 96 o 98 y bueno no nos enseñó una cosa científica sino la practica y la realidad nos enseñó que ese oxígeno estaba salvando vidas incluso nosotros hemos pagado el permiso a la DIGEMID también se ha pagado no es que nosotros lo hayamos hecho empíricamente o por encima de las normas también hemos esa acción de

pagar a la DIGEMID para los permisos necesarios, porque había un permiso especial, por ejemplo, en la segunda planta pagamos 3 250 soles para el permiso

**I: Para el funcionamiento o para una supervisión, habilitación.**

**E:** Para que pueda funcionar tenemos el permiso de funcionamiento y en la ADUANA para la segunda planta 51 643,40 soles digo yo hacemos el bien y nos cobran.

**I: Y explicaron por qué tenía usted que pagar en la ADUANA o porque era parte de una donación o como lo canalizaron ustedes.**

**E:** Ósea la primera planta lo compramos a través de este proveedor entonces en eso no nos metimos nosotros entregaron la planta en Cajamarca la segunda planta de 20 metros cúbicos nos atrevimos a importarla nosotros mismos porque ya teníamos personería jurídica estábamos inscritos en la SUNART en la SUNAT

**I: Como colectivo**

**E:** Como colectivo Cajamarca Respira, entonces yo pregunté, pero ¿por qué tenemos que pagar el impuesto, porque tenemos que pagar el impuesto si somos una entidad sin fines de lucro en ayuda a la población, pero nos cobraron igual.

**I: Y le dieron una explicación a ustedes las razones.**

**E:** No. Simplemente nos cobraron por eso digo, o sea, yo creo que hay que hacer políticas que tengan yo sé que hay gente que ha comprado plantas para ganarse la vida y explotar a la gente y asaltar a las personas porque muy bien, pero para eso está el Estado que tiene dentro de su economía tiene que ver cómo puede hacer esas cosas, oiga usted para dónde lo lleva investigar y ver quiénes son ustedes y dónde está la planta o dónde está lo que trae, para qué va a ser desciende. Entonces yo creo que tiene que haber alguna política y también o sea si no pueden decir pues mira lo que hemos pagado.

**I: Ustedes tuvieron algún tipo de acompañamiento o supervisión en este proceso de importar la planta de instalarla de ver mantenimiento por parte del Ministerio de Salud o alguna otra institución pública.**

**E:** No, solamente nos vinieron a preguntar si estaba instalado y cuánto tiene de pureza y ya está gracias a Dios como le en el colectivo, un buen equipo de médicos y de técnicos es excelente ellos son los que han hecho y los que los sacerdotes lo único que hacíamos era poner la figura de los sacerdotes para que la gente nos ayude porque el resto lo ha hecho el colectivo.

**I: Y cuál diría usted que ha sido el principal reto para conseguir los fondos, digamos, las donaciones.**

**E:** Reto fue primero es confiarnos en los grandes digamos eso para mí y yo les dije a los a nuestros oiga no nos confiemos mucho de los grandes porque no lo sé yo siento que no y fue así no nos dieron nada, salvo la Southern, como le digo que nos dieron 30 y después nos dieron 25 balones más en la segunda ola y luego ya se sumó la Golfis que nos han regalado algunos balones, 10 balones.

**I: Ya eso fue ya para la segunda**

**E:** después de la segunda ola, pero lo principal lo ha hecho la gente

**I: A cree usted que se debe este desinterés o rechazo por apoyar de las empresas que son grandes en Cajamarca, no tienen una sola empresa minera tienen varias grandes la lácteas en fin diversas.**

**E:** Sí, si como les digo, Gloria creo que por insistencia nos dio algo el resto no era tanto porque no quería sino por lo que vendían las autoridades que no necesitábamos oxígeno en Cajamarca que teníamos oxígeno de sobra.

**I: Una desinformación.**

**E:** No creo que era desinformación ellos muy bien sabían lo que estaba pasando, sino que era el discurso porque había plantas en aquí, porque había plantas allá la minera Y regaló al hospital, H también tienen ahí y yo siempre les decía es oxígeno para el hospital, para los pacientes en el hospital pero en Cajamarca la gente no quería irse al hospital entonces había muchísima gente en su casa y ese era donde nosotros atacábamos y a pesar de todo la planta de simón bolívar llenabas cada 2 h y media llenaba 5 balones entonces decíamos 3 para el hospital 2 para el colectivo Cajamarca Respira, el hospital Simón Bolívar también ha usado el oxígeno de la planta y chiquitita de segunda como decía el gobernador también dio oxígeno porque hubo un momento como 15 o 20 días que todas las plantas se malograron hasta la del comercio, la de la empresa y la única que funcionaba era la del colectivo que nos quedábamos hasta las 3 o 4 de la mañana que nos quedábamos haciendo las piezas por eso digo, tenemos unos técnicos fenomenales que inventaron las piezas y a las 7 de la mañana estaba funcionando de nuevo lo hemos pasado para ayudar a la gente porque, claro, ese es el problema se malogra la planta, se malogra una máquina, se malogra un carro que hacen las autoridades los abandonan y ahí lo dejan como no es mío, hoy estamos en ese plan también cuidando que las plantas tengan su mantenimiento que estén funcionando .

**I: Las 2 que ustedes lograron comprar.**

E: De lo que sé es que las que están funcionando en este momento son las del colectivo Cajamarca, porque hasta la planta del seguro de EsSalud que son la misma capacidad que nosotros trajimos la segunda de 20 m cúbicos les ha costado cada planta 2 millones y medio a nosotros nos ha costado 300 000 instalada no sabemos por qué me imagino que será una planta de otro tipo, pues no sea prefiero pensar así, pero están malogradas

**I: Pero están malogradas por un uso excesivo por una falta de mantenimiento.**

**E:** Falta de mantenimiento porque la planta del colectivo Cajamarca respira le da todos los días sabemos que se va a malograr, porque no son máquinas que tienen que trabajar todos los días esas máquinas, pues nos explicaron los chinitos de la empresa que compramos nosotros en la segunda planta, nos explicaron con ellos conversábamos casi a diario yo veo una cosa y yo siempre lo decía en el colectivo prefiero ver malograda esa planta salvando una vida porque luego que tenemos que arreglarlo, fondos de donde sea para arreglarlo, porque mientras lo teníamos nosotros ahora lo tiene el de nuevo como se dice la nueva gestión en el Simón bolívar y la gestión en la Universidad que ellos ya se encargan del mantenimiento.

**I: Ustedes ya hicieron efectivo, digamos, la donación ya ha pasado a ser parte de los bienes de la dirección regional.**

**E:** Si pero la de universidad de Cajamarca no esa esta en sesión de uso esa sigue siendo nuestro.

**I: Y esa, por ejemplo, ¿quién se encarga del mantenimiento de esa planta?**

**E:** La universidad

**I: Y en este momento que ya no estamos en pandemia, bueno hay casos, pero no ya no reviste la gravedad que antes y cuál es el fin o cuál es el uso que se le está dando al oxígeno para qué están sirviendo.**

**E:** Está sirviendo para la gente que tiene problemas pulmonares, de corazón los pacientes que salen del hospital y requieren oxígeno en su casa más o menos un promedio de 10 balones diarios. Hay mucha gente que se ha quedado afectada.

**I: Entonces durante la pandemia, las personas que hacían tenían acceso o que recurrían a los balones de oxígeno me dice usted que no querían ir a los establecimientos de salud que preferían quedarse en casa. Sabe usted si estas personas tuvieron el acompañamiento de personal de salud de alguien que las monitoree, las acompañe en la administración del oxígeno en sus viviendas.**

E: Sí tuvimos lo que se llama el SAMU y luego también por la misma gente llamaba a los médicos, a las enfermeras particulares, y sabemos todo lo que ha habido ahí la ganancia que han hecho y eso da pena porque yo sé que había el peligro de contagiarse lo digo por mi persona también por el padre Juan Carlos y los 2 chicos que nos ayudaban que íbamos nosotros ya luego con el serenazgo que luego después ya nos ayudaba, pero al principio éramos solo nosotros porque nadie más podía hacerlo todo el mundo tenía miedo todo el mundo y nosotros hemos cargado los balones al hombro para llevarlos de un sitio a otro y ponerlos en la camioneta y no había más pero lo que nos da pena es que se aprovechó nosotros hemos ido a la Villa salud, donde habían 130 camas de graves ahí hemos entrado a visitarlo no solamente por el oxígeno, sino también como sacerdotes hemos entrado a los hospitales porque decíamos mira si no nos contagiamos cargando oxígeno ya no creo que nos contagiamos ya aquí visitando a los enfermos era también el soporte espiritual desde nuestra ministerio a los demás, como sacerdotes, no había nadie más y el colectivo tiene como particularidad que integramos gente de todo tipo incluso religiosos, somos distintos, política, profesional, religiosos gracias a Dios coincidimos en un solo objetivo para ayudar en el momento en que corresponda.

**I: Padre en este en esta labor que usted ha realizado tanto visitando los establecimientos de salud estando en contacto con los profesionales de la salud también ¿cuál diría usted que es la principal carencia en cuanto a los recursos para los programas de oxígeno? porque son como varias cosas no es contar con el balón, pero también es contar con la infraestructura para el uso, contar con el personal en fin, una serie de recursos que se requieren para que todo funcione ¿Cuál diría usted que es la principal en el caso de los establecimientos de salud de Cajamarca los que ha podido usted tener acceso?**

E: Yo la verdad que la primera cosa para la cuestión del oxígeno necesita una máquina que realmente los abastezca porque nos dijeron en la DIRESA nos dijeron que salía más barato comprar el oxígeno que producirlo eso nos dijeron cuando los técnicos habían sacado números yo qué sé lo que hacen la técnica, la tecnología habían sacado que para llenar un balón de oxígeno de 10 metros cúbicos era solamente entre operarios, luz y todo lo que se necesite se gastaba 28 soles pero para ellos decía que era más barato comprarlo cuando se llenaba un balón de oxígeno por 500 800 soles o 1 000 al entonces yo creo que lo importante es convencer a las autoridades que se necesita una planta de oxígeno criogénica o yo no sé verdad como es un poco internacional lo que están haciendo ustedes avisar a las a las empresas grandes que hacen este tipo de maquinarias que hagan estas máquinas pues que sean mucho más factibles ya le digo por aquí hemos podido lograr por el equipo técnico que tenemos de lo contrario esa planta hubiera estado parada hace tiempo pero creo que es importante producir en la verdad si es fácil se coge del aire,

entonces yo que sé, pero hacer máquinas que sean menos complicadas yo me imagino que no lo sé habrá que sugerir a las empresas o aquí en lo que tiene que hacer pero abastezcan de oxígeno yo veo aquí y entiendo que hay una empresa que trae el oxígeno pero uno que está todos los días trayendo un carro lleno de balones vacíos y cuando en un hospital debería estar conectada como el agua el oxígeno y en Essalud tiene 2 máquinas y no los hace funcionar que incluso han pedido al colectivo Cajamarca respira sin que lo sepamos nosotros que les llenemos bolones para el seguro ya eso está mal porque el seguro cobra a asegurados para darles brindarles un correcto servicio de salud.

**I: Una consulta ustedes en la universidad que está abierta ustedes cobran por el oxígeno.**

E: Hasta ahora es gratuito, yo sé que en algún momento tampoco yo como también lo he dicho no es que no se ha dicho, de era un momento de dificultad de y cuando hay un momento de emergencia para mí personalmente no hay ni pobres ni ricos necesitamos todos necesitamos porque no había donde comprarlo y no había plata o no había dónde comprar. Entonces le dio el oxígeno personalmente también que en una economía en un país dar gratis las cosas tampoco es bueno para mí ya en un momento que tendrá que pensar vamos a cobrar de una manera simbólica, pero algo se tiene que hacer porque y dar cosas o bonos porque es pobre estamos mal acostumbrando a la gente personalmente digo yo lo que se tiene que darle a la gente es trabajo digno y bien remunerado en entonces no habrá necesidades.

**I: Usted ha tenido la oportunidad de tener diálogos, conversación con el personal de la DIRESA para ver este tema ya sea ahora o digamos durante la pandemia.**

E: Sí hemos dialogado muchísimo diciendo les estamos dando una máquina para que tengan oxígeno en el hospital, lo que ustedes no pueden tenerlo y encima nos están poniendo trabas, en resumido las conversaciones que, y teníamos, ayúdennos.

**I: ¿Y cuál era la respuesta de ellos?**

E: Que tenemos que cumplir las normas que tenemos que las órdenes de Lima que tenemos que cumplir las órdenes les digo pero se está muriendo la gente ya no puede, en este momento nosotros nos levantamos, nos vamos y sacamos la planta de ahí del Simón Bolívar y la ponemos en la plaza de armas con un letrero que dice que ustedes no quieren que demos oxigeno eso les asustó y nos dieron el permiso.

**I: Ya para la primera planta cuando instalaron esa**

E: Para la segunda ya no hubo problema ya no dejaron instalarla y fueron a ver para ver cómo estaba si estaba bien instalado, si daba la pureza, pero bueno después nada mas no nos ayudado.

**I: Digamos, fue más la presión que ustedes sintieron de la decisión de ellos.**

E: Ellos por su parte no querían eso estaba claro por eso decimos algo tiene que haber yo personalmente todos tuvimos de acuerdo cuando dijimos, oigan la razón que ustedes tienen para no dejarnos que la planta funcione por supuesto no dijeron nada no hay ser muy inteligente para imaginarse uno, si un balón costaba aquí 800 a 1 000 soles llenarlo o recargarlo era un negocio redondo en ese momento.

**I: Bien y en Cajamarca en los establecimientos de salud quienes digamos son los responsables de regular este tema del oxígeno medicinal alguna dirección en específico.**

E: Sí, hay una, hay una como la DIGEMID que ser como la DIGEMID chiquita será, la verdad no me acuerdo ahora el nombre nos llamaron varias veces a pedirnos documentos, papeles si teníamos el permiso de la DIGEMID que si la planta tenía las dimensiones que decía en el papel pues nosotros le dábamos lo que nos pedían incluso recientemente con la nueva gestión que hay me están pidiendo de nuevo los mismos

papeles que dimos bueno si es necesario además ya le hemos regalado a ustedes pero nos siguen pidiendo.

**I: Además de esa de esa solicitud de documentación luego hay otro tipo de supervisión, control.**

E: Yo, que sepa el de Simón Bolívar que está dentro del hospital está la DIRESA queda ahí mismo el RIS también me imagino que si piden, pero ya nosotros hemos salido; de la universidad según los operarios se que habrán ido en todo este tiempo unas dos veces, pero simplemente para ver si no vota humo yo que sé nada, más o sea no hay una ayuda.

**I: Por la experiencia que usted ha tenido, por lo que ha podido ver también con lo que ha ocurrido en el país en otras partes en su opinión, cuáles son las áreas claves en las que debería centrarse política nueva sobre el oxígeno medicinal que debería tener en cuenta.**

E: Bueno, ya lo mencioné antes no sé cuál será la cobertura, la participación que se pueda tener ver las máquinas que sean lo que sé más prácticas digo yo primero y luego yo creo que habría que insistir a los gobiernos locales que hagan algo sobre eso donde sean ellos los ven aquí mismo hay una planta que compró en la gestión anterior del municipio y está sin función, un elefante blanco para que tienes ahí y luego también creo que es importante ya la parte de la población que no se le acostumbre siempre a dar gratis creo yo creo que el refrán chino de no dar un pescado sino darle una caña y enséñele a pescar. Y lo que dice San Pablo el que no trabaja que no coma en serio eso habría que ponernos en educar a nuestra población también porque hoy en día ese es el problema yo lo vivo aquí, a la gente solo está acostumbrado a pedir pero no es capaz de decir oiga yo puedo hacer algo para ganarme el diario no solo pedir ni pedir yo creo que tiene que ver una política ahí de devolverle la dignidad humana a la persona no es un mendigo, somos seres humanos que podemos trabajar que conseguir lo que queramos y que nosotros, como colectivo lo hemos logrado en el momento era por la necesidad, pues pero creo que hay que ir pensándolo en serio que no es siempre esto.

**I: En general, para usted. ¿Cuáles fueron los principales retos que se tuvieron respecto al oxígeno medicinal durante la pandemia? me ha hablado un poco del financiamiento un poco del poder importar ya de la instalación, pero si usted tuviera que todo tener una perspectiva así en conjunto, ¿cuál diría que fueron los principales retos que tuvieron que afrontar?**

E: El principal: convencer a las autoridades que había una necesidad y eso creo en cualquier situación, están preocupadas más en cuántos votos van a tener que realmente ayudar a la población yo creo que para nosotros aquí en el colectivo, en el reto más grande que tuvimos fueron las autoridades.

I: Las autoridades de ese entonces ahora que han cambiado como les ven a los actuales autoridades como preparadas, están dispuestas a escuchar, asumir responsabilidad.

E: Yo verdad que los veo casi lo mismo a las autoridades, yo personalmente no conozco a los recientes pero al alcalde si pero los veo en la misma y además no solamente a ellos, sino ya uno ven en las noticias y ve los alcaldes de aquí, los gobernadores, los congresistas que están preocupados en que poner el día del chupetín y otras cosas y tu dices que realmente pienso yo creo que la primera reto que tenemos es fomentar profesionales en la administración pública pero que sean gestores, administradores que no solamente bueno, porque hoy ganaron pues ya ahora ya estamos aprovecharnos aquí de todo lo que nos tenemos que aprovechar porque de aquí a 4 años o 5 nos vamos entonces yo creo que habría que implementar una de administración pública, porque eso es lo estamos viendo, y no solo en la administración pública le decía a la doctora Patricia hasta en la Iglesia sale un cura de una parroquia, viene

otro y acaba con todo lo que ha hecho el anterior, entonces lo digo con toda sinceridad mientras no tengamos una buena capacitación de administración pública esto creo que no va para arriba.

**I: Y cómo cree que está la situación hoy en día en relación al tema del oxígeno medicinal, ¿usted cree que se ha aprendido algo? Ha cambiado algo ¿Cómo estamos?**

E: Yo creo que la gente ha aprendido que el oxígeno es importante en primer lugar, pero también ha aprendido que hay que aprovecharse del oxígeno es la lo que salva vidas, pero esto es la verdad, es que nadie dice que no pones una empresa para perder, pero no te aproveches de las desgracias de los demás creo que el oxígeno por lo menos a podemos pensar sacar algo nos ha enseñado que no podemos aprovecharnos de la desgracia hoy en día.

**I: Eso a nivel de la de la población y a nivel de las autoridades de los gestores, usted nota que ha habido algún aprendizaje, algún cambio.**

E: Aquí en Cajamarca, le digo con sinceridad más ha habido una terquedad del anterior gobernador regional, porque incluso ha un libro no se si se equivocó enviándomelo un libro donde él sale como que es el salvador de Cajamarca este señor no ha aprendido nada, político, simplemente político buscar ganancias de lo que el no hizo hasta me molesto un poco los que hemos hecho el bien al pueblo ayudándoles con todo lo que hemos podido en el tiempo de la pandemia no hicieron nada con todo el dinero que tuvieron hasta ahora se encuentran cajas abandonadas llenas de mascarillas ahí que no lo repartieron no han aprendido nada para mí.

**I: Se que esto puede ser como medio abstracto, pero a usted cree que si las autoridades hubieran tenido mayor interés o se han comprometido realmente con el trabajo que hacen el tema durante la pandemia la situación hubiera sido distinta, las condiciones lo que hubiera pasado con la pandemia.**

E: Hipotéticamente sí porque tenían todo el dinero que podían, pero yo no sé qué han hecho con ese dinero porque no implementaron camas UCI, no implementaron respiradores, no compraron aquí por lo menos aquí que yo sepa porque esta es la realidad en la que estoy y no he escuchado en otros sitios tampoco pero no compraron una sola planta una como gobierno local y yo decía oiga usted puede comprarle un 5 pero no, no era la política, seguro.

**I: Sabe usted si se orientaron los recursos a otro aspecto: recursos económicos me refiero, a otro aspecto vinculado a la salud en la región.**

E. Pues mire decían que sí, pero nosotros no lo hemos visto, no se contrató más personal, la gente tenía miedo, pero yo verdad, no y todos sabemos lo que ha pasado vendían las camas a precios por debajo y el que tenía como decimos el refrán, el que tenía padrino se bautizaba el que no, no, no lo yo sé que tampoco era el momento, porque nadie ha podido pensar esta esta desgracia que hemos tenido esta emergencia pero la verdad que no le he visto que hayan hecho algo serio por paliar un poco la situación de la población, la población se moría aquí y decían que en el a nivel de la DIRESA decían que hemos contado hasta 70 u 80 eran 40 que se morían en los hospitales, pero la gente en sus casas, eso no estaba registrado entonces, yo verdad que sí hay que incentivar una formación de la administración pública y habría que hablarle a la gente al corazón como decía Newman un cardenal, hay que hablarle al corazón porque mientras no cambiemos el corazón de las personas ya podemos hacer las políticas que podamos, pero no va a cambiar nada por qué no se trata estructura política e ideológica pero se trata de las personas las que mueven y hacen funcionar cualquier ideología, pensamiento, cualquier ideología, son las personas, no hay otra cosa.

**I: Ya como para ir finalizando en su opinión, si en este momento se diera una nueva pandemia o tuviéramos un desastre natural importante. ¿Cuál sería la situación del oxígeno en su región?**

E: Lo mismo porque no hay, porque ya le digo las plantas que están funcionando en este momento son las de Cajamarca respira.

**I: Son las 2 únicas, ni en otro en otra provincia, ni nada.**

E: No, sé que están malogradas, me imagino que logra que una de esas o estaremos nuevamente corriendo bueno haciendo lo posible nuestras autoridades al alcalde actual de Cajamarca ya le digo como 4 veces que lo encuentro le he dicho lo mismo, señor alcalde haga funcionar esa planta que tienen ustedes ahí, ya ya pero no funciona entonces no se ha aprendido como se dice somos un país que actúa a lo que viene no somos previsores, yo también le pensaba yo digo, una planta a nosotros nos ha costado así pero que a unos les ha costado 1 000 200 y al otro, 2 000 000 y medio que yo sé que son plantas muy caras para comprar una planta para tenerlo 15 días parada, 15 días funcionando que está bien según la economía o según los parámetros monetarios, entre lo que es ganancia y beneficio que si pensamos así, pues, no vamos a hacer nada, como le dije una vez a un médico que dijo que nosotros los médicos, debemos ganar mucho más porque estudiamos tantos años y yo simplemente le contesté y le dije entonces yo tengo que ganar el doble que usted porque usted ha estudiado 7 años yo he estudiado 14, gana gloria aparte, no es eso es el servicio que prestamos que como empleados como profesionales que tienen que como dignidad, respetar su profesión, su capacidad pero no es porque yo estudié más tengo que ganar más no es eso pero como le digo no hemos aprendido. Una planta ahí malograda no los hace funcionar, otra que está ahí parada que no lo hacen funcionar, las de H también igual, la de Cajabamba igual, la de chota igual

**I: No funciona solo por el tema del mantenimiento o porque no hay la necesidad, porque a veces, o sea, usamos algo en tanto lo necesitamos, pero no se instala realidad de la zona.**

E: Pero aquí por ejemplo las 2 plantas están funcionando, es decir, están dando oxígeno no es que por funcionar, no está ahí hay harta demanda de oxígeno por problemas pulmonares, cardiacos no es que no haya necesidad hay demanda, ahora ya les he dicho yo en el colectivo ahora si hay que dar a la gente que no tiene recursos en este momento, porque ya no es un momento de emergencia ya es estado normal de enfermedades y complicaciones, pero la demás están paradas y hay mucha gente que lo necesita 2 o 3 balones de oxígeno semanales aunque ya hoy les cobren 150 o 200 para llenar pero son 3 no hay una economía fácilmente que aguante eso.

**I: Padre me queda una inquietud de la información que usted tiene, tener plantas de oxígeno podrían ser como una única opción para los establecimientos de salud, o se podría tener algún otro sistema u otra forma de proveer oxígeno a la población.**

E: Yo como le digo la planta en el sistema de la misma máquina es complicado para realidades de los caseríos o las postas médicas o los centros de salud de distritos es complicado, porque el sistema de la máquina es duro, es difícil entonces no lo veo yo tampoco lo que puede ser una un sistema porque también lo pensamos como colectivo, hacer la el sistema de microginesis que se congela el oxígeno se lo trae y se descongela y produce oxigeno creo que es un sistema que podría ser un menos complicado de enfriamiento por la piezas, y yo creo que es importante que se tenga un sistema de proveer el oxígeno a los centros de salud grandes grande en principio los pequeños ya sabemos que eso siempre derivan que tengan su balón de oxígenos allí llenos para las emergencias que necesitan per lo otro yo creo que habría que buscar un sistema que sea mucho más factible y práctico las plantas yo lo veo un poco complicado.

Si, por ejemplo, la de la universidad en este momento se necesita un promedio de 17 000 sol para ponerla de nuevo a todo poder.

**I: Ya, pero eso para qué, para hacerle mantenimiento**

E: Mantenimiento y cambios de algunos motores porque son caros, pues hasta ahora son caros.

**I: Y la energía eléctrica y todo eso también**

E: Claro eso es menos porque eso lo hacen a nivel de trifásica y no quieren poner trifásica

**I: Son varios requerimientos, no varias necesidades**

E: Para el tipo de planta de maquinaria que se tienen en este momento, pero habría que buscar o ya para esos, tendrán que sentarse los técnicos, los mecánicos y pensar en una cosa más práctica.

**I: Y ustedes entiendo las dificultades que pasaron con las autoridades del Gobierno local o regional en algún momento tuvieron acercamiento con las autoridades de nivel nacional como para ver el tema de las plantas o las necesidades de oxígeno que había en la región la dificultad que ustedes estaban teniendo.**

E: Sí. Como le dije en este momento, le escribimos a no sé si lo habrá recibido porque en ese momento estaba MA todavía como congresista nosotros a través de él hicimos llegar alguna carta, pero nunca tuvimos respuesta yo me imagino que no llegó, pero sí, pero sí intentamos hablar, intentábamos decirlo a través de los medios también lo hicimos, pero no.

**I: Nunca tuvieron comunicación directa con autoridades del Ministerio.**

E: No, no directa, en algún momento pensamos incluso viajar como colectivo, pero tampoco se dio porque bueno, ya las circunstancias cambiaron todo lo demás yo creo que es importante que se le diga a las autoridades que no se puede pensar en un tipo como la pandemia o una emergencia, estar pensando en ganancias personales o lo que sea cuando hay una necesidad de una población que está sufriendo por lo menos yo en las entrevistas que he tenido como presidente del colectivo, pues yo lo he intentado decir, o sea, con todo respeto pero que nuestras autoridades tienen que pensar en lo que para lo que han sido elegidos para ayudar a la población.

**I: Padre, ya para cerrar la entrevista si usted pudiera señalar 2 lecciones claves respecto del tema del oxígeno medicinal, lo ha ido como ya comentando en varias preguntas, pero si usted pudiera identificar de todo lo que hemos venido hablando 2 lecciones clave que nos podría decir.**

E: La primera, que siempre tiene la iniciativa el pueblo, las autoridades no, en el pueblo como le digo por la experiencia la iniciativa fue iniciativa del pueblo. Y la otra, pues la solidaridad del pueblo que soluciona los problemas que las autoridades no lo hacen; y ya si quieren un tercera la negligencia de nuestras autoridades.

**I: ¿Negligencia en qué sentido?**

E: Bueno la inteligencia en saber ayudar al pueblo, no saber usar su cargo para el bien de la población, esconderse o decir simplemente no me toca, una apatía a veces.

**I: Bueno, padre, muchísimas gracias por compartir este tiempo con nosotros por apoyar y participar en el estudio ya no tengo más preguntas, pero no sé si usted quiere señalar algo más, algo que considere importante, es decir, que tal vez yo no lo he preguntado.**

E: Finalmente decir, que solo trabajando en equipo saldremos adelante no hay otra cuando vamos individuales ni que fuéramos superhéroes, o sea, no se puede, sólo en equipo podemos hacerlo y eso lo hemos demostrado en Cajamarca. Respira muchas veces, no con muchos miedos también discusiones a veces no nos poníamos de acuerdo, pero si hay con un objetivo y el objetivo se plantean todos juntos se sale adelante, pero si cada 1 jala agua para su molino eso no funciona.

**I: Ok, Muchas gracias. Padre.**

E: A ustedes muy bien que Dios les bendiga y cualquier cosa pues aquí estamos.

**I: Perfecto. Gracias. Voy a detener la grabación**

# 010 Entrevista

Meeting started: 21/6/2023, 5 PM

Participantes: E, I

I: Investigador

E: Entrevistado

**I: Hoy es 11 de julio del 2023 vamos a iniciar una entrevista realizando con el estudio que viene realizando la universidad peruana cayetano heredia respecto del oxígeno medicinal. En este momento voy a conversar con una responsable de un área de una institución pública. Y para confirmar que está de acuerdo con la grabación ¿podría usted señalar que está o no de acuerdo con esto?**

**E:** Estoy de acuerdo con la grabación, he leído el consentimiento informado. Conforme

**I: Muy bien, muchas gracias. Entonces para ir comenzando o también para tener un contexto o tener la idea de con quién estamos conversando y tenerlo registrado aquí, podría por favor comentarnos su función en relación a los servicios del oxígeno medicinal , cuánto tiempo lleva trabajando en este puesto¿Que ha cambiado en su labor en los últimos años? no sé si siempre ha sido el mismo puesto que ocupaba, en fin un poquito para conocerla. Está en mute, debe activar su…**

**E:** Sí, bueno mi nombre es___ , soy químico farmacéutico, actualmente directora XXXX, de XXX de cajamarca XXXX. Tengo la responsabilidad desde hace ya más de 4 años, información en el, mi experiencia en el tema sanitario en el sector público, en este octubre del 2023 cumpliría ya 13 años. Prácticamente me he formado en los procesos de gestión de suministro.

**I: OK. Entonces, ya por lo que me cuenta es bastante tiempo. Siempre en la misma institución o en otra región, o siempre en cajamarca**

**E:** Siempre en cajamarca y en el periodo 2016 -2017 laboré en el hospital regional docente pero siempre en cajamarca.

**I:** OK. Entonces cuénteme un poquito acerca de cómo ha sido el tema del oxígeno medicinal respecto de lo que ha ocurrido en cajamarca. Como ustedes, entiendo que pasaron, todo el país en realidad ha tenido dificultades serias con el tema del oxígeno medicinal durante la pandemia. Pero cada región tiene sus peculiaridades ¿no? Entonces me gustaría conocer cómo desde su puesto, desde su labor usted vio este tema

**E:** Sí, bien básicamente pues es la pandemia por COVID-19 el oxígeno, tanto el oxígeno medicinal como los equipos de protección personal¿no? Se convirtieron en insumos críticos¿no? Para obviamente garantizar el manejo y tratamiento a nuestros pacientes por COVID-19. Nosotros en cajamarca y mi persona, fue quién de alguna manera lideró el tema de poder diariamente realizar monitoreo, el análisis, el seguimiento y por tanto asegurar la disponibilidad de oxígeno medicinal en nuestros 8 hospitales de la región cajamarca. Nosotros en cajamarca pues tenemos 9 hospitales, tenemos 13 provincias; en 9, en 7 de ellas tenemos hospitales y desde DIRESA cajamarca obviamente nuestro rol y nuestra función prioritaria era ello. Recuerdo mucho que el primer caso, el primer paciente fallecido en cajamarca fue en abril 2020 y bueno nosotros desde la primera semana de abril del 2020, implementamos el registro diario tanto de stock como de consumo de nuestros, vuelvo

a repetir, de nuestros 8 hospitales porque obviamente en los hospitales se concentraba el manejo de los pacientes de COVID-19. Para nosotros fue elemental, obviamente, porque si no conocíamos el stock, el movimiento estaríamos perdidos. Si bien es cierto, a nivel país se tuvo situaciones muy difíciles y críticas de garantizar la disponibilidad y abastecimiento de oxígeno medicinal; y gracias a Dios en Cajamarca pudimos manejar ello¿no? En nuestros hospitales y ya en tercera ola digamos en nuestros establecimientos del primer nivel se garantizó el oxígeno medicinal, y vuelvo a repetir uno de los factores principales fue el monitoreo, monitoreo de la disponibilidad, cuál es su stock, de cuánto consumes, cuántos balones de oxígeno teníamos en cada hospital, cuánto es tu consumo diario¿no?, cuánto es incluso coordinamos con el proveedor,¿cuánto es tu stock que tú tienes en planta? Porque obviamente en cajamarca solamente teníamos un proveedor¿no? Y no es un proveedor obviamente fabricante, este proveedor este… se suministraba desde Ancash, Lima parece en algún momento. Entonces…

**I: Perdón¿ cuál es el nombre del proveedor antes que se me pase?**

**E:** Nosotros… garantizar la disponibilidad de oxígeno… fue el rol tanto del ministerio de salud, hay que reconocerlo CENARES porque Cenares financiaba, financió el oxígeno. En la primera ola sí lo financiamos como DIRESA, recuerdo mucho que todo nuestro presupuesto fue destinado para la adquisición de oxígeno, ya el CENARES es a través de los decretos de urgencia establecía que el ministerio de salud debería financiar el oxígeno; ya tuvimos un respiro financieramente¿no? Financieramente porque fue interminable obviamente el día a día, vuelvo a repetir en el tema del monitoreo de garantizar esta disponibilidad de oxígeno en nuestros hospitales. Y nosotros tenemos hospitales en las provincias de Cajabamba, Celendín, Cutervo, Bambamarca ,Chota , Jaén¿no? la distancia por ejemplo , y cajamarca mismo obviamente, con nuestros 2 hospitales regional de cajamarca y Simón bolívar que fue hospital covid en la primera ola. De cajamarca a cajabamba, 2horas y media, 3 horas a 3 horas y media obviamente por la carga significa, que significaba transportar los balones de oxígeno. El suministro era a veces diario¿no? Porque… los pacientes, obviamente teníamos más casos de pacientes, el consumo de oxígeno era mayor, en algún momento cajabamba, en el hospital de cajabamba, los médicos decidieron utilizar las cánulas de alto flujo y no midieron obviamente que el consumo que las cánulas de alto flujo obviamente requerían significaba pues que en 6 horas la disponibilidad que estaríamos garantizando para 2 días obviamente, se consumiera no, simplemente uno o 2 pacientes. En la distancia de cajamarca-cutervo 6 horas¿no? El proveedor ah ya, usted me preguntaba quién es el proveedor; el proveedor en cajamarca fue Oxicax; operador logístico oxicas. Es la única empresa obviamente que se tiene en cajamarca y esta empresa pues era…

**I:** Perdón, es oxicas con C

**E:** Oxicax, con C . OXICAX, es operador logístico no recuerdo bien el nombre final pero es Oxicax. Ellos se encargaban de garantizar el suministro a los hospitales de la región¿no? Un solo proveedor. Entonces obviamente también se coordinaba en forma permanente con ellos a fin de ver el número de unidades de transporte¿no? Que tenían, el número de balones de oxígeno que esta empresa en cesión de uso debía asegurar en nuestros hospitales¿no?, cuánto era el stock que tenían en planta ¿no? Para vuelvo a repetir evitar estas situaciones de quiebre de stock y obviamente preservar la vida y la salud de nuestros pacientes.

**I: Usted me dice de que en la primera ola, ustedes destinaron todo el presupuesto que tenían para el oxígeno.¿Este presupuesto es un presupuesto específico para el tema del oxígeno o de qué presupuesto estamos hablando?**

**E:** fue un presupuesto de todas las fuentes, no solamente para oxígeno. Sabemos que en covid, todo el presupuesto se destinó a garantizar la disponibilidad de equipos de protección personal para nuestros, para los profesionales de salud, oxígeno y obviamente el tratamiento para COVID-19, sobre todo los tratamientos de alto costo en UCI¿no?.

I: Eso quiere decir que todo el presupuesto…

**E: fue de todas las fuentes, donaciones y transferencias, recursos ordinarios,RDR.**

**I**: Ya¿eso es sólo para la primera ola?

**E: sí porque ya en, recuerdo que a partir de septiembre del 2020, ya el ministerio de salud establece aprueba los diferentes decretos de urgencia. Y los cuales se transfiere por supuesto al CENARES , destinados para la adquisición de oxígeno.**

**I**: y cómo les fue a ustedes, el presupuesto que tenía¿cubrió las necesidades? Cómo hicieron porque fue diverso en realidad, las olas eran terribles, una más grave que la otra, no sé cómo en fin ; no sé cómo lograron ustedes asumir eso

**E:** Sí, como le menciono gracias a Dios en cajamarca nosotros sí garantizamos la disponibilidad de oxígeno en los distintos hospitales. Probablemente existían pacientes con uso de oxígeno en domicilio… que el financiamiento de alguna manera era un gasto de bolsillo¿no? Pero no era porque nos faltaba oxígeno en los hospitales sino porque ya no había cama que eso también fue un tema crítico a nivel país. Pero nosotros establecimos la ruta del oxígeno porque obviamente como equipo de gestión identificamos ello¿no? O sea largas colas, los pacientes comprando oxígeno ¿no? Eso pasaba de alguna manera de forma irracional, porque tampoco se trataba de comprar oxígeno, el oxígeno es un medicamento. Como equipo de gestión se estableció la ruta del oxígeno con el equipo samu de DIRESA cajamarca, se identificaban a los paciente que necesitaba oxígeno en casa, se realizaba el monitoreo, las visitas… logramos de alguna manera también poder… más que todo realizar el seguimiento oportuno del uso de oxígeno nuestros pacientes. Pero vuelvo a repetir, y nuestros hospitales no fue fácil tampoco no fue todo tranquilidad porque vuelvo a repetir no desde el inicio de la pandemia hasta el 2022 finalmente hemos tenido que realizar, y actualmente hasta el día de hoy los hospitales envían el stock y consumo de oxígeno¿No? Ya en el 2021 el minsa bueno a través del ministerio de salud establece el reporte en el aplicativo Renoxi¿no? De stock de oxígeno que obviamente sirve, nos ayuda a realizar el monitoreo. Como regiones podemos podemos establecer alertas, pero como región cajamarca nosotros eso bueno una plantilla en Excel, en un drive lo establecimos desde abril del 2020 los hospitales y hasta el día de hoy lo reportan; y eso fue clave porque si nosotros no tenemos información de costos, de consumo, obviamente estamos a la deriva¿no? La información era muy importante, entonces en el tema financiero vuelvo a repetir, en la primera ola que nosotros sí tuvimos que usar garantizar el financiamiento de oxígeno sobre César todo para nuestro hospital Simón bolívar que era el hospital covis, donde se manejaban todos los casos. El, el financiamiento fue de todas las fuentes, felizmente antes de pandemia teníamos, digamos una, un stock de seguridad que servía para la la demanda normal de nuestras de las y IPRES del ámbito de nuestra ejecutora. Pero financieramente no tuvimos problemas digamos para poder este…

**I: Abastecer**

**E:** No, no se tuvo problemas gracias a Dios. Vuelvo a repetir en **(Ininteligible, MINUTO 15:38)**que el Cenares inicia el financiamiento. Con CENARES también tuvimos que…

**I: OK, sí a eso iba. Porque ustedes han tenido como una primera, asumieron la responsabilidad en la primera parte y me dice que no no tuvieron dificultad en cuanto al presupuesto. Pero qué tal con CENARES, qué tal a través del minsa ya con la intervención de una gestión de algún nivel diferente**

**E:** Este… fue buena¿no? Fue buena en las coordinaciones con los responsables de los suministros de oxígeno también eran de forma permanente, veíamos que el proveedor estaba incumpliendo según la cantidad de balones que nosotros requeríamos, en algún momento ellos enviaron, realizaron también recuerdo mucho convenios, coordinaciones con la empresa privada o con las mineras¿no? Estas mineras adquirieron oxígeno líquido¿no? Oxígeno líquido que acá el proveedor obviamente lo transformaba a oxígeno gaseoso, eso también nos ayudó en la segunda ola. Lo que también nos ayudó en la segunda ola fue las plantas generadoras de oxígeno¿no? Porque el tiempo, vuelvo a repetir el tiempo de suministros¿no? Por qué nosotros podríamos tener ,CENARES bueno realizó la adquisición de más de 30000 o 40000 m³¿no? ¿Pero dónde se encontraba stock?, ese stock se

encontraba o bien en el proveedor¿no? Pero no en nuestros hospitales porque obviamente no teníamos la capacidad suficiente de balones de oxígeno¿no? Llegamos a consumir, recuerdo mucho el pico, en segunda ola en un día en el hospital Simón bolívar 1000 m³ de oxígeno, es decir 100 balones de oxígeno; eso significaba, yo les decía, mi consumo es 100 balones por lo tanto yo tendría que tener 100 balones de oxígeno como reposición. Yo no puedo esperar que se me acabe y recién llamar al proveedor. Uno de los factores críticos en primera ola fue los balones de oxígeno¿no? Y es por eso que también el minsa luego adquiere balones de oxígeno porque eso era uno de los de los **(Ininteligible, MINUTO 17:43)**críticos. Y es por eso que obviamente las plantas generadoras de oxígeno sí ayudaron¿no? Eso fue en 2021, en segunda ola.

**I: OK, ok**

**E:** Donde fue el otro, este… la otra fuente de suministro de oxígeno…las plantas generadoras de oxígeno

**I: Sí. A ver usted me dice de que inicialmente, cuando ustedes están viendo directamente el tema con su presupuesto , el vínculo o el proveedor era esta empresa que me ha mencionado que es OXICAX.**

**E:** Operador logístico

**I: Sí, operador logístico. Una vez que pasa CENARES¿ sigue siendo este mismo proveedor o ya tienen otro?**

**E:** Sigue siendo el mismo proveedor, y por qué vuelvo a repetir porque en cajamarca es la única empresa que tiene todo este soporte logístico, entonces para nosotros era crítico tener una sola empresa proveedora y que garantice la disponibilidad para los 8 hospitales de la región. En jaén había otra empresa que fui…Cenares también contrató a otra empresa, fue… LINDER. Linder, pero el Linder incumplió; por ejemplo en Linder ganó en chota, recuerdo chota, cutervo y jaén. Sin embargo el tiempo de oportunidad de distribución no ,no ayudó nada, no ayudó en nada.

**I: ¿Cómo así? Cuántos si se… o sea no llegaron a concretar ningún pedido, a proveer ningún pedido o cuál fue…**

**E:** Se solicitaba pero la empresa incumplía. Porque obviamente no tiene en cajamarca, digamos una sede que les permita realizar las, las reposiciones solicitadas¿no? Lo que hace Linde sí, es el tema de oxígeno líquido que si hubo realizada la reposición al hospital regional de cajamarca y también a jaen pero que en jaén también incumplió y nos puso en crisis¿no? El tema de oxígeno líquido.

**I: Estoy, tengo la percepción de que es como, se cumple más en la capital de la región que en las provincias¿es una cuestión logística o cuestión de stock suficiente?**

**E:** Le comento, en cajamarca, en los hospitales tenemos el suministro de oxígeno gaseoso y oxígeno líquido¿no? Oxígeno líquido con tanque criogénico, y quien tiene tanque criogénico es el hospital regional de cajamarca, en cajamarca, y el hospital general de jaén en jaén¿no? Probablemente para linder sea más fácil el transporte de Lima cajamarca versus que de Lima a jaén, entonces esas situaciones es en pandemia si nos pusieron en una situación en algún momento crítico en jaén.

**I: Ok,ok. Y los costos fueron como los mismos¿sabe usted?**

**E:** No,no ,no. Recuerdo que en el 2019 , espero no equivocarme, el costo de metro cúbico de oxígeno fue, era 9 soles, 8 soles ¿no?en pandemia este se l elevó a 20 soles, 21 soles, 22 soles.

**I: Ya, pero¿ era proveído por la misma empresa?**

**E:** Sí, por la misma empresa

**I: Y les dieron una explicación por qué el costo , se elevó el costo. El insumo sigue siendo el mismo**

**E:** Ellos indicaban¿no? Que por pandemia también hay quienes ellos compraban también habían incrementado los precios. El tema de la escasez…

**I: 20 soles el metro, o sea más del 100%**

**E:** Por supuesto

**I: 120% más por lo menos. ¿Y usted sabe cuánto está en este momento? Si digamos estamos ya…**

**E:** 16 soles, hemos realizado una compra…

**I:¿cuánto?**

**E:** 16 soles.

**I: Ya, o sea que tampoco ha bajado mucho**

**E:** Lo que pasa es que también a nivel mundial¿no? El costo de los medicamentos se ha elevado, hay una situación crítica de desabastecimiento de varios productos por múltiples causas, en este caso del oxígeno no sabría precisarle pero sí el costo es 16 soles aproximadamente, actualmente.

**I: De acuerdo. Usted también ha estado comentando que a partir del año pasado del 2021, 2022 me dice que ya han comenzado a registrar los datos en la base de datos del minsa. Pero antes de eso ¿cómo ustedes controlaban o tenían información acerca de por ejemplo las necesidades que tenía respecto del oxígeno y la disponibilidad que tenían de este insumo?**

**E:** En pandemia se refiere

**I: En pandemia, antes de que se tenga el programa**

**E:** Sí, como le menciono desde abril del 2020 nosotros como DIREMID, DIRESA a cajamarca, implementamos el registro de información diaria en nuestros 8 hospitales de la región¿que informaban? Stock en mi IPRES, stock en mi proveedor, consumo, número de pacientes¿no? Número de pacientes hospitalizados, número de pacientes en emergencia, número de pacientes en UCI¿no? Y vuelvo a repetir esto se mantiene hasta hoy, ¿por qué? porque RENOXI me permite registre el consumo de balones entregados pero no necesariamente que sean consumidos. Es así, es así como nosotros este… manejábamos y teníamos la información. Sí la información es en forma diaria

**I: Ya. Entonces, pero y antes del 2020. Ustedes tenían algún tipo de…**

**E:** A través del ICI, de informe de consumo integrado del aplicativo SISMED que es donde se maneja el stock y el consumo de los medicamentos.

**I: Ok, ok respecto por ejemplo al trabajo que hacían con CENARES de manera conjunta con ustedes, entiendo que eso ya fue a ser parte de la gestión de CENARES ¿pero hubo el trabajo conjunto con ustedes?¿Cuál fue la labor ya que tuvieron ustedes en ese momento?**

**E:** ¿Se refiere a las adquisiciones que realizó CENARES?

**I: A las adquisiciones, sí. Así es.**

**E:** La gestión desde DIRESA era igual¿no? Verificar el, el stock a nivel de IPRES y obviamente el stock a nivel de proveedor. Porque CENARES compraba, se me ocurre 10000 m³¿no? Y esos 10000 m³ que posterior también CENARES realizaba el monitoreo, el hospital registraba el stock que tenía la IPRES pero el CENARES no realizaba el monitoreo ,en un inicio de cuánto ya le quedaba al proveedor. Entonces, a ti te he comprado 10 minutos cúbicos pero ya consumiste 9000, te queda 1000 y qué hago ¿no? Porque una compra tampoco era inmediata. Entonces eso CENARES ya… meses después también manejaba¿no? Stock a nivel de proveedor, stock este… metros cúbicos consumidos digamos¿no? Y obviamente prever la oportuna adquisición de oxígeno, que hemos tenido desde cajamarca obviamente que realizar este… sustentos técnicos porque un hospital 'X'tenía una situación crítica de disponibilidad y en el hospital 'Y' tenía un sobre stock, entonces teníamos que llamar a CENARES mediante informes técnicos, realizar una redistribución¿no? Lo que le toca al hospital 'Y' por favor la necesito el hospital 'X'. Hasta que se genere la siguiente compra.

**I: Y en algún momento tuvieron las dificultades, algún inconveniente para digamos las coordinaciones o todo fluyó muy bien y no hubo ningún…**

**E:** En realidad… ¿con CENARES?

**I: Con Cenares o con los proveedores, o con minsa en este… no sé si por ahí también la intervención de DIGEMID**

**E:** DIGEMID no intervino, tuvo un rol pasivo lamentablemente. Pero con los proveedores la comunicación pues hasta medianoche,una de la mañana ,tres de la mañana, porque los hospitales no decían 'sabes que, mira tengo 20 balones y de pronto en 4 horas se me acaba' entonces proveedor ¿dónde estás?¿cuánto tiempo va a demorar la reposición? Con CENARES a veces teníamos (Ininteligible, MINUTO 27:48)oxígeno solamente para 2 días¿cómo va tu compra? Necesito que autorices esta redistribución, la comunicación fue permanente. Hoy en realidad me parece que esto fue fácil, pero en realidad toda la etapa del 2021, 22 incluso ha sido complejo. Entonces las comunicaciones… no no hemos dormido, porque obviamente era una responsabilidad¿no? El hospital decía 'sabes qué me queda tantos… simplemente tengo algunas horas, si no llega el, si no llega el proveedor pueden fallecer pacientes' entonces definitivamente eso no podíamos permitir. Vuelvo a repetir, lo que nos ayudó también fueron las plantas generadoras de oxígeno. Porque ya teníamos 2 fuentes¿no?

**I: ¿Qué tanto así les ayudó? ¿En qué medida?**

**E:** Nos ayudó en el hospital regional de cajamarca,el… 40% del consumo era con la fuente de plantas generadoras de oxígeno, sobre todo en la regional de cajamarca que teníamos 35 camas uci. Teníamos el tanque criogénico de 20000 m³ y en primera ola 2 plantas generadoras de oxígeno¿no? Y sí, ayudó. El 40% De lo consumido era suministro de PGO. Y para los pacientes, obviamente que se encontraban en domicilios en las provincias, el 60% de la producción de las PGO eran para pacientes en domicilio¿no? Entonces las plantas generadoras ayudaron.

**I: OK,ok.¿Cuál tendría que haber sido la labor de la DIGEMID? Usted me dice que ha sido un tanto pasivo¿cuál debería a ver sido su labor?**

**E:** Primero de control¿no? De control de oxígeno, de habernos ayudado a poder realizar compras de plantas generadoras de oxígeno con las condiciones establecidas; porque las plantas generadoras de oxígeno en la región cajamarca y a nivel país son producto de donaciones¿no? Tanto del gobierno local, de la parte privada pero todos sabemos que nadie conocía de plantas generadoras de oxígeno¿no? Entonces ahí, DIGEMID posterior a ello su rol fue era más este… de autoridad ¿no? '¿A ver cumple con las condiciones para que tenga la autorización excepcional?' ¿No?muy frío¿no? Cuando debió ser un poco este… más personalizado el acompañamiento más cercano¿no? Ven el tema de costos, el tema de costos, el tema de la disponibilidad de oxígeno en nuestro país, el tema de que los proveedores cumplan; eso es el rol, el rol rector de DIGEMID.

**I: Ya. Pero ¿ustedes tuvieron comunicación, algún flujo, no sé alguna reunión en la que pudieran expresar la necesidad de mayor intervención de la DIGEMID en este caso?**

**E:** Digo que es pasivo porque no recuerdo haber tenido una coordinación con DIGEMID. Si me dicen '¿con quién es coordinado oxígeno?' Es CENARES¿no? Con Cenares,DIGEMID No lo recuerdo

**I: Porque he conversado con otras personas de cajamarca y me comentaron la enorme dificultad que tuvieron por ejemplo para la compra de una planta generadora¿no?**

**E:** Por eso

**I: Financiada por donación específica, no era del minsa, era de otra institución. Y ahí me llamaba la atención, bueno y a la persona también le llamaba la atención mucho el tema de DIGEMID, entonces quería saber si usted había tenido como contacto, trabajo conjunto, información, como algún tipo de apoyo ahí**

**E:** Claro, las plantas generadores de oxígeno fue algo nuevo y obviamente el tema de una planta generadora de oxígeno es un dispositivo médico electromecánico, entonces nosotros desde medicamento nuestro rol era... Y es garantizar la calidad de oxígeno que producen estas plantas generadoras de oxígeno. Pero el tema electromecánico es competencia de otros profesionales, y vuelvo a repetir,o sea si hubieron charlas¿no? Pero vuelvo a repetir muy… particularmente no percibí ese apoyo en que DIGEMID tuvo que tener mayor presencia. Si nos daban orientaciones, pero vuelvo a repetir, las plantas generadoras eran algo nuevo¿no? Entonces quienes adquirían las PGO los gobiernos locales, la empresa privada¿no?la sociedad civil. Y les adquirían porque era una necesidad extrema¿no? Pero no se adquirió con las condiciones establecidas¿no? O sea la quiero porque la quiero y luego ya cuando se solicita la autorización excepcional que es una… que es un requisito y que obviamente DIREMID , en este caso en las regiones debe conducir, pues también era complejo¿no? Porque ¿me has avisado que ibas a comprar esta PGO? Tampoco avisaban¿no?

**I: En cuanto a, no sé las… qué podríamos decir acerca de las carencias o la principal carencia de recursos como para tema del oxígeno. No solamente esta tener este insumo sino también tener las condiciones adecuadas de infraestructura, contar con personal que vea ese tema, en fin.¿Cómo fue para ustedes ese aspecto?**

**E:** Creo que a nivel país la brecha del recurso humano es alta¿no? Fue alta antes de la pandemia por covid, durante el covid se disminuyó la brecha de recurso humano, sabemos el tema del vínculo laboral de los Cas Covid¿no? Sin embargo actualmente aún existe brecha de recurso humano, sabemos que obviamente no teníamos especialistas, sobre todo en nuestros hospitales y más aún en cajamarca¿no? porque los especialistas se concentran más en las capitales. El tema de recurso humano definitivamente¿no? Siempre ha… siempre… mejoró pero obviamente no fue suficiente, en la pandemia misma. En relación a lo sí yo vi crítico, y lo recuerdo es el tema de los balones de oxígeno, que ahora ya pues tenemos balones en nuestros hospitales que ya no lo requieren porque definitivamente el consumo ha disminuido enormemente¿no? Enormemente. Sin embargo uno de los aspectos, críticos fue la disponibilidad de balones de oxígeno. Si yo tengo más balones de oxígeno en mis hospitales, si tuviese más balones de oxígeno, yo tendría disponibilidad no para 2 días o para 3 días, tendría disponibilidad para 7 días y eso me permitiría al proveedor digamos, que al cuarto día ir nuevamente a realizar la reposición. Los proveedores, proveedor nos ayudó porque obviamente gracias a , gracias a esta cesión de uso de balones de oxígeno los hospitales podían tener almacenamiento de oxígeno, porque como direcciones regionales obviamente no teníamos esta capacidad. Luego ya se realizó la adquisición, pero uno de los puntos críticos fue el tema de balones de oxígeno, la parte del recurso humano¿no? Que vuelvo a repetir... Con la emergencia sanitaria se ha incrementado en la contratación de recurso humano, pero no obviamente , tenemos brechas, sobre todo en cajamarca¿no? Son 860 establecimientos, a nivel país la región con el mayor número de establecimientos.

**I: OK. Y me hablaban también acerca de por ejemplo algunas dificultades en cuanto a infraestructura para la instalación y puesta en marcha en funcionamiento de las plantas de oxígeno¿usted vio algo de eso?**

**E:** No entendí bien la pregunta , por favor

**I: Sí. Me comentaban de que para la instalación de las plantas de oxígeno hay ciertos requerimientos técnicos, que se requieren en cuanto a electricidad, ubicación, espacio básicamente, en fin ciertas condiciones que se requieren pero eran que algo deficientes en varias regiones del país. Entonces¿usted vio en algún momento alguna situación de este tipo en cajamarca?**

**E:** Sí, sí por supuesto. Por supuesto, se tenía la planta y no el espacio¿no? Se tenía la planta y no teníamos las condiciones eléctricas que son básicas, vuelvo a repetir es un equipo mecánico eléctrico. Sí,lo hemos visto en Bambamarca, lo hemos visto en Chota por ejemplo teníamos ese problema se tuvo que coordinar con… con la empresa de electricidad… lo que puedo resaltar es que sí hubo bastante compromiso tanto de la empresa privada, del gobierno local y DIRESA para poder establecer estrategias de mejora finalmente frente a toda la problemática ,y vuelvo a repetir¿no? las plantas generadoras de oxígeno eran algo nuevo hoy podemos entender algo, sin embargo en cajamarca aún es difícil encontrar empresas¿no? Como podríamos encontrarlas en Lima que puedan hacerle un mantenimiento a las plantas de oxígeno, no los encontramos o sea… de que estas plantas generadoras adquiridas fueron ensambladas¿no? Traía una pieza de acá, una pieza de allá… mucho desorden, mucho desorden y que sí, yo he manifestado el equipo de gestión en su momento mi incomodidad¿no? Porque con medicamentos somos responsables del oxígeno¿no? Y también asumimos la responsabilidad de las plantas generadoras de oxígeno pero oye me avisas que tenemos una planta generadora de oxígeno ahorita por qué no me avisaste cuando en el proceso estaba este... El proyecto de implementación o la propuesta de una adquisición para que técnicamente obviamente coordinar con DIGEMID y que nos den pues una orientación y evitar pues la adquisición de plantas que han tenido algunos problemas¿no? O por ejemplo no saber cómo realizar plan de mantenimiento de una planta generadora de oxígeno, que es la situación actual ¿no? porque ya la pandemia pasó pero las plantas las tenemos¿no? Y es una inversión alta ¿no?

**I: Y su dirección digamos¿es como la responsable de verificar esto? Digamos ver el tema de la seguridad...**

**E:**Sí, nosotros como medicamentos, como dirección de medicamentos tenemos que asegurar la calidad de la producción de oxígeno. Las plantas de generadores de oxígeno tienen que encontrarse este bajo la conducción del profesional químico farmacéutico, de ingenieros, de todo un equipo¿no? Desde la dirección de medicamentos ,sí lo que hicimos fue al menos conformar los equipos responsables de la gestión de oxígeno¿no? Que estos equipos obviamente tenían varias funciones establecidas en el decreto supremo 010-2021¿no? Relacionada al reglamento de uso de oxígeno, ello¿no? Pero ya cuando nos hablan del tema mecánico obviamente nuestras competencias no, no están por ahí. Y para ello en DIRESA cajamarca se implementó la, se creó la unidad de gestión de infraestructura y equipamiento, donde también los ingenieros veían el tema de… la operatividad de las plantas generadoras de oxígeno en coordinación ya con DRIEMID¿no? Pero sí se dieron ello

**I:Ok. Digamos es una, es competencia de su área, de su dirección**

**E:** Es un trabajo articulado

**I: ¿Entre ustedes y DIGEMID?**

**E:** Entre nosotros como DIREMID y planeamiento estratégico. Lo que pasa es que las plantas generadoras…

**I: De…**

**E:** De DIRESA.

**I: Ya, ya ok. Quería saber el nivel con el que trabajaban, a lo mejor era minsa por un momento pensé.**

**E:** Debía ser

**I: Ok. Y esta por ejemplo el mantenimiento y todo esto¿ustedes tienen alguna dificultad? Porque no sé si es el caso de todas las plantas de cajamarca pero ya están …**

**--Problemas de conexión---**

**I2: ¿Hola?**

**E:** Este, sí. Se congelo

**I2: Lizzete tiene un problema con el internet ahora pero está tratando de solucionarlo para continuar con la entrevista.Por favor ¿podemos esperar unos minutos?**

**E:** Sí, no se preocupe .Yo me encuentro acá

**I2: Genial, gracias.**

**I2: Quizá en lo que va Lizzete solucionando el tema del internet, de su conexión ¿nos puede ir contando acerca de las problemáticas que ha tenido con la normativa de repente?**

**E:** Sí. En relación al decreto supremo 010 2021 en la cual precisaba que la (Ininteligible, MINUTO 44:59)me parece debería publicar un modelo de plan de mantenimiento de las plantas generadores de oxígeno, no es solamente hasta este 2023 que recién lo publicó¿no? Vuelvo a repetir las PGO eran algo nuevo para nosotros para todos, a pesar que se tienían,se tiene ingenieros ellos tampoco conocían. Entonces esta falta de conocimiento¿no? Ha generado de que las plantas obviamente este... No operen. Por ejemplo actualmente nosotros tenemos 2 plantas inoperativas en el hospital de Celendín y en el hospital de Cajabamba hace más de 1 año¿Y por qué? Porque simplemente no se ha podido generar un plan de mantenimiento correcto¿no? Por otro lado no hay proveedores en nuestra región cajamarca, que se hayan presentado¿no?… que hayan permitido poner en marcha estás plantas generadoras. Entonces sí, sí se tiene problemas relacionados a ello. Lo otro fue también que en el 2020¿no? La resolución ministerial, no recuerdo en este momento el número, pero decía que la pureza de oxígeno podría ser mayor o igual al 90% y el decreto supremo 010-2021 se aprobó en marzo dice que la pureza de la producción, del oxígeno producido por las plantas generadoras de oxígeno no debería ser menor al 93%¿no? Entonces aquellas plantas generadoras adquiridas en el 2020 tenía un porcentaje de pureza del 90%¿no? Y obviamente contradictorio con la disposición que se emite en el 2021 mayor al 93% entonces por ahí de pronto serían uno de los eh… de las controversias relacionadas con la normatividad. Después por lo otro es muy preciso este… lo dispuesto en el decreto 010 2021. Este año también se aprobó¿no? Lo relacionado a la calidad de oxígeno, ya podemos nosotros como medicamentos mencionar este… cuáles son las condiciones. Un ratito está…

**-Entró una llamada-**

**E:** Sí, como le mencionaba ya en este 2023 también se aprueba lo relacionado a las, los requisitos de calidad que deben… y que debe cumplir las plantas generadoras de oxígeno¿no? Entonces el minsa ha demorado y las regiones hemos tenido que... Salvarnos solas podríamos decir¿no? Me dice la licenciada Lizzete volverá a iniciar sus equipos. De lo contrario podríamos terminar vía telefónica

**I2: Ok, ¿entonces le esperamos un momento?**

**E:** Sí, la espero Claro que sí.

**I2: Muchas gracias**

**-Se reanuda grabación-**

**I: Entonces doctora, le estaba preguntando acerca del tema de las carencias que se tuvieron en la región por el tema de infraestructura, el personal y usted me decía que todo… si bien el tema de personal estaba medio resuelto porque se había contratado a más personal por el tema de la emergencia sanitaria y qué más bien por el tema de la infraestructura había sido sí un poco complicado por qué no se tenían las condiciones para la instalación y funcionamiento de las plantas. Y eso me lleva a pensar sí hay alguna**

**normativa, no sé si, entiendo que a nivel nacional hay una normativa que regula el tema del oxígeno medicinal pero no sé si hay algo como más específico que ustedes manejen la dirección le cajamarca¿cómo hicieron ustedes para ver esta parte más normativa, más legal, más de sí eso más normativa de cómo debería trabajar en el tema del oxígeno medicinal?¿Alguna directiva tal vez a través de la DIRESA?**

**E:** El oxígeno medicinal es un medicamento¿no? Y como tal teníamos o tenemos 3 fuentes de suministro, hasta cuatro ¿no?el oxígeno líquido a través de los tanques criogénicos, el oxígeno gaseoso balones de oxígeno, las plantas generadoras de oxígeno y también los concentradores de oxígeno. En relación a las plantas generadoras de oxígeno que veníamos conversando, donde eso… estos equipos… mecánicos eléctricos¿no? En los cual es el país obviamente tenía desconocimiento sí, se tuvo falencias. Y vuelvo a repetir la sociedad civil, la empresa privada, los gobiernos locales realizaron la adquisición de estas plantas generadoras de oxígeno y que muchas de ellas … no cumplían con las condiciones que la DIGEMID exigía; y qué es lo que la DIGEMID exigía, y qué es lo que se exige ¿no? Que todo medicamento, dispositivo médico, producto farmacéutico para poder ser comercializado en el país tenga su registro sanitario, en este caso de la emergencia sanitaria lo que se solicitaba para las plantas generadoras de oxígeno era la autorización excepcional¿no? Sin embargo muchas de nuestras plantas generadoras de oxígeno no, DIGEMID no ha logrado otorgarles esta autorización excepcional. Y obviamente se convierte en un problema, por qué, porque esta autorización excepcional solamente podía emitirse en, durante la emergencia sanitaria. Una vez que termina la emergencia sanitaria, DIGEMID ya no va a exigir una, requisitos digamos básicos,DIGEMID va a exigir requisitos muy complejos, no para que se le otorgue una autorización excepcional, sino para que cuenten con el registro sanitario. Y DIGEMID obviamente.. Ellos este… en metieron algunos requisitos exigiendo simplemente la autorización excepcional porque sabían que a nivel país ninguna planta generadora de oxígeno cumplía con las condiciones, entonces esto se convierte en un problema. Vuelvo a repetir,DIGEMID , no he visto su acompañamiento porque como ustedes mencionan , a ver cuál es el paso a paso, cuáles son los requisitos que debería tener una planta generadora de oxígeno no se tenía. Al inicio en el 2020 fue un tema que para los gobiernos locales que realizaban la adquisición y puesta en funcionamiento de estas PGU, PGO perdón, salieron algunos lineamientos. Pero luego de ello ¿qué más podíamos exigir? ¿Qué más sabíamos? No sabíamos mucho y quien lo sabía pues parecía que lo guardaba este.. Entre cuatro llaves¿no? Luego ya la DGOS este… generó algunas capacitaciones pero… también digamos muy,muy macros, muy, no muy específicas como debió haber sido este manejo de plantas generadoras de oxígeno¿no? Debió ser algo más específico pero se dio de modo muy general. Entonces eso sí fue un problema y nosotros obviamente como, como dirección de medicamentos sí establecimos una… una acta de inspección para verificar condiciones adecuadas de las plantas generadoras de oxígeno de nuestra región, sí lo establecimos como ,como DIREMID

**I: Ok. Y exacta era con la que ustedes supervisaban, revisaban, verificaban**

**E:** Sí así es. Verificamos cuando ya este, en el 2022 se adquiere… algunas… en coordinación con el gobierno local algunas PGO DIRESA -cajamarca ya obviamente teníamos un poco, un panorama digamos un poco más claro¿no? Pero al inicio no. Entonces sí hemos tomado como modelo esta, esta acta en función a … a lo que establece la normatividad y obviamente a..a las coordinaciones con DIGEMID ¿no? A lo que ya pedía SUSALUD, entonces hemos de alguna manera enriquecido y se a recopilado todo lo que debe exigirse a las PGO

**I: Ok.¿Y esos mismos, digamos lineamientos se mantienen hasta hoy o ya hay otro tipo de directiva…?**

**E:** Se mantiene ,el acta ,obviamente con algunas modificaciones en función ya a la directiva administrativa relacionada a la calidad de oxígeno que fue aprobada recién este año. Y también en relación a, al plan de mantenimiento de las PGO que también fue aprobada este año, recién este año en el 2023 después de 3 años de pandemia recién el minsa aprobó sus documentos normativos¿no? **(Ininteligible, MINUTO 58:18)** inoportuno.

**I: Ok. Si hablásemos de una nueva política sobre el tema del oxígeno¿cuáles usted considera que deberían ser las áreas clave en la que debería centrarse esta nueva política?**

**E:** En relación a la política de oxígeno... Tendría que verse lo relacionado a la calidad, debería mantenerse¿no? lo que establece de repente algunas mejoras, siempre es importante el monitoreo de la pureza de la calidad por un profesional¿no? con las competencias. Lo otro en relación a políticas de oxígeno puede ser de los costos¿no?

Y los proveedores(Ininteligible, MINUTO 58:11) sería un tema de monopolio¿no? Y que obviamente el minsa¿no? El minsa a raíz de esta experiencia que tuvo de adquisición, en emergencia sanitaria, debería también incluir dentro de los productos a ser requeridos,a ser adquiridos mediante compra corporativa¿no? Ya tiene un modelo, ya tiene un modelo entonces debería servirle para obviamente generar una compra corporativa , que las compras corporativas obviamente su gran ventaja es economía (Ininteligible, MINUTO 59:55) obviamente optimizar nuestro presupuesto

**I: Usted me menciona el tema de los presupuestos y del proveedor¿ A qué se está refiriendo con ello?**

**E:** Lo que pasa es que CENARES conduce la compra corporativa¿no? A nivel país, ellos lideran la compra corporativa que obviamente, por ejemplo un paracetamol que me puede costar 0.10 céntimos en cajamarca, el CENARES mediante compra corporativa obviamente lo va a adquirir a 0.05; entonces definitivamente financieramente es una ventaja ¿no? Entonces lo mismo puede suceder con el oxígeno, a nosotros nos puede costar 16 soles pero a lo mejor mediante compra corporativa el costo se minimiza. Vuelvo a repetir, ya se tiene la experiencia del Covid¿no? Entonces adquirir oxígeno este… no sería complejo. Lo otro es que ya tenemos las plantas generadoras de oxígeno ,que es una inversión grande y qué estás obviamente deberían mantenerse operativas, para eso se debe garantizar el presupuesto de mantenimiento, el presupuesto para el funcionamiento que implica los costos de recurso humano, los costos de energía eléctrica… debemos ya de ver, asegurar una… un proceso de distribución y transporte óptima del oxígeno. Por ejemplo nosotros tenemos plantas de oxígeno en hospitales pero estas deberían proveer a nuestros establecimientos del primer nivel de atención, para ello obviamente se requiere una logística¿no? Relacionada al tema de distribución y transporte, al tema de personal. Entonces esos costos deberían asegurarse y deberían ser parte de una política , en este caso,de regulación de oxígeno¿no? Ya abastecimiento, uso y regulación de oxígeno

**I: Hasta ahora hemos hablado acerca de la gestión propiamente desde su dirección y también un poco del minsa. Pero cómo ha sido la intervención de los actores gobierno regional, los gobiernos locales¿cómo ha sido o como ha visto usted su interés…**

**E:** Muy buena, muy buena. El gobierno regional, el gobernador estuvo muy de cerca¿no? Con DIRESA cajamarca gestionando la adquisición, la distribución oportuna de oxígeno dede CENARES ¿no? Siempre hemos tenido el respaldo del gobierno regional y creo que eso fue vital obviamente para poder garantizar la disponibilidad de oxígeno en nuestros, en nuestros establecimientos de salud. Con el gobierno local también¿no? Los gobiernos locales han realizado la adquisición de las plantas generadoras de oxígeno, con la empresa privada de igual manera y la sociedad civil. Entonces sí, sí sí ha tenido el respaldo del gobierno regional, lo reconocemos; muy muy cercano el interés por esto.

**I: Si usted, ya como para ir cerrando la conversación con usted. Si usted pudiera señalar¿cuáles fueron los principales retos que tuvieron que enfrentar con respecto al tema del oxígeno durante la pandemia, cuál pudiera señalar? y si pudiera señalar 2 o 3 exactamente**

**E:** El reto para garantizar la disponibilidad de oxígeno en la pandemia, el reto fue obviamente garantizar el suministro de oxígeno desde el proveedor, del proveedor en cajamarca para nuestros hospitales. Considerando que solamente teníamos un único proveedor en cajamarca y que el proveedor no tenía una, después ya tuvo un tanque criogénico pero al inicio no, y que las distancias de cajamarca a nuestras provincias, caso cutervo solamente 6 horas y que por ejemplo en cutervo no tenían una planta generadora, tienían una planta generadora tienen de 10 $m^3$ de la productividad pues no lograba asegurar la necesidad de consumo¿no? El reto grande fue eso y el otro gran reto fue obviamente poder cerrar la brecha de balones de oxígeno¿no? En una segunda ola, sin balones de oxígeno obviamente que podíamos hacer¿no? Sin plantas generadoras. Entonces eso fueron los 2 grandes retos. Y el otro gran reto fue este tener información oportuna¿no? Información oportuna donde los jefes de farmacia de nuestros hospitales pues al día¿no?y monitorizaba ellos también 'sabes que ya me queda, tengo tantos pacientes, el consumo por pacientes se incrementó, sabes qué faltan 6 horas y si no se repone en 2 horas oxígeno pues vamos a tener una situación crítica' creo que fueron los 3 grandes retos.

**I: Usted durante la conversación que hemos tenido me he ido comentando como las soluciones de esas dificultades que han tenido. Pero ¿diría usted que funcionó todo? O sea ¿llegaron a resolver estas dificultades con las medidas ,las estrategias que usaron o(Ininteligible, MINUTO 1:05:26)?**

**E:** Yo creo que sí. Porque si no hubiésemos resuelto y actuado con oportunidad o haber… no previsto sino, si no hubiésemos digamos previsto esas situaciones críticas, definitivamente ¿no?algunos de nuestros hospitales se hubiesen quedado sin oxígeno, situación que no sucedió¿no? Incluso cuando el proveedor en algún momento no tenía disponibilidad o su recurso humano mismo no… no aseguraba el suministro a tiempo, las plantas generadoras de oxígeno del hospital regional de cajamarca ayudaron a poder suministrar oxígeno a nuestros hospitales de cutervo, cajabamba, cajamarca, Simón bolívar. El hospital de bambamarca también ayudó su planta generadora. Entonces todo fue un trabajo coordinado, articulado¿no?DIRESA cajamarca tuvo el respaldo del gobierno regional, de los gobiernos locales, de la empresa privada, de la sociedad civil, del ministerio de salud a través de CENARES; entonces no, no trabajamos solos porque obviamente…nadie pudo hacerlo solo el poder hacer frente de la pandemia. Entonces puedo rescatar el trabajo integral, coordinado de los diferentes… a nivel interinstitucional¿no? Que nos permitió afrontar la pandemia ,sobre todo con el consumo alto de oxígeno que se tuvo. Eso es lo que podría precisar en relación a la pregunta

**I:¿Hola?**

**E: Sí la escucho**

**I: Le decía que ¿cuál es la situación del oxígeno medicinal hoy en Cajamarca?**

**E:** Actualmente se cuenta con disponibilidad de oxígeno medicinal, las fuentes de suministro son oxígeno líquido en el hospital regional de cajamarca a través de su tanque criogénico ellos tienen operativas cuatro plantas generadoras de oxígeno, lamentablemente como le mencionaba en nuestras provincias en... 2, en 3 de nuestras provincias las plantas generadoras de oxígeno no se encuentra operativas por falta de mantenimiento, esa falta de mantenimiento se ha originado con la falta de proveedores y probablemente por la falta de conocimiento de poder generar un plan de mantenimiento. Se encuentra en tránsito, se encuentra en tránsito… la contratación de una empresa que realice este plan de mantenimiento, las plantas generadoras de oxígeno en nuestras otras provincias no se encuentran operativas no porque porque se encuentra en una lograda , sino el consumo en realidad de oxígeno es bajo versus el costo que significa operarlas¿no? Por ahí por ejemplo hay un problema. En pandemia nos sirvió pero después de la pandemia el consumo ha bajado y obviamente… esos consumos se van a mantener¿no? es la tendencia. Actualmente nosotros estamos también realizando la adquisición de oxígeno gaseoso con el financiamiento de la entidad¿no?

**I: Ok. Si tuviéramos nuevamente una pandemia ahora, cuál sería la situación del oxígeno en cajamarca**

E: si tuviésemos una pandemia actual, una pandemia con los niveles de consumo de…que se observó en Covid…

**I: Similares, así es**

E: Actualmente, con el stock que tendríamos actualmente en el hospital regional de cajamarca donde se tienen las camas uci sí se podría controlar. En nuestros hospitales de Cajabamba obviamente y Celendín… tendríamos una disponibilidad para y 4, 5 días con esos picos máximos ¿no? Que se vivió en pandemia y obviamente tendríamos que realizar coordinaciones de adquisición y compras inmediatas. Pero en el regional donde tendríamos, y en jaén, donde sobre todo en el regional donde tenemos el mayor número de camas uci sí se tendría la disponibilidad, hace más o menos 3 semanas que se realizó una… se realizó una evaluación del estado de esas plantas generadoras de oxígeno y cuál sería, cuál sería la disponibilidad¿no? Ante una situación semejante a covid y si garantizamos la disponibilidad.

**I:Finalemente ¿Qué lecciones clave podría compartir usted acerca del oxígeno medicinal que ha ocurrido durante la pandemia? Si pudiera mencionar 2 o 3**

E: ¿Lecciones me indicó?

**I: Así es, lecciones clave, lecciones yo hubiera podido usted identificar respecto de todo lo ocurrido durante la pandemia y también con miras tal vez a que podría ocurrir. En fin**

E: Las lecciones aprendidas ¿no? Las lecciones aprendidas… uno es que se debe trabajar en forma coordinada, articulada e integral en una situación de crisis. Dos de que la información es clave, información real, en tiempo real es clave para tomar decisiones oportunas. Tres es el compromiso¿no? El compromiso obviamente que debemos tener todos a todo nivel¿no? Y yo pienso que esas son las lecciones aprendidas, la comunicación oportuna que va con el tema de trabajar de forma articulada e integral. Yo decía ¿por qué tú compras plantas generadores de oxígeno y recién cuando recién te das cuenta que va a producir oxígeno y que medicamentos es el responsable recién me avisas? Cuando debiste de avisarme antes porque somos todos parte del proceso. Entonces el trabajo es articulado e integral y en un equipo de gestión ,por eso es un equipo de gestión, porque todos somos importantes¿no? Todos los procesos son transversales a todos. Esas fueron las lecciones aprendidas¿no?

**I: Doctora una consultita final. Usted ha hablado acerca de los sistemas de información, están ,entiendo que ustedes que tienen, tenían en el 2020 generaron un sistema a nivel de cajamarca reportes. Y luego está ahora el que tenemos a nivel de minsa. Para usted ¿estos son útiles¿ ¿reportan realmente lo que se tiene en la actualidad o hay alguna dificultad por ahí para generar esos reporte?**

E: En el caso del RENOXI obviamente depende la información de, de quien registra la información¿no? Por decir se registra manualmente ,si derrepente yo como sé si es información es real¿no? Lo que necesitaríamos es… tener, generar un sistema¿no? En el cual me permita saber con certeza cuál es el consumo de oxígeno real,en (Ininteligible, MINUTO 1:13:49) de hospitalización¿no? Que se pueda realizar mediante tecnología, se puede realizar. Actualmente ayuda, pero yo no sé si eso realmente es cierto lo que me reporta el RENOXI, que debería ser cierto porque es una declaración jurada¿no? Pero ahí como repito el consumo es en función a los balones que se entregan desde mi servicio de farmacia o desde el servicio de farmacia, si yo entrego 3 balones de 10 $m^3$ significa que se han consumido 30 metros pero no se han consumido, se están entregando el consumo probablemente es menos. Entonces eso es lo que sí ,deberíamos tener equipos¿no? Que me mira cuál es mi consumo real y eso es básico porque una emergencia sanitaria, o sea recién me voy a poner a verificar cuál es lo real, cuál es mi stock, cuál es… si la persona no cumple¿no? Como también desde farmacia este… el responsable de farmacia, los licenciados en enfermería le están enviando esta información correcta¿no? Se han implementado… algunas matrices, fórmulas pero creo que sí deberíamos apuntar a conocer en tiempo real cuál es el consumo de oxígeno. Y se puede realizar, se puede realizar lo habíamos visto con una…con una este… ONG ¿no? Que también veían y nos preguntaban lo mismo '¿usted cree que el RENOXI ayuda o no ayuda?'¿no? Entonces por ahí se pueden hacer mejoras y creo yo que podríamos hacer muchas mejoras.

**I: OK. Bien doctora muchísimas gracias por su tiempo, su disponibilidad y disculpe estas fallas. La verdad es la primera vez que me ocurre, no sé qué está pasando con la conexión.**

**E:** No, no se preocupe gracias a ustedes, gracias por la oportunidad ,yo espero que les ayude de pronto un poquito de lo que les he comentado. Particularmente fue un gran reto poder conducir digamos está… poder conducir y asegurar la disponibilidad oportuna de oxígeno en nuestras IPRES que si no hubiese sido por el trabajo coordinado, vuelvo a repetir, a nivel de gobierno regional, empresa privada, los gobiernos locales, los diferentes directores del equipo de gestión de DIRESA quizás no hubiésemos tenido resultados satisfactorios , puedo llamarlo el día de hoy¿no? Y poder contarlo porque sí, particularmente creo que todos los esfuerzos que hoy ,como les decía no, de repente hoy se cuenta de una manera tranquila, parece que todo fue tranquilidad, no hubo estrés, no hubo preocupación, no hubo nerviosismo pero en el momento sí, o sea trabajábamos así ,a 1000 por hora y… '¿tienes EPP y tienes oxígeno?' Eran los dos grandes artistas ¿no? Y ahí estaban, entonces fue un trabajo, vuelvo a repetir, no de uno solo fue un trabajo integral. Y un compromiso sí debo reconocer de mis colegas responsables de los servicios de farmacia que ellos también tenían esa responsabilidad enorme ¿no? Garantizar la disponibilidad de oxígeno, ver los balones, todo, todo mundo en realidad en ese momento.

**I: Muchísimas gracias doctora, gracias**

**E:** A ustedes

**011 Entrevista**

Meeting started: 12/7/2023, 11:00 Am
Participantes: E, I
**I: Investigador**
**E: Entrevistado**

**I: Solo para confirmar con la grabación**

E: Estoy de acuerdo con la grabación

**I: Hoy es 12 de julio del 2023 voy a comenzar una entrevista con u actor vinculado al tema del oxígeno medicinal por el estudio que la Universidad Peruana Cayetano Heredia viene realizando como para ir contextualizando podría usted comentarme cuál es su labor, en que área ha trabajado o trabaja que tenga que ver con el oxígeno medicinal, cuanto tiempo ha estado en el puesto si ha estado en el mismo puesto hace tiempo como para ir conociéndolo.**

E: De acuerdo primero agradecer por la entrevista para poder aportar en el desarrollo de la investigación que ustedes están realizando, soy médico de profesión y con una especialidad de administración de salud también en la Universidad Peruana Cayetano Heredia y una maestría concluida, para resumir mis últimas actividades desde el año 2017 yo he elaborado en el Ministerio de Salud en la Dirección General de Operaciones en Salud, que tiene a su cargo 2 direcciones ejecutivas, la dirección de monitoreo y la dirección ejecutiva de infraestructura y equipamiento. Entonces, desde esos tiempos ya mis actividades han estado directamente relacionados con uno de los factores de producción críticos en los servicios de salud, que es el equipamiento y dentro del equipamiento están un conjunto de dispositivos relacionados con la aprovisionamiento de oxígeno medicinal en los servicios de salud

**I: Ahora, respecto al tema del oxígeno medicinal previo a la pandemia, ¿cómo era la situación de abastecimiento del flujo, en fin, de todo lo que se relaciona al tema del oxígeno y que era provisto en los establecimientos de salud?**

E: A ver en los últimos 30 años a más inclusive los servicios de salud se han venido abasteciendo de oxígeno medicinal a través de proveedores, y, digamos, privados o proveedores externos y la principal fuente de generación de oxígeno era es oxigeno criogénico, oxígeno líquido que llega a los establecimientos a través de tanques criogénicos o Izo tanques que también les llaman es decir este ha sido el 95% del aprovisionamiento de oxígeno medicinal, en el Ministerio de Salud teníamos registrado antes de la pandemia 9 plantas de oxígeno medicinal pero estas eran plantas tipo PCA plantas muy pequeñas que producen entre 10 metros cúbicos a 30 metros cúbicos en promedio y que algunas de ellas estaban conectadas a red inclusive algunas no eran muy utilizadas en verdad, la mayor la fuente de oxígeno medicinal viene actualmente sigue siendo la fuente por proveedores privados.

**I: Y era suficiente es decir yo sé que el tema de la pandemia abierto por las características propias del COVID y cómo es que afectó a las personas se necesitaba se requería un volumen muy alto de oxígeno, pero previo a la pandemia en algún momento ustedes detectaron que hacía falta contar con más plantas o contar con mayor abastecimiento, o era suficiente?**

E: Antes de la pandemia no hubo alguna alerta, algún requerimiento de oxígeno medicinal que no haya sido atendido digamos que lo que había en el mercado y en las fuentes de origen del oxígeno era suficiente

para el volumen de demanda que tenían nuestros establecimientos de salud a nivel nacional, por todo tipo de institución inclusive.

**I: Ok. Y el abastecimiento solamente me dice de usted, lo tenían a través de los proveedores externos y de estas plantas que se contaban, que me dice que son 9 únicamente verdad ¿dónde estaban ubicadas estas plantas en lima o en las regiones?**

E: Había unas cuantas acá en Lima, pero la mayoría esta fuera eran como 4 o 5 fuera y unas cuantas aquí en Lima, eran como 3 a 4 acá en Lima y el resto estaba en otros hospitales.

**I: Alguna región en especifico**

E: No, justo quise revisar dónde estaban y pedí la información, pero no lograron alcanzarme tendría que buscarla y, si me es posible, si la ubico se las puedo alcanzar posteriormente.

**I: Si por favor, porque sería interesante saber dónde estaban ubicadas, entonces ya me dice que era como suficiente la cantidad de oxígeno que teníamos no hacía falta más y esto estaba propiamente el tema del oxígeno medicinal respondía a una normativa específica en ese entonces hablo de antes de la pandemia para un poco para mirar también si la parte normativa que han ido cambiando hasta ahora o siguen siendo las mismas.**

E: De hecho que antes de la pandemia, como que la normatividad si bien es cierto que se mencionaba mucho de una disposición que dio el ministerio de salud en el cual se solicitaba que la concentración de oxígeno sea del 99% pero no se había digamos en las implicancias de ese acuerdo porque como el sector privado lo asumía, o sea, nosotros le pedíamos señores, LINDEY y señores PRACSAI, señores OXIMAN que son los proveedores privados y hay otros más inclusive, LINDE y PRAXARSE se juntaron, OXIMAN hay otro más que no recuerdo pero igual también les puedo alcanzar esa información y como ellos eran los que tenían que asegurar las concentraciones máximas poco que no se vio las implicancias de que al producir oxígeno ahora el sector público también tenga que cumplir ese requisito y ahí es donde aparecieron algunas restricciones porque las plantas de oxígeno no todas tienen esa capacidad de llegar a un 99% entonces porque son plantas con una tecnología muy particular son TCA y de fábrica ya vienen condicionadas a ciertas características que están en la normatividad internacional OMS, OPS no piden 99% entonces en el mercado internacional también había ese condicionamiento por eso es que la normatividad no tuvo un énfasis en la pre pandemia ya se sintió la presión en la misma pandemia donde se vio la necesidad de regular primero el abastecimiento extraordinario y, segundo, ya el aprovisionamiento regular que debería de implementarse en el país y de eso lo dispuso inclusive una ley específica de aprovisionamiento de oxígeno donde se establece que la concentración debe ser 93% a más.

**I: Ese digamos, la normativa que tenemos que se ha considerado y está vigente hasta ahora.**

E: Si, correcto en el 2020 y 2021 se dieron estas nuevas normativas para el manejo de la pandemia.

**I: Hay una ley también la 28905, que es ley para la Atención De Situaciones de Emergencia o desastres naturales esto se ha estado aplicando, se aplicó, fue de utilidad durante el tiempo de la pandemia.**

E: No, no tuvimos alguna incidencia en que se tenía que tomar en cuenta esta ley para alguna condición.

**I: En cuanto a la normatividad con la que se maneja en este momento el tema, usted considera que está acorde ahora a la necesidad o a la realidad que nos ha dejado la pandemia o será que hay la necesidad de hacer algún ajuste alguna precisión.**

E: Por ahora mi posición técnica es que lo que tenemos ya es suficiente para establecer una adecuada relación entre la oferta y el conjunto de tecnologías y dispositivos para oxígeno y lo que tiene la demanda, ósea la demanda al pasar la pandemia, ha disminuido significativamente y lo que la pandemia ha dejado es una oferta que ahora hay que evaluar cómo utilizarla porque cubrió ciertas brechas generadas por la pandemia y ahora esas brechas ya no están entonces hay que evaluar qué hacemos con la oferta que se generó en los tiempos de la pandemia.

**I: Y cómo están viendo ese tema, quién es el responsable, el área, la dirección que debería haber este aspecto es su dirección.**

E: Es una de las direcciones, pero en mi dirección es la que ve la parte de las tecnologías de tanques de oxígeno de concentradores, ventiladores que usan oxígeno, ve tanques criogénicos, plantas pero nosotros no hacemos análisis de la demanda en función de los pacientes eso lo tiene que ver otra área del Ministerio de Salud u otras direcciones, e inclusive en los niveles subnacionales, los gobiernos regionales que están a cargo de la atención de los pacientes con sus instalaciones sus redes sus establecimientos tienen que hacer el análisis de la demanda y, en función de eso establecer los requerimientos de oferta entonces el tema es multidimensional, multifactorial, multiinstitucional, inclusive considerando la fragmentación de nuestro sistema como es salud, como sanidad de las fuerzas armadas y policiales, sector privado, etcétera.

**I: Y pensando, por ejemplo, en los Gobiernos regionales y locales ¿Cuáles son como los retos que se tuvieron para garantizar el compromiso de estos Gobierno, repito, regionales y locales? Para el tema del oxígeno.**

E: De hecho como era de esperarse en una emergencia de este nivel, que fue una emergencia mundial en los gobiernos regionales tuvieron la iniciativa de poder ellos mismos abastecerse de algunos tipos de generaciones de oxígeno muchos de ellos han adquirido plantas de oxígeno y han enfrentado con sus recursos y no solamente a nivel de gobiernos regionales, sino los gobiernos locales, llámese municipalidades provinciales, municipalidades distritales a nivel nacional tomaron la iniciativa de comprar plantas de oxígeno esas plantas actualmente están asignadas el 90% más de ellas a nuestros establecimientos en los ámbitos regionales.

**I: Y está respuesta que dieron los gobiernos locales, regionales, provinciales usted de lo que pudo conocer, observar cumplía con los requerimientos técnicos cumplía con una planta el instalar, exige que cumpla ciertos requisitos e infraestructura en personal y tiene una serie de aspectos eso fue así respondieron ¿Estaban de acuerdo a esas exigencias? o más bien, fue algo más difícil de cumplir tal vez o no sé si tuvieron alguna dificultad para hacerlo. Le preguntaba por estas iniciativas que tuvieron los gobiernos regionales locales para responder a esta necesidad de contar con oxígeno en la población y la pregunta era si estas plantas estuvieron o respondían a los requisitos o las exigencias que técnicas que se requieren para contar con esos equipos de manera adecuada.**

E: Esa es una pregunta muy importante porque en principio las adquisiciones en el sector público están muy reguladas en los recursos de las municipalidades han tenido que ejecutarse en función de los procedimientos tanto de la ley de presupuesto público como de la ley de contratación entonces primero ha tenido que haber disponibilidad presupuestal; segundo, que se han tenido que regir por los procedimientos tiene que ver un área usuaria tiene que ver un requerimiento y tiene que ver una especificación técnica. Entonces digamos que en nosotros, en nuestra página web tenemos

especificaciones técnicas de plantas de oxígeno o algunas municipalidades se agenciaban de estas especificaciones técnicas pero digamos que no tenemos y un estudio claro que nos diga estas vinieron con especificaciones que permitieron la compra de estas plantas, porque también hemos tenido otra variable muy importante que han sido las donaciones y las donaciones han venido por embajadas, han venido con instituciones privadas sociedad en las organizaciones de la sociedad civil, la Iglesia todo el mundo se anotó y empezó a apoyar a diferentes ámbitos, y han aparecido estas plantas entonces en la actualidad tenemos 497 plantas distribuidas a nivel nacional entonces en menos de 2 años pasamos de 9, 10 plantas a más de 450 plantas actualmente en nuestro sistema de información tanto en el observatorio de bien como en el RENOXI que es un sistema de información a cargo de DIGEMID figuran 497 plantas de oxígeno medicinal y de esas 497, 127 están operativas, pero apagadas porque no tienen demanda y tenemos alrededor de 68 plantas que están inoperativas y que pueden a través de los mantenimientos o preventivos o correctivos recuperarse y volver a este a estar operativas pero muchos de nuestros funcionarios regionales y acá DIRIS o Lima nos dicen ¿Cómo voy a arreglar una planta si no necesito oxígeno? ¿Como justificó gastar en un mantenimiento cuando no necesito oxígeno? Entonces ahí tenemos un problema crítico muy complejo que requiere de una inteligencia especial de un equipo de trabajo especial para definir el rumbo de estas plantas.

**I: Esto es más o menos alrededor de 300 que estarían no operativas**

E: Y tenemos 301 operativas. Operativas y funcionando y produciendo oxígeno.

**I: Entonces tenemos que 68 podría no estarlo si no se vuelven a reparar o hacer un mantenimiento que se podrían perder digamos**

E: Se podrían perder.

**I: Ok, ¿Qué interesante esto? Y sabe usted en dónde es que se digamos, se concentran estas plantas que no están operativas entre regiones.**

E: Las plantas inoperativas si las tengo, las tengo mapeadas y las puedo alcanzar la distribución hay 11 11 en la libertad tenemos de los de mayor número, 8 en acá en Lima metropolitana, 6 en Piura, 7 en Cajamarca y así tengo la lista detallada se la puedo pasar.

**I: Ok sí por favor para a saber, o sea, para ver si en qué regiones es que tenemos más plantas reconocer un poco la distribución y también ver dónde se necesitan ponerle algo más de atención al tema del mantenimiento que es una parte importante.**

E: La pregunta que viene a continuación sería bueno que se han hecho por estas plantas inoperativas y cuál es la respuesta que ha tenido el Ministerio de Salud desde el mismo 2021 a pesar de que todavía estamos en plena pandemia ya programamos recursos alrededor de 4 o 5 000000 de soles para mantenimiento de plantas y se dio la disposición que se distribuyan estas recursos independientemente de qué institución fuente de origen de estas plantas, si eran donadas, si eran de gobiernos regionales, se les asignó un determinado monto en el 2021 en el 2022 a través de la ley de presupuesto público ósea ya

vino programado con un año anterior, se programaron un número de plantas más de 100 a 30 plantas se programaron para mantenimiento y en este año 2023 en el presupuesto público tanto en lima metropolitana como en gobiernos regionales hay un monto por arriba de los 15 000000 de soles asignados para la prevé para el mantenimiento de plantas de oxígeno y el próximo año estamos programando 24 000 000 de soles.

**I: Ok. Pero una cosa es el presupuesto programado y otro el ejecutado sabe usted si en las regiones se ha logrado ejecutar este presupuesto y darle efectivamente el mantenimiento a las plantas con las que cuentan en cada región.**

E: Es una muy buena observación, el nivel de ejecución está en alrededor del 85 al 90% de lo asignado eso es lo que hemos evaluado en los últimos requerimientos porque el mes nos dice hoy cuánto vas a programar? Sí, pero que siempre se mira hacia atrás y cuánto ejecutaron ¿Cómo ejecutaron? ¿Por qué ejecutaron? Y uno de los principales problemas que se tiene es que estas plantas al tener algunas todavía no están registradas en el sistema de información de gestión de los bienes lo que llamamos el SIGA patrimonial

**I: ¿Que qué dificultades? ¿A qué se refiere con esto?**

E: Por ejemplo, no están saneados en los aspectos de la liquidación de los CEOAR, la liquidación logística, el registro patrimonial, entonces son procedimientos administrativos que tienen que cumplirse para poder decir a este bien ya es mío y si ya es mío entonces este le puedo destinar recursos para sus mantenimientos.

**I: Se refiere usted a que, por ejemplo, que aquellas plantas que fueron donadas todavía están en proceso sesión no sé si ese es el término correcto y no ha sido oficializado aún y no es parte del digamos del patrimonio del MINSA.**

E: Claro, eso es.

**I: Ya y cuánto tiempo más o menos toma este procedimiento, ¿cuánto puede normalmente tardar de una manera regular? entiendo que la cuestión administrativa en el sector público puede ser a veces un poco más lento porque se debe cumplir una serie de procesos. pero un promedio, cuanto más o menos debería de tomar de tiempo.**

E: Debe demorar más o menos unos 60 a 90 días más o menos un procedimiento regular. Pero en la emergencia han aparecido situaciones un poquito complejas, como, por ejemplo, y si hubo alguna observación al bien, si el bien cumplió con las características y las especificaciones, y se hizo en un periodo de gestión de una autoridad anterior la nueva autoridad ya no le da prioridad entonces hay varios factores.

**I: Entiendo y en cuanto a los aspectos técnicos, nosotros sabemos que, bueno, todos en realidad toda la población sabe que durante la pandemia hubo digamos empezaron a construir no a nivel nacional plantas en fin para cubrir las falencias y llegaron algunas de afuera importadas y la prisa por construirlas por a llevarlas a las regiones puede que tal vez haya a construir algunas plantas que no necesariamente tienen los requerimientos técnicos específicos como que una planta nueva porque fueron como elaboradas me comentaban que asumía unas piezas y otras las recomponían en fin salvar como para salvar el momento de la dificulta no sé si esas todavía son las condiciones de las plantas en este momento.**

E: Mejor le comento algo con un ejemplo al hospital Loayza llegó una planta de 160 metros cúbicos, una planta bastante y digamos capacidad a diferencia de las de 20 de 30 que estaban instalando pero este era de 160 una planta donada por una empresa minera y ya pues se hizo la instalación para nosotros era como una bendición caída del cielo porque este la gran capacidad de la planta, ya esta planta no ha funcionado ni durante la pandemia ni post pandemia porqué arreglar esa planta de oxígeno era más caro que comprarse una nueva planta

**I: Y a que se debió**

E: Como era donada que este la empresa dijo ya yo la pongo en tal lugar y cuando ya el bien está donado entonces uno digamos no hace o no es que primero la pruebo la planta que me produzca oxígeno y después ya digo, ya la plata estaba instalada y cuando se fueron a hacer las pruebas de funcionamiento ahí encontraron que habían este defectos técnicos que no nos permitían su uso entonces muchas plantas han tenido este problema que sus características técnicas no han sido lo suficientemente, digamos las condiciones técnicas no han sido adecuadas para que respondan rápidamente a la producción de oxígeno. Un hospital recibe una planta de y ya esa planta de oxígeno para llegar a una cama de unidades de una cama de cuidados intensivos requiere de una red, esa red, colocar esa red vale entre 100 a 150 dólares por punto entonces, si me unía de cuidados intensivos, tiene 20 puntos, entonces yo necesito por lo menos 2 000 dólares para para este implementar esa red entonces muchas plantas se implementaron para dar oxígeno a través de cilindros y no de red porque además, las redes requieren de una presión y de otras condiciones técnicas que llegue realmente a la presión y a la concentración de oxígen0 por arriba del 93%, algunas tenían red pero la concentración y la fuerza de la planta llegaban 90 80% y eso no era adecuado para el uso de los pacientes entonces pudieron haber problemas técnicos en la planta, en la distribución o en el uso del requerimiento del paciente porque esa esa conexión ya no lo puedo colocar a un ventilador, porque me malogra el ventilador.

**I: Y si hablamos de ese tipo de dificultades técnicas, de cuántas plantas estamos hablando con esas dificultades.**

E: Se necesita hacer un estudio para eso y a través de Socios en Salud se ha hecho una evaluación y estamos esperando el reporte, pero ellos solamente a veces han evaluado 25 plantas entonces es una pequeña, un pequeño de las 490 casi 500 plantas que tenemos.

**I: Ya a ver, pero estas plantas que han tenido estas dificultades técnicas, por llamarlo, están siendo contadas dentro de casi 500 que se tienen.**

E: No, forman parte de las 500, pero me refiero a que se requiere una evaluación especializada para poder asegurar cuál es la capacidad de cada una de estas plantas para cumplir los diferentes procesos y momentos de la distribución de oxígeno ese estudio lo necesitamos un urgentemente

**I: Compleja la situación, por lo que usted me comenta.**

E: Muy complejo, muy pero muy compleja.

I: Pero imagino que es algo por lo que se debe.

E: Pero felizmente que tenemos un proveedor bueno en este caso un aliado, un socio que es Socios en salud, que es una organización no gubernamental trabaja con el Ministerio de Salud en diversos temas hemos logrado insertar este pedido, han venido cooperantes hace un par de semanas hemos tenido reunión de trabajo con ellos y están bien interesados en poder ampliar el estudio.

**I: Ok, para tener una muestra un poco más grande. Entonces el tema es como para ampliar la muestra de las plantas o de los equipos que van a ser revisados se refiere con la ampliación del estudio, la investigación que están. Bueno, eso es con respecto a la parte de los requerimientos del aprovisionamiento, ustedes como dirección han hecho visitas en las regiones para ver, por ejemplo, el**

**tema de seguridad del oxígeno o hay alguna otra área dirección del Ministerio que se encargue de esta labor.**

E: En verdad debería de ser competencia de la Dirección General de Productos Farmacéuticos, considerando que el oxígeno es un medicamento, se han tenido reuniones de coordinación, se diseñó a partir de estas reuniones de coordinación ya saliendo de la pandemia un sistema de información que se llama RENOXI en el cual regularmente se registran el estado situacional de estas plantas y de otros dispositivos y se espera que con otras direcciones como la misma dirección de monitoreo de voz o las direcciones de normas, la dirección general de intercambio prestacional de DEGAIM y otra dirección también el mismo CENARES que de una otra forma es el que compra oxígeno a proveedores externos de deberían de establecer una línea de trabajo para poder asegurar la calidad de la aprovisionamiento en cada ámbito regional están en las también DIRESAS, GERESAS están los directores de medicamentos y en las direcciones de medicamentos hay profesionales que son químicos farmacéuticos que miden la calidad del oxígeno si está al 93% o 95% ellos tienen unos equipitos que les ayuda a hacer estas mediciones pero lo hacen como una actividad regular, lo que se necesita es a sumar a esa actividad regular es la estrategia para poder establecer realmente cómo manejar la demanda saber el volumen de demanda y sobre eso vería la articulación con la oferta que ha quedado post pandemia.

**I: Bueno, usted me dice son los que deberían, pero tiene información usted si se lo han hecho o se están haciéndolo.**

E: Están haciéndolo, están haciéndolo como le digo en las regiones, acá en las mismas DIRIS, sus responsables de medicamentos, pero como una actividad regular no como una que aseguren la garantía del aprovisionamiento de oxígeno.

**I: Y en este sentido, para el tema del mantenimiento, usted me hablaba de una normativa o disposiciones que ya se han establecido. ¿Es así?**

E: Sí, claro tenemos directivas para la formulación de los planes de mantenimiento de plantas de oxígeno que están aprobadas con resolución ministerial. Tenemos la formulación de los planes que han culminado en la asignación de los recursos en los últimos 3 años, desde el 2021 hasta el 2023 para el mantenimiento de las plantas.

I: Ok y ya mirando un poco el tema del compromiso más de las autoridades en el ámbito regional y local usted, como los ve a ellos, están como dispuestos interesados porque ya me hablaba de que el cambio de gobierno, el cambio de autoridades ha generado también algunas no sé no podría decir desinterés, pero sí cambios

E: Cambio de prioridades podríamos usar ese término.

**I: Exacto, es correcto un cambio de prioridades es como ocurre usted diría en la mayoría de regiones, solo algunas usted tiene algún caso muy aislado esto pensando en el futuro básicamente, de lo que puede ocurrir no solo con las plantas sino con todo el aprovisionamiento de los que se tienen.**

E: A ver qué tenemos, qué tenemos para el futuro. Primero, establecer claramente cómo vamos a gestionar el aprovisionamiento de oxígeno a través de 2 niveles de intervención en el ámbito público y en el ámbito privado, en el ámbito público, ya sabemos cuántas plantas hay donde están, cómo están, cuántas operativas, cuántas inoperativas, cuántas operativas apagadas y podemos desarrollar estudios para ver el comportamiento de la demanda en el ámbito público, pero también hay una situación en el

ámbito privado en el ámbito privado la pandemia nos ha dejado como lección aprendida que no pudo responder a la demanda de del gran desastre de la gran magnitud de daño que nos ha hecho la pandemia ósea el sector privado pre pandemia nos abastecía con el 90% del mercado pero vino la pandemia y no tuvo capacidad de respuesta entonces está latente que si seguimos en la misma condición dependiendo del sector privado, una próxima pandemia mundial o acá en el mismo país, ya lo estamos viviendo con Dengue los pacientes graves de dengue terminan en una unidad de cuidados intensivas, los pacientes graves de Gillian barre terminan en una unidad de cuidados intensivos y terminan requiriendo oxígeno, entre otros bienes. Entonces las pandemias que vamos a sufrir y que estamos sufriendo van a requerir de una política de una y de una acción pública bien definida, en la cual tenga que participar el sector público y el sector privado osea la solución no viene solo hallo este con unas 497 plantas yo enfrento a la pandemia, no lo vamos a poder hacer por la complejidad de las condiciones tecnológicas, por la complejidad de nuestra demanda, de nuestra geografía, de acceso, etcétera; el problema se traduce en una en una condición multidimensional entonces, ¿qué hacemos con lo público primero? Primero hay que tener los estudios de dimensionamiento de la demanda y de la oferta, y eso es un tema pendiente, no hemos dimensionado claramente la demanda y la oferta que en este momento se presenta como una sobreoferta no la tenemos del todo controlada, porque para empezar existen diferentes actores con diferentes niveles de responsabilidad hay un actor de tipo institucional Essalud tiene sus plantas, MINSA tiene sus plantas, los gobiernos regionales tienen sus plantas esa es una dimensión institucional, pero también hay otra dimensión de nivel de gobierno, MINSA tiene una responsabilidad con los gobiernos regionales, los gobiernos regionales tienen una responsabilidad con las municipalidades que han comprado plantas de oxígeno, y esa es una dimensión gubernamental y así podríamos ir desarrollando diferentes formas de análisis pero que tienen que converger hacia una solución pública, entonces mi opinión ya muy digamos muy de más de orden profesional técnico es que necesitamos cuantificar la realidad y las variables primero necesitamos cuantificar cuántas plantas de las que están operativas, sí cuánto es el costo de producción o sea cuánto cuesta cuánto le cuesta al estado peruano un metro cúbico de oxígeno y si al Estado peruano le cuesta 5 soles el metro cúbico de oxígeno y lo estamos comprando a 2 soles en el sector privado, entonces las plantas de oxígeno, habría que ver qué otra utilidad.

Entonces le decía que el punto de quiebre de la solución multifactorial es tener un estudio de costos, porque el estudio de costos nos va a permitir abordar la dimensión pública y la dimensión privada y qué hacemos con el estudio primero quién lo haría en el caso del Ministerio de Salud ya hemos hecho un convenio con una organización PATH los señores de PATH son una organización que tienen una sede en Washington y hemos hecho un convenio para hacerlo un estudio de costos, hemos tenido ya reuniones de trabajo, hemos diseñado las variables, los formularios y en este momento estamos en una fase de campo, claro que ellos nos han dicho yo te puedo ayudar hasta creo, 20 plantas y después de las 20 tú te coges la metodología y después ya lo puedes ampliar por algo habría que empezar y qué esperamos de este estudio de costos este estudio de costo nos va a decir el metro cúbico de la producción de oxígeno en una planta de tales características, de tales condiciones, con tal horario de funcionamiento, con tanto de personal, etcétera, va a ser le ponía el ejemplo 5 soles entonces ahí ya podemos decir ok, si está 5 soles y el sector privado me lo vende a 3 o a 2 entonces me conviene mejor irme por el sector privado, pero si el sector privado me lo está vendiendo a 10 soles entonces uy un momentito recupero las plantas, invierto en las plantas, pongo más personal establezco redes de distribución complementos los temas técnicos de redes de la llegada de la oxígeno, aseguró la calidad, la concentración, etcétera. Entonces, pero si ya sé que el sector privado me está cobrando por arriba y está llegando en el ejemplo que les decía a 10 soles el metro cúbico, pero en tanto no tengas información no puedo tomar decisiones claras y correctas.

**I: Ok, Y una vez que tengan esa información o en función a esa información, usted considera que habría que hacer algún cambio en la política de oxígeno medicinal a nivel normativo.**

E: Si por supuesto, de hecho, que esa información y ese estudio es clave para definir una política para establecer claramente una estrategia que va a ayudar no solamente al Ministerio de Salud, sino también a los gobiernos regionales a Essalud y al resto de las instituciones públicas.

**I: Hay una postura imagino también de parte de las empresas privadas al respecto. ¿Cómo ve usted el interés o la participación de la empresa en este tema?**

E: De hecho, que, al sector privado lo que le mueve es el interés de tener una rentabilidad económica y eso no debería de estar mal es lo esperado en una sociedad que o como la nuestra establece dentro de 1 de sus medios de desarrollo el manejo del mercado, y ahí lo que necesitamos es que desde nuestro lado, como sector público establezcamos un mecanismo de conversación primero y de hacer que el sector privado entienda y visualice con claridad que durante la pandemia no nos ayudó, no nos ayudó.

**I: En qué sentido no les ayudó.**

E: No nos ayudó en el sentido de que la demanda generada por Covid-19 sobrepasó sus capacidades, que no sabemos si por capacidad instalada por falta de capacidad instalada porque ellos también producen oxígeno para la industria para otros sectores entonces eso es parte de lo que ellos van a tener que evaluar y de acuerdo a cómo ellos evalúen, podrán decir, no llegue a esta capacidad porque éste no tenía canales de distribución o mis 2 plantas mis 3 plantas que tengo a nivel nacional no fueron suficientes. Entonces, ¿cómo nos preparamos para el futuro para otra pandemia? eso es lo que tenemos que hacer en el trabajo con ellos.

**I: Y a usted Ha percibido que hay un interés de las empresas privadas por hacer este tipo de estudios por saber o conocer o prepararse para el futuro, porque podría quedarse o centrarse en la cuestión**

E: De las veces que hemos podido en un momento de la pandemia ellos integraban formaban parte de un grupo de trabajo liderado por la Presidencia del Consejo de ministros y participaban en las reuniones diferentes proveedores inclusive y ellos participaron de hecho que es cuestión que se le convoque y en función de eso medir su nivel de predisposición para enfrentar el problema futuro que tenemos.

**I: Ok, ya como para ir cerrando algunos temas me gustaría saber así en general o consolidado ya lo que usted ha mencionado, cuáles fueron los retos más importantes si puede señalar 2 o 3 con respecto del oxígeno medicinal durante la pandemia a partir del trabajo de la gestión del área en que usted estuvo laborando.**

E: A ver, de hecho que en el área de infraestructura y equipamiento del Ministerio de Salud se dieron en diferentes momentos retos muy críticos acuérdense que primero apareció el problema de las camas UCI, no sabíamos cuántas camas y dónde estaban estas camas UCI entre comillas y por qué decimos que no sabíamos donde o cuántas camas y por qué lo pongo entre comillas, porque en verdad uno va a un hospital y la única forma de saber cuántas camas hay en ese momento haciendo un corte transversal y decir este es mi número de camas por qué de un día para otro el número de camas varía los que trabajamos en los servicios, los que conocemos el funcionamiento de nuestros hospitales sabemos de qué la forma de saber es a través de un censo y ya tenemos inclusive enfermería tiene su procedimiento a las 12 de la noche hace su corte, claro eso en una emergencia, no era posible y lo que teníamos eran fuentes entre comillas que nos alimentaban a las 8 de la mañana a las 2 de la tarde y a las 8 de la noche

para decirnos cuántas camas habían disponibles cuantas ocupadas y en función de eso el sistema arrojaba un determinado volumen y número de camas de cuidados intensivos entonces ese ha sido un Hito que se trabajó a través de un sistema de información con Susalud y con la Dirección General de Operaciones monitoreábamos esa disponibilidad de camas UCI pero pasó la premura eso se respondió a través de una modalidad que le llamamos y hospitales de ambientes temporales los HT y sacó una norma se implementaron más de 3000 camas mucho más mas de 30 mil camas tenía una duda del decimal , camas de hospitalización y alrededor de 2 500 camas UCI, entonces las camas fueron un hito se defendió una estrategia, se dio una respuesta y recordará que después de las camas y las camas  están asociadas a oxígeno, no o sea hay cámaras de hospitalización que requerían de oxígeno hay camas UCI que una cama de hospitalización requiere de 14 metros cúbicos por día, las camas UCI requieren de 60 m cúbicos día entonces son 4 o 5 veces más que una cama de hospitalización. Y después del problema de las camas apareció el problema de los ventiladores, no había ventiladores a comprar ventiladores, a definir especificaciones técnicas el Ministerio de Salud tuvo que aliarse con algunos operadores logísticos de reconocida trayectoria en el país como LEGADOS, como ARCC para aprovisionarse de alguno de estos equipos y después de los ventiladores vino el tema de los cilindros de oxígeno y nuestra población que formaba colas buscando un cilindro de oxígeno eso fue una cosa muy terrible.

Esta Dirección General de Operaciones y la misma Dirección de Infraestructura y equipamiento, como que eran el núcleo central de una serie de medidas y respuestas ante esta emergencia que se pasó entonces eso ha sido un enorme reto, pero de hecho que las cifras no son buenas ósea hemos tenido una mortalidad muy alta y hemos sufrido mucho los efectos de la misma complejidad y de lo malo que han pasado nuestras familias y nuestros pacientes.

**I: Ok, y ¿Cómo diría usted que está la situación del oxígeno medicinal hoy día que se ha aprendido, que ha cambiado?**

E: Hoy día, lo resaltante es que tenemos una sobreoferta de oxígeno ósea hay una enorme capacidad para poder responder a un evento muy imprevisto ya sea por el lado de dengue por el lado de Gillen Barre no nos va a faltar oxígeno para poder atender esas 2 entidades no vamos a tener déficit, no deberíamos de esperar alguna situación este inadecuada, inapropiada para poder abastecer nuestros servicios de oxígeno eso lo tenemos bajo control en relación a oxígeno pero si nos tenemos que continuar trabajando para poder aprovechar esto que nos ha dejado  en lo positivo y en lo negativo, en lo negativo la necesidad de definir esta política  que concluya si es por el lado público lo que tenemos que fortalecer o es seguir dependiendo del lado privado.

**I: Por lo que me dice en este momento por el tema del dengue y Guillen Barre estamos cubiertos, pero ¿qué pasaría si de pronto tenemos una pandemia con la magnitud que tuvimos por el por el COVID-19? ¿Cuál sería la situación? ¿Qué pasaría? ¿Estaríamos en condiciones de afrontar una situación similar?**

E: A ver este por el lado de volúmenes de toneladas de oxígeno esta brecha de los 374, de las 374 plantas que están entre MINSA y gobiernos regionales que representan casi el 75% del volumen total de plantas si ayudaría a enfrentar una pandemia de este nivel, pero lo que ocurriría es llegar a ese nivel. A ese volumen a una relación en la cual la demanda se equilibre con la oferta, pero debemos de construir esa esa relación o sea debemos de asegurar los diferentes canales para poder decir ah si se presentara a través de una demanda de 500 toneladas por día de oxígeno yo sí la voy a suplir dando 200 toneladas con plantas de oxígeno, 100 toneladas con concentradores y estas 200 toneladas con tanques criogénicos. Ese análisis necesitamos hacer como parte de una propuesta de política.

**I: Pero nuevamente, si en este momento tuviéramos una pandemia con las características similares a las que tuvimos, podríamos afrontarla.**

E: En materia de oxígeno en este momento sí.

**I: Si en materia de oxígeno, básicamente. Ok, finalmente, me gustaría que como para ir resumiendo todo toda la conversación que hemos tenido o si tiene algún aspecto que no hemos mencionado como una lección aprendida o una lección clave sobre el tema si pudiera mencionar 2 o 3 respecto de este tema tan complejo que es el tema del oxígeno medicinal.**

E: A ver, primero que requerimos un marco normativo que esté debidamente consensuado por todos los sectores y por todos los actores, ahora el Ministerio de Salud tiene una norma, pero hay que ver si esa norma realmente está cumpliendo y está ayudando a que se concretice en asegurar un mejor acceso a los servicios y a los pacientes en condiciones de oxígeno ese es un reto que tenemos.

**I: Sí, porque no solamente son plantas también concentradoras. ¿Están bien estos equipos portátiles que hay personas que han ido adquiriendo, que personas que han tenido COVID y quedaron afectadas requieren un flujo constante oxígeno y que no hay regulación para la importación o la compra fuera?**

E: Correcto y eso nos lleva al otro extremo que es el ¿Para qué necesito el oxígeno? estando en una situación actual, la situación actual nos dice que hay una sobreoferta pero también que ya la pandemia bajó pero existen otras fuentes de uso de oxígeno en nuestros pacientes, por ejemplo, pacientes asmáticos, pacientes con fibrosis pulmonar, pacientes de la tercera edad que tienen algún tipo de requerimiento de oxígeno con condiciones de ubicación en este usos en altura, por acceso geográfico que tienen otras condiciones otras particularidades tecnológicas y que requieren de gente de oxígeno; entonces ese volumen debe utilizarse para poder decir esta brecha si es factible darle una un uso racional, un uso más coherente, más de beneficio para ciertos segmentos de población que van a necesitar infección respiratorias que va a ser un denominador común en los tiempos siguientes van a requerir de que se utilicen diversos dispositivos de oxígeno, entonces esa es una segunda línea de este de trabajo de poder enfrentar en esta situación actual.

**I: Ok, yo no tengo como más preguntas en este momento, pero no sé si usted quiere hacer algún comentario, tal vez sobre algún tema que no he tocado o algo que digamos no ha podido usted explayarse de la manera que hubiera querido.**

E: El tema de las plantas de oxígeno también tienen algunas acciones de control que hay que visualizar en el futuro posterior, y eso es algo evidente que se tiene que dar lo que hay que este mencionar es que en el caso del Ministerio de salud tuvimos la observación de las plantas de oxígeno que le encargamos a la Universidad de ingeniería. Inicialmente le habíamos encargado la implementación de 47 plantas de oxígeno pero que no se pudo concretar y después se entró a una conciliación se quedó en el compromiso de esta conciliación que nos implementaran 22 plantas de oxígeno y a la fecha todavía no han logrado cumplir entonces tenemos ahí 22 plantas de oxígeno con unos procesos de control que ya están inclusive a nivel judicial o de arbitraje, entonces esos son temas que también van a afectar significativamente el enfoque que se le dio durante la pandemia porque están inmersos en hasta en situaciones de manejo político eso digamos como para que, como un aspecto que es importante mencionarlo en el análisis que se va, que se está realizando, porque puede afectar y va a afectar o va a requerir que se ponga en la mesa con más claridad algunos otros aspectos y no solamente con plantas de oxígeno por ejemplo, en cilindros, usted debe haberse percatado que hace unos 3 meses un poco más de repente salió una noticia

periodística donde habían 10 000 cilindros en un almacén de LEGADOS entonces ahí detrás de esa información hay toda una historia que me imagino que en algún momento no solamente va a requerir de la evaluación de los órganos de control y de las instituciones competentes si no habría que tomarlo como un punto este de actualidad ya que hay que decidir que se va hacer con esos 10 000 cilindros en este momento están en ese almacén. ¿Qué es lo que debería recomendarse en función a los criterios técnicos y en función a los criterios normativos y en función a la necesidad real del uso de estos bienes? esos aspectos son temas que están pendientes de un análisis que ya vienen haciéndolo los órganos de control, pero que nuestra población en algún momento va a necesitar conocer el fondo de lo que ha ocurrido.

**I: Usted, me comenta que hay la necesidad de establecer mayor control en los aspectos técnicos únicamente o con el proceso, por ejemplo, de compra de adquisición, la instalación propiamente dicha en fin, en que o es en todo que está la necesidad.**

E. En este momento, habiendo una sobreoferta de plantas de oxígeno, no deberíamos digamos aprobar la implementación de otra planta sin una evaluación previa, ósea se puede construir un hospital en Piura, hay que analizar si el hospital de alta complejidad de Piura va a requerir de una planta de oxígeno, pero yo no podría decir ponlo en tu expediente técnico que lo construyan y compren la planta y después ir a decir a estuvo bien o estuvo mal, que es lo que hace contraloría, desde antes ya tenemos que decidir si estos nuevos hospitales van a requerir de una planta de oxígeno y ese tema es un pendiente enorme.

**I: Pero, ¿y ese pendiente tendría o se está procurando, resolver de manera inmediata o no se está como Previendo en futuro?**

E: En este momento todavía no se ha puesto en agenda.

**I: Vale decir que, si mañana alguien decide poner una planta, instalar una planta en algún establecimiento salud, lo podría ser sin mayor análisis.**

**E: Sí, porque no hay una norma que se lo impida, no hay un acuerdo político que se le impida.**

**I: Bueno, muchas gracias ahora si no tengo más preguntas y si surgen espero que usted esté en la disponibilidad de que tal vez podamos contactar más adelante y lo que sí es que, por favor, si nos pudiera enviar esta estos datos que usted había comentado acerca de las plantas, los lugares, la distribución en la región.**

E: Y si se lo pasó, ahorita voy a entrar a una reunión, pero en la tardecita se lo estoy alcanzando.

**I: Muchísimas gracias y en un momento también le voy a enviar el consentimiento el documento del consentimiento informado correspondiente a esta entrevista**

E: De acuerdo

# 012 Entrevista

Meeting started: 21/7/2023, 3 PM

Participantes: E, I

I: Investigador

E: Entrevistado

**I: buenas tardes, mi nombre es LizzeteNajarro hoy es 21 de Julio del 2023. Esta es una entrevista como parte de la estudio economía política del sistema de oxígeno medicinal en el Perú y en este momento vamos a conversar con una doctora, una médica que trabaja en gestión pública en una institución en Iquitos. Doctora para comenzar por favor si nos pudiera comentar un poquito acerca, solo para una cuestión de registro diría yo, nos podría contar un poquito acerca de cuál es su labor, en que… cuál es el puesto que tiene en este momento en relación al tema del oxígeno medicinal y si esa labor o esa tarea ha cambiado en los últimos años**

**E:** Buenas tardes (Ininteligible, MINUTO 01:15) de salud pública , en la época del covid yo estuve como médico asistencial este…en los centros de atención primaria de pacientes con covid en las 3 olas que tuvimos. Actualmente(Ininteligible, MINUTO 01:38) ocupo un cargo funcional, soy directora XXXXXX de Lima pero de nivel regional. Bueno también tiene este… una gran parte en cuanto al monitoreo de las enfermedades, como son en este caso del covid, y bueno estoy siguiendo de cerca otro tipo de… brotes epidemiológicos que hay en la región. Eso es todo

**I:OK. Doctora por favor si nos pudiese repetir la primera parte, se cortó el audio y no logré escucharla. Sólo el primer minuto.**

**E:** Mi nombre es BCH, soy médico con maestría en salud pública. En la época del covid estuve como médico asistencial de los centros de atención temporal para covid en las 3 olas.

**I: OK de ahí en adelante ya la pude escuchar. Muchas gracias. Bien, doctora para ir comenzando entonces usted podría comentarme cómo es la parte normativa institucional a nivel regional que ha tenido que ver con el tema de la emergencia sanitaria y también con el tema del oxígeno medicinal de manera específica en Iquitos o en la región.**

**E :** En la… una pregunta ¿la entrevista es de los datos retrospectivos?¿Esa pregunta es retrospectiva o es actual?

**I: sí,sí y luego usted me comentarás si eso cambió en el tiempo ¿No? Cómo ha sido la situación durante la pandemia, sabemos por información que se difundió a nivel nacional que hubieron muchísimas dificultades pero me gustaría comprender a partir de lo que usted pudo ver, a partir de su experiencia como fue el tema. Si pudiéramos tal vez empezar comentando¿qué pasó?¿cómo fue? Para tener un registro a partir de su experiencia.**

**E:** Bueno nosotros como loreto fuimos la primera región en ser afectados con covid ¿no? A nivel de Perú… por ser de provincia, la atención este…llegó tarde, tal vez no nos tomaron en cuenta el inicio porque como es una ciudad que tiene costumbres, los pobladores tienen costumbres muy este… liberales, no sé qué adjetivo más usar, son muy amigables¿no?, que salen a reuniones sociales y eso; para ellos fue fácil, para el virus fue fácil la

diseminación. Entonces como era una enfermedad nueva, creo que este… ha habido varios comportamientos, un grupo se han aislado en sus hogares y han tratado de usar medicina casera, y otro grupo han tratado de acudir a los centros de salud. Este…y lo malo fue que casi los centros de salud no estaban muy bien equipados y se empezó a trabajar la parte de asistencia… asistencia temporal, o sea han abierto colegios ,lugares donde atendían a los pacientes¿no? Se atendían ciento y tantos, 200 ,300 diario y se les daba una atención primaria. Pero más llegaban ahí por la falta de oxígeno, porque no había oxígeno disponible para la población … tenemos 2 hospitales acá en Iquitos y solamente el hospital regional producía ponte, estoy diciendo cualquier número, 10 balones diario por ejemplo de oxígeno¿no? Que servía para casos puntuales de enfermedades respiratorias y que no había tanta demanda al mismo tiempo¿no? Entonces de esos 10 balones tenían que empezar a producir 300 por decir, o sea de frente fue un número fuerte. Y este…eso fue primero hasta que se llene el hospital, entonces sacando nuestros cálculos nos dábamos cuenta de que nosotros mismos nos proyectábamos, porque yo en ese momento estaba en el primer nivel, no estaba en la parte decisoria ni de análisis si no estaba en el primer nivel nos dimos cuenta que el oxígeno y vamos a carecer de oxígeno, o sea iba a llegar un momento en el que iba a faltar y no íbamos a tener, todos empezábamos a pedir

**I: Más o menos cuando es que se dieron cuenta, se percataron de que eso iba a ocurrir**

**E:** Ya, eso por ejemplo al mes más o menos de que empezó la pandemia o sea como en el grupo estábamos personas que En algún momento tenían una actividad pues este importante como médicos; me acuerdo que entre médicos el tratamiento era mantenerlo vivo al paciente hasta que se recupere¿no? o Hasta darle chance que alguien descubra qué es lo que tiene más efectividad¿no? Porque estábamos con los retrovirales, o sea le damos todo lo que alguien se le ocurría pero no era porque había unas, estaba demostrado¿no? entonces lo que teníamos que ganar es tiempo para que el paciente dure ¿no? Y nos comentaba, tenía varios amigos que... Uno que trabajaba en Essalud porque yo estaba trabajando como minsa y me decía pues de que en el Essalud de acá estaba bastante copado, que la gente hacía cola y sólo había un balón en emergencia Imagínese ah, un solo balón. Entonces tenía… o dos, pero la cosa es que tenía conexión para dos pacientes, entonces dice que él en su desesperación o sea venía deprimido¿no? Yo le he dicho '¿como te ha?' ido le digo Y me decía que se sentía mal porque él tenía que decidir a quién le daba el balón este… la mascarilla con oxígeno¿no? Dice que ya estaba bien el paciente X y venía uno azul, tosiendo entonces le decía ya le quitaba y le daba al que estaba viniendo para que se tranquilice y deje de toser ,y recuerdo que me dice que el que ya estaba tranquilo a los minutos otra vez empieza a toser y a ponerse azul y eso, entonces no sabía qué hacer, se desesperaba¿no? Eran tantos que no se nos va a borrar a nosotros, solo por falta de oxígeno ni siquiera otra cosa ni siquiera otra cosa.

**I: Doctora, y antes de la pandemia ¿ustedes en algún momento habían tenido la necesidad de contar con oxígeno, tenían ya provisión de balones o de un sistema de oxígeno de provisión de oxígeno en el hospital?**

**E:** Este, como le decía tenemos una cantidad x no no supiera decirlo de los hospitales, pero que era todo nuestro hospital que alberga 250,350 camas llegaron a ser 500 o más porque utilizábamos todos los pasadizos y todo, todos necesitaban un balón de oxígeno. El detalle es de que de acuerdo a la intensidad, sacando cálculos, cuando no era muy intenso la necesidad de oxígeno necesitaban 3 balones por día entonces eso multiplicale por número de personas y el requerimiento diario sobrepasaba la… la estación de oxígeno que tenían en el hospital regional. Entonces ahí es donde ellos este hecen en el contrato, y nosotros calculando que eso solamente en el hospital regional, calculando que con la …teníamos 3 centros este ambulatorios ¿no? Donde la gente iba de los centros de salud a pedir ayuda, entonces este sacando pluma nos dimos cuenta de que no iba a alcanzar para loreto, para Iquitos porque había sólo 2 o 3 empresas que daban oxígeno y que justo un proveedor nos dice que él, le preguntamos cuántos balones de oxígeno llenan al día ¿no? Producen al día y nos dio un número y sacando cuentas nos dimos con la sorpresa de que no íbamos a llegar, nos dio tanta pena. Entonces ahí ,yo me acuerdo que le comento a mi amigo que él estaba en el colegio médico y le dije pues 'nos vamos a quedar sin oxígeno en algún momento, la gente se va a empezar a morir por falta de oxígeno no va a ser por falta de medicina' aunque bueno el oxígeno es un insumo, una medicina. Entonces este... Ahí es donde varias personas empiezan a comprar(Ininteligible, MINUTO 12:28)

**I: Por favor…**

**E:** Sí dígame

**I: Sí le perdí por un poquito. 'Ahí es donde' hasta ahí le seguí**

**E:** Aya, ahí es donde empezamos a hacer compras este de oxígeno, de balones de oxígeno a Lima ya y los casos eran así teníamos pocos casos todavía pocos; era manejable todavía teníamos tiempo de conversar, de almorzar, de ir al baño, bueno de todo. Y ahí también es donde el padre Raymond se da cuenta que necesitábamos oxígeno y él empieza a hacer sus gestiones para buscar como podría ser para conseguir, qué tan difícil es tener una planta de oxígeno porque él también atendía ahí en Canatari que es una casa de retiro, él iba y atendía entonces este…me acuerdo que otro mes más, en un mes más ya estaba todo colapsado teníamos 300, 400 pacientes en un colegio, un colegio emblemático los 3 pisos estaban llenos de pacientes, faltaba a manos para poner el medicamento y todo, igual que en el hospital regional ¿no? Algunos también estaban… había un grupo que se salvó sólo teniendo oxígeno pero sin medicamentos porque las enfermeras no se abastecían y otros que no, no habían recibido … habian recibido otro tipo de medicinas y llegaban ya en mal estado de salud. Y bueno el hospital efectivamente no se abastecía porque sólo producía…no me acuerdo cuántos balones al día pero solo alcanzaba para un piso no más, no para todo.

**I: Ahora, doctora el hospital producía, usted me dice, ¿tenía una planta o es que compraban tenían un proveedor de oxígeno privado?**

**E:** Este, no solamente es la planta tiene que tener también su mecánico, el que le opere su operador así etcétera etcétera. Ellos ya tenían un contrato con una empresa que les proveía oxígeno, pero era les daban por ejemplo una vez al mes le llenaban, algo así. Los que más utilizamos oxígeno eran los pacientes hospitalizados en uci, era en sala de partos y en sala de urgencia; y eso se tenía un consumo promedio estándar que no no pasaba y de ahí era las épocas que ese aumentaban era cuando había enfermedades respiratorias por el friaje, no sé no tengo el dato de cuánto era el consumo promedio la cosa que eso era solamente un 20% de lo que realmente llegamos a necesitar después. Y ahí es donde se empresa contratar otras empresas.

**I: OK. Sólo para tener la precisión, el hospital siempre tenía un proveedor no es que tenía una planta generadora previa al covid.**

**E:** Los 2 tenía, tenía una planta pero que era insuficiente

**I: Ya ya ya, OK. Y a la vez tenía su proveedor digamos…**

**E:** Y aparte tenía un proveedor porque de todas maneras la demanda era un poquito más y este, también el otro dato que nosotros sacamos ,preguntamos cuánto tiempo se demora en llenar un balón de oxígeno ¿Ya? Poniéndole a trabajar a la planta a las 24 horas del día creo que sólo producía 10 o 15 balones¿no? Cómo es ese tiempo no me acuerdo mucho

**I: Era una planta pequeña entonces**

**E:** Era una planta pequeña que no producía muchos balones por eso es que tenía un proveedor aparte, y lo otro era que como toda planta necesita mantenimiento y siempre tenían el contrato con un proveedor a veces le pedían ponte 5 y otras veces 15 balones se me ocurren, él suplía eso hasta que la planta sea puesta operativa

**I: Se le haga el mantenimiento**

**E:** Igual esa planta proveedora en un momento nos decía que ya había cumplido no sé cuántas horas de trabajo y tenía que entrar mantenimiento o si no iba a colapsar y la gente se desesperaba porque se iba a quedar un día sin producir oxígeno, todos entraban en pánico ahí, ya sabían ya que si no estaban ahí por favor rogaban que estén dentro para que no se queden al aire, la gente hacía cola todo el día, era como en la época de Alan García que hacían cola por arroz pero hacían cola por oxígeno en esta oportunidad

**I: Ok. Y una vez que el el tema de la falta de oxígeno ya es una crisis evidente, entiendo que entra a tallar las plantas de oxígeno que se consiguieron a través de donaciones¿no? Porque supimos a través de las noticias, de información en medios de comunicación que se hicieron grandes campañas para recolectar donaciones y comprar plantas de oxígeno dirigidas para Iquitos¿cómo fue este tema allá?**

**E:** Bueno me puedo equivocar, que yo sepa este… no mandaron, me puedo equivocar al decir, que yo sepa el minsa no mandó una planta adicional, no sé tendría que hablar usted con algún director de esa época para que le cuente ¿no? No sé. Lo que ayudó fue la parte privada… porque Essalud por ejemplo tenía su propia planta creo que ellos sí trajeron uno adicional pero no me acuerdo.Pero se demoraron ,Essalud Fue lento más lento que el minsa pero sí llegaron a cubrir la necesidad. Ahora este… un candidato compró, un candidato para el gobierno regional, compró una planta de oxígeno y él puso de ahí este… bueno el padre Raymond consiguió otro con las donaciones que hizo. Pero la parte del minsa no nos hacían caso esa era la realidad, en la primera este…

**I: Ola**

**E:** Ola, Lo que eso sí hicieron fue mandar balones de oxígeno mandaron 100, 200 pero no mandaron una planta, no mandaron eso sí me acuerdo

**I: Y ustedes…**

**E:** Entonces nosotros incluso mandamos a traer este…balones de yurimaguas, de tarapoto así, En deslizador. (Ininteligible, MINUTO 20:28) Si no hubiera sido por la parte privada no hubiéramos tenido esa soltura , esa independencia de conseguir hubieran muerto más personas

**I: Doctora, ustedes como hospital a quienes le solicitaban la compra de oxígeno o de la planta de oxígeno. Dependían de quién ¿de la DIRESA?**

**E:** Sí mira yo estaba trabajando como primer nivel ,no se olvide. Estaba trabajando en un centro de atención primaria, un CAT, dependíamos de GERESA. GERESA había pagado un proveedor para que nos dé oxígeno porque lo que el hospital producía era para ellos pues, antes nos daba, nos podía dar unos cupos pero la cola era inmensa.

**I:OK**

**E:** Y a veces no teníamos, llegabamos un momento en decir que teníamos atención pero no tenemos oxígeno igual que en el seguro cuando yo pedía referencia de un paciente, porque era atención de un primer nivel como una IPRESS I-3 nomás , y me decía yo le decía 'doctor el paciente tiene Essalud' porque nosotros atendíamos de todo, incluso a gente que que tenía seguro privado entonces por favor para que lo atienda 'ya Bersy' me decía 'te acepto la referencia pero que venga con su balón de oxígeno porque acá no hay oxígeno' así nos decían

**I:Ok**

**E:** Y algunos no querían ir porque había mucho más burocracia allá

**I: Ajá, luego en la segunda ola ¿qué ocurrió?**

**E:** En la segunda ola ya teníamos el, ahí ya ya no había problema de oxígeno a mi parecer. De todas maneras era un poco más lento pero no había ese desborde de pacientes que teníamos 300 ,400 pacientes, no no había ese problema. En la segunda ya se pudo actuar de una forma más, ya había el… 2 plantas de oxígeno, del regional y del padre raymond y el padre raymond pidió otra una segunda más, o sea 2. Porque qué pasaba, él se dio cuenta de que no tenía él hizo un convenio con GERESA creo con el hospital para que… no solamente pues es comprar un equipo ¿no?, tienes que darle mantenimiento, la seguridad, tienes que tener quién va a el operador que va a ser y todo eso . Y el vicariato no tiene presupuesto¿no? Para mantener, dar mantenimiento ¿quién le va a pagar a las personas que ejecutan, que manipulan la máquina?¿no? Por eso es que él entrega al este... a la GERESA y este la GERESA coloca la planta de oxígeno dentro del hospital regional porque tampoco no tenía otro lugar como para decir ya pues ahí¿no? Entonces eso le daba ya la responsabilidad al hospital regional para que su personal de mantenimiento u el operativo pueda manipular, cuidar, resguardar …la máquina. Entonces ahí es donde Raymond se da cuenta de que todas maneras no tenía la libertad de pedir porque él atendía a sus pacientes también en la iglesia porque él es médico, y ponte quería 5 balones no podía así que hace otra campaña y pide otra donación para que él ya le instale y pueda tener independencia de poder pedir...

**I: Para los pacientes que él veía….¿hola?**

**E:** …Como no había para todos, siempre pues hay una… la intervención tal vez no es la más justa¿no?, no sé cómo clasificar. Entonces él este… venía pues primero todos los que están más graves, todos los que están en uci. Un paciente consumía hasta 10 galones por día imagínese entonces teníamos 11, 12 pacientes en uci pues ¿no? Pero al final cumplía con uci, como voy a dar que ellos querían cumplir primero con uci, de ahí con intermedios, de ahí hospitalizados y los que estaban en casa pues estaban en la cola de la cola¿no? Cola, cola,cola, ese era el detalle, y la cola para hacer oxígeno para pedir demoraba días ahí está , lloraba la gente desesperada. Y eso que el mismo del hospital, o sea yo por ejemplo tenía familiar yo tenía un familiar ahí y teníamos conocidos que también estaban sus familiares ahí , y un familiar estaba haciendo cola pese a que su papá estaba en intermedio, imagínate. O sea no le garantizaba porque lo que antes había imagínate, había un solo mecánico para instalar la bomba en el balón de oxígeno, o sea las cánulas esas cosas de ahí que usas con la llave maestra. Y nosotros tuvimos que aprender, es tanto tanto es así que yo aprendí porque el mecánico, uno de los mecánicos se enfermó con covid y él ni siquiera tenía fuerza para ser él mismo, y. él nos indicaba 'hagan así, así, no ponle así' no sabíamos cómo porque ahí tuvimos que aprender pues a hacer cargadores, a ser mecánicos, todo. Ahí es donde él nos enseña, cuando él se empieza a mejorar me acuerdo, ya cuando se sienta por qué no se podía ni sentar, no se podían ni o sea estaba recostado ¿no? bien echado porque le faltaba el aire y cuando ya se empieza a mejorar empezaba a ayudarles a todos los pacientitos que estaban ahí en nuestra sala de atención del Canatari me acuerdo¿no? Veía que nos desesperábamos y eso o que ya se estaba acabando un oxígeno y no sabíamos a quién quitarle su reserva y cómo teníamos la garantía de que iba a venía y me decía 'solo présteme un ratito, mi papá, mi hermana está en la cola le falta a 10 personas nomás para que llegue ahí le vamos a devolver' y no sabíamos qué hacer y si no llegaba ya se iba a acabar, pucha de verdad era bien…bien tedioso. En la segunda ola ya tuvimos los concentradores de oxígeno

**I: Ya**

**E:** Pero acá lo malo era de que como nosotros estábamos, nos llegó como 10 así un montón de concentradores pero los concentradores no daban la potencia de oxígeno igual como los otros¿no? No sé si era la calidad o potencia pero la saturación no era… tan buena, no era tan buena como debería como era con el otro oxígeno. No llegaba a su saturación a los niveles que deseábamos y también era la presión que ejercía.

**I: ¿Presión en qué sentido?**

**E:** Por ejemplo, al principio en la primera ola sólo usaban, usaban 3 vías para el oxígeno una era cánula binasal que es por la nariz, el otro era por la nariz y boca una máscara que solo llegaba hasta 5 L, con la cánula llega hasta bien hasta los 3 L, o sea de acuerdo a las necesidad le dábamos, y la otra era con bolsa de reservorio, que le daba este como un recambio para que pueda aspirar y llegaba hasta 12 L que era lo ideal hasta 12 podría llegar hasta 15 pero se perdía. Entonces este la… los concentradores de oxígeno tenían solamente esos modelo que nos llegaron que compró el MINSA sólo llegaban hasta 10 L nomás y no era… y a veces le poníamos 10 y su saturación no llegaba al mínimo que nosotros necesitábamos, entonces le cambiábamos(Ininteligible, MINUTO 30:07) usábamos el balón pues y con eso sí este…tenía más fuerza y ahí sí se notaba, le ponía 10 L y su saturación estaba en 99 por decir pero yo le ponía la máquina 10 L, 97, 96. Entonces de todas maneras había un pequeño margen de diferencia qué habrá sido será la marca, que se yo no le podría decir. Entonces eso era uno pero eso era en pacientes que estaban más comprometidos, en pacientes leves no era, no era tan notorio¿no? Y lo otro era que Canatari era un centro de, era un centro de aislamiento inicial para que no se contagien con la familia, luego se hizo un centro de atención primaria ahí ya se atendía. Y…Imagínese colocar 20 concentradores porque queríamos recepcionar más y este (Ininteligible, MINUTO 31:21) no estaba en la capacidad para

**I: No le escuché, le perdí hasta que querían instalar 20 concentradores en Canatari ¿qué decía luego?**

**E:** Y cuando aprendíamos 10 colapsaba la luz, la intensidad tenía que tener otro y de ahí lo otro era que la luz a veces se iba y ahí nos desesperábamos.

**I: Entonces por lo que usted me cuenta, sí dígame**

**E:** O sea los 2 lados tenía sus desventajas ¿no? Uno más que otro

**I: Por lo que usted me dice han tenido estos concentradores sabe usted ¿en qué cantidad llegaron a través del minsa?**

**E:** No, no, no sé pero ahí solamente en Canatari llegaron como 20 pero ellos repartieron a todos los establecimientos I-4, incluso a los I-1, a los I-2,I-3. El detalle es que no se percataron, usted sabe que el minsa puede tener buena intención¿no? Aparte que cuando haces tu número, sacas tus números dices pues ya '¿cuántos establecimientos hay? Bueno loreto tiene 300, 450 establecimientos ya le multiplico por 2 concentradores por cada uno' y 'fua' pero no tienen en cuenta que no todos los centros de salud tienen luz¿no? Y Cómo va a funcionar. Vi algunos centros tienen su concentrador pero no lo utilizan porque la falta de , del servicio. Lo bueno es que el covid se demoró en llegar a las comunidades porque ellos cerraron sus comunidades, tenían su sistema de que (Ininteligible, MINUTO 33:37)

**I: Perdón, perdón no la estamos escuchando**

**E:** Solamente, o sea así con casos (Ininteligible, MINUTO 33:53)

**I: Discúlpeme le perdí, le perdí por favor si pudiera repetir. Nos decía de que las comunidades se demoró en llegar el covid**

**E:** Sí, o sea solamente está más que todo(Ininteligible, MINUTO 32:22) estábamos que todo en capital de provincia ¿me escucha?

**I: No, no se escucha**

**E:** Capital de provincia

**I: Ya ahora sí**

**E:** O sea Nauta, Yurimaguas o sea la capital no más pero en las comunidades más lejanas, las comunidades tomaron la decisión de si alguien venía no dejaban entrar ni salir a nadie y cuando alguien tenía que salir o entrar le tenían aislado 15 días y de ahí recién le dejaban meterse a la comunidad. Ese fue su (Ininteligible, MINUTO 35:05)

**I: Su estrategia, ok. Doctora y luego de estas 2 primeras olas que fueron como las más intensas¿cómo fue resolviéndose el tema del oxígeno en adelante?**

**E:** Bueno este(Ininteligible, MINUTO 35:26)

**I: No le escucho, no le escucho. Ahora sí.**

**E:** Cómo bajo la… en la tercera ola los casos se fueron más leves¿no? O cuando terminó ya la segunda ola aunque de todos modos había uno que otro caso pero sí había capacidad para oxígeno, no ves que ya habíamos mejorado la… teníamos un mínimo ya de requerimiento y al bajar los casos ya teníamos suficiente

**I: ¿Cómo vio usted la respuesta tanto de la GERESA como de gobierno regional en esos momentos más críticos de la pandemia?**

**E:** Bueno este… pienso que han hecho lo que han podido ¿no? Por qué no había, no recuerdo si el gobierno regional llegó a comprar una planta, creo que no que compró, no llegó a comprar… no sé qué tanta participación tuvieron pero sí hicieron ,como gobierno regional, pero sí este apoyaron en algunas cosas logística, eso¿no? Que exactamente no supiera decir pero como veo desde lejos, como vi desde lejos era insuficiente¿no? insuficiente labor que realizaron

**I: ¿Y usted sabe si hubo alguna parte, no sé alguna directiva, alguna norma que establecieron a nivel de la región para ver el tema del oxígeno medicinal en los establecimientos de salud?**

**E:**Este… teníamos, no, no este… exactamente solamente se sacó lo de directivas a través del minsa. Que yo me acuerde no,no recuerdo. Ya no, o sea se empezó a reforzar porque se pensó en otra ola y mandaron a colocar una mini planta o un concentrador grande en los establecimientos I-4 ya o sea ellos ya tenían su propia red, pero

eso vino del minsa me acuerdo, colocaron toda una red para que también este… con tomas(Ininteligible, MINUTO 38:40)

**I: Doctora no la escucho, no la escucho**

**E:** La señal es mala

**I: Sí, si me toco el oído es que no lo estoy escuchando**

**E:** Ya, le decía que el miss a través de (Ininteligible, MINUTO 39:13)

**I: No la escucho**

**E:** El minsa¿ahí? El minsa colocó red de oxígeno, una mini red, no una red sino una como una central para que pongan, para que administre oxígeno en las emergencias, o sea tenía la capacidad de 6 balones de oxígeno que le conectaban a unos túbulos y salía pues en la… en los lugares ¿no? En cada cama de observación y en sala de partos. Eso fue lo que hicieron cuando ya pasó la segunda pandemia si no me equivoco, pusieron en todos los 1-4

**I: Ya pero esta infraestructura que usted me dice que le colocaron¿antes no tenían esto?**

**E:** No no había, sólo tenía el hospital regional y Essalud también tenía su red pero solamente uci porque los únicos que necesitan…

**I: Doctora¿ y esas redes están en funcionamiento hasta ahora?**

**E:** No le supiera decir, supongo que si no he probado no…

**I: ¿Y las plantas de oxígeno están en funcionamiento hasta ahora?¿Sabe usted qué ha sido de las plantas de oxígeno?**

**E:** (Ininteligible, MINUTO 40:52) que sí. Había una que estaba malograda justo la de Raymond, como Raymond compraron una planta de oxígeno de segunda o sea no era nueva él salió a denunciar de que no le habían dado el mantenimiento adecuado¿no? Porque supone cuando alguien te dona, la persona que lo recepciona ya está asumiendo el mantenimiento¿no? Parece que no tuvo el adecuado mantenimiento y un poco que se deterioro. Sin embargo creo que alguien , alguien le puso¿no? Le puso este, le arreglo y ya está funcionando no supiera decirle tuviera que hablar con alguien del hospital. Actualmente no sé si está funcionando, las 2 pero si funciona la del hospital.

**I:Ok. Y Generadores, los respiradores ¿esos sí han quedado en funcionamiento?¿Que ha pasado con todo ese equipo que fue llegando a Iquitos?**

**E:** Todo está en su lugar, han venido a comprar este… ha venido a controlar la defensoría, organismo de control interno para que vean dónde están sus actas todo, siempre hay un control.

**I:¿Quién realiza el control?¿Solamente a nivel regional o también van del minsa?**

**E:** Supervisa a nivel del minsa y la opinión pues no(Ininteligible, MINUTO 42:32) lo que le decía OCI que es del gobierno pero es un ente sancionador y la defensoría del pueblo que también es pues de la población¿no? Para que vea los derechos del poblador.

**I: ¿Y sabe usted sí está supervisión, esta revisión de los equipos se ha hecho recientemente o cuándo es que se ha realizado?**

**E:** Hacen casi todos los años, cuando yo estaba en su momento en una Ipress sí se iban y verificaban¿no? Pero como le digo ahorita mi función es fuera de un centro de salud, que hayan llegado del minsa para supervisar y se hayan presentado no. Como nosotros somos una región endémica hemos estado más, ahorita tenemos una epidemia de dengue y como que no sé si no se abastece, que se yo pero ahorita es full dengue. Pero nosotros tenemos otras arvovirosis pues no solamente es dengue.

**I:Ok. Usted desde su labor asistencial y ahora su labor en gestión¿cuál diría, digamos que son la principal o las principales carencias en cuanto al tema de oxígeno? Son más de digamos infraestructura, más de personal, más de recursos económicos para la compra de oxígeno que no o de las plantas¿dónde está la mayor dificultad?**

**E:** Niveles señorita, acuérdese que nosotros tenemos todos los establecimientos de salud fueron construidos en la época de o la mayoría el(Ininteligible, MINUTO 44:43)

**I: no le escucho, no le escucho**

**E:** La mayoría de establecimientos de salud¿me escucha?

**I: Sí**

**E:** La mayoría de establecimientos de salud fueron construidos en la época de Fujimori. Con Fujimori se hizo bastantes arreglos, cuando yo veo las placas de inauguración y eso más veo de eso, incluso con nosotros presidentes fue muy poco la inversión en cuanto a infraestructura, fue muy poco. Entonces si usted se pone a analizar el crecimiento poblacional, el alza de la demanda ha dificultado una buena atención ¿no?porque la infraestructura del establecimiento ya no están las condiciones para el crecimiento poblacional que han tenido en los últimos 20 o 30 años. Por ejemplo antes no había SuSalud, o tal vez había no lo sé pero la cosa es que las reglas ahora son más específicas¿no? Qué tiene que tener un consultorio debe tener tantos metros por paciente, por esto, que tiene que tener un lavado de manos y tanta cosa(Ininteligible, MINUTO 46:16) mayoría se encuentra en infraestructura, eso es uno y que no afecta solamente lo que es del covid, afecta todo porque nosotros estamos en una todos los años prácticamente de enero a marzo tenemos incremento de casos de dengue¿no? Entonces nosotros estábamos en casos de dengue cuando el covid desplazó pasamos al dengue, no tuvimos descanso nos pasamos del uno al otro

**I: ¿Cuáles….? No le estamos escuchando**

**E:…** hablando, dígame

**I: Sí, sí nos decía que ustedes pasaron de la ola del covid al dengue inmediatamente. Hasta ahí la escuchamos**

**E:** Dengue, sí dengue

**I: Doctora si tuviéramos una cuestión o una epidemia similar a la que tuvimos con el covid¿cuál sería la situación del oxígeno en este momento en Iquitos?**

**E:** (Ininteligible, MINUTO 47:49)

**I: No la escucho, no lo escucho doctora**

**E:** Tendríamos mejor acceso pero no suficiente si es que tuviéramos la intensidad de la primera ola, si es la segunda ola yo creo que sí tuviéramos mayor casos

**I: Mayores posibilidades**

**E:** Mayor posibilidad

**I: ¿Qué diría que se ha aprendido?¿qué diría usted que se ha aprendido respecto de o que ha cambiado respecto del abastecimiento o en general sobre todo el tema del oxígeno en su región?**

**E:** ¿Como dice? No se le escuchó bien

**I: Sí, le preguntaba¿ qué se ha aprendido o que ha cambiado en su región en el tema del oxígeno ahora? Las autoridades, los directivos de los establecimiento de salud, el personal de salud en general. En general ¿qué cosas se habrá aprendido?¿Qué cosa ha cambiado si es que ha cambiado algo?**

**E:** Bueno, no supiera exactamente qué decirle... Usted sabe que el cambio es más este de conducta ¿no? Ahora creo que todos se han quedado más en(Ininteligible, MINUTO 49:51) como a nivel este de familia¿no? Pero sin embargo, entonces este sin embargo creo que la parte de organización en el nivel regional es insuficiente y que el apoyo, es mi opinión personal, el apoyo por parte del minsa ha sido muy lento ha sido. De todas maneras sigue siendo lento ahora con el dengue también yo veo que la respuesta es muy lenta, no nos hacen mucho caso. Si bien es cierto ahora dependemos del gobierno regional, pero correrse que como gobierno regional a veces no se puede comprar las pruebas diagnóstico porque por ejemplo ¿no? Este... Nos mandan plata para comprar y hacer pruebas de qué tipo de dengue está circulando porque según el tipo nosotros podemos prever qué tan agresivo puede ser¿no? O cómo se va a comportar¿no? Los síntomas entonces nos dicen que para poder entrar a la plataforma el INS debe por ejemplo no solo capacitarnos y no autorizarnos, ellos mismos tienen que venir y autorizarnos para que podamos entrar a las páginas y registrar. Entonces yo le pregunto a mi directora '¿cuándo va a venir el INS?¿Cuándo va a venir el INS?' Imagínese la emergencia del dengue está desde , desde febrero 28, 27 de febrero y hasta ahora no nos da porque dice 'Ay el INS está muy ocupado viendo todos los casos de piura¿no? Que no tiene tiempo' y ya pues yo le entiendo ¿no? Pero ya han pasado cuántos meses y seguimos sin sin ese apoyo diagnóstico¿no? Tenemos que dar el tratamiento según la clínica ¿no? Y otras pruebas diagnósticas que tenemos, ya nos vamos a quedar sin dengue y sin saber qué serotipo era el que estaba circulando. Entonces de todas formas una dependencia, obvio pues que ellos no se van a abastecer yo me imagino que será 15 personas

**I: La perdimos completamente, perdimos lo que nos estaba comentando. Hasta la dependencia de parte de ellos fue lo que le escuché**

**E:** No se abastecen, no hay un plan b para extender(Ininteligible, MINUTO 52:57) para ampliar nuestra oferta de servicio

**I: ¿Para qué?**

**E:** Para ampliar nuestra oferta ante la demanda, se supone que el laboratorio no sé cuántas personas habrán encargadas de virología que es en el caso de dengue y no pueden todos salir de viaje hacer el soporte técnico porque si no quién se queda para ver¿no? O sea no hay un plan de contingencia para que recluten más personas y que hay un grupo que esté viajando por las regiones mientras el grupo local se queda procesando o haciendo las otras actividades. O sea tenemos un dengue, 2 pandemias de covid, deberíamos de estar mucho más familiarizados en el manejo sin embargo se sigue cometiendo algunas imprudencias más que todo en la parte organizacional

**I: Y usted por lo que ha visto¿es una cuestión solo de capacidad del personal o también habrá por ahí un tema de interés, de organización de ellos o no sé tal vez de digamos permanencia de directores? No lo sé hay diversos problemas por los que pasa el minsa también¿no? Rotación de personal tiene una diversidad de situaciones pero desde su perspectiva ¿cuál sería como lo más importante, la dificultad más importante para que puedan llegar a ustedes?**

**E:** Es que la organización es un todo, no es solo una persona es toda una red o sea el cambio tendría que ser de forma… o el reforzamiento tiene que ser no sólo la cabeza sino también la parte operativa, ese es el detalle no sé si me dejo entender.

**I: Sí ,es toda la estructura por lo que me dice**

**E:** O eso, hacia ahí iba la pregunta sí era de estructura

**I: Ok, bueno no tengo más preguntas hasta aquí pero me gustaría saber si usted tiene algún comentario final que quisiera hacer respecto al tema del oxígeno por la experiencia que ha vivido tanto en la labor asistencial como en la labor de gestión que tiene en este momento**

**E:** Bueno este… ha sido momentos muy difíciles, de verdad no quisiera pasarlo de nuevo yo este… pero si hubiera otra epidemia así yo creo que igual nos volvería a golpear por el mismo hecho que tenemos una infraestructura insuficiente, no solamente en loreto sino también en establecimientos de salud de puno no sé .Escuchándoles en zoom a los que están golpeados por el dengue se nota que todavía esa brecha es alta ¿no? El

otro es la falta de recursos humanos por ejemplo nos han mandado plata para contratar más personal pero no quieren, no hay pues porque no es suficiente personal y no pueden ahí leyes que no nos dejan trabajar en 2, 2 lugares para el estado, entonces alguien del Braem se quejaba y decía '¿quién va a venir por 5000 soles poniendo en riesgo su vida? Este y por ese sueldo' sacaron una emergencia y pusieron el mismo sueldo para todos los médicos, enfermeras, biólogos a nivel nacional imagínate; si sabemos que las realidades son distintas en cada región, o sea no se ha aprendido lo suficiente o tal vez el grupo que estaba en covid es otro en comparación con el que está acá no podría asegurarle eso pero no hay lógica¿no? De que te manden plata y que no puedas usar porque ni nosotros pagamos tan poco a los médicos y ellos han puesto ese precio¿no? ese sueldo que es obvio que nadie va a aceptar, todavía dependemos del centralismo(Ininteligible, MINUTO 58:12) yo pienso eso no que si hubiera otra pandemia pasaríamos, tal vez no con la misma intensidad porque habremos mejorado un poco, pero sí que no podríamos manejar como tal vez se debería ¿no? Solamente por la misma infraestructura, falta de recursos humanos, falta de medicamento mira con esto del dengue no hay pruebas, se han acabado las pruebas para el dengue(Ininteligible, MINUTO 58:48) nos han dicho que compremos, que veamos de dónde vamos a comprar 'busquen un proveedor' el mismo INS pero eso sí nos dicen que compremos de buena, de garantía porque si no es garantía están malversando los fondos, gastando en algo que no es¿Entonces qué hace uno,no? Ya ha superado su capacidad porque todo está centrado en piura y eso que sólo están con dengue determinados distritos, 53 distritos o sea el covid no pasó por Perú y seguimos teniendo ese centralismo, ese cuello de botella, esos este… documentos¿no? Esas Normas técnicas, los decretos de urgencia encasillados en un modelo que no está adaptado a la realidad de cada región. Ese es el detalle.

**I: Muchísimas gracias doctora B ha sido un gusto poder conversar con usted. En este momento voy a apagar la grabación**

**013 Entrevista**

Meeting started: 25/7/2023, 20:30 pm
Participantes: E, I
**I: Investigador**
**E: Entrevistado**

**I: Buenas noches, hoy es 25 de julio del 2023. Voy a comenzar una entrevista con participante de una región de la selva del Perú y a solo para que quede registrado, doctor, podría usted confirmarme que está usted de acuerdo con que grabemos la entrevista**

E: Si estoy de acuerdo.

**I: Ok, este esta entrevista es parte del estudio que la Universidad Cayetano Heredia está realizando y cuyo título es economía política Del sistema de oxígeno medicinal en el Perú. Eh, doctor, solo para una cuestión general como una referencia para saber con quién estamos conversando, podría usted, por favor, comentarnos a cuál es su labor, a que se dedica a usted y tal vez su relación con el tema de los servicios de oxígeno medicinal durante la pandemia, no sé si ha sido así siempre o es parte de su tarea cotidiana.**

E: Yo soy médico especialista en enfermedades infecciosas, he sido director del hospital 3 años y medio hace unos años y durante la pandemia estuve a cargo de organizar la respuesta frente al elevado número de casos que hubieron de COVID-19.

**I: Esto como parte del equipo del hospital, parte del equipo de la DIRESA**

E: Yo era responsable de implementar la respuesta en el HR

**I: Usted, continua en esa labor en este momento me refiero a si sigue laborando**

E: Era para la pandemia nada más para la respuesta a la epidemia y ahora yo soy un médico del departamento, ya no estoy en ese cargo. Y tampoco es necesario porque no había que no hay que el Hospital Regional fue un hospital dedicado exclusivamente a la atención de pacientes con COVID-19. Todos los hospitales, todos los pacientes del Ministerio de Salud que se enfermaban y que requerían manejo especializado eran llevados. Tuvimos en algún momento como a 600 pacientes y la capacidad del hospital es 270.

**I: Podría con comentarnos cómo fue el tema allá en relación, sabemos por la información a través de las noticias que Iquitos fue una de las zonas más afectadas por el tema del COVID, pero concretamente respecto el tema del oxígeno, podría usted contarnos ¿cómo fue?**

E: Bueno para nosotros fue particularmente crítico debido a que el nivel de consumo de oxígeno que tienen los ventiladores mecánicos es muy alto. Son varios balones al Día de consumo de cada uno, el hospital no tiene o no tenía una planta de oxígeno con capacidad para satisfacer ni el 10% de la demanda y para Iquitos, el transporte de oxígeno es extremadamente complicado porque no hay una planta o no hay un lugar donde se pueda comprar el oxígeno no hay ninguna empresa que la que venda oxígeno medicinal, así que el oxígeno era necesario que sea traído desde Lima. Por esa razón se estableció un puente aéreo donde traían balones de oxígeno porque los que habían localmente eran extremadamente

insuficiente, yo creo que no superaba y de la de las necesidades se estaría cubriendo entre un 60% con todo el esfuerzo de traer oxígeno de fuera y además es lo que el mismo hospital podía proporcionar.

**I: Previo la pandemia ¿Conoce usted cuál era la necesidad de oxígeno? ¿Si digamos, es la suficiente para atender a los pacientes de diversas condiciones que requerían de este insumo?**

E: Sí. Previo a la pandemia sí, en hospitalización hay el sistema de lo que pasa es que los pacientes tienen requerimientos alto de oxígeno cuando tiene un compromiso pulmonar severo y no es usual que tengan en las caracterización comunes este tipo de pacientes, por lo tanto, pacientes con daño pulmonar severo que se veían en los unidad de cuidados intermedios o que se veían en la unidad dos intensivos y puedo decir que el abastecimiento de oxígeno era suficiente, nunca por lo menos he tomado conocimiento de que haya ocurrido un desabastecimiento que haya puesto en riesgo la vida de las personas. ¿puede a ver ocurrido? Si, recuerdo que en algún momento ha ocurrido un período breve que hubo sí, en la UCI una falta de oxígeno, pero fue una cuestión totalmente inusual en general sí había suficiente oxígeno para las necesidades del hospital.

**I: Entonces digamos esta necesidad de oxígeno propiamente esta demanda, alta demanda fue propiamente por el tema del COVID ¿recuerda usted más o menos cuándo empezó para para ustedes esa necesidad?**

E: El déficit de oxígeno se produjo alrededor de la quincena de abril aproximadamente

**I: Y ¿cuáles fueron las acciones? ¿Cómo es que fueron sufriendo resolviendo el tema?**

E: Lo único que el Ministerio de Salud proporcionó algunos equipos de oxígeno pequeño que dan hasta 10 Litros y lo demás fue traído y no se pudo implementar ninguna planta nueva durante ese periodo todavía porque había un desabastecimiento de plantas y lo único que nos ayudó fue ese puente que hicieron donde traían, yo no puedo tener el dato exacto en este momento, pero eran algunos deberían ser uno 100 o 200 balones, ese dato exactamente no lo tengo porque yo no manejaba el dato de la cantidad de oxígeno pero sí diariamente llevaban y traían, llevaban balones vacíos y traían. Para Iquitos para proporcionar oxígeno solamente era vía aérea no hay forma de que por vía fluvial pueda llegar oxígeno se tomaría dos semanas y es un sistema que no se optó porque no la necesidad era urgente así que la única forma de abastecernos era transportándolos por vía aérea.

**I: Y fue, digamos, sencillo o tuvo algunas dificultades está el establecimiento de este puente aéreo.**

E: Es muy caro porque tiene que traerlo en avión, costos exactos es son 1 hora y 30 minutos de vuelo, por lo menos a 1 hora y 45. Entonces es extremadamente oneroso y caro y deben haber implementado unos dos o tres vuelos para la cantidad suficiente de oxígeno que se requería.

**I: Esto fue viabilizado a través del Minsa o del Gobierno regional.**

E: El Minsa.

**I: Y qué otras medidas o qué otras formas tuvieron ustedes de afrontar la el déficit de oxígeno en Iquitos.**

E: No hay alternativas, esos oxigenadores pequeños que pueden producir conectados hasta, pero hubieron unos cuántos no fueron muchos no fueron muchos en realidad no es una solución. Realmente probablemente muchas personas se complicaron porque tuvieron insuficiente oxígeno porque no, no

tenían el oxígeno, que era vital para poder sobrevivir y también no había ventiladores mecánicos suficientes

**I: Y eso fue digamos al inicio, luego entiendo de que se implementaron plantas de oxígeno o no llegó.**

E: Sí hubo una donación de una planta, pero en realidad la producción fue poca Y gradualmente la demanda fue reduciéndose, así que por lo menos como para junio ya la demanda empezó a bajar. Entonces el período crítico fue entre, digamos, la quincena de abril, mayo hasta la quincena de junio, ya a fines de junio ya fue reduciéndose la demanda.

**I: Y en adelante ustedes no tuvieron más dificultad con el tema del oxígeno medicinal.**

E: No, ya cuando vino la segunda ola para el año siguiente ya se había implementado una nueva planta de oxígeno en el hospital y con eso había crecimiento suficiente.

**I: ¿Y la implementación de estas plantas tuvo alguna dificultad? ¿Cómo fue que se llegó a implementar?**

E: Bueno, los detalles de los procesos para las plantas no conozco bien porque era parte de la gestión del director y yo no estaba personalmente encargado de eso pero era muy difícil poder encontrar plantas de oxígeno que podían ser armadas rápidamente y luego no teníamos vuelos, o sea, la logística en Loreto es complicada por todos lados, porque si queremos tener un equipo, el equipo tiene que ser transportado por vía aérea, así que nuevamente los costos se elevan un vuelo pequeño no baja de 15000 a 20000 dólares para transportar cuatro o 6 personas estoy solamente hablando de eso entonces la información que he tenido es que se hizo la gestión hubo la donación de una planta por parte de los un grupo de sacerdotes pero la planta no respondió bien, empezó a funcionar, pero no fue suficiente ya luego crearon otra planta pero eso fue como unos varios meses después, 6 meses después que tuvimos la planta, por eso ya cuando hubo la necesidad en la segunda ola ya el oxígeno estaba disponible.

**I: Y eso fue el contar con esta planta en el hospital fue como suficiente para toda la necesidad que se tenía**

E: Para la necesidad que hay ahora y que había después también cuando ya no había el número tan elevado de casos era suficiente

**I: Y en algún momento en otras regiones nos han comentado que tuvieron algunas dificultades para la implementación de la planta más de estructura de espacios en los hospitales**

E: Nuestro hospital es bien grande es un hospital que tiene como 50 años y si tenemos bastantes espacios donde poner una planta de oxígeno

**I: ¿Sí, pero y las condiciones? ¿Lo que es requerimientos técnicos? Porque tener una planta en un hospital no solamente el espacio, no es tener las conexiones.**

E: El problema es que la planta que teníamos en el hospital estaba hecha no solamente para abastecer a través de un sistema de suministros empotrado para los lugares críticos, porque usualmente no se ponen para todos y además tenía capacidad para embotellar balones, el problema es que cuando empezó la epidemia este sistema no estaba operativo al cien por cien quiere decir que la planta cuando se apareció la necesidad la planta no tenía toda la capacidad de poder hacer el embotellado de balones de oxígeno, porque esta planta producía para suministro en alguna parte de la red que de suministro y también prepara o preparaba balones, no llenaba balones que era lo que se distribuían a lugares donde se

necesitaba de manera especial pero no estaba bien los sistemas de válvulas habían malogrado, no podían embotellar o la cantidad de botellones que podían llenar de balones era solamente unos cuántos que estaba lejos de la de la necesidad.

**I: Y a cuando ya implementaron esta esta planta nueva, porque me dice que fue el posterior, ¿se superaron esas dificultades?**

E: Sí, yo por lo menos hasta donde puedo advertir y opinar no he podido ver que haya existido nueva deficiencia o falta de oxígeno después de esa época.

**I: Y a nivel más de normas entiendo que a nivel nacional se dieron algunas directrices, algunas normas para la respuesta, pero no sé si a nivel regional ustedes o como equipo en su equipo de respuesta Hubo alguna directriz, alguna norma que digamos**

E: No en relación al nivel de concentración de oxígeno que había dispuesto la norma ese no fue inconveniente para nosotros, nosotros tenemos tan limitadas opciones que no había la posibilidad siquiera de poder acceder a menores niveles de concentración o niveles más altos, solamente lo que establecía la norma era lo que hemos podido con lo que hemos manejado, no habían opciones de aparte, tampoco se puede la forma líquida de oxígeno que se puede tener y que podría ser, tiene un alto rendimiento no aplica para Loreto, no era una opción para nosotros porque no se puede transportar por vía aérea y por vía fluvial, también es un tremendo inconveniente, a diferencia de lo que sí en la costa se puede hacer.

**I: Entonces la única posibilidad de que ustedes tenían eran los balones de oxígeno y producir allá a través de las plantas**

E: Así es.

**I: Desde lo que usted ha podido ver, si bien es cierto, en ese momento eran las únicas posibilidades que tenía. ¿Usted considera si la norma de necesita ser ajustada o revisada teniendo en cuenta la realidad de Iquitos o de Loreto o la selva en realidad? No** necesariamente la ciudad.

E: Bueno, hasta cuando conozco la norma yo no creo que eso tenga un impacto sobre la disponibilidad de oxígeno en Iquitos.

**I: ¿Y en función, a qué?**

E: No creo que tenga un impacto sobre Iquitos en nuestro caso no conozco el detalle en las normas porque es una es mi énfasis entonces de lo que tengo conocimiento la norma no nos afecta.

**I: Digamos como que no se aplicaría a la realidad que ustedes tienen, a eso se refiere.**

E: Yo no puedo afirmar eso porque yo no conozco bien la norma oxígeno no es mi énfasis, entonces yo he manejado solo el requerimiento, ya no puedo afirmar que no se ajusta las necesidades nuestras, ahora si me preguntas específicamente algún tópico, yo podría responderte o decirte si eso aplica o no aplica algún ítem en exclusivo

**I: Ya y respecto de los requerimientos, ustedes tuvieron alguna dificultad o facilidad para el tema del financiamiento para la adquisición del oxígeno medicinal.**

E: Sí. Pero no solo es ojo que la demanda que aparece en la ciudad no solamente es el hospital, sino también de todos los pobladores que quieren tener su balón adicionalmente. Nosotros nuestra necesidad estaba muy por encima de lo que había disponible muy por encima.

**I: ¿Y cómo hicieron para, digamos conseguir o lograr la compra de esto adicional que se tuvo durante la pandemia?**

E: Yo entiendo que el Gobierno regional y Ministerio de salud ayudaron con eso

I: Y usted digamos, sabe si se tuvo dificultades y si así se tuvo alguna.

E: La dificultad es que tuvimos, ah para la compra eso tomó un poco de tiempo en esas circunstancias ni el dinero que servía porque tienes donde comprarlo no había plantas. Si querían donar a alguien una planta no había dónde comprarla, pero los recursos fueron proporcionados no creo que hubieran dificultades en acceso en ese momento si el hospital recibió apoyo para contrato de personal y el abastecimiento de oxigeno

**I: Y lo que no había era, digamos los proveedores y ustedes en Iquitos en el hospital ¿quién es el proveedor del oxígeno para el hospital?**

E. El hospital tiene su planta, pero la planta está solamente lo que producirá suficiente en realidad y se compraba un adicional, se compraba unos balones adicionales cuando la demanda se superaba, entonces entre lo que el hospital producía y el incremento que podría ocurrir se completaba con lo que podía tengo entendido que hay un proveedor local también pero no tiene mucha capacidad de venta de oxígeno y con eso se completaba pero el hospital estaba solvente para la necesidad de oxígeno que había.

**I. Previo a la pandemia, dice usted.**

E: Antes de la pandemia.

I: Y estos proveedores el local y no sé si solamente local digamos ¿cuál fue su nivel de abastecimiento? ¿hubo una mayor producción?

E: Yo no conozco el detalle de cómo ellos cuánto capacidad de envase tienen o si ellos lo envasan o los traen eso no lo puedo tendría que averiguarlo no puedo precisarlo, pero tremendamente insuficiente, ni siquiera podíamos contar con eso.

**I: ¿Cuál diría usted que es la principal carencia de recursos para el abastecimiento del oxígeno medicinal? Ya me ya me ha hablado de que el tema digamos de los espacios el hospital no es una dificultad para usted, puesto que hay bastante, o sea tienen lugar donde, pero el personal, tal vez la infraestructura, no sé manejar una planta requiere implica varios recursos.**

E: Mira Recursos Humanos, técnicos que la manejen ya teníamos una planta que no era muy grande y se mantenía aceptablemente si creo que no tenía un plan de mantenimiento y reparación bien organizado y es por eso que no estaba al tope su funcionamiento cuando se presentó la necesidad. Pero tenemos recursos Humanos, lo que no es el plan y eso es una cuestión ya de gestión y tenemos el espacio también.

**I: ¿Usted sabe si se viene haciendo mantenimiento a las plantas en este momento? la situación de las plantas de oxígeno en este momento.**

E: No, no tengo información de eso no sé si le darán mantenimiento actualmente

**I: Pero y están en funcionamiento todavía, las dos están funcionando.**

E: Sí, las dos están funcionando.

**I: Respecto de quién digamos es la responsable en no sé si hospital o si usted conoce también lo que ocurre a nivel de la de la GERESA. ¿Quién es el responsable del tema de la seguridad del oxígeno medicinal?**

E: En la cereza, no. El hospital es una unidad ejecutora y ellos se mueven de manera independiente la GERESA no está a cargo de eso, el hospital tiene su presupuesto y se mueve de manera independiente.

**I: Y usted sabe cuál es el proceso, procedimiento que siguen para saber si hay suministro en suficiente cantidad, si se cuenta con todos los insumos o dispositivos que se requieren en el hospital.**

E: Actualmente, eso no lo puedo precisar

**I: Y durante la durante la pandemia. ¿Quién era digamos el responsable de ver este tema?**

E: El soporte de la planta. ¿A esto se refiere?

**I: Sí, el soporte de la planta, saber si digamos si está produciendo la cantidad necesaria, ver los flujos que se necesita**.

E: En realidad eso de ahí era desde mi punto de vista manejado de manera bien empírica, no hay una planificación de la necesidad ni hay una previsión de cómo podría incrementarse la capacidad si fuera necesario y la mayoría de trabajadores en realidad los equipos funcionaban hasta donde fuera posible y la mayoría de los trabajadores estaban enfermos en ese momento, pero cuando no ocurre en epidemia, sí hay un grupo de técnicos que son los que le dan mantenimiento le dan soporte.

**I: Ustedes llevan o llevaban durante la pandemia algún control, un registro de las necesidades de oxígeno y de la producción del oxígeno para ver cuál era el déficit o si o si había o no déficit de la producción o algo así.**

E: Nosotros lo que hacíamos llegar a la dirección era el requerimiento de lo que de lo que teníamos. Pero en realidad era una cosa desmesurada comparado con la capacidad que tenía, pero esa parte de planificación la ven los de gobierno, no la ve director, la ven en los de logística, los operativos no vemos esa parte de planificación.

**I: ¿Qué tan grave fue el déficit que ustedes tuvieron de oxígeno? Yo sé que ya me comentó que no tiene como datos exactos, pero sí usted nos diese como una versión cualitativa del del tema, ¿Cuán grave o cuán difícil fue ese el déficit de ustedes tuviera?**

E: ¿Como cuánto nos puede haber faltado? Entre ventiladores mecánicos y porque ventiladores mecánicos también era una necesidad yo estimo una aproximación que sea más o menos, lo que pasa es que la dirección era incapaz de poder programar cuanto se necesitaba y cuando tenía, solamente recibían o pedían todo lo que podían y los distribuían hasta donde alcanzaba, nada más, entonces yo no puedo estar seguro a cuántos o tantos lugares y no he podido verificar una estadística de cómo iban haciendo y donde simplemente el que no tenía oxígeno. Pero se hizo un gran esfuerzo las personas recibieron, yo podría decir que un 10% no, ya no tuvo, pues porque yo me encargaba de conseguirle balones, pedimos prestados a los de UCI, que tenían cuatro balones y le dábamos a los de piso hasta que el otro le llegue su balón y llegue la otra remesa. 10 o 15% no debe haber tenido ya suficiente oxígeno para lo que necesitaba

**I: Usted me dice que la dirección es responsable ¿la dirección del hospital o de algún área en específico?**

E: La dirección del hospital estaba, con ellas coordinábamos nosotros.

**I: En función a la experiencia lo que usted pudo ver observar en la región ¿cuáles considera usted que podrían ser? Y no sé si eso es algo que está relacionado con su trabajo y está bien si me dice que no ¿Cuál es diría usted que son como las áreas o los aspectos claves en los que podría enfocarse, centrarse cualquier nueva política sobre el oxígeno? No sé si te atreves algún enfoque sanitario.**

E: Yo creo que el hospital y los hospitales deberían tener un plan de respuesta frente a un eventual incremento de la demanda y tener primero digamos un incremento del 30 a 50 o 100% si se presentara otra epidemia. De esa manera, prever si yo tengo mi capacidad ahorita estimar la capacidad actual y con esa capacidad actual ver hasta cuánto se podría ampliar al máximo como respuesta ante un incremento de la demanda y tener previsto que se va a hacer si la demanda se duplica, triplica, yo dudo, yo no sé directamente pero no existe un plan de ese tipo por lo menos en el hospital donde yo estoy.

**I: Y he de acuerdo a lo que usted también ve en el hospital ¿Qué áreas requerirían como mayor énfasis o mayor desarrollo o aspectos de lo que usted ve en el hospital que deberían ser tomados en cuenta?**

E: Primero, tienen que ser un diagnóstico de base. ¿Qué capacidad de suministro de oxígeno tiene? ¿A cuántas camas y cuántos ventiladores pueden suministrar oxígeno de manera adecuada y aquellos que tengan altas demandas? Y ¿Y qué área debería hacer eso? debería ser liderado por el director, debería estar en el área que va depender de quien hace el plan, quien hace el plan para que haga el requerimiento, los usuarios tendrían que dar una expectativa, pero también tendría que estar suscrito por la visión del de los que gobierna tanto del área de director de la Dirección General para saber qué cosa, qué tipo de plan quieren hacer y eso coordinarlo con su área de planificación y logística.

**I: Y eso solamente a nivel de hospital Allí tendría que ver algo el Gobierno regional, porque es como yo me dijo Unidad Ejecutora.**

E: Sí en el hospital cuando tiene una necesidad que sobrepase su capacidad, acude al Gobierno regional, pero el Gobierno regional no está obligado a solucionarlo como una prioridad para ellos hay una serie necesidad que tiene el hospital que el Gobierno regional no puede ayudarle, el hospital tiene que ser autosuficiente en solucionar sus problemas hasta donde sea posible, solo en casos excepcionales ya interviene el Gobierno regional a con apoyo económico financiero técnico.

**I: Usted hace unos momentos me ha mencionado que para digamos qué aspectos debería ser tomados en cuenta, me dice el tema de conocer o tener el diagnóstico de la demanda y también de lo que se podría se debería incrementar, pero qué hay con el tema de los Recursos Humanos que no sé si también con el tema de la infraestructura, la adaptación de la infraestructura.**

E: Recursos Humanos si tuviera que yo creo que de todas maneras es necesario contar con más personal técnico para mantenimiento de la de la planta y el soporte, lamentablemente el hospital no tiene capacidad ya de poder contratar más gente y menos aún gente con buen nivel de calificación para esos puestos pero sí se requeriría personal porque son equipos que trabajan 24 horas al día y una gran demanda entonces hay que tener todo un soporte técnico y personal de mantenimiento, eso es insuficiente. Espacios si tuvieran que construir una nueva planta más grande, si tiene el hospital.

**I: Y las conexiones, si es si todavía están con balones o ya tienen una red para el tema.**

E: No, no está montado el sistema propiamente, han mejorado, lo han incrementado, pero solamente el abastecimiento a través de tuberías es para lugares específicos. Ha mejorado un poco, pero es un el sistema, ya tenía un sistema, pero el sistema estaba sin mantenimiento estaba deteriorado por eso hay múltiples lugares, salas que el oxígeno no ha llegado, pero ojo que estamos hablando, pues cuando la demanda de oxígeno es pequeña, no pocos litros, pero cuando hay demandas de grandes litros ahí sino. Entonces el sistema sí de suministro al interior del hospital no es adecuado, está deteriorado.

**I: ¿Y si usted, Ah, ¿cómo de manera general nos pudiera comentar cuáles fueron para usted los retos más importantes respecto del oxígeno medicinal durante la pandemia?**

E: Lo más difícil fue que no había donde más conseguir porque somos una isla realmente ósea cuando la demanda se incrementa no hay de dónde más traer de dónde más poder conseguir suministro de oxígeno, o sea el hospital por las características de está ubicado debería tener un plan robusto de respuesta frente a un incremento de la demanda y tener ya calculados si van a hacer van a traerlo porque vía cuánto tiempo planificarlo y poder, tendría que tener una buena planificación de cómo abastecer de oxígeno para una alta demanda. Pero que localmente no se ha incrementado el número de plantas que hay en la ciudad porque cuando no hay demanda no tiene sentido para una empresa vender oxígeno medicinal cuando no hay nadie que te lo compre el hospital tiene su capacidad y te compro un poco entonces tiene que preverse, cómo se va a abastecer y de dónde se van a traer pueden ser de lugares que tienen carreteras como Pucallpa, Tarapoto y de ahí el tramo en lancha para que pueda bajar los costos, pero eso tiene que ser una respuesta rápida y organizada porque este tramo desde Lima o de otra ciudad toma tiempo, toma 5 o 6 o 7 días por lo menos.

**I: Revisando todo lo ocurrido en durante la pandemia. ¿Usted diría que fue suficiente que fueron suficientes las acciones que se realizaron para resolver el tema de la deficiencia de oxígeno que tuvieron, que se podría si no fue así, qué se podría haber hecho mejor?**

E: No, no fueron suficientes y las personas colaboraron mucho, las personas que ya no ya el hospital; las propias personas conseguían sus balones para sus pacientes, el hospital no tenía la capacidad y cada una de manera individual veía la forma porque había múltiples formas, seguramente traer diferentes a las del puente aéreo y la gente conseguía y llevaban sus propios valores es que en realidad la demanda fue tan excepcionalmente grande que es difícil poder esperar que ocurra algo así de nuevo, pero yo creo que si volviera a ocurrir un brote de ese tipo, volveríamos a tener la misma necesidad de oxígeno, no considero que estemos ahorita listos para responder un nivel donde hay nosotros nuestra sala de UCI solo tiene 6 camas y de pronto se convirtieron en 20 los ventiladores, 20 camas, 30 camas y cada 1 tiene un consumo de 3 o 3 balones al día dependiendo de la concentración, en solamente eso hay una tremenda demanda entonces yo creo que el hospital no fue capaz de responder a toda la necesidad que tuvo, recibió el apoyo, pero fue un apoyo que se demoró y que fue insuficiente. y que afortunadamente el número caso fue gradualmente reduciéndose y de nuevo yo creo que el punto de la planificación para la respuesta debería ser una consideración partiendo del diagnóstico de base, de qué capacidad tenemos para producir y también rediseñar y reparar y dar mantenimiento a todo el sistema de suministro del hospital que está deteriorado, pues en múltiples áreas.

**I: Teniendo en cuenta lo que usted me comenta de la situación actual del hospital. ¿qué se ha aprendido? Porque digamos la situación que ustedes vivieron fue bastante crítica, bastante difícil, compleja. ¿Pero usted percibe que hubo algún aprendizaje, algún cambio en el hospital? ¿A raíz de esto?**

E: Mi impresión es que si hubiera otra epidemia experimentaríamos lo mismo, siento que no hay una enseñanza que se ha traducido en hechos concretos, simplemente bajaron los casos y pasó las circunstancias no creo que puedo si vuelve a aparecer un número así de casos no va a haber una buena respuesta, vamos a tener las mismas deficiencias o por lo menos parecidas.

**I: ¿Pero de quién depende que haya o que se tomen las medidas adecuadas que se genera? (42:03)**

E: Lamentablemente los directores duran poco tiempo, no hay una duración en las gestiones y no hay la preocupación y no hay la priorización para considerar que este es un tema crítico cuando ya no hay casos los tomadores de decisiones se preocupan cuando hay un problema, cuando el problema pasa ya lo dejan de lado porque tienen muchísimos problemas, más deficiencias, falta de pago de personal, faltan medicinas, faltan insumos de laboratorio, tienen tantos problemas que no prevé ni tienen planificado cómo responder si hubiera un incremento de la demanda y probablemente ni siquiera tengan bien calculado hasta cuánto pueden abastecer.

**I: Entiendo que en Iquitos hubo también apoyo de la Iglesia de otras instituciones que digamos hicieron compras de plantas de oxígeno y trataron de implementar otras plantas en la ciudad ¿cómo vio usted la participación o el trabajo que ellos hicieron fueron efectivos realmente lograron el apoyo pudieron concretar el tema de las plantas de oxígeno?**

E: Hubo el apoyo de la Iglesia, pero la planta no ayudó mucho, no funcionó bien y eso es todo lo que hubo. Nadie más.

**I: Bueno, ya para ir cerrando. ¿Qué lecciones clave usted podría compartir a raíz de la experiencia del trabajo que usted dio o del equipo que usted participó para la respuesta del COVID-19? Por el tema concreto, el oxígeno medicinal**

E: Yo creo que una de las enseñanzas es que debemos estar preparados para una elevación de la demanda. Luego que servicios críticos debería mantenerse un sistema de suministros en buenas condiciones, entonces los servicios disponibles y abastecimiento de oxígeno deberían recibir un permanente y adecuado mantenimiento preventivo y reparación cuando sea necesario. Luego tener una planta que tenga por lo menos 1 capacidad de incrementarse al doble la producción y de esa manera, ante cualquier eventualidad tendría la capacidad.

**I: Usted me ha comentado hace un rato que a veces la balanza de dificultades o de problemas que hay en el día a día hacen que se pierda el foco de la previsión de lo que podría ocurrir en una situación de emergencia y que, bueno, los directores se ven como desbordados por esa situación, pero habría alguna forma de generar no sé si a través de una norma o través de un cambio en la cultura institucional, no sé alguna forma en la que las autoridades pudieran tener esto en cuenta.**

E: Es que las autoridades tienen que responder de acuerdo a las prioridades, ellos tienen una serie de tantas deficiencias.

**I: Eso a nivel del del hospital, pero y si nosotros escalamos ese nivel de responsabilidades o de si bueno podría ser responsabilidades, ¿quién estaría como como digamos o de quién dependería de quién podría hacia arriba intervenir?**

E: Ellos, miren los gerentes y los tomadores de decisiones en las de los servicios de salud del Minsa me refiero específicamente están en el día a día solucionando sus problemas, ellos están respondiendo a los

apremios, a las dificultades que se les presenta permanentemente en un hospital antiguo, como sin mantenimiento, sin equipo, sin personal entonces yo dudo que ellos tengan la capacidad y la disposición y sea un problema en este momento, por ejemplo, en estas circunstancias, una de las prioridades para ellos y esto porque no se ha aprendido que puede haber la necesidad y hay que priorizarlo, entonces lo que sería recomendable es que se les dé un soporte y esto tiene que ser una iniciativa vertical que venga desde arriba hacia abajo a través de nuestras autoridades de alto nivel a través de disposiciones como directivas del propio Minsa que establezcan que este es una línea prioritaria que debe tenerse en cuenta en la planificación y previsión de los servicios. Y esto debería ser mandatorio para que ellos desarrollen y hagan un plan de cómo darle mantenimiento a reparar su empresa, reparar su planta, mejorarla pero a la vez tiene que proveerle recursos porque el cambio de tubería y todo el mantenimiento necesita recursos entonces es importante que la iniciativa parta de las autoridades que tienen la visión que deberían tener la visión clara de esto, yo encuentro muy complicado que lo puedan hacer como iniciativa individual, porque como he referido no es una de las prioridades que ellos tienen.

**I: ¿Usted cómo observó La labor del Minsa durante la pandemia respecto del abastecimiento de oxígeno?**

E: Hizo lo que pudo con nosotros.

**I: Los acompañó en el proceso de, digamos, la implementación de plantas o el establecimiento de esta de este puente aéreo, tal vez luego con la cuestión de hacer un diagnóstico de las demandas o algo**

E: Ellos la necesidad era en todo el País, así que abastecían hasta donde era posible entonces su abastecimiento fue valioso pero insuficiente.

**I: Eh, bueno, no tengo más preguntas como tal, ¿pero no sé si usted quisiera hacer algún comentario final o agregar algo que yo no le he preguntado que considere usted que es importante para el estudio?**

E. No, no tengo mayores comentarios, solo decir que, si volviéramos a enfrentar una epidemia, ese tipo las circunstancias serían muy parecidas, quizás un poco o en algún grado una mejor respuesta, pero igual colapsaríamos en nuestra necesidad de oxígeno.

**I: Bueno, esperemos que no ocurra eso nuevamente porque lo que vimos fue bastante difícil, pero bueno, también pensar en que tenemos que estar preparados para todo.**

E: Así es.

**I: Bien, muchas gracias, doctor, ha sido un gusto poder conversar con usted en este momento voy a parar la grabación.**
