# Supplementary material for: Challenges in the medical oxygen ecosystem of Peru: A political economy analysis
Source: PLOS Glob Public Health. 2025 Dec 19;5(12):e0005667. doi: 10.1371/journal.pgph.0005667 (PMC12716698; doi:10.1371/journal.pgph.0005667)
Supplement: Attachment — Submitted filename: Response to Reviewers.docx [file pgph.0005667.s008.docx]

Lima August 5, 2025

PLOS Global Public Health

PGPH-D-025-00102

Dear Editors:

Thank you so much for reviewing our manuscript entitled: Challenges in the medical oxygen ecosystem of Peru: a political economy analysis. Please find in this letter the responses to each point raised by the editor and reviewers.

We are also downloading a revised version of the manuscript (Revised manuscript with Track changes) and an unmarked version of the manuscript.

Thanks for considering the manuscript for publication.

Patricia J. Garcia

**Journal Requirements:**

    1. **Your current Financial Disclosure states,** **“The case study documented in the article was supported by an award to Makerere University School of Public Health from the Meeting Targets and Maintaining Epidemic Control (EpiC) Project – FHI 360 (prime), made possible by the generous support of the American people through the US Agency for International Development (USAID) contract no. 7200AA19C00002. Cayetano Heredia University, Lima, Peru, provided salary support to PJG, JMG and LN while Makerere University provided salary support to FEK and FS, Every Breath Counts Coalition provided salary support to LG and Karolinska Institute provided salary support to CK.”. However, your funding information on the submission form indicates that you received funding from “FHI 360”. Please indicate by return email the full and correct funding information for your study and confirm the order in which funding contributions should appear. Please be sure to indicate whether the funders played any role in the study design, data collection and analysis, decision to publish, or preparation of the manuscript.**

Apologies for the confusion. We have simplified and clarified the statement as follows:

“The case study documented in the article was supported by an award from the US Agency for International Development (USAID) contract no. 7200AA19C00002. Cayetano Heredia University, Lima, Peru, provided salary support to PJG, JMG and LN while Makerere University provided salary support to FEK and FS, and Karolinska Institute provided salary support to CK. The funders played no role in the study design, data collection and analysis, decision to publish, or preparation of the manuscript”.

**2. In the online submission form, you indicated that The datasets used and/or analyzed during the current study are available from the corresponding author upon reasonable request.**

**All PLOS journals now require all data underlying the findings described in their manuscript to be freely available to other researchers, either**

**1. In a public repository,**

**2. Within the manuscript itself, or**

**3. Uploaded as supplementary information.**

**This policy applies to all data except where public deposition would breach compliance with the protocol approved by your research ethics board. If your data cannot be made publicly available for ethical or legal reasons (e.g., public availability would compromise patient privacy), please explain your reasons by return email and your exemption request will be escalated to the editor for approval. Your exemption request will be handled independently and will not hold up the peer review process, but will need to be resolved should your manuscript be accepted for publication. One of the Editorial team will then be in touch if there are any issues.**

We understand the policy of PLOS. The data ( interview transcriptions ) is provided now as supplementary information.

**3. We have noticed that you have uploaded Supporting Information files, but you have not included a list of legends. Please add a full list of legends for your Supporting Information files after the references list.**

We have added the full list of legends for our supporting information, after the references list.

4. **Add more details on the problem-based PEA approach and explain why this approach is suitable for your work. In addition, the references used for this (i.e., 17 and 18) approach doesn’t seem to be appropriate. Please add relevant references such as:**

- **Fritz VB. 2014. Problem-Driven Political Economy Analysis: The World Bank’s Experience. Washington, DC: The World Bank**
- **Harris D. 2013. Applied political economy analysis: a problem-driven framework. Overseas Development Institute – methods and resource. Overseas Development Institute.**

Thank you so much for your suggestions. We have added the explanation in the methods and discussion and added the recommended references.

5. - **Line 139: “Each document was reviewed by two independent readers”. I think you mean “two independent reviewers”.**

Thank you, we have corrected the sentence.

6. **Please add more details on the study participants/interviewees (n=13). You can add a summary characteristics table, including age/age range, role, gender, years of experience etc.**

We have added Table 1 and included some relevant information within the text . However, we were cautious about providing too much detail, as it could increase the risk of identifying participants and compromise confidentiality.

*A total of 13 interviews were conducted with stakeholders from national and provincial governments, civil society, the private sector, healthcare providers, medical professional associations, and multilateral agencies. Most participants were aged 45 and older, approximately one-third were women, and the majority had 20 to 30 years of professional experience (Table 1)*

7. **Currently the quotations in the results are referenced generically as ‘Health provider’ or ‘national government’. It is common to use participant ID or pseudonym to reference quotations.**

We have added the codes, according to what is now shown in table 1.

**8. Also, please state the potential limitations of this study at the end of the discussion.**

We have added the following paragraph:

Potential limitations of the study include the relatively small number of key informant interviews (n=13), which, while diverse in stakeholder representation, may not fully capture the breadth of perspectives across all regions or levels of the Peruvian health system. Additionally, the study relied heavily on secondary sources such as grey literature and publicly available policy documents, which may be incomplete or biased. The retrospective nature of the analysis, particularly regarding the COVID-19 pandemic response, may also introduce recall bias among interviewees. Furthermore, while the political economy analysis provides rich contextual insights, its findings may be less generalizable to other settings without similar political, regulatory, or market dynamics.

**Reviewer #1: Great paper on an important subject! National oxygen planning is critical, as is equitable distribution to rural areas. It is also complex, political, and hard to do. This paper really captures that well.**

Thank you !

**9. Can you provide more clarity on Table 1? The "Type of oxygen" column is confusing. It mixes production method, purity level, and distribution method. LOX producers can distribute oxygen in gaseous cylinders or in liquid oxygen tanks. For OxyCusco, Oxigeno Iquitos, Oxygen Loreto, Oxigeno Tao, or Villabolos Rufino, what is the mode of production? 93 or 99% is the purity, but cylinders can have 99% purity. Maybe table should be separated out to show purity level, method of production (LOX, PSA, VSA, etc), distribution method (LOX tanks, cylinders, etc)?**

Thank you very much. Now the table is table 2, we have added the columns and the information suggested and hopefully it is clearer.

**10. (p. 8), 235 Are you sure there was enough oxygen at periphery hospitals? Looks like only Lima levels were established via a Lima source (although I could be mistaken here). Often, levels are low without facilities realizing until health worker training highlights needs.**

You are absolutely right—we cannot be certain there was enough oxygen. In sentence 235, we stated, 'according to the interviews...'; however, to emphasize the lack of objective data, we have revised the text accordingly.

*"We found no objective data on pre-pandemic oxygen demand or supply levels, particularly in peripheral hospitals. Interviewees reported no awareness of shortages, but this may reflect limited monitoring and under-recognition of oxygen needs in some settings."*

**11. So important to highlight transportation and regional disparities like on line 52 of page 9. Glad this is here.**
 Thank you very much!

12. **Individually procuring oxygen may have increased costs, but cost may not have been the only driver. Often, individual procurement drastically reduces the time to get emergency supplies. In my experience, often going through national procurement systems can take months or years to procure items that are needed immediately. P. 13 291**
You are right, we have edited the text to add : -likely intended to simplify processes and expedite access to critical supplies—.

**13. p.15 402 Suggests that lack of maintenance plan may be due to corruption. Could it also be that most plants were donated without formal maintenance plans? This is a problem we see across the globe. Procuring spare parts across multiple disparate companies is hard. Many hospitals don’t know how to budget for spare parts, because this ongoing costs are not given at the time of installation. It could be corruption, but this is happening globally, and it is partially because donors aren’t planning for 6 months after installation. This suggests lack of foresight and planning more than malice, in my opinion.**

We are reporting what the interviewees told us. What the text of the manuscript says is
“Additionally, some believe that maintenance delays might be linked to potential corruption in medical oxygen procurement”.

We agree with you about the importance of maintenance plans. This section starts by saying “ The process of donating, distributing, and implementing oxygen plants in Peru was marked by opportunistic actions without plans for maintenance or replacement”.

**14. I see the value of a national oxygen system, but I wonder if there may be a role for the suppliers and/or the private sector. Could existing plants/suppliers partner with the national oxygen system to diversify oxygen sources (less risk of failure), while a national system negotiates more regular delivery and lower price points?**

We agree with you. We envision a **National Oxygen System** as coordinated, government-led framework that oversees the planning, production, procurement, distribution, quality control, and maintenance of medical oxygen across all levels of the health system. Its primary goal is to ensure equitable, reliable, and sustainable access to medical oxygen—especially during public health emergencies.

We have edited the text to make this clearer. It definitely includes suppliers and the private sector.

**Reviewer #2: Reviewer comments:**

**15. In Financial Disclosure, provide clarity on the following statement, “Every Breath Counts Coalition provided salary support to LG”, seems like an author is missing.**

Thank you for pointing out that error. We have erased that sentence. Every Breath Counts Coalition did not provide any support to this study.

**16. Line 104: “lower respiratory infections” should be “lower respiratory tract** infections”.

Thank you, we have edited accordingly.

**17. Line 104 to Line 107: If the data (in %) is available for lower respiratory tract infections and non-communicable diseases, it should be stated here like it has been done for chronic respiratory conditions (4.6%).**

Thanks so much for the comment, we have edited the paragraph for clarity and we have added the percents suggested.

**18. Line 201:For better understanding, in Table 1, a column should be added which mentions the process/technology used for oxygen manufacturing such as ‘cryogenic distillation’ for LMO for 99% pure oxygen, or ‘PSA/VSA/VPSA’ technology for 93% pure oxygen?**

According to the comment from reviewer 1 and the data available, we have improved the table which is now table 2.

**19. Also, maybe add another column to indicate if the Company is LMO manufacturer only, LMO-based refiller (cylinder refiller), or both. Also, it would be great to include to include another column to highlight the form in which oxygen is stored when manufactured. For e.g., large LMO manufacturers (capacity greater than 50 MT/day) will store it in liquid form (99% pure), small LMO manufacturers (4-20 MT/day) would store in gaseous form (99% pure) and PSA/VSA/VPSA technology plants will store it in gaseous form (93% pure). Also, if data available, daily manufacturing capacity in case of LMO and PSA/VSA/VPSA manufacturers should be included and if it is an LMO-based refiller, may be their daily refilling capacity could be included.**

Unfortunately, we were not able to get such detailed information .

**20. Line 226: “rechargeable” should be changed to “refillable”.**

Done.

**21. Line 252-253: “Most high-purity liquid oxygen plants are located along the central coast of Peru, while the few PSA plants in the Andean and jungle regions produce only limited quantities of gaseous oxygen at 93% purity”. Are the high purity plants LMO plants and 93% purity plants PSA plants? If yes, it could be specified as shown above.**

Done. Now the text reads:

Most high-purity liquid medical oxygen (LMO) plants are located along the central coast of Peru, while the few plants in the Andean and jungle regions are pressure swing adsorption (PSA) plants that produce only limited quantities of gaseous oxygen at 93% purity

**22. Line 254: “High-purity packaging facilities (booster compressor system)”, I believe the author is talking about facility where liquid medical oxygen is packed into gaseous cylinders using a booster compressor system. If yes, please include ‘booster compressor system’ in bracket as shown above.**

Thank you for the comment. We have edited as suggested.

**23. Line 257: Is the author talking about lack of LMO tankers to move LMO from manufacturing sites to hospitals with LMO tanks and LMO-based refillers for cylinder refilling? If yes, please clearly specify that. Or were there also not enough normal trucks/vehicles to move the 1500 L and 7000 L gaseous cylinders?**

We have edited the text for clarity as follows:

During the pandemic, these inequalities became evident as Andean and jungle regions faced severe shortages—not only due to limited local oxygen production but also because of inadequate distribution infrastructure. This included a lack of both LMO tankers and vehicles for transporting gaseous oxygen cylinders, as well as poor road conditions in the highlands and the absence of road access to key jungle cities like Iquitos.

24. **Line 323: “isotanks” should be “LMO tanks and LMO tankers”.**

Done

**25. Line 355: “procure and implement” should be “procure, install and commission”.**
Done

**26.Line 381: “496 oxygen plants” should be “496 PSA oxygen plants”.**

Done

**27. Line 385: “donating, distributing, and implementing oxygen plants” could be changed to “donating, distributing, installing and commissioning PSA oxygen plants”.**

Done
**28. Line 469: “< >” should be changed to “>”.**

Done, thanks for catching that typo error!.

**29. Line 548: “Every Breath Counts Coalition provided salary support to LG”, may have to be removed.**

Done.  **30. Between Lines 385 and 423, if the authors feel appropriate, they could add the following points about why PSA plants are not being used as much:**

**o Myths about PSA plants being too expensive. There is literature on it from India.
o Clinicians not preferring PSA plants 93% oxygen as they feel 99.99% purity is what is needed, may be studies comparing clinical outcomes of 93% pure oxygen vs 99% pure oxygen should be conducted to address this concern.**

**o PSA plants remove dependence on third party vendors which impacts under the table income of procurement managers.**

**o PSA plants operations and maintenance need more technically experienced staff which is not available.**

**o PSA plants need continuous electricity, voltage stabilisers and diesel generator set for back up (which can be very expensive to run depending on the diesel prices in the country). Any evidence on using solar power source to provide uninterrupted electricity for their operations?**

**o PSA plants are sensitive to heat, humidity, and altitude, which affect the purity.
o PSA plants donated from overseas have no local supply chain of spare parts and once they breakdown, they become defunct as there is no local spare parts industry to fill the gaps.**

Thank you so much for these interesting points about PSA plants and its use. The paragraph between lines 385 and 423 are still results from the literature review about Peru and the interviews, so we don’t think it would be appropriate to add these points there. However, we have added two paragraphs regarding these issues to the discussion.

**31. Moreover, it should be highlighted that if PSA plants are left unused for over 2 months, the zeolites (nitrogen adsorbing material) get damaged and replacing them alone costs over 50% of the PSA plant procurement cost. If PSA plant is maintained well, the zeolites should last up to 7 years without issues. Therefore, if the demand is low and they are not required to be used, they should be switched on once every 2-4 weeks and run for about 8-10 hours and then switched off, and this should be repeated until they are used more regularly. To increase their utilisation, booster compressors could also be used to fill gaseous cylinders which could be supplied to nearby facilities (in rural areas) in hub-spoke model. However, to install booster compressors on hospital premises may require necessary regulatory approvals from safety organisations as pressures for refilling are as high as 250 bar which can be lethal if done without training and supervision. Are there regulations for PSA plants being used for refilling cylinders with booster compressor systems?**

Thank you for your comments. We have added in the discussion the following : “Notably, if a PSA plant remains unused for over two months, the zeolites (nitrogen-adsorbing material) can be damaged—replacing them costs over 50% of the plant’s value. With proper maintenance, however, zeolites can last up to seven years”.

There are two regulatory documents which refer to PSA plants in Peru:

1. REGULATIONS OF LAW No. 31113, THE LAW THAT REGULATES, AUTHORIZES, ENSURES, AND GUARANTEES THE USE OF MEDICAL OXYGEN IN PUBLIC AND PRIVATE HEALTH FACILITIES NATIONWIDE <https://sni.org.pe/wp-content/uploads/2021/03/APRUEBAN-EL-REGLAMENTO-DE-LA-LEY-N%C2%B0-31113.pdf>
2. GUIDELINES FOR INVESTMENTS IN MEDICAL OXYGEN GENERATING PLANTS AND COMPLEMENTARY ASSETS MANAGED BY LOCAL GOVERNMENTS <https://www.minsa.gob.pe/Recursos/OTRANS/08Proyectos/2020/LINEAMIENTOS%20PARA%20INVERSIONES%20DE%20PLANTAS-GENERADORAS-OXIGENO-ACTIVOS-COMPLEMENTARIOS-GL.pdf>)

But we haven’t found anything specifically about regulations for PSA plants being used for refilling cylinders with booster compressor systems.

**32. The authors may also talk about a new French technology for on-site oxygen production, the ION Ionic Oxygen Generator, has been developed by NOVAIR in collaboration with NASA. This generator produces ultra-pure oxygen directly from ambient air, offering a reliable, compact, and user-friendly alternative to traditional oxygen supply methods. The ION generator can produce oxygen with purity levels of greater than 99.99%.**

Thank you so much for the information, but we think that goes beyond the intention of the manuscript.

**Some other points the authors can consider including to improve the understanding of the reader:**

**33. Talk about availability of LMO tanks at the facilities and LMO tankers to move the liquid oxygen from manufacturing site to the hospitals with LMO tanks or LMO based refillers.**

As requested in 23. We have added the information of the limitations regarding distribution associated to tanks and tankers.

**34. Any new installation of LMO tanks at hospitals during or post COVID-19? We see that 496 PSA plants were installed. And how about procurement of any new LMO tankers to move the liquid oxygen from one place to another?**

There was no procurement of LMO tankers to move liquid oxygen as far as we know. “The investments and donations were mainly related to PSA plants, cylinders and some oxygen supplies (see line 387). We have added the following in line 393: We found no information regarding investments or donations related to LMO tanks or LMO tankers (to improve transportation of LMO)”.

**35. Any information of any liquid argon or nitrogen tankers being converted to LMO tankers to meet the demand?**

We did not collect such technical information.

**36. Any information on converting industrial gaseous oxygen cylinders to medical oxygen gaseous oxygen cylinders?**

No. None of the interviewers mentioned the issue, and we didn’t find anything regarding that in the review of the literature (gray or academic).

**37. Any use of IoT devices to monitor the consumption of oxygen from LMO tanks to notify the LMO supplier on refilling?** **Any use of IoT devices to monitor the utilisation of PSA plants?**

We did not explore this specific point during the study. However, we recently asked a technical officer at the Ministry of Health, and he stated that he does not know whether IoT devices are being used to monitor oxygen consumption or notify suppliers.

**38. Role of portable oxygen concentrators for home-care use and hospital use (especially in secondary and primary care facilities) have not been discussed. Are they not available or used in Peru?**

Before the pandemic, oxygen concentrators were scarce in the country. During the pandemic, there were both donations and purchases that increased their availability. Our study focused primarily on oxygen provision for the most severe cases, where high-flow or hospital-grade oxygen was required. This is noted as a limitation in our current work, as it affects the broader understanding of the oxygen ecosystem.

**39. How did the country fair on availability of oxygen concentrators, ventilators, HFNC, CPAP and BiPAP devices? I believe there should not have been any shortage of patient monitors, ABG machines and various types of pulse oximeters.**

Although important information, it was beyond the scope of the manuscript. We have acknowledged this as a limitation in our current work.

**40. Are all the hospitals equipped with medical gas pipelines systems and manifolds? This a must have for PSA plants and LMO tanks.**

No, not all hospitals are equipped with medical gas pipeline systems. The Ministry of Health has been working to update its inventory, but currently, there is no comprehensive data available on the nationwide status of these systems

**41. Was there no black marketing or hoarding of oxygen cylinders or concentrators?**

Yes, there was. We have edited a paragraph for clarity and added quote related to that (line 303-305) and 312-315. (version with track changes).

**42. Was industrial supply redirected for medical use during COVID-19?**

Not really. As mentioned in lines 215 and 216 , In Peru there are two main business groups producing industrial oxygen for the mining and metallurgical sectors (their primary clients), and also medical oxygen. In the early months of the pandemic, industrial suppliers initially continued delivering oxygen despite reduced operations. However, as economic activities resumed, companies shifted back to industrial oxygen production and were unable to meet the growing demand for medical oxygen. We have added one paragraph related to this in the manuscript (294-296).
