# Supplementary material for: Challenges in the medical oxygen ecosystem of Peru: A political economy analysis
Source: PLOS Glob Public Health. 2025 Dec 19;5(12):e0005667. doi: 10.1371/journal.pgph.0005667 (PMC12716698; doi:10.1371/journal.pgph.0005667)
Supplement: Attachment — Submitted filename: Response to Reviewers Nov 2025.docx [file pgph.0005667.s009.docx]

Lima November 4, 2025

PLOS Global Public Health

PGPH-D-025-00102

Dear Editors:

Thank you so much for reviewing our manuscript entitled: Challenges in the medical oxygen ecosystem of Peru: a political economy analysis. Please find in this letter the responses to each point raised by the editor and reviewers.

We are also downloading a revised version of the manuscript (Revised manuscript with Track changes) and an unmarked version of the manuscript.

Thanks for considering the manuscript for publication.

Patricia J. Garcia

**Reviewer #1:**

**The revisions look good. Very glad to see more of the oxygen system clarified. Oxygen regulations are an important part of the system and have a huge impact on availability. The problems of national oxygen distribution are also key, and more literature is needed on these topics.**

**Two small proof reading comments:**

1. **p. 11, Line 314 doesn't quite make sense**

Thank you for the comment, we are deleting the sentence.

**2. p. 14 &15, Some exact sentences repeat in different paragraphs "Regional barriers...maintaining plants."**

Thanks so much again!

Indeed, we found duplications in line 427, page 14 and line 438 page 15. We have eliminated the paragraph on page 15 and edited a little the paragraph in page 14.

**3.A question I still have: While a National Oxygen System would certainly help with lack of data and clear regulations, how can it be ensured that a National Medical Oxygen System would avoid the same pitfalls of long approval times, national politics, and lack of distribution to politically less-important geographies if it is a government agency?**

**Would it have the power to ensure budget? Are there guidelines or specific by-laws that would ensure efficient and reliable access, so that parallel private oxygen acquisition systems are not needed and do not emerge? Some of this may be outside the scope of this text, but could be great future research.**

We thank the reviewer for this insightful comment. We agree that the establishment of a National Oxygen System would face potential challenges similar to those that affected previous centralized mechanisms, including bureaucratic delays and political influence. We have now acknowledged this point in the Discussion and highlighted the need for clear governance frameworks, accountability mechanisms, and sustainable financing to ensure such a system’s effectiveness and equity.

**Reviewer #2:**

1. **Line 66: “lower respiratory infections” should be “lower respiratory tract infections”. No further review is needed from my end once this final comment is addressed.**

We have edited accordingly. Thank you!

**Regarding the journal requirement regarding figure resubmissions, we uploaded the actual figures into the program and got this answer for both: Image complies with specifications. So, we left the figures that we uploaded at the submission. Hopefully that is ok.**
