# Supplementary material for: Challenges in the medical oxygen ecosystem of Peru: A political economy analysis
Source: PLOS Glob Public Health. 2025 Dec 19;5(12):e0005667. doi: 10.1371/journal.pgph.0005667 (PMC12716698; doi:10.1371/journal.pgph.0005667)
Supplement: Attachment — Submitted filename: 2Response to Reviewers Nov 2025.docx [file pgph.0005667.s010.docx]

Lima November 28, 2025

PLOS Global Public Health

PGPH-D-025-00102

Dear Editors:

Thank you so much for reviewing our manuscript entitled: Challenges in the medical oxygen ecosystem of Peru: a political economy analysis. Please find in this letter the responses to each point raised by the editor and reviewers.

**Journal Requirements:**

1. Please amend your detailed online Financial Disclosure statement. This is published with the article. It must therefore be completed in full sentences and contain the exact wording you wish to be published.

a) State the initials, alongside each funding source, of each author to receive each grant. For example: “This work was supported by the National Institutes of Health (####### to AM; ###### to CJ) and the National Science Foundation (###### to AM).”

b) State what role the funders took in the study. If the funders had no role in your study, please state: “The funders had no role in study design, data collection and analysis, decision to publish, or preparation of the manuscript.”

For more information, please go to our submission guidelines:

https://journals.plos.org/globalpublichealth/s/submission-guidelines#loc-financial-disclosure-statement

*We have addressed the comment. Now the paragraph reads as follows:*

*“This work was supported by the US Agency for International Development (USAID) under contract no. 7200AA19C00002 to FEK. Cayetano Heredia University (Lima, Peru) provided salary support to PJG, JMG, and LN; Makerere University provided salary support to FEK and FS; and the Karolinska Institute provided salary support to CK. The funders had no role in study design, data collection and analysis, decision to publish, or preparation of the manuscript”*

2. Please ensure that the funders and grant numbers match between the Financial Disclosure field and the Funding Information tab in your submission form. Note that *the funders must be provided in the same order in both places as well.*

*Ok, done*

3. If the reviewer comments include a recommendation to cite specific previously published works, please review and evaluate these publications to determine whether they are relevant and should be cited. There is no requirement to cite these works unless the editor has indicated otherwise.

*There are no comments from the reviewers in the email sent to us.*

4. Please review your reference list to ensure that it is complete and correct. If you have cited papers that have been retracted, please include the rationale for doing so in the manuscript text, or remove these references and replace them with relevant current references. Any changes to the reference list should be mentioned in the rebuttal letter that accompanies your revised manuscript. If you need to cite a retracted article, indicate the article’s retracted status in the References list and also include a citation and full reference for the retraction notice.

*We have reviewed it and it is ok.*

**Additional Editor Comments:**

Thank you for addressing reviewers concerns. Both reviewers have some additional minor comments. Please address them in your manuscript.

*There are no comments from the reviewers in the email sent.*

Thanks so much

Patricia J. Garcia
